# Opportunities and barriers to implementing antibiotic stewardship in low and middle-income countries: Lessons from a mixed-methods study in a tertiary care hospital in Ethiopia

**DOI:** 10.1371/journal.pone.0208447

**Published:** 2018-12-20

**Authors:** Gebremedhin Beedemariam Gebretekle, Damen Haile Mariam, Workeabeba Abebe, Wondwossen Amogne, Admasu Tenna, Teferi Gedif Fenta, Michael Libman, Cedric P. Yansouni, Makeda Semret

**Affiliations:** 1 School of Pharmacy, College of Health Sciences, Addis Ababa University, Addis Ababa, Ethiopia; 2 School of Public Health, College of Health Sciences, Addis Ababa University, Addis Ababa, Ethiopia; 3 Department of Pediatrics and Child Health, School of Medicine, Addis Ababa University, Addis Ababa, Ethiopia; 4 Department of Internal Medicine, School of Medicine, Addis Ababa University, Addis Ababa, Ethiopia; 5 Department of Medicine, Infectious Diseases and Microbiology, and JD MacLean Centre for Tropical Diseases, McGill University Health Centre, Montreal, Quebec, Canada; Universitat Autonoma de Barcelona, SPAIN

## Abstract

**Background:**

Global action plans to tackle antimicrobial resistance (AMR) include implementation of antimicrobial stewardship (AMS), but few studies have directly addressed the challenges faced by low and middle-income countries (LMICs). Our aim was to explore healthcare providers’ knowledge and perceptions on AMR, and barriers/facilitators to successful implementation of a pharmacist-led AMS intervention in a referral hospital in Ethiopia.

**Methods:**

Tikur Anbessa Specialized Hospital (TASH) is an 800-bed tertiary center in Addis Ababa, and the site of an ongoing 4-year study on AMR. Between May and July 2017, using a mixed approach of quantitative and qualitative methods, we performed a cross-sectional survey of pharmacists and physicians using a pre-tested questionnaire and semi-structured interviews of purposively selected respondents until thematic saturation. We analyzed differences in proportions of agreement between physicians and pharmacists using χ^*2*^ and fisher exact tests. Qualitative data was analyzed thematically.

**Findings:**

A total of 406 survey respondents (358 physicians, 48 pharmacists), and 35 key informants (21 physicians and 14 pharmacists) were enrolled. The majority of survey respondents (>90%) strongly agreed with statements regarding the global scope of AMR, the need for stewardship, surveillance and education, but their perceptions on factors contributing to AMR and their knowledge of institutional resistance profiles for common bacteria were less uniform. Close to 60% stated that a significant proportion of *S*. *aureus* infections were caused by methicillin-resistant strains (an incorrect statement), while only 48% thought a large proportion of gram-negative infections were caused by cephalosporin-resistant strains (a true statement). Differences were noted between physicians and pharmacists: more pharmacists agreed with statements on links between use of broad-spectrum antibiotics and AMR (*p*<0.022), but physicians were more aware that lack of diagnostic tests led to antibiotic overuse (*p*<0.01). More than cost, fear of treatment failure and of retribution from senior physicians were major drivers of antibiotic prescription behavior particularly among junior physicians. All respondents identified high turnover of pharmacists, poor communication between the laboratory, pharmacists and clinicians as potential challenges; but the existing hierarchical culture and academic setting were touted as opportunities to implement AMS in Ethiopia.

**Conclusions:**

This knowledge and perceptions survey identified specific educational priorities and implementation strategies for AMS in our setting. This is likely also true in other LMICs, where expertise and infrastructure may be lacking.

## Introduction

Antimicrobial resistance (AMR) and the decreasing effectiveness of antibiotics is leading to significant health and economic losses worldwide [1].

Low and middle-income countries (LMIC) in Sub-Saharan Africa and Asia carry the greatest burden of infectious diseases. They further have to contend with weak regulatory infrastructure, over-the-counter sales or counterfeit antimicrobials, and inappropriate prescription practices coupled with significant gaps in diagnostic testing and surveillance [2]. This had led many to predict that AMR will disproportionally impact populations living in LMICs [3, 4].

Global action plans to tackle AMR include the implementation of antimicrobial stewardship (AMS). While several studies have attempted to identify the strategies most likely to have beneficial effects [5], few have directly addressed the challenges to AMS implementation faced by LMICs. One recent study in a network of private hospitals in South Africa showed that substantial reductions in antibiotic use could be achieved by focusing on a few basic interventions led by pharmacists, suggesting that successful implementation of AMS is a function of organizational commitment and tailoring of existing systems and resources [6].

Prior to initiating a prospective longitudinal study of Hospital Associated Infections and AMR in a tertiary care hospital in Ethiopia, a setting considered “low-resource but moderate infrastructure” as defined in previously published criteria for assessment of laboratory infrastructure in LMICs [7], we conducted a point prevalence survey of antibiotic prescriptions among hospitalized patients, and noted that close to 80% of those hospitalized in medical or pediatric wards and 100% of those in an intensive care unit were on broad-spectrum antibiotics at the time of the survey; furthermore, less than 1% of patients had had microbiological workup prior to initiation of antibiotics (*Unpublished data*). We subsequently focused on improving microbiological diagnostic capacity through implementation of automated blood culture testing and systematic identification and antibiotic susceptibility testing of bacterial pathogens [8]. Contrary to our expectation that improved diagnostics would lead to more rational use of antibiotics, use of broad-spectrum antibiotics among in-patients did not decrease after microbiological testing became standardized and available. That year, annual antibiotic expenditures amounted to 9,853,453 Ethiopian Birr (447,885 USD)—higher than the year before and equivalent to 18% of the total pharmacy budget. Vancomycin alone accounted for 30% of the antibiotic expenditures, followed by meropenem (16.3%). Four antibiotics (vancomycin, meropenem, ceftazidime and ceftriaxone) accounted for 66% of the total antibiotic budget.

At the time of this study, Ethiopia had developed a national action plan to combat AMR [9]. Acknowledging the large increase in use of costly broad-spectrum antibiotics nationally in recent years, one of 5 strategic objectives outlined in the plan was to optimize use of antimicrobials through effective stewardship practices, with plans to pilot these programs in selected institutions then expand nationally–though a practical implementation guide was still in preliminary draft form. The objective of the current study was to explore some of the behavioral determinants that influence in-patient prescribing practices in Ethiopia, in order to better inform the design of practical but comprehensive AMS strategies for LMICs.

## Methods

### Study setting

Tikur Anbessa Specialized Hospital (TASH) is the largest teaching and referral hospital in Ethiopia. The hospital has 800 operational beds and services about 530,000 patients per year (about 200,000 inpatients, with an average length of stay of 9.3 days, and 330,000 outpatients). There are 80 pharmacists on staff, of which 5 are clinical pharmacists, and 1059 physicians (594 residents and 465 attending physicians).

### Study design and ethics

We used a concurrent mixed approach of quantitative and qualitative study designs. A cross-sectional survey was conducted among physicians and pharmacists working in TASH to assess general awareness about AMR and perceptions towards stewardship and other potential containment strategies. In-depth interviews of selected participants were also conducted to specifically explore perceptions of barriers and facilitators for successful implementation of AMS in LMICs. The study received ethics approval from the Institutional Review Board of the College of Health Sciences, Addis Ababa University *(Protocol Number*: *045/15/IM)* and informed verbal consent was obtained from all study participants. Responses and identities of key informants and survey participants were kept strictly confidential.

### Recruitment of survey participants

All physicians from the departments of surgery, pediatrics, hematology-oncology, emergency, gynecology/obstetrics, and medicine were approached to participate in the study during Morning Sessions and Grand Rounds, and pharmacists were approached at their worksite during working hours. A self-administered questionnaire was distributed to all study participants for the quantitative survey.

Participants for the qualitative interview were selected using a purposive sampling technique, aimed at identifying key informants who would best provide an in-depth understanding of prescribing patterns and potential antimicrobial stewardship implementation solutions. We used the following criteria for selection: i) physicians with a reputation of being “thought leaders”, influential or authoritative in the institution; ii) physicians with expertise in infectious diseases; ii) professionals who “frequently” prescribe (eg: general surgeons, hematologists-oncologists) or dispense antibiotics, and iv) physicians or pharmacists who indicated they had reflected on the concept of antimicrobial stewardship at least to some extent in the recent past.

### Data collection and management

#### Quantitative survey

We used a pre-tested semi-structured questionnaire adapted from a similar study [10]. This 56-item questionnaire was designed to collect information on respondents’ attitudes towards antimicrobial prescribing/dispensing practices, their knowledge on the scope and key contributors of AMR, their perceptions on AMS and their rating of potential interventions to control AMR (S1 Table).

#### Qualitative survey

Qualitative data was collected through semi-structured interviews with flexible probing techniques. The interviews were conducted between May and July 2017, in the offices of the participants or the first author, and were classified into four sections: (i) socio-demographic information; (ii) respondents’ view on the current use of antibiotics and factors contributing to misuse; (iii) perceived magnitude of AMR, and, (iv) barriers and enablers to implementing AMS in the hospital (S2 Table). The English version of the interview guide was translated to Amharic (national language of Ethiopia) (S1 document) and back translated to English to check its consistency before using the Amharic version.

The first author (GBG), a pharmacist and PhD candidate trained in advanced qualitative methods, conducted and audio-recorded all interviews in Amharic, transcribed the interviews verbatim then translated them to English. A random selection of 5 interviews were also re-transcribed and translated by a research assistant to verify the accuracy of the primary work. The interviews were held in either in the participants’ or GBG’s office and lasted from 15 to 74 minutes (mean duration 37±15 minutes).

Recruitment of participants continued until the lead investigators (GBG and MS) reached consensus on the predominant themes, meaning that they agreed no new information was emerging from the in-depth interviews and that there was significant redundancy in the responses.

### Data analysis

Quantitative survey: Data was coded and entered into SPSS version 21 for analysis. Descriptive statistics such as frequencies, percentages, mean and standard deviations was used to characterize the variables of interest. Frequency of degree of agreement to statements was reclassified into “agree” (strongly agree/agree), “disagree” (strongly disagree/disagree) and “neutral”. The *χ*^*2*^ and fisher exact tests with significance level of *p*< 0.05 were employed to explore differences in proportions of agreement between physicians and pharmacists.

Qualitative survey: Thematic analysis was applied to analyze each interview transcripts immediately after these were conducted [11]. The complete transcripts of the interviews were coded for repetitive key words, patterns of sentences or of sentence fragments using OpenCode 4.03 software (S2 document). Additionally, through line-by-line reading and rereading of transcripts, relevant concepts were organized into categories to allow themes to emerge. Key themes and findings were then shared with five randomly selected qualitative study participants to confirm that interpretations were reflective of their perceptions and experiences.

## Results

### Quantitative findings

#### Socio-demographic characteristics of respondents

**Fig 1:**
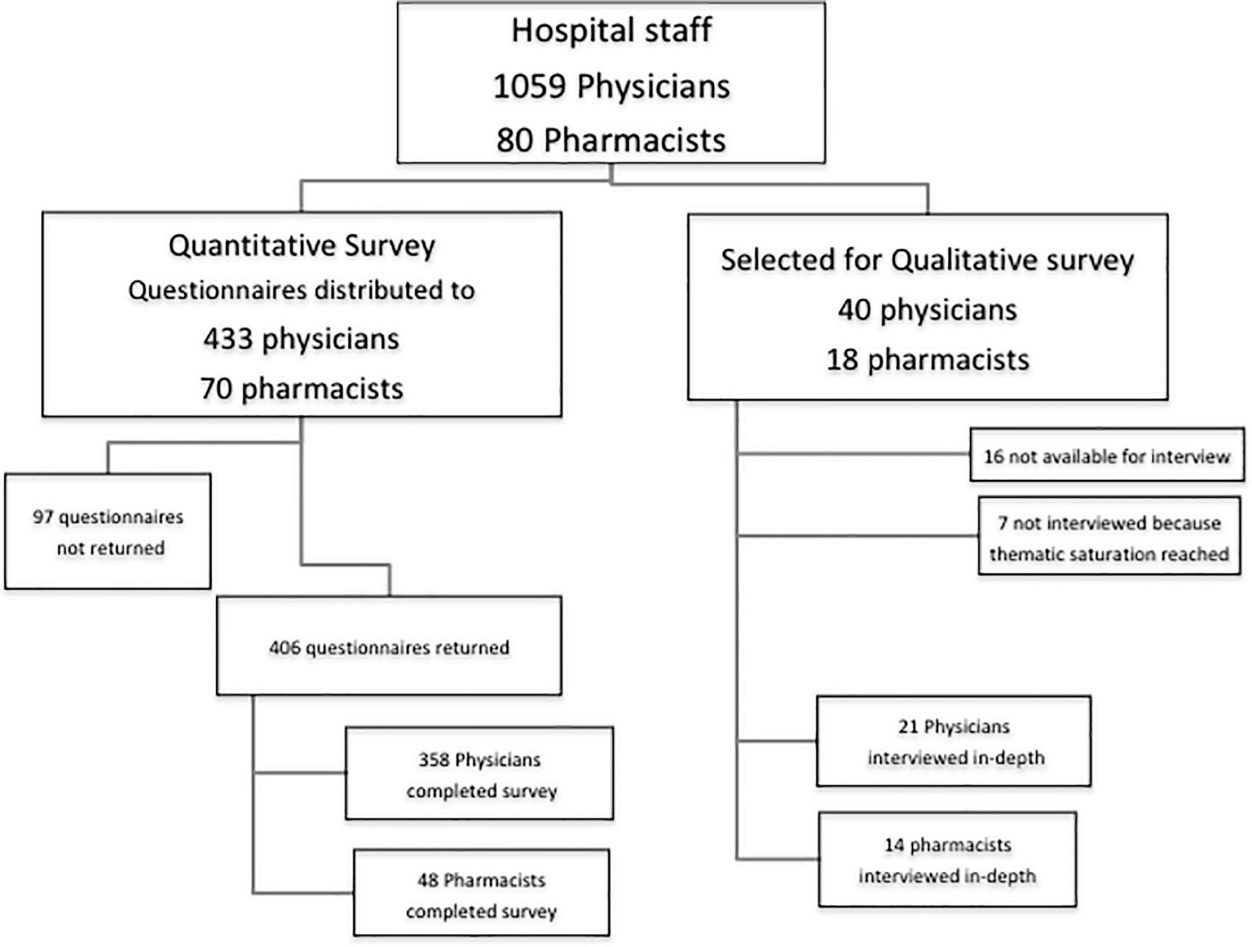
Flow chart of participant enrollment for the quantitative and qualitative surveys.

A total of 503 quantitative survey questionnaires were distributed and 406 (81%) were returned; 35 participants were interviewed in the qualitative analysis (Fig 1). The majority of respondents were physicians (88% and 60% in quantitative and qualitative surveys respectively) and male (78% and 83%). Participants in the qualitative survey were on average older (35 versus 28 years old), had more work experience (60% had worked more than 5 years, versus only 7% in the quantitative survey group), and prescribed antibiotics to more patients (on average 95 patients per week, versus 20 patients/week) compared with the participants of the qualitative survey (Table 1).

**Table 1:** Demographic characteristics of quantitative and qualitative study participants.

Characteristics		Respondents of Quantitative Survey (N = 406)		Respondents of Qualitative Interviews (N = 35)	
		n(%)	Mean ± SD (Range)	n(%)	Mean ± SD (Range)
**Age (in years)**			28.0 ± 4.0 (21, 56)		35.0±9.0 (25, 63)
**Gender**					
**Male**		305(78.2)		29(82.9)	
**Profession**					
	Physician	358(88.2)		21(60.0)	
	Pharmacist	48(11.8)		14(40.0)	
**Primary work area or unit**					
	Medicine (non-surgical)	67(16.5)		6(17.1)	
	Surgery	99(24.4)		1(2.9)	
	Pediatrics	61(15.0)		5(14.3)	
	Gynecology/Obstetrics	46(11.3)		2(5.7)	
	Emergency	13(3.2)		3(8.6)	
	Oncology	16(3.9)		4(11.4)	
	Rotation (among wards)	56(13.8)		-	
	Pharmacy	48(11.8)		14(40.0)	
**Work experience**			3.0±6.0 (1,25)		11.0±9.0(2,44)
	< 5 years	339(92.6)		14(40.0)	
	≥5 years	27(7.4)		21(60.0)	
Average number patients treated per week*			42.0±42.0 (2,250)		95.0± 35(10,150)
Average number patients treated with at least one antibiotic per week*			20.0 ±20.0 (2,150)		50.0±30(10,100)

*Respondents were only physicians

**Scope of antimicrobial resistance and key contributors:** Almost all survey respondents (>90%) perceived AMR as a global and national threat, though pharmacists were more likely to consider it an issue of local (institutional) concern. Overall, 66% agreed with the statement that patients are highly likely to develop drug-resistant infections during their hospital stay, and that AMR had a substantial impact on their practice.

Nearly two-thirds (58%) of the respondents thought that a significant proportion of local *Staphylococcal* isolates (>30%) are resistant to methicillin (MRSA) but only 48% thought that a high proportion (>30%) of gram-negative isolates are highly drug-resistant in their hospital (Table 2).

**Table 2:** Physicians’ and pharmacists’ perception on Antimicrobial Resistance (AMR) and contributing factors.

Statements		Proportion (%) of respondents who agree/strongly agree with each statement			
		ALL N = 406	Physicians N = 358	Pharmacists N = 48	*p-*value
Scope of antimicrobial resistance problem					
	AMR is a significant problem worldwide	94.6	94.1	97.9	0.254 ^a^
	AMR is a significant problem in my country*	91.7	90.6	100.0	0.012 ^a^
	AMR is a significant problem in my hospital*	85.6	84.3	95.6	0.041 ^a^
	AMR is a problem in my daily practices	68.0	66.5	79.5	0.066
	A patient is highly likely to develop drug-resistant infection during their hospital stay*	66.0	63.5	84.8	0.014
	Very high proportion (>30%) of gram negative infections are highly drug- resistant (resistant to all cephalosporins, and some are even resistant to carbapenems)	48.0	49.5	37.8	0.116
	Very high proportion (>30%) of *Staphylococcal* infections are resistant to methicillin	57.8	57.1	66.2	0.513
Beliefs on factors contributing to antimicrobial resistance in the study hospital					
	Inappropriate use of antibiotics is a major cause of AMR *	82.0	80.2	95.7	0.034
	Easy access to antibiotics without a prescription contributes to AMR *	84.3	82.4	97.9	0.003 ^a^
	Prescription of broad-spectrum antibiotics is directly linked to AMR *	63.6	61.2	82.2	0.022 ^a^
	Lack of adequate diagnostic tests leads to overuse of antibiotics thereby contributing to AMR *	64.4	66.4	48.9	0.010 ^***a***^
	Sporadic supply of antibiotics leads to interruptions of therapy thereby contributing to AMR	62.1	62.1	62.2	0.943
	Lack of close clinical follow-up during antibiotic use contributes to AMR*	60.2	57.2	82.6	0.003
	Patient demands and expectations increased overuse of antibiotics thereby contribute to AMR*	29.3	26.8	48.9	0.007
	Poor infection control practices by health professionals significantly contributes to increase AMR	65.9	65.7	67.4	0.124
	I suspect that antibiotics available in the hospital are of poor quality and contribute to AMR*	26.5	28.0	15.2	0.049
	The hospital performs adequate surveillance for drug resistant organism	8.3	8.5	6.8	0.461
	The hospital provides adequate staff education regarding antibiotic use and resistance	10.8	11.1	8.9	0.125

* Significant at p<0.05

^a^ If fisher exact test is employed, otherwise χ^2^ test is used.

With regard to participants’ beliefs on the causes of AMR, the vast majority (82%) agreed that inappropriate use and easy access to antibiotics were key contributors; only a small minority felt that the institution performed adequate surveillance and staff education on drug-resistant organisms (8% and 11% respectively). About 2/3 of respondents also agreed that lack of adequate diagnostic tests, sporadic supply of antibiotics, poor infection control practices and lack of close clinical follow-up were significant contributors to AMR. Only 26% pointed to poor quality antibiotics as significant factors in the development of AMR, while 30% felt that patient demands/expectations played a role (Table 2).

#### Antibiotic prescription/Dispensing practices

Close to one third (27%) of the physicians estimated that they had prescribed broad-spectrum antibiotics empirically for longer than 3 days for over 50% of their patients, while 35% stated they prescribed them for 10–50% of their patients in the previous week. The majority of respondents (85%) disagreed with the statement that results from the microbiology laboratory were communicated in a timely manner, and fewer than 35% stated they routinely check microbiology laboratory results to guide the choice of therapy. The majority (70%) agreed with the statement that they prescribe broad-spectrum antibiotics empirically because microbiology lab results are not available in a timely fashion. Most study participants agreed with the statement that their choice of antibiotics is highly influenced by cost considerations (80.3%) and availability of antibiotics (68.9%) (Table 3).

**Table 3:** Proportion of agreement on antibiotics prescription/dispensing practices.

Statements	Proportion (%) of respondents who agree/strongly agree with each statement			
	ALL N = 406	Physicians N = 358	Pharmacists N = 48	*p-*value
**Microbiology lab results are communicated to the health professionals in a timely manner**	15.9	14.8	25.6	0.213
**I routinely step down iv antibiotics to PO alternative antibiotics** *	60.7	63.6	35.0	0.002
**I routinely narrow antibiotics spectrum** *	38.5	38.9	34.6	0.002
Cost considerations for the patient affects my choice of antibiotics*	80.3	83.9	51.1	0.000
My choice of antibiotics is often influenced by the availability of the antibiotics rather than by the local antibiogram or by the etiologic cause of disease*	68.9	71.8	46.7	0.001
I routinely choose very broad-spectrum antibiotics empirically because most patients are infected with DRO*	36.6	35.3	47.6	0.012
I routinely choose very broad-spectrum antibiotics empirically because microbiology results are not available in a timely fashion*	68.9	71.4	47.6	0.003
**I routinely check microbiology laboratory results to guide my choice of antibiotics**	34.9	34.8	35.9	0.960
**In the past 7 days, I prescribed broad spectrum antibiotics for > 3 days for 10–50% of my patients**	34.3	34.3	*N/A*	-
**In the past 7 days, I prescribed broad spectrum antibiotics for > 3 days for more than 50% of my patients**	26.8	26.8	*N/A*	-

* Significant at p<0.05

*N/A*: Not Applicable as respondents were only physicians

#### Perceptions towards an antimicrobial stewardship program

The majority (>73.0%) of respondents agreed or strongly agreed with the statements that AMS would improve quality of care, reduce costs, and reduce the overall impact of AMR. Over 80% of respondents accepted the need for direct feedback on their antibiotic selection, and only a minority felt that AMS can be an obstacle to good patient care and override prescribers’ decision autonomy (Table 4).

**Table 4:** Perceptions of physicians’ and pharmacists towards implementation of an antimicrobial stewardship program (ASP) at Tikur Anbessa Hospital (TASH), Addis Ababa, Ethiopia.

Item	Mean* ±SD	Degree of agreement, n(%)		
		Strongly disagree/Disagree	Neutral	Strongly agree/Agree
**ASP improve quality of patient care**	4.22±0.96	19(5.4)	43(12.2)	290(82.4)
**ASP reduces antibiotic use overall and can result in cost savings**	4.06±0.99	24(6.7)	71(19.8)	263(73.5)
**ASP reduces duration of hospital stay and associated costs**	4.02±0.86	14(3.8)	70(19.2)	280(76.9)
**ASP reduce the problem of AMR**	4.13±0.82	13(3.6)	49(13.5)	301(82.9)
**ASP impact an institution’s nosocomial infection rates**	4.02±0.85	17(4.7)	71(19.6)	275(75.8)
**The hospital has the capacity to establish and implement an effective ASP**	3.72±1.06	39(10.9)	110(30.7)	209(58.4)
**I would like more feedback on my antibiotic selections**	4.10±0.83	17(4.5)	48(12.7)	312(82.8)
**ASP can be an obstacle to good patient care**	2.28±1.16	234(65.0)	67(18.6)	59(16.4)
**ASP override prescribers’ decision autonomy**	2.57±1.11	183(51.4)	97(27.2)	76(21.3)
**Infectious diseases experts that can provide guidance on antibiotic usage are available**	3.16±1.25	104(29.8)	94(26.9)	151(43.3)
**Pharmacists with sufficient training to provide guidance on antibiotics usage are available**	2.62±1.20	166(47.7)	100(28.7)	82(23.6)

*Mean of responses rated according to 1–5 scale, with 1 = strongly disagree; 2 = disagree; 3 = neither agree or disagree; 4 = agree; 5 = strongly agree

A little over half of the respondents (58%) agreed with the statement that the hospital has the capacity to implement an effective AMS, and that their individual efforts in AMS can significantly impact the issue of AMR. Nevertheless, there was less agreement on the availability of infectious diseases experts and trained clinical pharmacists to provide guidance on antibiotics.

#### Beliefs on potential interventions to reduce antimicrobial resistance

The majority (>83%) of respondents agreed that education, active participation from infection control, institutional guidelines, access to institutional antibiograms, and prospective audit and feedback interventions would be the most effective ways to reduce AMR; fewer than half felt that antibiotic cycling or formulary restrictions would be effective (Table 5).

**Table 5:** Physicians’ and pharmacists’ beliefs on potential intervention to combat AMR at Tikur Anbessa Hospital, Addis Ababa, Ethiopia.

Potential Interventions	Rank	Mean ±SD	Degree of agreement, n(%)		
			Definitively ineffective	Unsure	Definitively effective
Education on antimicrobial therapy to medical and pharmacy staff	1	2.91 ±0.35	8(2.0)	20(5.1)	367(92.9)
Active involvement of hospital infection prevention and control team	2	2.86 ±0.41	10(2.6)	33(8.5)	347(89.0)
Develop new institutional guidelines for empiric antimicrobial use	3	2.85 ±0.42	10(2.5)	38(9.6)	364(87.8)
Access to institution-specific antibiogram to treating teams	4	2.81 ±0.46	10(2.5)	56(14.2)	328(83.2)
Implementation of prospective audit and feedback	5	2.80 ±0.48	14(3.6)	52(13.2)	328(83.2)
Antibiotic restriction intervention	6	2.54 ±0.65	34(8.8)	109(28.2)	244(63.0)
Antibiotic cycling intervention	7	2.47 ±0.63	29(7.6)	144(37.8)	208(54.6)

### Qualitative findings

A total of 35 key informants (21 physicians and 14 pharmacists) were interviewed. The demographic characteristics of the participants are presented in Table 1.

#### Perceptions of antibiotics use

The majority (34 of 35) of the professionals interviewed voiced the concern that antibiotic misuse and overuse is widespread in the country. Physicians generally felt the problem was of greater magnitude and scope in community pharmacies and in private hospitals, where financial incentives were major drivers of antibiotic prescriptions, rather than in the public-sector hospitals:

“*Antibiotics are used like sugar or any other commodity*. *If you properly pronounce the name of any antibiotic*, *you can easily get it*. *Even the lay public [has] started to recognize the problem [*..*] one day*, *a child was prescribed Ceftriaxone*, *and his father expressed his frustration by saying [*..*] wait*, *is this medicine that should be prescribed to all patients like holy water*?*” (29 years male physician*, *3 years work experience)*.

*“The problem of antibiotic use is enormously high in private facilities where there is considerable patient pressure to prescribe more potent and costly antibiotics while in public hospitals*, *patients don’t exert pressure because most of them have low literacy and are poor*, *so they indisputably accept whatever you give them” (26 years male physician*, *2 years work experience)*.

*Most of the prescribing practice in the private sector is market oriented [*. . . .*] for instance*, *I never used ertapenem in this hospital but I see it is frequently overprescribed in the private hospitals” (36 years male physician*, *12 years work experience)*.

Most physicians confirmed they routinely prescribe broad-spectrum antibiotics and felt this practice was reasonable considering the severity of illnesses they treated. They admitted to frequent switching, combinations, and to using broad-spectrum antibiotics for surgical prophylaxis and continuing these post-operatively to prevent surgical site infections. Most physicians admitted they were rarely guided by microbiological results, and that patients remained on antibiotics for long durations with antibiotic cessation simply coinciding with discharge of the patient from hospital. Two of the physicians interviewed stated they use narrow spectrum antibiotics only when availability or costs are an issue for patients.

*“We frequently use broad-spectrum antibiotics for prophylaxis and for the post-op; generally guidelines recommend one or two doses but regardless of that we tend to prolong the duration even up to seven days or more*, *for fear that there could be infectious complications […] yes we know we are overusing them but again we don’t want to take risks nor miss any opportunities” (36 years female physician” 12 years work experience)*.

*“Since the possibility of acquiring multiple infections or resistance is high*, *we prescribe combinations of antibiotics*. *We don’t have hospital guidelines and our antibiotics prescribing practice isn’t guided by microbiology results*, *rather it is subjective mostly relying on personal experiences [*..*] Another challenge to inappropriate prescribing is the erratic supply of antibiotics” (39 years male physician*, *13 years work experience)*.

Pharmacists expressed that many physicians neglect to obtain detailed antibiotic histories, failed to adjust doses when indicated (particularly in pediatrics) and often omitted to provide a treatment plan. They note that junior physicians (interns and residents) are the group more likely to prescribe broad-spectrum antibiotics; they further speculate these practices are driven by poor knowledge, a tendency to follow antibiotic prescribing “trends” combined with weak communication between professionals. Of note, some speculated that fear of poor performance evaluations (a form of academic punishment) drives junior physicians to prescribe broad-spectrum antibiotics which they deem to be “safer”, rather than prescribing narrow spectrum antibiotics and thereby risk a negative comment on their training evaluations.

*“Poor follow-up and poor transcription/documentation practices are common problems. Sometimes physicians prescribe broad-spectrum antibiotics without checking history of previous medications or with no plan when to stop. I personally have seen patients on Meropenem and Ceftriaxone for forty days … When you ask for[12]means to correct, they blame one another [..] I encountered patients taking Ceftriaxone and Ceftazidime at the same time” (26 years male pharmacist, 4 years work experience)*.

*“Frontline prescribers are students (residents or interns) [*..*] They usually prescribe broad-spectrum antibiotics to avoid seniors’ retribution*, *especially if suspected pathogen is not covered*. *Most are concerned about their educational career instead of the risk to patien’s life*. *It is also very common to see trendy prescribing behavior especially if the antibiotic is new*, *like someone following fashion [*…*] Probably this might be attributed to pharmaceutical promoters’ influence” (26 years male pharmacist*, *4 years work experience)*.

#### Perceptions on the optimal approach to implementing AMS

Although only a minority of the interviewees could clearly describe an optimal AMS, most felt implementing a program with clearly defined goals and objectives, and detailed descriptions of the roles of key stakeholders was needed. They stated that antibiotics are resources that should be “audited, monitored and controlled”, with the same stringency regulating financial resources. All but 3 physicians felt that a strategy consisting of prospective audit and feedback *with* restrictions of certain antibiotics would be the most effective for Ethiopia.

*“Both [restriction and auditing] are quality improvement tools and it is better to adopt a mix of interventions*. *Auditing is very good intervention but resource-intensive and it might be difficult to apply in all wards*. *So*, *it is better to start auditing in selected wards and implement pre-authorization policy for selected antibiotics” (37 years male physician*, *13 years work experience)*.

#### Challenges to implementing AMS

All participants expressed the concern that AMS strategies are currently not *concretely* supported by institutional or national policies, and not uniformly implemented across health facilities in the country—making it more challenging to persuade prescribers concerned about prescription autonomy. They nevertheless underscored that resistance from prescribers would need to be handled through continuous discussions and engagement.

Weak laboratory infrastructure was touted as a major obstacle for implementation of AMS. Almost all physicians acknowledged they either did not send specimens or did not follow up on results because of delayed result reporting, and communication gaps between the laboratory and the treating teams. Insufficient infectious diseases expertise, a historical disconnect between pharmacists and physicians in the hospital, and high turnover of clinical pharmacists were identified as potential challenges.

#### Enabling factors to implementing AMS

The urgency of the global AMR crisis and the ongoing national initiative to develop an AMS implementation protocol were identified as important enabling factors. Survey participants pointed to the teaching environment and the existing prescribing hierarchy [interns-residents-senior consultants] as important advantages to implementing AMS.

*“Since I am rounding in a congested hospital with many patients having resistant pathogens, I personally can tell you how scared I am especially my children. And I can assure you this is a growing concern of many physicians and policymakers. So, if you came up with interventions known to decrease resistance, acceptance won’t be a problem [..] Even policymakers will support you, if not with money at least in terms of enforcing the policies (57 years male physician, 26 years work experience)*.

## Discussion

Low and middle-income countries (LMICs) are currently the largest consumers of antibiotics worldwide in terms of total tons of antibiotics. Though their *per capita* usage remains below that of wealthier nations, their total antibiotic consumption and rates per capita have been dramatically increasing in the past 15 years [13]. This phenomenon correlates closely with growth in incomes and gross domestic product per capita and underscores the critical importance of implementing strategies to reduce antibiotic use in LMICs.

In this study we explore the perception of physicians and pharmacists towards AMR, antibiotic use, and the feasibility of implementing effective stewardship in a tertiary care hospital in Ethiopia.

Similar to other published studies conducted both in high and low income countries, participants generally agreed AMR was a global threat but tended to think it was a bigger problem elsewhere [14–18]. All key informants and most surveyed respondents clearly understood that overuse of antibiotics (exacerbated by illegal over the counter dispensing of antibiotics) were significant drivers of AMR [19–22]; however, unlike several studies conducted in low-resource settings, they did not perceive patient demand as having a significant impact on their antibiotic prescriptions [23, 24].

Most respondents felt that antimicrobial stewardship is a key strategy to limit AMR and favored an approach based on education, access to local AMR surveillance data, and prospective non-confrontational feedback, similar to surveys conducted elsewhere [25, 26] and to approaches recommended by international societies [27, 28]. Interestingly, fear of losing prescribing autonomy was not a recurrent theme among our respondents.

A striking finding from our study was the erroneous perceptions from physicians’ that drug-resistant gram-positive infections (especially MRSA) are of greater concern than drug-resistant gram-negative infections, despite knowledge of ongoing outbreaks and several reports on highly drug-resistant gram-negative infections but relatively few cases of MRSA in the same institution [29,30]. This common misperception, probably fueled by familiarity with literature on MRSA infections from other countries, is likely directly linked to the unnecessary use of vancomycin, which alone accounted for close to one third of the antibiotics budget and 6% of the total pharmacy budget of the hospital. Reducing vancomycin prescriptions therefore represents an obvious “low-hanging fruit” for stewardship as well as for cost-control efforts. From a larger perspective, it highlights not only the significant gap between knowledge and practice, but also suggests that guidelines based on literature emanating from high-income countries adversely impact prescribing behaviors in LMIC and can undermine stewardship efforts. For Ethiopia and other LMICs, significant scale-up of diagnostic infrastructure and up-to-date local surveillance need to be prioritized for any antimicrobial stewardship efforts to be successful [31, 32].

Physicians readily acknowledged they routinely failed to send specimens for culture and/or to follow up and act on results. While most agreed with general statements on the importance of diagnostics in stewardship, when interviewed about specific factors that would help them optimize their antibiotic prescriptions, they focused on the need for institutional *empiric* treatment guidelines and omitted to comment on improving diagnostic capacity. This attitude is reflective of the deeply embedded, widely endorsed, syndromic approach to managing suspected infections in LMICs, and represents in our opinion the greatest barrier to implementing effective stewardship in those settings. Other surveys conducted in Africa have suggested similar tendencies: only a minority of physicians use microbiology tests to guide their prescriptions, even when these are available [33]. Implementing systematic microbiological testing and focusing educational efforts on appropriate use of diagnostic tests for infections constitutes a major paradigm shift for LMICs, but certainly deserves to be a core objective in stewardship efforts in these settings. Recent developments such as WHO’s Essential Diagnostics List (to complement the long-standing Essential Medicines list) [34], and a growing literature on building bacteriology capacity in LRS are encouraging steps in that direction [32, 35, 36].

Another major finding from our study was that most respondents admitted their antibiotic prescription behaviors are driven more by perceived risk of treatment failure and fear of retribution or academic punishment (especially for junior physicians), rather than evidence for infection. While this has not, to our knowledge, been previously reported for antimicrobial prescriptions, it is not surprising junior physicians operating in hierarchical academic centers might fear a judgment of incompetence from seniors, and would therefore be influenced by their *perceptions* of seniors’ expectations–suggesting that training and educational efforts should perhaps initially target senior physicians in academic LMIC settings.

Finally, pharmacists were more likely to identify inappropriate combinations, doses or other pharmacokinetic/pharmacodynamic parameters than physicians, but tended not to communicate their observations to the prescribing physician for the simple reason that these professional groups hardly ever interact. Including clinical pharmacists within the treating teams and facilitating their participation in clinical rounds for example, could lead to significant improvements in antibiotic prescriptions.

Our study has some limitations. Since our primary goal was to gauge feasibility and acceptability of a stewardship intervention in a large African tertiary care center with moderate infrastructure, we did not perform in-depth knowledge assessments and therefore only draw limited conclusions on specific inappropriate antibiotic uses from our survey. We did not include nurses or administrators since these professionals are not involved in prescribing or recommending antibiotics; surveying a broader range of professionals might have generated insights on institutional policies and logistical challenges, but would have added significant complexity to the analysis. Finally, as the study was conducted in a single academic tertiary care center (the largest in Ethiopia), some of the lessons learned might not be generalizable to smaller regional or district level hospitals, or to private hospitals where availability of antibiotics and diagnostic tests might be different. Nonetheless, we provide a detailed quantitative and qualitative exploration of the perceptions and beliefs of the two largest groups of professionals who prescribe/dispense antibiotics, and demonstrate that performing a locally adapted survey of beliefs and perceptions prior to implementing a stewardship intervention can be useful to identify systemic obstacles and specific stewardship intervention targets for LMICs.

## Supporting information

S1 Table Quantitative survey questionnaire. (DOC)

S2 Table Qualitative survey questionnaire. (DOCX)

S1 Document Amharic Version of Questionnaire. (PDF)

S2 Document Interview transcripts in English. (DOCX)
