# Supplementary material for: Opportunities and barriers to implementing antibiotic stewardship in low and middle-income countries: Lessons from a mixed-methods study in a tertiary care hospital in Ethiopia
Source: PLoS One. 2018 Dec 20;13(12):e0208447. doi: 10.1371/journal.pone.0208447 (PMC6301706; doi:10.1371/journal.pone.0208447)
Supplement: S1 Table — (DOC) [file pone.0208447.s001.doc]

| **HEALTHCARE PROFESSIONAL’S PRE-INTERVENTION PERCEPTION SURVEY** |
| --- |
| **Introduction: This survey asks for your perceptions about antimicrobial resistance (AMR) and antimicrobial stewardship programs (ASP). The findings of this survey will be used as input for developing an effective ASP in the hospital. It will take you about 10 to 15 minutes to complete. Your honest response is essential. If you do not wish to answer a question, or if a question does not apply to you, you may answer n/a.** |

| 1. **ANTIMICROBIAL RESISTANCE: SCOPE OF THE PROBLEM AND KEY CONTRIBUTORS** | | | | | | | | | | | |
| --- | --- | --- | --- | --- | --- | --- | --- | --- | --- | --- | --- |
| Please indicate your agreement or disagreement with the following statements by encircling ONE number on the provided scales: **1= Strongly Disagree; 2= disagree; 3=Neutral; 4= Agree; 5= Strongly Agree**. If you don’t feel that you have the knowledge to rate the item, please tick mark (**√**) on the **“n/a"** column. | | | | | | | | | | | |
| **Items** | | | **Your Level of Agreement** | | | | | | | | |
|  | Antimicrobial resistance is a significant problem worldwide | | 1 | 2 | 3 | 4 | | 5 | | n/a | |
|  | Antimicrobial resistance is a significant problem in my country | | 1 | 2 | 3 | 4 | | 5 | |  | |
|  | Antimicrobial resistance is a significant problem in my hospital | | 1 | 2 | 3 | 4 | | 5 | |  | |
|  | Antimicrobial resistance is a problem in my daily practice | | 1 | 2 | 3 | 4 | | 5 | |  | |
|  | Inappropriate use of antibiotics is a major cause of antimicrobial resistance in this hospital | | 1 | 2 | 3 | 4 | | 5 | |  | |
|  | The easy access to antibiotics without a prescription in Ethiopia contributes to antimicrobial resistance | | 1 | 2 | 3 | 4 | | 5 | |  | |
|  | The prescription of broad-spectrum antibiotics is directly linked to antimicrobial resistance in this hospital | | 1 | 2 | 3 | 4 | | 5 | |  | |
|  | This hospital performs adequate surveillance for drug resistant organisms | | 1 | 2 | 3 | 4 | | 5 | |  | |
|  | The lack of adequate diagnostic tests in this hospital leads to overuse of antibiotics | | 1 | 2 | 3 | 4 | | 5 | |  | |
|  | This hospital provides adequate staff education regarding antibiotic use and resistance | | 1 | 2 | 3 | 4 | | 5 | |  | |
|  | A patient is likely to develop drug-resistant infection during their hospital stay at this hospital | | 1 | 2 | 3 | 4 | | 5 | |  | |
|  | I suspect that antibiotics available in my hospital are of poor quality and might not be effective | | 1 | 2 | 3 | 4 | | 5 | |  | |
|  | My choice of antibiotics is often influenced by the availability of the antibiotics rather than by the local antibiogram or by the etiologic cause of disease (availability of laboratory results) | | 1 | 2 | 3 | 4 | | 5 | |  | |
|  | The sporadic supply of antibiotics in my hospital leads to interruptions of therapy thereby contributing to antimicrobial resistance | | 1 | 2 | 3 | 4 | | 5 | |  | |
|  | The lack of close clinical follow-up during antibiotic use in my hospital contributes to antimicrobial resistance | | 1 | 2 | 3 | 4 | | 5 | |  | |
|  | Patient demands and expectations contribute to overuse of antibiotics in this hospital | | 1 | 2 | 3 | 4 | | 5 | |  | |
|  | Cost considerations for the patient affects my choice of antibiotics | | 1 | 2 | 3 | 4 | | 5 | |  | |
|  | Poor infection control practices by health professionals significantly contributes to the spread of antimicrobial resistance in this hospital | | 1 | 2 | 3 | 4 | | 5 | |  | |
|  | Adherence to hand-hygiene protocols is acceptable at this hospital | | 1 | 2 | 3 | 4 | | 5 | |  | |
|  | Patient rooms and equipment are cleaned appropriately as per hospital cleaning protocol once a patient carrying a drug resistant organism (DRO) has been discharged from this hospital | | 1 | 2 | 3 | 4 | | 5 | |  | |
|  | I think a very high proportion (>30%) of gram negative infections are highly drug- resistant in this hospital (resistant to all cephalosporins, and some are even resistant to carbapenems) | | 1 | 2 | 3 | 4 | | 5 | |  | |
|  | I think a very high proportion (>30%) of Staphylococcal infections are resistant to methicillin (MRSA) in this hospital | | 1 | 2 | 3 | 4 | | 5 | |  | |
| 1. **ANTIBIOTIC PRESCRIBING/DISPENSING PRACTICES** | | | | | | | | | | | |
|  | | Microbiology lab results are timely communicated to the health professionals in this hospital | 1 | 2 | 3 | 4 | | 5 | | n/a | |
|  | | I regularly refer to/consider the antibiotic susceptibility pat­terns at this hospital (institutional antibiogram) when empirically prescribing or recommending antibiotics | 1 | 2 | 3 | 4 | | 5 | |  | |
|  | | If medically appropriate, I routinely try to step down intravenous antibiotics to an oral alternative antibiotic after about three days | 1 | 2 | 3 | 4 | | 5 | |  | |
|  | | If medically appropriate, I routinely try to step down broad-spectrum antibiotics to a narrow-spectrum antibiotic after about three days | 1 | 2 | 3 | 4 | | 5 | |  | |
|  | | Restrictions on antibiotics could impair my ability to provide good patient care | 1 | 2 | 3 | 4 | | 5 | |  | |
|  | | I routinely prescribe/recommend very broad-spectrum antibiotics empirically because I believe most patients are infected with a drug-resistant organism | 1 | 2 | 3 | 4 | | 5 | |  | |
|  | | I routinely prescribe/recommend very broad-spectrum antibiotics empirically because microbiology lab results are not available in a timely fashion | 1 | 2 | 3 | 4 | | 5 | |  | |
|  | | I routinely check microbiology laboratory results to guide my choice of antibiotics | 1 | 2 | 3 | 4 | | 5 | |  | |
|  | | In the past seven days, I have prescribed broad spectrum antibiotics for longer than 3 days for fewer than 10% of my patients; | 1 | 2 | 3 | 4 | | 5 | |  | |
|  | | In the past seven days, I have prescribed broad spectrum antibiotics for longer than 3 days for 10-50% of my patients | 1 | 2 | 3 | 4 | | 5 | |  | |
|  | | In the past seven days, I have prescribed broad spectrum antibiotics for longer than 3 days for more than 50% of my patients | 1 | 2 | 3 | 4 | | 5 | |  | |
| 1. **ANTIMICROBIAL STEWARDSHIP PROGRAMS (ASP)**   **(A formal multidisciplinary program that monitors and manages the appropriate use of antibiotics)** | | | | | | | | | | | |
|  | | Antimicrobial stewardship programs (ASP) improve quality of patient care | 1 | 2 | 3 | | 4 | | 5 | | n/a |
|  | | ASP reduces antibiotic use overall and can result in cost savings | 1 | 2 | 3 | | 4 | | 5 | |  |
|  | | ASP reduces duration of hospital stay and associated costs | 1 | 2 | 3 | | 4 | | 5 | |  |
|  | | ASP reduce the problem of antimicrobial resistance | 1 | 2 | 3 | | 4 | | 5 | |  |
|  | | ASP impact an institution’s nosocomial infection rates | 1 | 2 | 3 | | 4 | | 5 | |  |
|  | | This hospital has the capacity to establish and implement an effective antimicrobial stewardship program | 1 | 2 | 3 | | 4 | | 5 | |  |
|  | | My individual efforts in antimicrobial stewardship can significantly impact this hospital’s antimicrobial resistance problem | 1 | 2 | 3 | | 4 | | 5 | |  |
|  | | I would like more feedback on my antibiotic selections | 1 | 2 | 3 | | 4 | | 5 | |  |
|  | | Antimicrobial stewardship programs can be an obstacle to good patient care | 1 | 2 | 3 | | 4 | | 5 | |  |
|  | | Antimicrobial stewardship programs override prescribers’ decision autonomy | 1 | 2 | 3 | | 4 | | 5 | |  |
|  | | I do not have enough time to further invest into antimicrobial stewardship program | 1 | 2 | 3 | | 4 | | 5 | |  |
|  | | Infectious diseases experts that can provide guidance in antibiotic selection and prescription are available in this hospital | 1 | 2 | 3 | | 4 | | 5 | |  |
|  | | Additional staff education on antibiotic prescribing and use is needed in this hospital | 1 | 2 | 3 | | 4 | | 5 | |  |
|  | | Prescribing physicians are the only professionals who need to understand antimicrobial stewardship | 1 | 2 | 3 | | 4 | | 5 | |  |
|  | | Pharmacists with sufficient training to provide guidance on antibiotics (ex. *Antibiotic switches*, *IV to PO step-down,*renal dose adjustments) are available in this hospital | 1 | 2 | 3 | | 4 | | 5 | |  |
|  | | Implementation of electronic medical recording (e.g. receiving results electronically) can improve effect of antimicrobial stewardship program | 1 | 2 | 3 | | 4 | | 5 | |  |

1. **Beliefs on Suggested Potential Solutions for Preventing Antimicrobial Resistance**

| Please indicate your perception of the potential of suggested solution to reduce AMR by encircling ONLY ONE number on the provided scale: **1= Probably or definitively ineffective (Not useful); 2= Unsure; 3= Probably or definitively effective** | | | | |
| --- | --- | --- | --- | --- |
| **Suggested Solutions to Reduce Antimicrobial Resistance** | | **Agreement** | | |
|  | Education on antimicrobial therapy to medical and pharmacy staff | 1 | 2 | 3 |
|  | Develop new institutional guidelines for empiric antimicrobial use | 1 | 2 | 3 |
|  | Access to institution-specific antibiogram to treating teams | 1 | 2 | 3 |
|  | Implementation of prospective audit and feedback (multidisciplinary rounds on appropriate prescribing and use of antibiotics) | 1 | 2 | 3 |
|  | Active involvement of hospital infection prevention and control team | 1 | 2 | 3 |
|  | Antibiotic cycling intervention (e.g. scheduled rotation of 3rd or 4th generation cephalosporins with carbapenems and piperacillin-tazobactam for pre-determined time periods) | 1 | 2 | 3 |
|  | Antibiotic restriction intervention (certain antibiotics cannot be prescribed without infectious disease specialist approval for restricted antibiotics) | 1 | 2 | 3 |

1. **Background Information of Respondents**

| **Socio-demographic Questions** | | **Response** |
| --- | --- | --- |
|  | Age in years­­ | __________ years |
|  | Gender | 🞏 Male 🞏  Female |
|  | What is your primary work area or unit in this hospital? (Please check ONE answer) | 🞏 Medicine (non-surgical) 🞏 Surgery 🞏 Pediatrics  🞏 Gynecology/Obstetrics 🞏 Rotation (among wards) 🞏 Emergency (ED) 🞏 Pharmacy  🞏 Other (please specify): ________________ |
|  | What is your staff position in this hospital? | 🞏 Consultant physician or attending staff  🞏 Fellow (postgraduate) physician 🞏 Medical student  🞏 Resident physician/Intern 🞏 Pharmacist  🞏 Other (please specify): ________ |
|  | How long have you worked in this hospital? | ______________years; _________months |
|  | How long have you worked in your current specialty or profession? | ______________years; _________months |
|  | How many patients do you treat on average, per week (provide your best estimate) | __________ patients/week |
|  | How many of your patients do get antibiotic prescription, per week (provide your best estimate) | __________ patients/week |

1. Is there anything you would like share with us? Please write it in the space provided. ________________________________________________________________________________________________________________________________________________________________________________________________________________________________________________

**Thank you for your time!!!**
