# Supplementary material for: Opportunities and barriers to implementing antibiotic stewardship in low and middle-income countries: Lessons from a mixed-methods study in a tertiary care hospital in Ethiopia
Source: PLoS One. 2018 Dec 20;13(12):e0208447. doi: 10.1371/journal.pone.0208447 (PMC6301706; doi:10.1371/journal.pone.0208447)
Supplement: S2 Table — (DOCX) [file pone.0208447.s002.docx]

**Supplementary file 2: Interview Guides for Key Informant Interviews (Physicians and Pharmacists)**

1. **Demographic Information of Respondents**

| **Socio-demographic Questions** | | **Response** |
| --- | --- | --- |
|  | Age in years­­ | __________ years |
|  | Gender | 🞏 Male 🞏 Female |
|  | What is your primary work area or unit in this institution? (Please check ONE answer) | 🞏 Medicine (non-surgical)  🞏 Surgery  🞏 Pediatrics  🞏 Gynecology/Obstetrics  🞏 Rotation (among wards)  🞏 Emergency  🞏 Pharmacy  🞏 Laboratory  🞏 Other (please specify): __________ |
|  | What is your staff position in this institution? | 🞏 Consultant physician or attending staff  🞏 Fellow (postgraduate) physician  🞏 Resident physician/Intern  🞏 Pharmacist  🞏 Other (please specify): ________ |
|  | How long have you worked in this institution? | ______________years; _________months |
|  | How long have you worked in your current specialty or profession? | ______________years; _________months |
|  | How many patients do you treat on average, per week (provide your best estimate) | __________ patients/week |

1. **Interview Guide**
2. **Antibiotic Use**
   1. How do you describe the current use of antibiotics in Ethiopia and particularly in this institution?
   2. Do you think inappropriate use of antibiotics is a problem in Ethiopia and in this institution? Which problems are more prevalent or common in this setting? Why? How do you describe the pattern of this problem over time (Is it increasing, decreasing or stable)? Why?
   3. How do you compare the use of broad vs. narrow spectrum antibiotics? Do prescribers tend to prescribe very broad-spectrum antibiotics empirically and/or to over prescribe antibiotics? What are the potential reasons for the preference of broad spectrum antibiotics? What factors do contribute for the overuse of antibiotics?
   4. How do you describe patients’ pressure to prescribe antibiotics?
      - In your experience, do patients perceive of antibiotic overuse as a problem?
      - Do you ever feel pressure from patients to prescribe antibiotics? If yes, how?
      - Do patients ever express concern about themselves/ their child being prescribed antibiotics? Do they ever pressure you into not prescribing antibiotics?
3. **Antimicrobial Resistance and Contributing Factors**
   1. How do you describe the current status of antimicrobial resistance (at institutional and national level)? Should healthcare professionals be worried of this emerging problem? How serious is the problem? Do you think it is a growing public health treat or not?
   2. How frequently do you encounter drug resistance organisms within your clinical practice?
      - Which types of pathogens/infections do you think have more resistance profile?
      - Do you face any challenge in your daily practice as a result of antimicrobial resistance? How does it affect you? How does it affect the patient?
   3. What factors do you think are contributing to increasing emergence and spread of antimicrobial resistance? Please explain on how commonly each of them exist in your hospital?
   4. How do you describe the utilization of laboratory findings in the diagnosis of infectious etiologies?
4. **Antimicrobial Containment Strategies**
   1. Do you have concern in the way antibiotics are prescribed and used today? Do you think you can impact the problems of antibiotic use? If yes, in what ways?
   2. What possible measures do you recommend for better antimicrobial resistance containment practices to be implemented in this facility? Please relate your recommendations based on its importance.
   3. From your experience, can you think of potential factors that might influence the antimicrobial resistance containment practice in the hospital?
   4. Do you feel regular auditing have an impact on prescribers’ behavior? How?
   5. How would you feel if prospective audit and feedback is applied in this institution and a multidisciplinary team review and give you feedbacks on your prescribing?
   6. Can you help us understand the potential factors that might influence the implementation of prospective audit and feedback in this institution? How do these factors become barriers?
5. That is the end of my interview, is there anything you would like to add?

**________________________________________________________________________**

**________________________________________________________________________________________________________________________________________________**

**Thank you for your time!!!**
