# Supplementary material for: Opportunities and barriers to implementing antibiotic stewardship in low and middle-income countries: Lessons from a mixed-methods study in a tertiary care hospital in Ethiopia
Source: PLoS One. 2018 Dec 20;13(12):e0208447. doi: 10.1371/journal.pone.0208447 (PMC6301706; doi:10.1371/journal.pone.0208447)
Supplement: S1 Document — (PDF) [file pone.0208447.s003.pdf]

Amharic Version of the Key Informants Interview Guide for Physicians and Pharmacists

ከሀኪም እና ፋርማሲስት ጋር ለሚደረግ ቃለ ምልልስ የተዘጋጀ መጠይቅ

ሀ. የተጠያቂው መሰረታዊ መረጃዎች

ጥያቄ		ምላሽ
1.	ዕድሜ	___________ ዓመት
2.	ፆታ	☐ ወንድ ☐ ሴት
3.	አሁን የሚገኙበት የመጀመሪያ የስራ ክፍል ወይም ቦታ የትኛው ነው? (እባክዎትን አንድ መልስ ብቻ ይምረጡ)	☐ የውስጥ ደዌ (ከቀዶ ጥገና ውጪ) ☐ ቀዶ ጥገና ☐ የህፃናት<br>☐ የማህፀን/ የማዋለጃ ☐ በዝውውር (በዋርዶች መካከል)<br>☐ ድንገተኛ ☐ ፋርማሲ<br>☐ ሌሎች (እባክዎን ይግለፁት): _________________
4.	እዚህ ሆስፒታል ውስጥ ያለዎት የስራ ወይም የሙያ ድርሻ ምንድን ነው?	☐ አማካሪ ሀኪም ወይም የሆስፒታሉ ሰራተኛ<br>☐ ሳብስፔሻሊቲ ተማሪና ሀኪም ☐ የህክምና ተማሪ<br>☐ ድህረ ምረቃ (ስፔሻሊቲ) ተማሪና ሀኪም/የመጨረሻ ዓመት የህክምና ተማሪ<br>☐ ፋርማሲስት<br>☐ ሌሎች (እባክዎን ይግለፁት):_______________
5.	በሆስፒታሉ ውስጥ ለምን ያህል ጊዜ ሰርተዋል;?	_______________ዓመት; __________ወራቶች
6.	አሁን በሚሰሩበት የሙያ ደረጃ ወይም ስፔሻሊቲ ለምን ያህል ጊዜ ሰርተዋል?	_______________ዓመት; __________ወራቶች
7.	በአማካኝ በሳምንት ምን ያህል ህመምተኞችን ያክማሉ (በጣም ጥሩ ግምት የሚሉትን ያስቀምጡ)	___________ ታካሚዎች/ሳምንት
8.	በሳምንት ውስጥ ምን ያህል ታካሚ የፀረ ባክቴሪያ መድሃኒት የታዘዝለታል (በጣም ጥሩ ግምት የሚሉትን ያስቀምጡ))	___________ ታካሚዎች/ሳምንት

ለ. ስለ የፀረ - ባክቴሪያ መድሃኒት አጠቃቀምና የመላመድ ሁኔታ ለማወቅ የተዘጋጁ ጥያቄዎች

1. **የፀረ-ባክቴሪያ አጠቃቀም**

1.1. አሁን በኢትዮጵያ ውስጥ ያለውን፣በተለይ አሁን በሚገኙበት የስራ ተቋም ውስጥ የሚታየውን የፀረ ባክቴሪያ አጠቃቀም እንዴት ይገልፁታል?

1.2. በኢትዮጵያ ወይም በዚህ የስራ ተቋም ውስጥ የፀረ-ባክቴሪያ ያለአግባብ አጠቃቀም ችግር ነው ብለው ያስባሉ? በዚህ ተቋም ውስጥ የትኛዎቹ ችግሮች በብዛት ወይም በዋናነት ይጠቀሳሉ? ለምን? አሁን የሚገኘውን ችግር ከጊዜ ሂደት ጋር እንዴት ይገልፁታል (ጨምሯል፣ ቀንሷል፣ ወይስ ባለበት ሁኔታ ነው?) ለምን?

1.3. ለሰፊ እና ለተወሰኑ ባክቴሪያዎች የሚታዘዙ የፀረ ባክቴሪያ መድሃኒቶች አጠቃቀም እንዴት ያወዳድሯቸዋል? ሀኪሙ ለሰፊ ባክቴሪያዎች የሚሆኑትን መድሃኒቶች በሚያዝበት ወቅት ወደ ተግባሩን ወደተከተለ ወይስ/እና ወደ መጠን በላይ ትዕዛዝ ያደላል? ሀኪሙ ለሰፊ ባክቴሪያዎች የሚታዙትን መድሃኒቶች የሚመርጥበት ሁነኛ ምክንያቶች ምንድን ናቸው? የፀረ-ባክቴሪያ ከመጠን በላይ ለመጠቀም አበረታች የሆኑ ችግሮች ምንድን ናቸው?

1.4. ታካሚው የፀረ-ባክቴሪያ መድሃኒት እንዲታዘዝለት ያለው ግፊት ወይም ውትወታ እንዴት ይገልፁታል?

- ባልዎት የስራ ልምድ፣ ታካሚው ከመጠን በላይ የሆነ የፀረ-ባክቴሪያ መድሃኒት መጠቀም ችግር ነው ብሎ ነው የሚገነዘቡት?

- ከታካሚው በኩል የፀረ-ባክቴሪያ መድሃኒት እንዲያዙለት ግፊት ወይም ውትወታ አጋጥሞዎት ያውቃል? እንዴት ነበር?
- ከታካሚው በኩል በአፍ ከሚወሰዱ ይልቅ በመርፌ የሚወሰዱትን የፀረ-ባክቴሪያ መድሃኒት እንዲያዙለት ግፊት ወይም ውትወታ አጋጥሞዎት ያውቃል? ምክንያቱ ምን ይመስልዎታል?
- ታካሚው ለራሱም ሆነ ለልጁ በሚታዘዙት የፀረ-ባክቴሪያ መድሃኒቶች ላይ ያለውን ቅራኔ ገልፆ ያውቃል? እነዚህን መድሃኒቶች እንዳያዙስ ከታካሚው በኩል ግፊቶች ኖረው ያውቃሉ?

2. **የፀረ-ተህዋስያን መድሃኒቶች የመላመድ ሁኔታና አባባሽ ሁኔታዎች**

2.1. አሁን ያለውን የፀረ-ተህዋስያን መድሃኒቶች የመላመድ ሁኔታ እንዴት ይገልፁታል (ባሉበት የስራ ተቋም እና በአገር ደረጃ)? አሁን እየተስፋፋ ያለውን ችግር የጤና ባለሙያውን ሊያሳስበው ይገባል? ችግሩ ምን ያህል አደገኛ ነው? እያደገ ላለው የጤና ማህበረሰብስ አዳጋች ነው ብለው ያስባሉ?

2.2. ይህ ችግር (የመድሃኒት የመላመድ ችግር) አሁን ባሉበት የህክምና ስራ ውስጥ በምን ያህል ጊዜ ድግግሞሽ ይከሰታል?

- የትኞቹ አይነት ፀረ-ተህዋሲያን ወይም ኢንፌክሽኖች ናቸው የበለጠ የመድሃኒት የመላመድ ገፅታ ያላቸው?
- አሁን ባሉበት የቀን ለቀን የስራ ሁኔታ ውስጥ በፀረ-ተህዋስያን የመላመድ ችግር የተነሳ ያጋጠምዎት አዳጋች ነገር አለ? እርስዎ ላይ እንዴት ተፅዕኖ አሳረፈ? ታካሚው ላይስ የሚያሳርፈው ተፅዕኖ እንዴት ነው?

2.3. አሁን ያለውን የፀረ-ተህዋስያን የመድሃኒት የመላመድን ችግር እንዲበዛ ወይም እንዲስፋፋ አባባሽ ሁኔታዎች ምንድን ናቸው ብለው ያስባሉ? እባክዎትን ቀጥለው የሚገኙትን እያንዳንዱ ችግሮች ወይም ሁኔታዎች እንዴት በዋናነት እንደሚታዩ ይግለፁ?

2.4. ኢንፌክሽኖችን ለይቶና መርምሮ ለማውጣት ባለው ሂደት ውስጥ የላብራቶሪ ውጤቶች አጠቃቀም እንዴት ይገልፁታል?

3. **የፀረ-ተህዋስያን መድሃኒቶች አያያዝ እና አጠቃቀም እቅዶች**

3.1. በአሁኑ ወቅት ከፀረ-ባክቴሪያ መድሃኒቶች የህኪም ትዕዛዝ እና አጠቃቀም ጋር ተያይዞ የሚያሳስቦት አለ? ባለው የአጠቃቀም ችግር ላይ ለውጥ ተፅዕኖ ይኖረኛል ብለው ያስባሉ? አዎ ካሉ ፤ በምን አይነት መንገዶች?

3.2. የተሻለ የፀረ-ባክቴሪያ መድሃኒቶች የህኪም ትዕዛዝ እና አጠቃቀም እንዲኖር አሁን ባሉበት ተቋም ውስጥ ምን አይነት እርምጃዎች ቢወሰዱ ብለው ያሳስባሉ? እባክዎን ያልዎትን ማሳሰቢያ ካለው ጥቅም ጋር አያይዘው ይግለፁልን?

3.3. ካለዎት የስራ ልምድ አኳያ፣ አሁን ባሉበት ተቋም ውስጥ የፀረ-ባክቴሪያ መድሃኒቶች አስተዛዘዝ እና አጠቃቀም ላይ ሁነኛ ተፅዕኖ ያላቸው ችግሮች ምንድን ናቸው?

- ተጨማሪ ጥያቄ: የፀረ-ባክቴሪያ መድሃኒቶች አስተዛዘዝ እና አጠቃቀም ላይ ገዳቢ እና አበረታች ሁኔታዎች ምንድን ናቸው?

3.4. ቀጣይነት ያለው ቁጥጥር እና ምላሽን ተገን ያደረጉ ለውጦችን መተግበር

- ጊዜውን የጠበቀ ቁጥጥር በሀኪሙ መድሃኒቱን የማዘዝ ያለው ባህሪይ ላይ ለውጥ ይኖረዋል ብለው ይሰባሉ? እንዴት?
- አሁን ባሉበት የስራ ተቋም ከመድሃኒት የማዘዝ ባህሪዮ ጋር ተያይዞ ቀጣይነት ያለው ቁጥጥር እና ምላሽን ተገን ያደረጉ ለውጦችን ቢተገበሩ፣ ከተለያየ ሙያ የተቀነባበረ ቡድን ቢመለከተውና ተገቢ ምላሽ ቢሰጠው ምን ይሰማዎታል?
- ቀጣይነት ያለው ቁጥጥር እና ምላሽን ተገን ያደረጉ ለውጦችን መተግበር እንዳያስችል ሊያደርጉ የሚችሉ ተፅዕኖችን እንድንገነዘብ ሊረዱን ይችላሉ? እንዴት ነው እነዚህ ተፅዕኖዎች ገዳቢ ሊሆኑ የሚችሉት?

4. ቃለ መጠይቁ አልቋል፣ መጨመር የሚፈልጉት ማንኛውም ነጥብ ሊኖር ይችላል?__________________________

ለትብብርዎ አመሰግናለሁ!!!
