# Supplementary material for: Opportunities and barriers to implementing antibiotic stewardship in low and middle-income countries: Lessons from a mixed-methods study in a tertiary care hospital in Ethiopia
Source: PLoS One. 2018 Dec 20;13(12):e0208447. doi: 10.1371/journal.pone.0208447 (PMC6301706; doi:10.1371/journal.pone.0208447)
Supplement: S2 Document — (DOCX) [file pone.0208447.s004.docx]

# Qualitative survey

# TRANSCRIPTS OF INTERVIEWS

Transcribed by Gebre-Medhin Beedmariam

Transcripts reviewed by Workeabeba Abebe and Makeda Semret

Table of Contents

Qualitative survey 1

TRANSCRIPTS OF INTERVIEWS 1

Pharmacist KI#01 4

Pharmacist KI#02 9

Pharmacist KI#03 16

Pharmacist KI#04 23

Pharmacist KI #5 30

Pharmacist KI#06 32

Pharmacist KI#09 47

Pharmacist KI#10 53

Pharmacist KI#11 57

Pharmacist KI#12 63

Pharmacist KI#13 69

Pharmacist KI#14 74

Physician KI#01 80

Physician KI#02 88

Physician KI#03 94

Physician KI#04 99

Physician KI#05 104

Physician KI#06 108

Physician KI#07 111

Physician KI#08 115

Physician KI#09 119

Physician KI#09 131

Physician KI#10 142

Physician KI#11 151

Physician KI#12 157

Physician KI#13 162

Physician KI#14 168

Physician KI#15 173

Physician KI#16 179

Physician KI#17 185

Physician KI#18 190

Physician KI#19 195

Physician KI#20 203

## Pharmacist KI#01

Age: 26 years

Sex: Male

Department: Pharmacist [Oncology, Clinical Pharmacy]

Staff Position: Lecturer and preceptor of clinical pharmacist

Total work experience: 4 years

Number of patients treated per day: N/A

Number of patients with ABX prescription per day: N/A

Duration of Interview (Minutes): 55:28 Minutes

**Interviewer:** Ok thank you, to start with my question, how do you describe the current use of antibiotics in this institution or even at country level?

**Respondent:** I have no study to say about the national prevalence but from my experience, antibiotic use here in TASH has a fashionable/trendy approach prescription which is based on recommendation not or guidelines or evidence based prescribing. Prescribing doesn’t base on microbiological results but on their own personal experience and if one senior prescribe one antibiotic the junior continue to use it for all patients without questioning. ***It is very poor, over-prescribing or overuse is very common. I think they being used like antipain medication.***

**Interviewer:** So, Do you think inappropriate use of antibiotics is a problem? How do you describe the pattern of this problem over time (Is it increasing, decreasing or stable)? Why it is a problem?

**Respondent:** Both overuse and broad spectrum use of antibiotics are major problems. Yes there is an antibiotic overuse problem ***that’s increasing in pattern over time***. Since TASH is a territory hospital the ***perception of physicians is that patients have already been exposed to medications prior to this visit*** to Black Lion hence they tend to go with the option of empirical treatment for all patients. There is also a ***problem of availability*** which is why the ***prescribers are often influenced by supply rather than demand while prescribing and tend to overuse certain antibiotics only.*** In general since prescribing is not following guideline there is problem of use and even at times while patients are showing prognosis there is ***no tendency switch IV to PO probably*** they don’t want to take the possible risks, if it happen. ***Again there is no study that indicate the most common pathogens, resistance profile of these pathogens which means absence of antibiogram is the major reason.***

**Interviewer:** How do you compare the use of broad vs. narrow spectrum antibiotics? Why do you think so?

**Respondent: *Broad spectrum um medications are prescribed more often because doctors mostly treat empirically.*** This is because ***culture and sensitivity testing is costly*** and it is ***time taking to*** obtain results while the patient is ***severely infected and cannot afford to lose lots of time*** but doctors are more ***influenced by simple reluctance***  rather than this concern for the patient to go with empirical treatment.

**Interviewer:** How do you describe patients’ pressure to use of antibiotics or not to use antibiotics? In your experience, do patients perceive of antibiotic overuse as a problem?

**Respondent: *They don’t show concern for overuse.*** Most patients in TASH are of ***low economic status and cannot afford*** to buy their medications unless they are available to them for free in TASH pharmacies so doctors will be forced to prescribe whatever is available to them even though it isn’t the best option**. *So except in the case of cost issues patients do not pose resistance to accept prescriptions or exert pressure to get antibiotics.*** You can also consider our literacy level, which is low and you can imagine that patient usually accept whatever they are given.

**Interviewer:** How do you see patients preference of IV over PO antibiotics? Why do you think so?

**Respondent:** No, I haven’t had such an experience so far.

**Interviewer:** How do you describe the current status of antimicrobial resistance? How serious is the problem? Do you think it is a growing public health treat or not?

**Respondent:** It is a very big problem and a public health threat too. It beyond words of saying it is big challenge. Currently most patients are not responding to common antibiotics indicating that resistance is a challenge; then they empirically switched it again but again it doesn’t work. So, when you prescribed the available antibiotics we see the response is very low. In general resistance pattern is so frightening, for instance we have seen a preliminary report of ceftriaxone resistance profile to common pathogens and resistance was found to be >86%. This is for a single drug but it could be the same for others. ***And the major problem is in our treatment is trial and error but even with this problem culture and sensitivity is not done.*** Even new molecules Meropenem and pep/tazo are becoming resistant. The problem of resistance is also increasing over time. For instance a patient 3-4 years back ceftriaxone was effective but now it is response it not satisfactory. Meropenem and imipenem were very effective but we are now observing failure for this drugs; same is true for pep/tazo. These drugs are broad spectrum but even these drugs are not responding which indicates the increase in resistance.

**Interviewer**: How frequently do you encounter drug resistance organisms within your clinical practice? Which types of pathogens/infections do you think have more resistance profile?

**Respondent: *There is a very high frequency of encountering drug resistant organisms.*** For example in a study conducted in TASH, it was found that >80% of the disease causing pathogens were resistant to ceftriaxone and am sure many more of our currently used antibiotics also face this resistance by the infective pathogens. Even though it would be difficult to list down pathogens with resistance, the most feared in my area of practice would be ***Pseudomonas aeruginosa, staph aureus and E.coli.***

**Interviewer**: In which samples do you see a lot more reports of antibiotic resistance; is it urine, bllod or what?

**Respondent:** Some are obtained from urine samples in my experience (I wouldn’t be able to say they are the most common samples source for resistant pathogens though).

Interviewer: Do you face any challenge in your daily practice as a result of antimicrobial resistance? How does it affect you and the patient?

**Respondent:** The issue of antimicrobial resistance definitely challenges the practice of both the pharmacist and the physician. ***The physician faces the stress of having to select out of very few drugs available whilst the pharmacist dedicates much of his time in an attempt to provide medications that aren’t easily availed in the hospital pharmacy rather than being able to focus on other pharmaceutical care services he/she should be providing.*** So both professionals frustrate over not being able to achieve therapeutic success for a patient.

**Interviewer**: What factors do you think are contributing to increasing emergence and spread of antimicrobial resistance? I mean we can discuss it at institutional and country level.

**Respondent:** One cause for the emergence of antibiotic resistance is ***use of antibiotics by the public as if they’re OTC medications*** outside of the hospital setting or by obtaining prescriptions from private hospital physicians. The other cause is ***failure to adhere by the patient either due to availability and affordability*** or due to plain ignorance. As stated before ***laboratory unguided prescribing*** is also a contributing factor on the physician side in addition to the ***poor interrelationship between pharmacists and physicians to work together*** for a medically justified prescribing.

**Interviewer**: How do you describe the utilization of laboratory findings? Are microbiology lab results timely communicated in this institution?

**Respondent:** There are informal claims that results are sometimes not even reviewed after delivery to doctors but I think that the doctors can better answer this question themselves.

**Interviewer:** How frequently do treating physician request for cultures of relevant clinical samples to guide his/her choice of antibiotics? If less frequent, why?

**Respondent:** Laboratory tests (culture and sensitivity testing) aren’t often conducted not due to lack of awareness about their importance but possibly due to a lack of ***required reagents*** for conduction hence a ***discouragement on the physicians to send samples to the labs.***

**Interviewer:** Do you hear or have any complaints on the laboratory service?

**Respondent:** There is a scarcity in reagent supplies which restricts the service.

**Interviewer:** How do you see the existing interventions to improve antibiotics use?

**Respondent:** There isn’t any antimicrobial containment strategy in place for implementation by TASH but senior physician really do strive on their own to battle the evolvement of antimicrobial resistance on their own.

**Interviewer**: What possible measures do you recommend for better antimicrobial resistance containment practices to be implemented in this facility?

**Respondent:** Doctors should first **check for the commonest infection causing pathogens** so prescribing can be epidemiologic study based not empirical. Then they should find out the drugs that these pathogens are sensitive to by **developing an antibiogram**. The application of antibiograms by conducting epidemiologic research and studies, to guide with the use of culture and sensitivity tests. Also **continuously training of** health care professionals in a cost effective manner by which could for example be done by providing information on resistance issues in the morning sessions held 3 or 4 times weekly in TASH. The strict ***implementation of treatment guidelines*** could also serve as an important strategy but only when frequently updated guidelines are provided for the physicians to follow (this is necessary because the current guideline in use I believe is not updated). ***Another stride in this new battle against resistance could be increasing the recognition of the pharmacist’s role and its acceptance within the health care system; this could be done for example by providing pharmaceutical care section in the patient chart that is handled strictly by a pharmacist.***

**Interviewer:** From your experience, can you think of potential factors that might influence the antimicrobial resistance containment practice in the hospital?

**Respondent:** The only challenge in my opinion is going to be a ***financial or resource issue.*** For example: in terms of availing necessary medications for physicians to select from. And coming to the ***facilitators, acceptance or cooperation from the healthcare professionals is a major positive factor.***

**Interviewer:** How barriers do you anticipate if antimicrobial stewardship [full description of the prospective audit and feedback intervention; and restriction policy was given by the interviewer] is implemented in this hospital?

**Respondent:** The program is resource intensive [money, infrastructure and trained professionals] but considering its problem we can have excuses and these should not be a reason for its delay to implementation. Audit can be much better or preferable than restriction due to sustainability but  ***I think that prescribing restrictions along with prescription auditing and reporting should be carried out simultaneously for better success. I say this from experience because her in the oncology center restriction on certain antineoplastic agents is in practice and the results are very good as well as the response from the physicians. But also I can see that prescribers will have a hard time accepting auditing (suggestions) on their prescriptions at least in the beginning of this practice but it will hopefully resolve with time. Other challenges also include: lack team work culture, lack of infectious disease trained pharmacists, challenge to incentivize of professionals working the team or otherwise there will problem of commitment; and shortage of supplies and medicines.***

**Interviewer:** What other barriers and enabling factors do you think are there?

**Respondent:** The ***involvement of clinical pharmacists or at least their availability*** for when their involvement is required is a positive factor to help implement this prospective auditing and feedback intervention practice. And also the ***current antimicrobial resistance problem we’re facing will help draw attention to the problem and help gain acceptance*** for this method approach to resolve it. Another facilitator is the ***presence of DIC***. The challenge in implementing this approach could be ***lack of an actively functioning drug and therapeutic committee, poor teamwork or integration amongst the healthcare professionals, resource scarcity*** (that is the program could require additional man power or incentivization for the team or staff) or provision more reagents for the laboratories in addition to more working hours for this approach. ***Acceptance from the physicians could also be a challenge but all these will resolve with time as the program proves itself producing more good outcomes.***

**Interviewer**: That is the end of my interview, is there anything you would like to add?

**Respondent:** advise you if you could give more attention to work being done on the current infection preventive strategies as well as methods to further improve them with special focus on chronic infection patients with co morbid illnesses.

**Interviewer:** Thank you.

=================================================================================

## Pharmacist KI#02

Age: 26 years

Sex: Male

Department: Emergency Department

Staff Position: Clinical Pharmacist

Total work experience: 4 years

Number of patients with prescription dispensed per day: 40 patients

Number of patients with ABX prescription dispensed per day: 10 patients

Duration of Interview (Minutes): 59:37 Minutes

**Interviewer:** Thank you. So to start first, how do you describe the current use of antibiotics in this institution or at country level?

**Respondent:** I believe there is a problem in the current use of antibiotics both institutionally and country level; this can be attributable to major players that are the prescribers, the patients, and the suppliers.

**Interviewer:** Do you think inappropriate use of antibiotics is a problem? Which problems are more common and why? How do you describe the pattern of this problem over time (Is it increasing, decreasing or stable)?

**Respondent:** Yes I do. The ***prescribers aren’t evidence based but rather have trendy prescribing habits*** [prescribing only from others or their own experience at some point in time not based on evidence], the ***patients tend to use POM like OTC*** medications often prescribed for common infections & compromise their pharmaceutical care whereas the ***supply system due to inability to provide the necessary medications*** force prescribers into prescribing medications they do not believe to be the best option (force them to use the third or fourth generations due to absence of first and second line treatments). **Antibiotic use is increasing over time in task**, this could be attributed **to poor definitive diagnosis practice of prescribers** hence an empirical antibiotic treatment, it could also be because of the changing epidemiology of, the disease, the third could be due to prescriber misperception of the importance and safety of antibiotics, overuse or a ***disregard of the risk of resistance in spite of their awareness.*** Especially in ED over use of antibiotics is very and this is aggravated due ***poor communication among caregivers especially during shift-hours where the new physician may add other antibiotics without checking which antibiotic had been prescribed. Also we have poor documentation.***

**Interviewer:** How do you compare the use of broad vs. narrow spectrum antibiotics? What are the potential reasons for the preference of broad spectrum antibiotics? What factors do contribute for the these problems?

**Respondent:** In general overuse and broad spectrum use of antibiotics is increasing over time. I can say, except few patients, anyone coming to TASH took at least one antibiotic but in actual terms s/he may not need antibiotics. ***Broad spectrum use is preferred in TASH this is because one the treatment is empiric and two they believe that the patient will benefit from a wider coverage of the drug in treating the disease.*** The reason behind empiric treatment is that there is ***delay of laboratory result*** and an ***overcomplicated presentation of patients*** that come to TASH which lead to masking of the exact disease hence a ***poor definitive diagnosis*** rendering empiric treatment the only option. At the same time ***microbiology tests are not affordable for the patient***. There is also **a *lack of good antibiotic supply,*** and ***patient misconception*** that a successful treatment is one with a parental route of administration only. ***Another important thing is the perception and precaution of prescribers that antibiotics are the safest medicines;*** I mean even they prescribe as if antibiotics are harmless agents and say let me try this and that. It is this reason that they are considering as OTC like paracetamol. So why don’t we consider them like other drugs like NPS, anticancer and warfarin? ***For this they know the immediate effect and there is a close follow-up bit to me AMR is much worst problem than this problems but there is no such understanding among all including the management***. This is something that should be addressed.

**Interviewer:** How do you describe patients’ pressure to prescribe or not to prescribe antibiotics to themselves or their family?

**Respondent: Some but not most. Yes especially if they know that the antibiotics are administered to them as injections. *Patients believe that if they get IV medication, the physician knows their problem.*** Yes, most patients due to prior experience with IV medication fast relief request for IV rather than PO. It is not about antibiotics or other medicines but the route of administration matters. They prefer IV instead of PO. On the other hand, Some patients are aware of the problem of drug resistance and also the cost problems they would face in the future due to the resistance from the first and second line treatment options they believe they should be treated with now. few, well-educated patients are now asking about appropriateness of antibiotics and I have encountered one patient asking me why the physician gave him a potent and costly antibiotics***. At that time the patient had tonsillitis with Augmentin prescription and he was wondering why he got that expensive and potent medication while he can take Amoxicillin.***

**Interviewer:** from your experience, how do you describe the current status of antimicrobial resistance (at institutional and national level)? How serious is the problem? How frequently do you encounter drug resistance organisms within your clinical practice?

**Respondent:** Although I may not be evidence based, from my experience I can see antibiotic is an increasingly growing problem. Specifically like ceftriaxone is not responding to almost to all patients and this is mainly due to its extensive uses. You can see the magnitude of resistance from the fact that we see many treatment failures. During my clinical round I frequently encountered patients who were taking ceftriaxone but lately result of culture indicates it is already resistant to that antibiotic but the actual practice is in the absence of such culture results [which is not common] the patient continues to take that medication for longer time or additional antibiotic is given blindly.

**Interviewer:** Which types of pathogens/infections do you think have more resistance profile? And which samples do you see a lot more reports of antibiotic resistance

**Respondent:** E.coli and streptococcus are the most common.

**Interviewer**: In which samples do you see a lot more reports of antibiotic resistance?

**Respondent:** In blood samples.

**Interviewer**: Do you face any challenge in your daily practice as a result of antimicrobial resistance? How does it affect you? How does it affect the patient?

**Respondent:** It affects the practice because it decreases both the cure and survival rate of the patient, and the patient due to incurrence of unnecessary cost due to resistant pathogens which now require medications unavailable in TASH.

**Interviewer**: What factors do you think are contributing to increasing emergence and spread of antimicrobial resistance?

**Respondent:** Resistance spread is such a growing problem because ***antimicrobial stewardship is not being given the proper emphasis or even recognition*** that it should be receiving hence awareness about proper antibiotic use is very low amongst health care professionals. ***It is this lower attention that make physicians to think antibiotics are safest medication but why don’t we give attention to them at least like warfarin and others; to me consequence of AMR is much worst that these medicines.*** There is ***no training to healthcare providers and there is no up to date data*** on the resistance profile of pathogens. ***And also physicians whether its due to budget or availability issues, they are forced to use a single type of antibiotic for all sorts of cases.*** The other issue is also ***ignorance on infection prevention***. Infection prevention was only started about a year ago in TASH even though it could highly reduce the problem of resistance. The ***very slow laboratory result return*** (culture sensitivity tests result) is also another challenge that pushes prescribers to treat a patient empirically until the results get back to them for consultation. Sometimes certain antibiotics requiring special attention are brought into TASH pharmacies and at this time, the administration is conflicted on deciding to whom they should grant the prescribing authority but I feel that not only these types of antibiotics but all the rest too should be given attention and concern to battle antimicrobial resistance.

**Interviewer**: How do you describe the utilization of laboratory findings in the diagnosis of infectious etiologies?

**Respondent:** Not many of the doctors rely on the laboratory results conducted here in TASH and they don’t really rely on them for diagnosis. Culture sending is not very frequently and I always wonder why the culture of sending specimen is that much low? Of course there is delay in reporting and the system is weak. microbiology lab results are not timely communicated

**Interviewer**: Do you experience any complaints on the laboratory service?

**Respondent:** The trust in laboratory results is very low and this is one of the factor for less utilization of microbiology in the hospital. The very slow culture sensitivity test result probably due to high work burden of the laboratory is a major challenge.

**Interviewer**: Do you have concern in the way antibiotics are prescribed and used today? Do you think you can impact the problems of antibiotic use? If yes, in what ways?

**Respondent:** Yes I do. Yes, all medications that patients take should be monitored by healthcare professional in every ward and as a pharmacist I can do that to help.

**Interviewer**: What possible measures do you recommend for better antimicrobial resistance containment practices to be implemented in this facility?

**Respondent:** There really isn’t much done in TASH to improve awareness on resistance or much activity seen to control it but the hospital is in fact working on infection prevention, but I do not know if the perspective is from an attempt to battle resistance or not. ***Encouraging accountability*** for the problem of resistance hence ***promoting teamwork*** amongst the physicians, pharmacists and laboratory technicians as all three are all key players is very important in my opinion; integration amongst these three adding the ***hospital administration*** as a fourth key player to setup a system of control for this problem would be a great solution. Another important issue is **creating awareness** and an awareness shift to correct already established misconceptions from health care professionals ***i.e. we have to convene them that antibiotics are precious agents that should use wisely.*** Establishing a collaborative relationship with external organizations that have microbial stewardship programs and hiring professionals specifically dedicated to this cause to monitor the antibiotic use within the hospital is possible too. ***Establishment of a team dedicated to monitor antimicrobial use is also good which would give an opportunity for other professionals to contribute b/c physicians are complaining of patient load.*** Regarding the application of ***antimicrobial guide though useful it might be, the consistent updating and dissemination*** of the updated version to doctors in a timely manner is in question so the use of literature review or research evidence based prescribing guide is I think a better practice and should not be discouraged since TASH is a tertiary health care institute and the physicians should not be restricted to prescribe from guidelines only.

**Interviewer**: How do you see if antimicrobial stewardship [full description of the prospective audit and feedback intervention; and restriction policy was given by the interviewer] is implemented in this hospital?

**Respondent:** I believe both are very important and useful to battle resistance problems. Auditing can indeed save patients from unnecessary antimicrobial use and improve patient outcomes*.* ***But restriction is also important for early prevention and you know the proverb that prevention is better that cure.***

**Interviewer**: Can you help us understand the potential factors that might influence the implementation of these interventions.

**Respondent:** Regarding hindering factors to the prospective audit and feedback intervention the first factor is ***neglect of the responsibility by health care professionals***, the second is ***lack of resources even if specialists were to be assigned for the job*** and ***autonomy*** also poses be a question when the physician involved is an intern, resident or a junior one but prescriber autonomy poses no threat with senior physicians. ***So I do not believe acceptance by physicians would be a problem in implementing this approach especially the seniors do care more about the patient not about their dignity or profession[unlike junior physicians] as far as this approach brings benefit it will have full support.*** With regards to the laboratory practice in TASH though since audit and feedback should be functioning based on laboratory findings the ***mistrust on the TASH lab results by physicians is indeed a problem*** and should be corrected by improving the quality of the laboratory itself, the professionals and the practice. ***Another problem with antibiotics restriction is, yes definitely there will be a decrease in antibiotic use [we have seen from our experience when linezolid injection was restricted, we had huge expiry in the initial phase as procurement was not adjusted] but at the same time it could an unprecedent impact on the patient outcome as the assigned authorizing physician may not be always available.*** But even with those problems, restriction works. However, auditing is much better as there is no problem of discontinuity of care without authorizer. I have an experience that a patient was taking one drug (ceftazidime) with different brand b/c of different sources. with regard to guideline, I think this doesn’t work for teaching hospitals as they can make new decision based reading/doing systematic review or meta-analysis. But otherwise, where is the meaning of teaching hospital, it should be an open system that can adapt new treatment option and I think it would be difficult to control the prescriber using guidelines. Lack of resource, poor microbiology infrastructure, ***lack of on job training especially pharmacist specially trained in infectious disease may not be available and how can you bring them together with a subspecialty physician together on the same issue***, lack communication channel b/n different professionals are also barriers. ***Specifically for restriction the barriers are lack of adequate number of expertise is the bottleneck.***

**Interviewer**: Ok we discussed on the challenges but what about the enabling factors?

**Respondent:** As there are hindering factors there are also supportive factors for the implementation of these interventions. One is support from TASH management b/c this is now core area, the laboratory professionals and the senior physicians willingness to implement such quality improvement interventions are a good opportunity for its implementation.

**Interviewer**: That is the end of my interview, is there anything you would like to add?

**Respondent:** Studies on other contributing factors like drug supply management, drug use evaluation, antibiotic selection and antibiotic use pattern could help battle the antimicrobial resistance problems well.

**Interviewer**: Thank you.

=================================================================================

## Pharmacist KI#03

Age: 40 years

Sex: Male

Department: Pharmacy, ICU

Staff Position: Clinical Pharmacist

Work experience: 16 Years

Number of prescription dispensed per day: 16 patients

Number of patients with ABX prescription per day: 15 patients

Duration of Interview (Minutes): 54:23 Minutes

**Interviewer:** Ok, Thank you. So first let’s start discussing on antibiotic use. How do you describe the current use of antibiotics in this institution and generally in Ethiopia? Can you help us understand how antibiotics are used?

**Respondent:** So as we all know Tikur Anbesa specialized hospital is the largest hospital in our country. I am working in ICU and we see antibiotic use since the patients are critical, the use of antibiotics are started empirically and broad spectrum antibiotic are initially preferred as they wanted to have wider coverage. Major prescribers are residents. The residents prescribe and culture is sometimes sent for sensitivity. ***Blood culture comes after ten days and urine culture comes after 3 days in Tikur Anbesa. Quality of***  laboratory findings are even not reliable. Sometimes they finish it by empirical treatment. If the patient does not improve, additional antibiotic is prescribed for him/her.

**Interviewer:** How do you use see the general use of antibiotics? Is it overuse, under use or normal?

**Respondent:** We can say there is both overuse and underuse practices but overuse is very common. ***Personally, one time I faced a person/patient whose culture result indicating that she was resistant to all antibiotic available in Ethiopia***. I think both overuse and under use of antibiotics are contributing to development of more resistant pathogens. So the present use is generally irrational. Overuse is mainly due to fear of infections as the pathogen is not well identified. Addition of antibiotics is based on sign and symptoms like if there is fever then they add antibiotics which I think is irrational.

**Interviewer:** Do you think irrational use of antibiotics is increasing or decreasing from time to time?

**Respondent:** It is increasing. There is a delay of result from laboratory result (culture)which is a barrier to identify the exact bacteria and this leads to prescribing more and/or wrong antibiotics

**Interviewer:** What do you think are the reasons for use of overuse and broad spectrum use of antibiotics? I mean you were telling me before but just to get more clarity.

**Respondent:** One thing is related to consultation. For example a resident (R1) cannot directly consult the senior physician. The channel is R1 consults R2 and R2 consults R3 and finally R3 consults senior physician. ***This takes a very long time [exaggeratedly long..] while the patient is at critical condition, so the resident prescribes the broad spectrum antibiotic without considering its consequences and only focusing its immediate cure, I mean from prescribing the broad spectrum [with wider coverage].*** They have tried to discuss on the hierarchy of consultation but they could not come up with a solution. There is also lack of knowledge from some R1 students [prescribers]. So s/he can’t directly consult the senior physician, then they prescribe the broad ones.

**Interviewer:** How about availability of drugs in the pharmacy store?

**Respondent:** When we see the availability of drugs actually there is shortage of drugs. And this is related to management and PFSA [Pharmaceutical Fund and Supply Agency]. For one thing there is shortage of supply from the sole public procurement agency and if the medicine I stocked-out from PFSA, the medicine should be procured from other suppliers but the hospital management doesn’t allow you to do so. And the management don’t want to bypass the PFSA even in its stock-out. The management cannot buy drugs from the market. The only option is using what is at hand. This leads to irrational use of drugs b/c there is no option.

**Interviewer:** How do you describe patients’ pressure to prescribe or not to prescribe antibiotics?

**Respondent:** This is related to the psychology of patients and he/she may not take the ordered drug due to the attitude I mean the patient may say it will not heal me. There is a pressure to fulfill their need but its influence is rare.

**Interviewer:** Do you think there is pressure from patients to get IV antibiotics instead of PO? Why do you think so?

**Respondent:** With IV usually there is a fear of resistance. Thus you change from IV to oral but not vice versa. But sometimes it is related with psychology of the patient, a patient prefer to give them IV antibiotics, they think it is better. ***Sometimes it is done for the psychology of the patient as you don’t have the choice and if refused and prescribe PO instead of IV the patient will throw it away [will not use it], so you don’t have the choice but to prescribe IV. However such practice is rare.* *Sometimes, I have seen that prescribers prescribe the patient preferred antibiotic to satisfy their need and then add another antibiotic which they feel is necessary to treat the expected infection.***

**Interviewer:** So from your experience you mean there are patients who preferred IV?

**Respondent:** Yes, although not a larger number, some patient preferred IV. This is mainly due to their expectation of more effect from IV.

**Interviewer:** Do patients ever express concern about themselves/ their child being taking antibiotics?

**Respondent:** I did not encounter such things. Even the professionals are fast to prescribe antibiotics which is up to the expectation of the patient and I did not get such kind of patients who refuse or fear of antibiotics.

**Interviewer:** How do you describe the current status of antimicrobial resistance (at institutional and national level)? How do you describe the magnitude and how serious is the problem?

**Respondent:** ***If I should tell the truth, after some time, it will be difficult to treat patients in Ethiopia.*** B/c resistance is extremely increasing. Antibiotics are being prescribed empirically. The tradition of prescribing narrow spectrum antibiotics based on culture and sensitivity test is poor in our setup. ***Sometimes one physician prescribes broad spectrum without checking or plan for how many days the patient to take the medicine. I personally see patient who took meropenem and ceftriaxone for forty days. Once the patient starts antibiotics s/he continues with that medicine and no adjustment is done as there is no clear follow up. So resistance is highly [exaggeratingly high] increasing.*** ***When I ask for means to correct, they are blaming one another. The nurses say we give as the prescription and physician say no we changed it but nurses don’t change/adjust the antibiotics.*** Finally, when the culture and sensitivity result comes, the patient is resistant to all antibiotic available in Ethiopia. The antibiotic colistin for pneumonia was not available in Ethiopia. Such kind of cases, one or two, occurred in ICU. This indicates antibiotic resistance is very increasing. These time Klebssila pneumonia is becoming resistant to all antibiotics in ICU.

**Interviewer:** So are you telling me resistance is increasing overtime?

**Respondent:** Yes, it is increasing.

**Interviewer:** In which samples do you see a lot more reports of antibiotic resistance (urine, blood, sputum, pus or other body fluids)?

**Respondent:** Samples are taken from blood, urine and sputum. Antibiotic resistance is mostly seen on blood samples. But with urine it mostly associated with contamination. Klebssila is common pathogen but I am not about the other pathogens.

**Interviewer:** Do you face any challenge in your daily practice as a result of antimicrobial resistance? How does it affect you? How does it affect the patient?

**Respondent:** You observe resistance when the patient is desolates [deteriorated] overtime. If the patient does not improve it is indication of resistance. When you add another antibiotics you affect the normal flora and the patient will be harmed. This leads to another new adverse effect like diarrhea which necessitates another antibiotics. So there is disease complication and demanding additional antibiotics.

**Interviewer: But w**hat factors do you think are contributing to increasing emergence and spread of antimicrobial resistance?

**Respondent:** The main reason is prescribing broad spectrum antibiotics. There is no restriction for the broad spectrum prescription like I told earlier there is no good communication between (R1, R2, R3, senior). The major problem is who should prescribe broad spectrum and whom to consult. Sometimes there could be drug interaction. R1 cannot directly consult the senior physician and lack of knowledge on R1 has negative impact on the patient outcome. ***Lack of knowledge and poor communication [R1 can’t directly consult senior physician or ID] are highly contributing to increasing resistance.***

**Interviewer:** Ok, you told me issues related to prescribing what other factors do you think are there? How about infection prevention practice?

**Respondent:** There is a big problem related with infection prevention practice. For instance patient with resistant to acetobacter are easily contacted and so poor infection prevention has impact in increasing AMR.

**Interviewer:** You told me shortage of medicines, lack of knowledge are factors for increasing the problem. Also you mentioned about the laboratory including its quality is contributing but how do you see the effect of collaboration among healthcare professional (presence or absence of strong team work in the hospital)?

**Respondent:** Yes. In our case the physician is dominant and the communication usually among themselves. Role of other professionals like pharmacist is not yet strong. For instance, only one pharmacist is working in ICU. Currently physicians are requesting for presence of pharmacist as they are recognizing their roles especially in selection of antibiotics and drug interaction. But in my opinion, the right professionals are not efficiently used. I think every health professional has the capacity to feel the knowledge gap in the service provision.

**Interviewer:** What do you suggest to decrease the increasing antimicrobial resistance?

**Respondent:** To decrease this, we are planning to organize a workshop but I don’t see concrete actions being taken to alleviate the problem. So decrease the resistance there should be a special list of broad spectrum antibiotics and should be restricted, shouldn’t be allowed to be prescribed by anyone. It should be the specialist that must prescribe from the special list. The second thing is the diagnosis should be identified, I mean the common pathogens should be identified. The culture result should be available within 48-72 hours and use of broad spectrum should be deescalated. The laboratory facility should be strengthened. Another thing is the follow-up i.e. the adherence of the patient should be followed.

**Interviewer:** If you think of implementing antimicrobial stewardship […after describing what antimicrobial stewardship is and different approaches…], which one do you prefer to be applied in this hospital: restriction or audit and feedback team or both?

**Respondent:** I mentioned about the need of restriction but we also should audit the changes happening in the patient after the antibiotic is prescribed. If we neglect it, it is another issue. You are exposing the patient to another problem. Is the patient improving or deteriorating? If the drug is not working either the drug is wrong or there was a wrong diagnosis or the drug is overdose or under dose. ***You may restrict to selected antibiotics which is good to control but at the same time it very essential to audit the prescribing practice if the diagnosis and choice of antibiotics is right. So, both interventions (restriction; and audit and feedback)should go in parallel.***

**Interviewer:** You said both are important, but what do you think are the challenges or barriers to implement them?

**Respondent:** One challenge; when it is restricted it may result for prescribers to assume that is not my responsibility and may not discharge personal responsibility. The problem is in the seniors, the rest they are students, they simply ask and do. There is a fear from residents related to their result when asking the seniors. If restriction is applied, resistance is from the seniors but there is no resistance from lower. ***The senior may say to the residents why don’t you manage it by yourself. Hence, due to fear of the lower level staff they may opt to do it by themselves instead of asking seniors. So the resistance is manily from the senior physicinas.***

**Interviewer:** What other challenges do you see with restriction policy?

**Respondent:** Another challenge with restriction is if the resident waits for the senior for special order then the patient will be harmed. So, the problem is what to do if there are immediate problems to be solved, if the authorizing physician is not available.

**Interviewer:** Do you think the management will accept restriction?

**Respondent:** I think this is a must b/c it is a life. ***It is not only about an individual’s life but it also affects the country. If resistance develops, it will have an economic burden.. If we do not use wisely what we have, we might face a problem in the next time. Thus, the management should accept restriction for the sake of the patient and the country’s economy.***

**Interviewer:** Let’s talk about prospective audit and feedback (which is team approach), what problems do you think it has?

**Respondent:** When it comes to team work, the problem is related to managing and bringing teams together. It has advantages where everyone has contribution to the patient care but some members of the team may not come due to other commitments or probably assuming that his/her role will be covered by other group members. ***Another is related to payment; they should get paid for what they do on the basis of part time and the management should arrange this. Also there is a knowledge gap especially in the pharmacy professionals. Pharmacists are still in the traditional dispensing while the clinical exposure is minimal. This might be a challenge to bring all team members on the same page. I mean how can we expect the contribution of degree holder pharmacist to be on the same page with subspecialty/consultant physician. But I believe this can be solved by providing specific training, if the interest is there and planned accordingly.***

**Interviewer:** How would you see if prospective audit and feedback is applied in this institution and a multidisciplinary team review and give feedbacks on prescribing?

**Respondent:** In the past there was a problem. The physicians were saying I should prescribe/order everything but now they are accepting the professional comments from others. However, this does not mean they all accept, I am speaking about ICU.

**Interviewer:** How do you see the laboratory supply?

**Respondent:** The supply (including reagent) for laboratory is through the pharmacy unit. ***The pharmacy is not fulfilling the demand of physicians and that of the laboratory.*** Nowadays the laboratory is buying its supply by its own. However, there still problems or no trust in the quality of lab. ***There is difference of results of the hospital laboratory and when it is done in other private laboratories. So there is a growing belief that it is difficult to rely the laboratory findings.***

**Interviewer:** we discussed the barriers. Can you help me understand the favorable conditions to implement restriction and audit and feedback in the hospital?

**Respondent:** A strong team must be there for example in the pharmacy work force. But there is no strong team. I think we are creating the proble and we should solve the problem. Unless we solved the problems no one will solve them and create us the favorable conditions. ***Since we created the problem, we are the once who should create the favorable conditions and we should solve the problem..***

**Interviewer:** If you have anything to add please.

**Respondent:** I have nothing new to add but this is a big institution. We should use the huge potential at school of pharmacy. It is a big resource.

**Interviewer:** Thank you!

=================================================================================

## Pharmacist KI#04

Age: 26 years

Sex: Male

Department: Pharmacist [Medical, no-surgery, Clinical Pharmacy]

Staff Position: Lecturer and preceptor of clinical pharmacy

Total work experience: 4 years

Number of patients treated per day: N/A

Number of patients with ABX prescription per day: N/A

Total Interview time (duration): 59:41 Minutes

**Interviewer:** Thank you. In general I would like to know the overall use of antibiotics, how do you describe the current use of antibiotics in this institution or at country level?

**Respondent:** from my experience, ***antibiotics are medicines used without interruption in almost all wards for almost all patients.*** They are the one procured in higher quantity but they are the one stocked out quickly. The is an extremely high use of antibiotics within TASH. I think it’s exaggerated. It is also trendy a kind of fashion use, for instance this week you can see many of the patient prescribed with pep/tazo continuously and when this medication is stocked out all of them will again shit to same drug.

**Interviewer:** Do you think inappropriate use of antibiotics is a problem? How do you describe the pattern of this problem over time (Is it increasing, decreasing or stable)?

**Respondent:** Yes there is a problem of antibiotic use in Ethiopia which is rapidly increasing too.

**Interviewer:** How do you compare the use of broad vs. narrow spectrum antibiotics? Do prescribers tend to prescribe very broad-spectrum antibiotics empirically and/or to over prescribe antibiotics?

**Respondent:** The antibiotic prescribing behavior of doctors in TASH is high. Doctors in TASH prescribe majorly based on availability (their prescribing is highly influenced by availability), as soon as they get a notice from the DIC that a certain antibiotic is now available they will immediately start prescribing it.

**Interviewer:** What are the potential reasons for the preference of broad spectrum antibiotics? What factors do contribute for the overuse of antibiotics?

**Respondent:** They tend to go for broad spectrum because of ***unavailability* of narrow spectrum or older generations of medications** in TASH pharmacies, it is the choice of drug majority of the time because doctors ***aren’t very sure about the focus of infection ( they have poor diagnosis certainty), culture and sensitivity test are not conducted*** in TASH hence there is poor identification of the infective pathogen and drugs it is sensitive to, another factor is the ***high severity status of patients*** who come to TASH with more resistant infective strains which will possibly have masked culture and sensitivity results even if tests were to be conducted upon admission (because the patients have already been to 1° or 2° health care institutions and have been exposed to antibiotics rendering culture and sensitivity with no growth results misleading and less valuable). ***Patient pressure in light of eagerness for relief from pain is also a provocative factor to go for immediate empiric treatment with broad spectrum antibiotics. Doctors tend to immediately shift to newly incoming potent antibiotics because they believe they have better activity against resistant pathogens but they also do it to simplify their work by avoiding prescribing less potent yet possibly effective agents. Lack of data on pathogen distribution in the hospital set up is not known, so they prefer to empirically prescribe broad spectrum antibiotics fearing of missing infection and patient complains; it is the patient immediate relief that worries the physician not the resistance. Absence of antibiotic restriction i.e. where anyone can prescribe any antibiotics for instance intern can prescribe meropenem, is contributing factor.***

**Interviewer:** How do you describe patients’ pressure to prescribe or not to prescribe antibiotics?

**Respondent:** Yes patients do have influence in antibiotic use. As I mentioned earlier they pressure for drug use from eagerness to get relief from pain. Some patients also show reservations towards certain drugs if they’re associated with some discomfort but they aren’t really interested in avoiding antibiotic overuse or risk of developing resistance because they are not aware of the problem.

**Interviewer:** What about their preference on the route of administration? Do you patients prefer IV or PO? Why do you think so?

**Respondent:** Yes, there is high preference of IV. Patients usually express their preference to use IV drugs since most patients visiting TASH come from rural areas & their ***concept of effective medication is in IV form only.*** During round they complain as if they are not taking any medicine, if it is PO, in that sense patients are equating treatment only getting the right IV medicine. Yes, they don’t know what the medication type is but they need IV. So even you give them water for injection or glucose they think that they get the best treatment as far as it is injection type. So, inpatient don’t prefer PO antibiotics. Patient don’t know the drugs type and there is no fear of antibiotics consequence and the need to avoid taking these drugs to them or their family member.

**Interviewer:** How do you describe the current status of antimicrobial resistance? Do you think it is a growing public health treat or not? How do you see the pattern of resistance over time?

**Respondent:** I would describe the current use of antibiotics is inappropriate. Yes, professionals should be worried because there is a very frequent antibiotic misuse and hence the consequence of resistance is increasing. Patchy research findings are indicating that there is a growing evidence of increasing AMR. Awareness is now growing, and there is concern of AMR now but still we are talking and no visible action taken is taken. I have heard there was a study and that indicated the resistance of ceftriaxone was about 85% but that doesn’t considered as serious issue. ***The problem is also increasing overtime. Another example is previously almost all physicians were prescribing vancomycin but they heard that there is resistance, I am not sure if it is right, then anyways everybody shifted to meropenem. But the big fear is what will happen choice will we have if this drug developed resistance due to our irrational prescribing at all levels including lower level staff. Prescribing is like fashion I think it is increasing overtime.***

**Interviewer:** From your experience which types of pathogens/infections do you think have more resistance profile? And in which samples do you see a lot more reports of antibiotic resistance?

**Respondent:** Klebsiella pneumoniae & methicillin resistant staphylococcus aureus (MRSA) are the two most common pathogens that have developed resistance within the TASH setting. Plus E.coli & pseudomonas aeruginosa are common pathogens too. E.coli should have been sensitive to lower antibiotics but currently it is a common resistant to almost all antibiotics except meropenem.

**Interviewer:** And in which samples do you see a lot more reports of antibiotic resistance?

**Respondent:** Blood and urine are common.

**Interviewer:** Do you face any challenge in your daily practice as a result of antimicrobial resistance? How does it affect you and the patient?

**Respondent:** *The fact that there is an increasing drug resistance problem is increasing negatively affects daily practice because it decreases patient satisfaction due to therapeutic failure as well as the trust in the healthcare system which decreases the health seeking behavior of patients [may say they don’t want to go to health facilities] & it’s also a wastage of resources if drugs bought in cease to cure anymore. After all health professionals’ satisfaction will be lowered since they don’t see good patient outcomes.*

**Interviewer:** What factors do you think are contributing to increasing emergence and spread of antimicrobial resistance?

**Respondent:** The first issue is the ***irrational use of antibiotics and this is due to the unrestricted prescribing allowed,*** even interns are allowed to prescribe very potent drugs. The second challenge is ***lack of appropriate information dissemination or appropriate personnel assignment for the job [sometimes there is pharmaceutical promoters influence]***, the third challenge is that there is ***no culture & sensitivity testing*** and the other factor is inaccurate & insufficient data on which microorganisms are resistant to which drugs i.e. ***lack of institutional antibiogram;*** hence the ***prescribing is done blindly or unguided in addition to unorganized consultation process and affordability issue for patients.***

**Interviewer:** How do you describe the utilization of laboratory findings? How frequently are samples sent? Are results timely communicated?

**Respondent: Sending samples, not very frequently. Because results aren’t timely communicated and there is poor quality of work done by TASH laboratories.** Results are not timely communicated.

**Interviewer:** What do you think are the reasons for lower utilization? Do you hear any complaints about the service?

**Respondent:** The ***mistrust in the quality*** of laboratory results could either be due to professional ***incompetence or poor infrastructure of the laboratories*** themselves. ***There aren’t sufficient reagents or disc required to conduct culture and sensitivity tests which is why the laboratories have difficulty providing efficient service.*** On the other hand, there are discs for antibiotics which are not available in the hospital like they have disc for **Amikacin** while there is no disc for the other important antibiotics.

**Interviewer:** If we need to control the problem of resistance, what measures do you recommend for better antimicrobial resistance containment practices to be implemented in this facility?

**Respondent:** I can say there is no concerted effort to tackle the problem. We may have committees like infection prevention committee but they are not functional. In order to work on infection prevention it’s necessary to first create a conducive environment within the hospital setting***, this is because the hospital itself acts like an incubator for infective microorganisms***. We also need to work on availing the important antibiotics all year round so that doctors can prescribe without having to deal ***with scarcity as a limiting factor.*** There should also be a ***proper waste disposal system*** in place and also an effort to improve the laboratories in TASH. ***Disposal is a big problem, let alone antibiotics even we found anticancer drugs expired in 1970s. you can find expired medicines anywhere even in nursing wards. This poor disposal practice have an impact in antibiotic resistance.***

Assigning responsible individuals to run this practice from every department and ***establishing a coordinated team*** ***to create an appropriate consultation service and to create a restricted prescribing practice*** by setting up a controlling mechanism that sets a prescribing hierarchy would be best. Restriction policy is very essential but ***Restriction on prescribing could face challenges like resistance from the physicians, patient complaint due hustle especially in emergency departments, since the prescribing doctors might not be easily available. In addition, the infrastructure of the microbiology should be improved.***

**Interviewer:** From your experience, can you think of potential factors that might influence the antimicrobial resistance containment practice in the hospital?

**Respondent:** There is ***nothing done by the TASH administration to combat the problem of drug resistance and there is no coordinative effort amongst the hospital health care teams (i.e. the pharmaceutical care, the nursing care and the medical care)*** which could be a limiting factor even if the antimicrobial resistance containment practice were started. ***Accountability is also lacking and there is no system to monitor problems of antibiotics misuse or abuse in the hospital.***

**Interviewer:** How would you feel if antimicrobial stewardship program [the interviewer gave detail description of the program: restriction policy and prospective audit and feedback intervention] is s applied in this institution? How do you recommend us to implement these two programs?

**Respondent*: I don’t think prospective audit and feedback interventions would be easy to be implemented in the hospital because it would offend a lot of doctors. If you make auditing there will be resistance, they would say it is overriding their autonomy and even they think as they are defeated. I think it would be better to implement a face to face discussion prior to the prescribing practice rather than to have audit what the physician has already prescribed in writing, it would compromise their autonomy. Physicians also would prefer to avoid questions of ethical misconduct. You know ethics is now a big issue and physicians are highly alerted about this, so they don’t want to be told or audited for what they did as they may fear identified possible errors can be used to sue them and make them accountable by the ethics committee or the court.***

**Interviewer:** Can you help us understand the potential factors that might influence the implementation?

**Respondent: *Cost is a big barrier*, plus the professionals aren’t well aware and there is *no baseline data to start the stewardship* from or even to set goals, so I personally feel that it would be better *to focus on the restrictive prescribing practice as prospective auditing would be too costly for our economic capacity. I think prospective data is more applicable for developed countries where professionals are well aware, they have no problem of cost and baseline data. But for us, restriction of these valuable antibiotics is a must to do for us but later we can implement the prospective audit and feedback. The other problem could be, the availability of incentive to all team members. Yes, there will an improve in outcome but unless you incentivized them it would be difficult to have their commitment. Unavailability of antibiotics is a challenge to implementation, even if you include pharmacist in this team physicians may discourage the pharmacist why s/he is in the team while antibiotics are not available. Changing the existing trend by it self is a challenges, there will be resistance to change what know for long time.***

**Interviewer:** We discussed on the barriers, what do you think are the enabling factors?

**Respondent:** Conducive factors for important for implementation of this program, I believe, are the **fact that antimicrobial resistance is a hot issue right now both nationally and globally, the fact that it is possible to show positive outcomes and then support in shorter time as there is widespread inappropriate use antibiotics in the hospitals, *acceptance from professionals since it’s a fresher gear and the result will be satisfactory for everyone*, *there is sufficient manpower to booth into work for the success*** of the stewardship program and since most professionals work part-time in other private institutions they can serve as expansion tools for the program and help this rise to government official ears to make it a national practice and help make policies in support of the program [ i.e. the benefit can have a spillover effect]. There will be good acceptance from the policy makers.

Another major positive factor is the link between TASH and the college of health science, this link helps provide a lot of accommodations that can help fill the gap in this program by conducting research and the like. The fact that a lot of other institutes come to visit the school can also help the stewardship program get attention and support from external bodies that will further serve its success. TASH is a three-dimensional institute that has all the bright lights shining on it in terms of, approval from ministry of health and the government which makes it a trend setter and one of the best environmentally supportive institutes.

**Interviewer:** Thank you.

=================================================================================

## Pharmacist KI #5

Sex: Male

Department: Pharmacist [Pediatric, Clinical Pharmacy]

Staff Position: Lecturer and preceptor of clinical pharmacy

Total work experience: 7 years

Number of patients treated per day: N/A

Number of patients with ABX prescription per day: N/A

Total Interview time (duration): 39:41 Minutes

**Interviewer:** How do you describe the current use of antibiotics in Ethiopia and particularly in this institution?

**Respondent**: Hospital setup specially at impatient there is high utilization of antibiotics and also limited type antibiotics are over used. Example Ceftriaxone, just because it’s available, easy for use and good experience it’s abused. When new antibiotics are available at the market prescribing focuses on them.

**Interviewer:** Do you think inappropriate use of antibiotics is a problem in Ethiopia and in this institution? Which problems are more prevalent or common in this setting? Why? How do you describe the pattern of this problem over time (Is it increasing, decreasing or stable)? Why?

**Respondent**: it is a current challenge of the country. we see that it is increasing but the government doesn’t give the full attention it requires.

**Interviewer:** How do you compare the use of broad vs. narrow spectrum antibiotics? What factors for this practice?

**Respondent**: Broad spectrum antibiotics are highly used. Factors contributing include no clear guideline on use antibiotics, limitation on data available about which bacteria has developed resistance to a given antibiotic.

**Interviewer:** How do you describe patients’ pressure to prescribe antibiotics?

**Respondent**: I don’t see the influence of patient in prescribing antibiotics. They rather fully rely on your recommendation and even it exists, then it we that we should convince them that it is not good.

**Interviewer:** How do you describe the current status of antimicrobial resistance?

**Respondent**: From my experience I have observed that antimicrobial resistance is a big challenge to the health sector. And it is growing overtime. Especially, acetobacter MDR increased very highly. Hematologic malignancy and neurologic patient have high use of antibiotics and resistance development risk is high.

**Interviewer:** What factors do you think are contributing to increasing emergence and spread of antimicrobial resistance?

**Respondent**: Enough Attention is not given. There was no responsible person or team to ask question when there is unnecessary use of antibiotics, what was the measure to avoid it, also there was no regular update. There is a promoter influence.

**Interviewer:** How do you describe the utilization of laboratory findings in the diagnosis of infectious etiologies? Do you have any complaints on the laboratory service? Please help us understand those concerns.

**Respondent**: of course, the culture of using those services is very poor. To me the main reasons are: communications problem among professionals, there is a capacity problem is majorly seen in the microbiology laboratory and culture result arrives late.

**Interviewer:** What possible measures do you recommend for better antimicrobial resistance containment practices to be implemented in this facility?

**Respondent**: Organization of committed team that are recognized by the institution. Also, there is restriction experience in other departments like the oncology. There was restriction here as well on **Lipsomal amphotersin B** it was only prescribed by ID specialist. This drug is also a very expensive drug.

**Interviewer:** From your experience, can you think of potential factors that might influence the antimicrobial resistance containment practice in the hospital?

**Respondent**: Enabling factors include presence students that are willing to exercise the guideline, available of antibiotics and option to use in emergency. Also, strong support from stockholders.

**Interviewer:** Prospective Audit and Feedback Intervention

**Respondent**: Restriction alone will not solve the problem so prospective auditing is also important. If the auditing and feedback is specific to ward it could improve the outcome. Having a responsible, committed ID professional to consult is a relief for the physician concussion on which treatment to process with.

**Interviewer:** That is the end of my interview, is there anything you would like to add? Thanks, you.

**Thank you for your time!!!**

=================================================================================

## Pharmacist KI#06

Age: 32 years

Sex: Male

Department: Pharmacist [Clinical Pharmacy]

Staff Position: Lecturer and Clinical Pharmacy Preceptor

Total work experience: 10 years

Number of patients treated prescription per day: N/A

Number of patients with ABX prescription per day: N/A

Total Interview time (duration): 44:49 Minutes

**Interviewer:** Ok thank you. To start with my first question, how do you describe the current use of antibiotics in Ethiopia and particularly in this institution?

**Respondent:** From the drugs we have, in our hospital for example, even it is not based on research; from the ***drugs prescribed by physicians more than 50% are antimicrobials.*** The ***use of antibiotic drugs is increasing from time to time***. The ***reason is one, more patients are coming to hospitals; two there are more prescribers with different level of specialty. Due to the expansion of services antibiotic drugs are also highly being dispensed and prescribed.***

**Interviewer**: You said there is high use of antibiotics; but how do describe this, is it overuse, broad spectrum use or underuse?

**Respondent:** All are in place. For instance one example is there is a tendency of using ceftriaxone and cephalosporin is so ***high (overuse).*** Second, in my opinion, this common problem of antibiotic use in the community ***discontinuation or stopping*** before the time duration ordered for the antibiotics. Third is, the ***high use of broad spectrum*** b/c prescription is mainly empiric.

**Interviewer:** What do you think is the reason for overuse and broad spectrum use of antibiotics?

**Respondent:** The first is ***trend prescribing habit;*** for example in our hospital around emergency unit; if a patient comes with fever, inflammation, tenderness there is a tendency of prescribing antibiotic almost immediately. There is also a tendency of pushing patients to take the drugs from 5-7 days. That is there is no means of follow up and discontinuation of antibiotics in shorter duration. The second is, I think ***there is bad practice.***

**Interviewer:** What do you think is the reason, is lack of knowledge, facility or shortage of drugs?

**Respondent:** All could be reasons; example ***broad spectrum drugs can heal many diseases***. If a patient is suspected to have infection broad spectrum is prescribed then the patient will be re-visited and check if that is the right diagnosis. But since they ***do not revisit they keep the patient take the drug***. It is common practice ***not take culture in 48 hours***. ***The practice of changing IV into PO is very poor. Since these trends are not in place, patients are taking the drugs continuously.***

**Interviewer:** Do you think shortage of drugs influence physicians to prescribe broad spectrum drugs?

**Respondent:** Of course, yes. B/c there is limited drugs in governmental hospitals. ***There is a tendency of changing if there is an infection and if the patient complains i.e. fear of infection and believing that the narrow spectrum doesn’t work well.*** But as to my opinion without culture the antibiotic should not be changed. This is being done as common practice but there must be justification. Another big problem is, there is almost ***no re-visiting the patients***

**Interviewer:** Do you have any opinion about level of knowledge in overuse or underuse?

**Respondent: *Yes, there is knowledge gap.*** Even the new physicians are applying the old trend b/c they assume that it works. One indicator for lack of knowledge is after they prescribe the drug, they consult the senior of ID physician. So I think there is lack of knowledge.

**Interviewer:** How do you describe patients’ pressure to prescribe or not prescribe antibiotics?

**Respondent:** I did not have such experience.

**Interviewer:** How about patient’s preference of IV antibiotics instead of PO ? Why do you think so?

**Respondent:** Let me tell you; practically there is no such practice in our hospital. ***Our hospital does not have a trend of switching IV antibiotics to PO.*** However when patients are discharged, it could be done. But in their hospital stay, it is not. Otherwise there is criteria on when to change IV to PO. But from patient side, I don’t see any objection or preference.

**Interviewer:** How do you describe the current status of antimicrobial resistance)?

**Respondent:** In our hospital, as to my understanding, if the patient does not respond immediately within 48 hour, I consider there is a resistance. ***There is a trend of changing antibiotics now and then.*** As some researches show resistance ***to ceftriaxone is from 58% to 75%.*** In general resistance is a big problem but since culture sensitivity is not common our country, there is a difficulty to appreciate resistance pattern. ***But we are observing more resistance from time to time.***

**Interviewer:** From your experience; which types of pathogens/infections do you think have more resistance profile?

**Respondent:** Staphylococcus aureus, staph aureus, klebsiella and sometimes E.coli. Let me take you back, ***why we cannot appreciate microbial resistance in our hospital is most of the sent cultures result indicates no growth.*** We asked and there are so many factors why there is no growth and ***this discourages.*** They sent the sample to outside and we can’t appreciate resistance. ***From those that were sent, 50% of specimens reported no growth and this is eroding the trust of the lab.*** Most of the time samples are taken from blood.

**Interviewer:** You told me that the magnitude of resistance is high; what is the negative impact in daily practice as a result of antimicrobial resistance?

**Respondent:** The first may be ***treatment failure and the patient will stay longer in the hospital.*** Another is ***cost for the hospital and the patient***. Another is ***spread of infection due to resistance***. If there is a resisted pathogen, the resistant pathogen can easily be transmitted from patient to patient highly. Patients who developed resistance must be isolated. In general due to these reasons and others the patient may ***die or develop other complications.***

**Interviewer:** What factors do you think are contributing to increasing emergence and spread of antimicrobial resistance?

**Respondent:** They are many reasons for increasing resistance. First, ***not prescribing or selecting the right antibiotic to the right disease.*** Second, ***prescribing antibiotics without evidence***. Third, use of ***underdose and overdose***. Fourth, ***no revisit after 48 hours [poor follow up]***. Another is, health care providers starting from intern to the senior physician, everyone is prescribing antibiotics; ***there is no restriction for it***.

*When you go to health centers nurses are prescribing antibiotic and in community pharmacy the professionals there are selling antibiotics without prescription.* ***We are using these medicines like any other OTC medicines.*** *From patient side, there is a tendency of quitting of the drug quickly. From prescribers’ side, not informing the patients about the dose and duration.* ***Availability of drugs and laboratory is also another contributing factor.***

**Interviewer:** Do you think sufficient job is being done to prevent antimicrobial resistance? I mean for instance in this hospital?

**Respondent:** I do not know exactly what is being done but I do not think it is sufficient. For example antimicrobial stewardship service, I heard it on rumors but still it is not implemented in this big hospital. Second, the ***role of clinical pharmacy in this hospital is limited***: drug selection, antibiotic dose adjustment, changing IV antibiotics to PO. These are not well handled in this hospital. We are also using the old drugs. There are things being done in infection prevention. However these are unit and individual efforts. ***Team work is very limited or low.*** Even there is some change it is not clear and visible on the ground

**Interviewer:** What possible measures do you recommend for better antimicrobial resistance containment practices to be implemented in this facility? Please relate your recommendations based on its importance.

**Respondent:** They are many. Since this is a tertiary hospital, ***prescription of antimicrobial drugs must be restricted***. Interns, GPs and nurses must not prescribe antimicrobial drugs but R2 residents and senior physicians can prescribe antimicrobial drugs. ***Training should be given*** about rational use of antimicrobial drugs for health care professionals irrespective of specialty. ***Patient education training*** should be given for patients about antimicrobial use. The ***hospital leadership should design a policy regarding antimicrobial use.*** If possible for ***common pathogens, better for the hospital to have a treatment guideline***. Again ***antibiogram***, common pathogens with their susceptibility pattern should be prepared. ***Clinical pharmacists should part of the team (pharmaceutical care)*** at every unit in the hospital like in internal medicine, surgery. ***Finally antimicrobial stewardship should be implemented in this hospital.***

**Interviewer:** Which one do you prefer to implemented in this hospital: restriction or prospective audit and feedback intervention [the interviewer provide brief description about restriction policy and prospective audit and feedback intervention].

**Respondent: *I think both are equally important*** specialty in big hospitals since the cases are complicated infections. ***Restriction is very good particularly for the newer antibiotics***. Thus restriction is very important. As you said prospective audit and feedback also has its own advantage for patients.

**Interviewer:** What do you think are the barriers that might influence the implementation of restriction; and prospective audit and feedback in this institution?

**Respondent: *Enabling factors:*** It is very important. First, what we can do is, there are some ***trained ID physicians in this hospital***. In each department R3 and above should do it. Another, it should be done in consultation with senior staffs like microbiologists. B/c I believe the required human resources ***[although few, we have well trained ID physicians and trained microbiologist which is an enabling factor].***

***As to barriers,*** I think ***attitude is one, there will be resistance***. Since this work is done by limited senior staffs, the senior physicians should stay for longer period of times. Since the number of ***physicians is limited*** and the ***hospital is very big***. The burden will be on some physicians. ***Also the level of training of pharmacists may not be sufficient.***

We should separate those patients with contagious infection, there is a question how the hospital is ready to do this ***[ inadequate space or resource for isolation].*** The ***selected physicians need evidence based work*** and ***resource is very critical*** here like drugs but the ***microbiology is not strong*** to do this. These physicians should get better ***incentives and they should in turn stay in the hospital for longer time [availability might be a problem, so we have to devise mechanisms like use of on-call to alleviate this problem].***

**Interviewer:**  How about barriers for restriction?

**Respondent:** In our country we can start restriction but not apply it completely b/c antibiotics are being prescribed here and there. For example if one physician thinks to assesses a patient; and find infection or suspected risk of developing infection; the physician may prescribe antibiotic but at that time if the ***physician is not there when needed and these leads delay of treatment*** and these can create adherence problem. The ***patient can also complain for the delayed treatment.***

**Interviewer:** Do you think that there will be a question of autonomy?

**Respondent:** As we said, in order to decrease such things, ***institutional policy on how to work together must be designed, for example, what is the role of physicians, ID physician, microbiologist, pharmacist or clinical pharmacist; if it is clearly stated there will not a problem.*** ***Agreement is important.*** They should consult before prescription especially for higher antibiotics they should communicate one another. ***Obviously if there is no agreement and communication a question of autonomy will raise. So, we have to convivence them that the team is there to health them provide better service but otherwise they may say who is he?***

**Interviewer:** That is the end of my interview, do you have additional ideas to share with me?

**Respondent:** I think I also know other team working in quite similar quality improvement topic. So I advise you to work collaboratively.

**Interviewer:** Ok, thank you.

=================================================================================

Pharmacist **KI#07**

Age: 33 years

Sex: Male

Department: Pharmacist [Clinical Pharmacy]

Staff Position: Lecturer and Clinical Pharmacy Preceptor

Total work experience: 10 years

Number of patients treated prescription per day: N/A

Number of patients with ABX prescription per day: N/A

Total Interview time (duration): 32:28 Minutes

**Interviewer:** Ok , to start with my first question, how do you describe the current use of antibiotics in Ethiopia and particularly in this institution?

**Respondent:** It is ***difficult to say that there is appropriate use of antibiotics, especially in our setup.*** Of course, there might be so many factors. These ***factors could*** be ***availability of drugs***, professional recommendations and set-up facility related problem. If we see in tertiary level, availability is better but in lower health facilities availability is not that much. Thus antimicrobial use will be compromised. These could be ***due to under treatment*** and sometimes when you go to ***private institutions; it could be due to abuse of antibiotics***. The problem varies from setup to setup. But when it comes to tertiary level facility, it would be important if treatments are given based on ***culture reports for critical patients and empiric treatment is very high.*** When empiric treatment is given, it leads to use the ***higher level antibiotics and this in turn leads to resistance***. There is a ***facility problem for not giving culture based treatment for patients***. If the facility is well equipped and ***guidelines are prepared [no guideline]***, the professionals could be enforced. Anyways it is difficult to decide. ***In general the practice of using antibiotics is not evidence based***. ***As to my observation, the practice is not adequate even it needs more attention.***

**Interviewer:** Are there factors related with patients (cost, adherence)?

**Respondent:** When we see adherence, adherence of admitted patients is the responsibility of the professionals who are following the patient. ***There is an attitude in the community even in the professionals that high cost drugs are more effective than low cost drugs. The private institutions usually tend to prescribe costly and broad spectrum antibiotics.***

**Interviewer:** How do you describe patients’ pressure to prescribe or not to prescribe antibiotics?

**Respondent:** My experience is from hospital and such scenario is not common, patients take whatever medicine is prescribed. I did not see patients pushing for antibiotic prescription; patients may request more effective or better drugs, for instance, if he/she took the drug before they may say that they have already took it and they wanted to be changed.

**Interviewer:** How do you see the use or preference of IV and PO? Why do you think so?

**Respondent: *Such things or preference are performed by professionals. Rather patients may request for less cost drugs if they cannot afford, cost matters most. If the IV drug is expensive they request for PO which is less expensive.*** Most of the time, IV to PO conversion is done by professionals. Most of the time patients ask from cost and efficacy angle.

**Interviewer:** How do you see the current status of antimicrobial resistance?

**Respondent:** Although most studies are cross-sectional, studies indicated that AMR is very high. So, it is very serious and is increasing highly.

**Interviewer:** Which types of pathogens/infections do you think have more resistance profile?

**Respondent:** When we see from gram positive, even though I do have minimal exposure to microbiology, ***pseudomonas infection, staph aureus and MRSA are common.***

**Interviewer:** What factors do you think are contributing to increasing emergence and spread of antimicrobial resistance?

**Respondent:** Patients who are taking antibiotics are increasing. More antibiotic are being prescribed. Rate of switching prescription of antibiotics is very high and frequent which is responsible for increasing resistance. Another major factor is poor infection prevention practice. If there is no infection or if we can prevent infection we can minimize, it is possible to minimize antibiotics use. Of course it contributes a lot

**Interviewer:** How about the knowledge and skill of staff, does it have a contribution for resistance of antibiotics?

**Respondent:** As to my understanding, lack of knowledge, one health professional does not only live by the knowledge he/she get from school, he/she must always update himself/herself. This could be fulfilled by training like CPD, preparing workshops and by updating when there is an update. These are very important. ***And team work is a big thing and has immense contribution in filling gaps.***

**Interviewer:** Tell us what you know about the relation of physicians (attitude) to that of laboratory?

**Respondent:** I do not hear much about sensitivity and culture test. But there is a complaint about other laboratories; b/c unlikely findings are reported. But I did not hear such complaints about culture and sensitivity.

**Interviewer:** As you said resistance is a very serious issue; how do you see the containment strategy being done so far?

**Respondent:** In fact I do not have the comprehensive information and I did not see things implemented practically. It is not satisfactory.

**Interviewer:** What do you suggest to tackle AMR?

**Respondent:** I think if the stewardship program is better to be implemented b/c it is a common practice in part of the world and it would be a big thing for us. But I do not know how long it will take to be implemented. ***Until then habit of working together should be encouraged with the physician, microbiology, pharmacy and nurse even in seminars and workshops***. All concerned professionals should be involved in infectious disease.

**Interviewer:** If an infectious disease physician lead antimicrobial stewardship program including microbiologist, pharmacist is formed; do you think this will be applicable in this hospital? How do you see?

**Respondent:** The difference is; ***first, I think, we should do more on attitude b/c every professional has his/her own self esteem the same to others.*** We have to know that we have a common goal i.e. the serve the patient. Respecting and appreciating the role of others. Being ready to fill the gap left by others. It is possible to implement. ***This could be done and we can bring many changes b/c [enabling factor]; one- there is an educated community[there is human resource], two-above all there is knowledge.***

**Interviewer**: What are the favorable conditions you think to implement the antimicrobial stewardship program?

**Respondent:** Yes, there are. Education use is wider here than other setup. There are sufficient antibiotics in this hospital or better availability of antibiotics. It is possible to determine the level of their resistances which once are working and which once are not. ***We have the required human resource or professional.***

**Interviewer:** What do you think about restriction of antibiotics?

**Respondent:** As to the information I have prescription is restricted in developed countries but they restrict b/c they have information or evidence. When it is restricted, individual therapies are seen separately, they may deviate from what is written and this is clearly stated with criteria when and how to deviate; this all is done on the evidence they have at hand.

In Tikur Anbessa hospital the following ***barriers*** should be addressed; the ***common pathogens should be listed, resistant antibiotics should be known***, these antibiotics are sensitive to this infection but resistance to this infection should be addressed. Thus, we should have resistance profile. If not, it may affect the medication. If we do not have the correct data, patient will not improve and the complaint comes from the patient. This could be an obstacle. ***Restriction should be implemented after the baseline data is accomplished. If restriction is not based on data, there will be resistance from prescribers.*** Also, ***unavailability of professionals could be a barrier*** but we can devise a system to solve the problem.

**Interviewer:** One question I did not ask you regarding team work, how do you see it from time and compensation point of view?

**Interviewer:** That is the end of my interview, do you have additional ideas to share with me?

**Respondent:** I would say the culture of working in team should begin at the undergraduate level like during attachments. There should be a system to came up with the same plan in providing the services and that should be started at educational level, not only after graduation.

**Interviewer:** Ok, thank you.

=================================================================================

Pharmacist **KI#08**

**Interviewer:** Let’s start our interview by the hospital and your experience antibacterial how to use antibacterial and antibiotic medicine. how do you explain it normally?

**Respondent:**  I have 4 years experience in this hospital. I have worked 2 years in emergency and also ICU in this year. The antibiotic use doesn’t have guide line. One senior in emergency or other place may prescribe by himself, after that the others resident or intern will follow through. Second, they use the antibiotic, which is already there. I mean if the patient needs Meropenem if it is not available they use other antibiotic medication what the hospital has like Ceftriaxone. They see the cost when I say cost it means the patient affordability. If the patient can’t afford vancomycin even if it is needed there are times they prescribe another antibiotic. Other thing is in the emergency room when the patient comes in the room there is no culture done. When the patient comes to the emergency room, the physician just puts him on Ceftriaxone, this is common.so, In emergency room they use broad spectrum antibiotic. Broad spectrum is given to the emergency, the culture result should be done to change it to narrow spectrum. However, once they don’t change their prescription.

**Interviewer:** so, do you think that when you see these things they are rationally antibiotic use?

**Respondent:**  No, I don’t believe that there is rational antibiotic use because first if the duration of treatment is needed for 7 days but if the patient stays for 20 days for that day they continue using that medicine. The other is if a patient gets in emergency room and prescribed Ceftriaxone for 7 days and if he gets discharged and return back after a weak they make him start Ceftriaxone again because of lack of documentation.

**Interviewer:** so, there are some who wants to changed ROA from oral to IV?

**Respondent:**  they think they recover fast with IV

**Interviewer:** do you think there is impact on physicians on the prescription or do they pressure them from the experience?

**Respondent:**  from experience when the patient asks for IV antibiotics the physicians don’t give the antibiotic, but rather they give them anti pain like tramadol to satisfy the patient or there is what you call placebo.

**Interviewer:** you told me the antibiotic use but how do you see the antibiotic resistance use in this hospital?

**Respondent:**  In our hospital I can’t say study has been done but last time I faced one patient in ICU that has developed resistance to multiple antibiotics. After that culture has been done and at that time Meropenem wasn’t available. From the Outside market the medication costs 1000 birr per vial and at that time the patient couldn’t afford it.

**Interviewer:** what did the patient do?

**Respondent:**  that patient expire

**Interviewer**: I think expire word is common here

**Respondent:**  yes

**Interviewer:** so, you think there is impact if it can be focused there will be resistance

**Respondent:**  yes

**Interviewer:** which medication developed resistance?

**Respondent:**  Ceftriaxone is the most used as first line but after a while we see that is changed from Ceftriaxone to ceftazidime. Both of them are 3^rd^ generation Cephalosporin. The other

There is a big problem on time duration. The senior has to order for discontinuation of antibiotics otherwise the resident won’t even if the patient has improved.

**Interviewer:** what is the reason for the increase of resistance?

**Respondent:**  1^st^ there is no proper documentation of the patient medical record. It should be recorded and reviewed even in hospital. 2^nd^ allergy of the patient is not stated or known. He might be allergy for certain antibiotic. The other thing is absence of guide line for the ICU patient, for emergency patient… on duration of treatment.

**Interviewer:** so, I can say the mentioned causes of inappropriate prescribing?

**Respondent:**  Yes, also available of drug in PO form influence the change of RoA from IV to PO whenever appropriate.

**Interviewer:** can the infection prevention influence with this one?

**Respondent:** yes, by giving training, If the clinical pharmacists can involve. You remember we tried before me and … was doing a good job in the emergency but there was no documentation we gave suggestions day after day. But we never saw any change that on the things we recommended. Because we are human beings you get tired. So, now because of this I don’t have the interest to work on in clinical pharmacy anymore b/c we tried 2 years in emergency. It is always what they decide.

**Interviewer:** I take this as the lack of documentation. I am not going to ask you about microbiology you might not have information on that.do you think what is being done is enough minimize antibiotics resistance development?

**Respondent:**  I don’t know what the hospital thinks. Last time meropenem wasn’t available then it came but just a little amount. The ICU director was here doctor Dawit and I talked to him b/c as soon as Meropenem came all the doctors prescribed it to their patients but was not enough. Instead of prescribing to every patient I suggest to be given for those patients that are sensitive and have no other alternatives. He didn’t accept my suggestion and said the physician can prescribe it whenever they see fit prescribed.

**Interviewer:** what I take is no team work and I will talk to him later.do you think antibiotic restriction can be implemented? Can policies be implemented?

**Respondent:**  if the superior power or government order it, it would be applicable

**Interviewer:** what do you think the challenges will be?

**Respondent:**  the staff might resist. because of the hierarchy. i think the government can enforce to make it implemented by research.

**Interviewer:** what if the higher be must be consulted at all times.

**Respondent:**  for example, when linezolid is prescribed the pharmacists should see a signature before dispensing

**Interviewer:** you know what they do in other places if it is said let it be prescribed by seniors their signature has to be in it.so, the pharmacy will receive the seniors sign. if that person is not there by some emergency you will call so you have to make sure he is a senior. How did you decided did you talked with the mangers?

**Respondent:**  I got the information through my boss.my boss is case leader, he gets the order from pharmacy director. I don’t know whether the pharmacy director talks with the manager or not. If one doctor came to take linezolid I will just dispense it because I don’t know who the senior is. They just say it’s senior consulted, that means not specifying.

**Interviewer:** do you think it is possible to implement restriction? Or should it be applied?

**Respondent:**  ya I think it will be successful if it is applied. Especially physician who specialized in infection is consulted. The one who specialized in gynecology doesn’t know about infection. If you see, the surgeons they forget about the medication if he knows Ceftriaxone he might just always prescribe that. They might not have the knowledge of infectious doctor. That is one challenge. The senior has to be who specialize infectious should be consulted. Like the oncologist knows about the oncology medicine he might not know about antibiotics. The seniors will be better if they specialize in infectious decease. they have multidepartment so they can mention it there. If they communicate well, this will increase its successful rate.

**Interviewer:** what if the doctor and the pharmacist audit it together and work on the the feedback do you think it will be successful?

**Respondent:**  The team members that are involved have to be well educated. ID physician is already trained but we (pharmacist) weren’t getting well trained at that time. It is necessary to be trained in infectious disease. If it is said the ID physician and the pharmacist should train to make the decision then it should. The pharmacists should be trained well. If not the pharmacist level of knowledge will make limit. Now we gave up because we are not working on clinical pharmacist as before we are losing what we have.

**Interviewer:** what might be the other barrier?

**Respondent:**  as long as the pharmacy and the doctors work together there won’t be much problem. But still way of giving feedback is an issue.

**Interviewer:** which method do you think is easy to give the feedback from your experience.

**Respondent:**  as I see from this place most them are students even if they are resident 1 or 2. They fear the seniors. Even the senior is mistaken they won’t say anything because they consider them selves as a student.

**Interviewer:** they might scared legal issue if it’s written

**Respondent:**  as a solution in written but not in-patient card by making its own form and the pharmacist and the ID physician write the feedback together and give it to the senior.

**Interviewer:** you are saying if it is not included in patient card?

**Respondent:**  yes

**Interviewer:** do you have other barriers?

**Respondent:**  no

**Interviewer:** what can you say about the facility, finance or other issues to implement?

**Respondent:**  finance is needed from the higher up like the government. Management of time and finance is required.

**Interviewer:** the doctor might ask other things. from the pharmacy view shortage of medicine because when you tell him to convert from browed to narrow. Is there any problem which is related to pharmacy?

**Respondent:**  now is 90% better supply of antibiotics than before. That the availability is increasing.

**Interviewer:** do you have anything to add

**Respondent:**  As I heard it from you it is not only about the pharmacy and also include the doctors. If the nurses also included they are who need trained because the doctors order Cefazoline then they write Ceftazidime so we will give what is written. As we said broad spectrum antibiotics can’t be used for many times. The empirical treatment after culture tis done so it should be change to narrow spectrum.

**Interviewer: Thank you.**

=================================================================================

## Pharmacist KI#09

Age: 28 years

Sex: Male

Department: Pharmacist [Clinical Pharmacy]

Staff Position: Ward Pharmacist

Total work experience: 4 years

Number of patients treated prescription per day: 50

Number of patients with ABX prescription per day: 45

Total Interview time (duration): 37:18 Minutes

**Interviewer:** Ok thank you. We will talk about three major issues: your perception of antibiotic use; resistances and the containment strategies. To start with my first question, how do you describe the current use of antibiotics in Ethiopia and particularly in this institution?

**Respondent:** In my four years experience at this hospital and as a dispenser in community pharmacy, there is ***problem of antibiotic use from professionals and the public. People are using them as antipain medicines and I have experienced people asking for antibiotics for treatment of headache or other pains in the leg or throat.*** In my believe, first it might be due to ***lack of knowledge or awareness about antibiotic usage***; other thing is the health delivery system of our country i.e. patient go to health facilities are now ***unnecessary exposed to expenses like for excess/unnecessary laboratory investigations***. So, the public don’t want to visit the health facilities rather they opt to purchase antibiotics without prescription. Moreover, ***the use of antibiotics from health professionals perspective is inappropriate; you antibiotics right and left antibiotics prescribed.*** Another thing is if they go to health institution, physicians prescribe expensive and unaffordable medicines. ***In my view, the public in general and health professionals do have poor knowledge and unfavorable attitude towards antibiotic usage.*** Even health professionals ***do not use standards*** of hospital for prescribing antibiotic rather they use what they want to prescribe.

**Interviewer:** So do you think prescribers tend to over prescribe antibiotics or there is over use of antibiotics?

**Respondent:** Yes, definitely.

**Interviewer:** What about the use of broad-spectrum antibiotics as compared to narrow ones? Do prescribers tend to prescribe very broad-spectrum antibiotics empirically?

**Respondent:** The use of broad-spectrum antibiotics is common. This mainly due to inadequate knowledge of the professionals and the poor infrastructure of the diagnostic facility. They don’t have a diagnostic facility to correctly identify the exact etiology or infection. On the other hand, relatively is better in the country but the level of utilization of the already available set up in TASH is very low rather they simply prescribe whatever they want, mostly they want to prescribe the broad spectrum to get wider coverage. For instance, ***when client comes to health facility and say redness in my skin, then health professionals simply say most probably the cause of skin infection is Staphylococcus aureus bacteremia and they prescribe Cloxacillin to all. They do have the culture to request for investigation to differentiate etiology rather they want to prescribe broad- spectrum for any infection.***

**Interviewer:** You were telling me that public’s poor awareness/perception towards antibiotic use, weak diagnostic facility, poor knowledge and awareness of health professionals are contributing for the inappropriate use of antibiotics. Can you tell me other contributing factors?

**Respondent:** As I told you, internationally there is health policy like drug usage policy and other policy but in our country how much it is enforced/applied about antibiotic usage. I see these in two perspectives, the first one is antibiotics can be obtained purchased without antibiotics like another good you are goods from supermarket, so you can patients can easily get antibiotics. Second, how much are willing to work in team. There is no collaborative services. Customer are not satisfied by services of the health facility and they want to go to community pharmacy to get the targeted/specific drug for their problem. If they get improved by taking this antibiotic that motivated them to continue to use antibiotics from the community pharmacy. They don’t want to go to health facility because they are asked to pay extra price in health facility. ***There is no strong counseling of patients to create awareness for antibiotic usage rather they tend to prescribe expensive drugs like Augmentin for simple cases like tonsillitis or unnecessarily for common cold, so that over usage of antibiotic started from childhood and that affects in life long.***

**Interviewer:** How do you describe patients’ pressure to prescribe or not to prescribe antibiotics?

**Respondent:** I do not believe patients have an influence in antibiotic use. Probably from their previous experience in other hospitals some patients might need specific routes or medicines but I don’t see their influence in prescribing but rather I see patients fully believe what they are said. However, this might be true in private clinics where they might influence physicians.

**Interviewer:** How about their preference of PO and IV?

**Respondent: M**ost patients usually prefer injectable drugs rather than P.O drug because they believe injectable drug have rapid and better effect than PO drugs. On the contrary, there few patients we want PO rather than injectable drug due to fear of more side effect and believe PO are better. ***But anyways majority choose injectable drug and this might be related from the effect they see from antipains which is an immediate effect and they believe all medicines give better effect if given in injection.*** Due to that they simply want injectable drug for their problem. ***These attitudes come with anti-pain drug like diclofenac which give them immediate relief but most clients do not know the drug is diclofenac or antibiotic or other drug. When client comes to our dispensing pharmacy if prescribed drug is P.O, they say is this drug mine, do you have injectable drug***

**Interviewer:** As you told me now, overuse of antibiotic and broad spectrum use of antibiotic is common in your hospital, so that what is your insight of antimicrobial resistance or how do you describe the current status of antimicrobial resistance (at institutional and national level)? Do you think it is a growing public health treat or not?

**Respondent:** In my 4 years’ experience, mostly prescribed drugs are vancomycin and ceftazidime for injection, and these were most of the time stocked-out. Also, most commonly used antibiotic in our hospital was ceftriaxone and it is given for long time or duration in admission room as long as the patient stayed in the hospital. ***After ceftriaxone usage for longer time physician now change to most broad spectrum like meropenem, pep/tazo and cefepime without laboratory investigation or culture results..*** Most clients come to our hospital after start of ceftriaxone in ICU and other department. These show that most of cases are resistant or not responding for other antibiotics because drug prescribed without any laboratory investigation for antibiotic resistance. Most patients don not improved in our hospital after given of antibiotic so they have poor progression or high treatment failure which indicates there is high level of drug resistance in our hospital.

**Interviewer:** So do think antimicrobial resistance is increasing, stable or decreasing over time?

**Respondent:** I think antibiotic resistance is increasing over time. There is no data but my justification to this is: we see a rapid switch of antibiotics mostly to broad antibiotics, longer hospitalization and high hospital mortality. These all show an increase in prevalence antibiotic resistance over time. In my perspective antibiotic resistance is increasing day to day

**Interviewer:** Which types of pathogens/infections do you think have more resistance profile?

**Respondent: Methicillin resistance Staph aureus (MRSA),** Streptococcus **aureus, and** MDR-TB are common ones. even resistance to for fluconazole and now to Amphotericin B are increasing.

**Interviewer:** Do you face any challenge in your daily practice as a result of antimicrobial resistance? How does it affect you and the patient?

**Respondent: *It is increase antibiotic consumption, inappropriate use antibiotics result in frequent stock-out of antibiotics (shortage) which in turn would increase resistance and HAI.***

**Interviewer: *It is vicious circle type***. You were telling me that OTC sale of antibiotics as a factor to increase AMR but what other factors do you think are contributing to increasing antimicrobial resistance?

**Respondent:** One is the ***poor infrastructure of microbiology*** that make them not to ***correctly identify the etiology*** of the infection and ***prescribe tend to broad spectrum antibiotics***. The **easily prescribing policy** of antibiotics [any one can prescribe any antibiotics and there is no restriction], ***absence of strong follow up*** on the prescribed antibiotics, ***absence of IV to oral conversion policy*** and ***frequent stock-out*** of antibiotics that forced to prescribe other antibiotics are also contributing for the problem. Physician do not divert patient from IV antibiotic to P.O drug after some improvement rather they stay in IV and there is poor communication between nurse and physician. There is no follow up by progress of case after given of one antibiotics and not assess w/t is the cause of progress rather they change antibiotic without laboratory investigation As I mentioned it earlier interruption or frequent stock-out of antibiotics that forced to prescribe other antibiotics, poor patient adherence are the major cause of antibiotic resistance. Another thing, clients comes to our hospital after acquire of resistance (over all community dispensing of antibiotic)

**Interviewer:** What possible measures do you recommend for better antimicrobial resistance containment practices to be implemented in this facility?), how do you understand team work to control infection in hospital

**Respondent:** There is infection prevention team in hospital but it is not strong, it is weak. There is no strong networking among professionals for instance I don’t if pharmacist I involved in that so it is not strong and inclusive of all departments. They don’t ask or discuss about IPP with pharmacy department**.** Also ever health professionals like nurse, laboratory and doctors come to our dispensing unit with glove and touch everything then patients touch it, supporters touch it. So that it simply contaminate all over. This is due to no poor IPP and there is no controlling mechanism.

**Interviewer:** As you say there is high rate of drug resistance, from your experience do you believe that the way of controlling drug resistance is strong in your hospital. How do you describe it?

**Respondent:** I don’t believe it is. It is not strong enough. To control resistance one we have to monitor or restrict over the counter sales of antibiotics, second decrease rate prescribing of broad spectrum antibiotics, improve awareness of community about antibiotic resistance and usage, improving laboratory set up to differentiate etiology before prescribing antibiotics, increase availability of antibiotics because interruption of antibiotics can cause resistance.

**Interviewer:** You were telling me about restriction policy [the interviewer briefed how restriction policy works], how would you feel if it is applied in this institution? Do you think it is feasible and important? What are the barriers and enablers for this intervention?

**Respondent:** ***Definitely, there is no question about its importance or benefit and it can work*** but there is also challenge to implement. **For instance, in our hospital Narcotic and Psychotropic Substances are restricted via special prescription paper but the prescription by itself is no monitored…everyone can access the prescription including nurses.** Challenge is who prescribe it, by which it controlled, nurse, physicians. Because physician were not all time in working place, When all Professionals prescribe all kind of antibiotic it become resist for most drug. If you apply restriction, they may not stop prescribing it, they may find prescription from secreted place and can prescribe it. Using color prescription for narcotic drug is for specification or for restriction one best way and others use giving their own sign in dispensing unit and differentiate who prescribe the restricted drug but they can put fake similar signature.

**Interviewer:** So can you think of potential factors that might influence the implementation of restriction?

**Respondent:** Prescriber ***autonomy*** might be a question but I think we have to do implement it as far as it benefits the patient. The patient should be at the center of our care. Also another problem could be the ***interruption of services if the authorizing physicians are not there.*** The ***inadequacy of health professional*** is also a challenge.

**Interviewer:** What other enabling factors do we have?

**Respondent:** These fact that it is important to the patient will be accepted by most practitioners.

**Interviewer:** Another type of antimicrobial stewardship intervention is prospective audit and feedback [the interviewer briefed how it works]. So, How would you feel if prospective audit and feedback is applied in this institution? What are the barriers and enablers for this intervention?

**Respondent:** This issue is very important because we know that ***provision of health services in not one individual or profession responsibility, it is team work.*** But in this hospital everything is decided by one person and others follow or observe his/her decision. There is ***no culture of treating in team*** but I think it is a good idea Not only for this kind of problem but also all health care decision should be in team. The challenge; physicians think they are superior and they may not be ready to accept pharmacist and other junior professional’s suggestion. So there might be resistance. Another barriers is inadequacy of professionals. In laboratory aspect, laboratory professional’s number is low and there is high work load in laboratory, shortage of supply in lab is another problem (reagent, medical problem, no calibration of med equipment) and there in no culture of working team. knowledge gap is not a problem for this problem.

**Interviewer:** What enabling factors do we have?

**Respondent:** If you convince the impact of the problem and the benefit of the intervention, I am sure the ***hospital management are willing to support the intervention.***

**Interviewer:** That is the end of my interview, do you have additional ideas to share with me?

**Respondent:** I appreciate you because you done the most pressing issue (antibiotic usage…) and others see it as simple issue. As to my understanding it is the best scientific investigation because we see antibiotic resistance is increasing morbidity and mortality. Antibiotics use is increasing and many people are taking antibiotics without any reason and we see resistance increasing after short time. So this study is very important for our hospital and nationally.

**Interviewer:** Thank you for your insight information and your time.

=================================================================================

## Pharmacist KI#10

Age: 27 years

Sex: Male

Department: Pharmacist [Ward Pharmacy]

Staff Position: Pharmacist

Total work experience: 4 years

Number of patients treated per day: 20

Number of patients with ABX prescription per day: 15

Total Interview time (duration): 28:08 Minutes

**Interviewer:** Thank you. In general I would like to know the overall use of antibiotics, how do you describe the current use of antibiotics in this institution or at country level?

**Respondent:** To some extent the usage of antibiotic is proper but almost all patients who come to our hospital especially inpatient usually took at least antibiotics. I think, after admitted in inpatient no one is discharged without taking at least one antibiotic. By one or another reason, they repeatedly take antibiotics for longer duration but they may not need it or it should have been given for short time. Generally, I think the inappropriate usage of antibiotic is very common. The common problems are: overuse, and patient stay unnecessarily for longer duration in antibiotic. Patients who admitted for long time might be prescribed with antibiotics but don’t know when they started antibiotic and there is no plan to stop antibiotic after starting it. Treatment continues until the senior tell them to discontinue or stop the given antibiotics but if s/he doesn’t ask the patient will continue to take that antibiotic. For instance, ceftriaxone should stop in 7 days but such plan is not available in wards, so you should search or trace the document when was it started or prescribed.

**Interviewer:** How do you compare the use of broad vs. narrow spectrum antibiotics? Do prescribers tend to prescribe very broad-spectrum antibiotics empirically and/or to over prescribe antibiotics?

**Respondent:** ***Initially all physicians use broad spectrum*** because one, our ***Hospital is assumed as if it has highly resistant pathogen.*** So since there is ***no clear data about patients status of antibiotic usage and there is no culture sensitivity test results***, then we ***opted to use broad spectrum antibiotics.*** Due to these issues, during admission they use broad spectrum antibiotic to treat patients by empirically. ***Secondly, prescribing physicians are first year or junior residents and residents also fear the questions of seniors about complication and why s/he didn’t cover the possible pathogen. Hence, they worry for the senior question, fear their response but don’t worry about the risk to the patients’ life***. From pharmacy aspect, there is interruption of drug supply [no continuous supply of antibiotics] in hospital like third generation antibiotic, so we change the prescribed drugs to alternative drug after recommendation, most drugs are broad spectrum. ***Also some drugs, when changed, are costly to the patient [unaffordable] and broad spectrum.***

**Interviewer:** How do you describe patients’ pressure to prescribe or not to prescribe antibiotics? How about their preference of IV and PO antibiotics?

**Respondent:** In my opinion there is no pressure of patients to prescribe, change or not to prescribe antibiotics but most clients want to prescribe IV medicines. ***They even thin or prefer to inject them water for injection instead of PO medicines.*** Patient do not know why drugs are prescribed or for what purpose it, only the want to prescribed injectable (IV OR IM) drug. These is in all clients means it included educated groups too.

**Interviewer:** How do you describe the current status of antimicrobial resistance? Do you think it is a growing public health treat or not? How do you see the pattern of resistance over time?

**Respondent:** For most drugs, resistance of bacteria is increasing through years to years with different reasons are mainly drug supply problems and misuse of antibiotics. Resistance of antibiotic is increasing because consumption (by quantity and type) of antibiotic is increased indicating increase of resistance; again antibiotic effectiveness or response rate is decreasing in many patients or number of failure is increasing; we know some patients especially in ICU who were resistant to all available antibiotics which are only susceptible to Linezolid. Generally there is no clear data to say this patient resistant for this drug and other patient resisted for that drug but from clinical practice we can say resistance is increasing.

**Interviewer:** From your experience which types of pathogens have more resistance profile?

**Respondent:** Staph coccus, and pseudomonas are most common one.

**Interviewer:** What factors do you think are contributing to increasing emergence and spread of antimicrobial resistance?

**Respondent:** ***Most common reasons for AMR are overconsumption of antibiotics, inappropriate use, knowledge gap and wish of physicians, also not prescribing antibiotic by ID physicians [no consultation of ID]; due to this in some department we see they prescribed one kind of antibiotic for all patients, it may not important for that patients. So that physicians have low knowledge of it. Due to supply interruption of antibiotics and we change the drug for three or more time, simply discontinuation of drug because of unavailability of drug like vancomycin discontinued after 3 day intake.***

**Interviewer:** If we need to control the problem of resistance, what measures do you recommend for better antimicrobial resistance containment practices to be implemented in this facility?

**Respondent:** I feel that the efforts to control resistance is still weak. some drugs should be restrict to selected prescribers. ***I think this has been discussed in DTC to restrict some antibiotic to be prescribe only by ID physicians but not applied it. Meropenem, vancomycin and other drugs are taken by most patients but if we restrict it there will not be resistance.*** Other thing is infection prevention activities of our hospital. Infection prevention of our hospital is not good or it is poor. For instance in ICU room, everybody can enter [no movement restriction]. ***So, I suggest to improve infection control mechanism, restrict selected antibiotics and the supply of antibiotics.***

**Interviewer:** How would you feel if restriction [the interviewer gave detail description how restriction policy works] is s applied in this institution? Do you think it will be accepted?

**Respondent:** It needs time to accept restriction but yes it can be implemented. E.g. one time we [pharmacist] recommend to prescribe meropenem by restriction due to inadequate supply by that time but even the seniors refused to accept pharmacists recommendation, his response was pathetic and said that it should be the decision of the physicians. ***They need instruction from managers to accept it.*** Due to this, there may be negative response in physicians. But it is possible when higher official or hospital management instruct it to be done as a policy or approved by the DTC.

**Interviewer:** What barriers and enabling factors should we anticipate for this intervention?

**Respondent:** Barriers to implement restriction are: resistant from physicians, inadequate numbers of ID physicians and inadequate time to audit b/c they may not cover all patients due to their number ***[difficult to audit all patients unless targeted],*** so adding other seniors selectively could be the solution. *The enabling factors to apply restriction is patient improving from their cases and the duration of patients stay in our hospital is long which can convince everyone. So minimizing patients stay in hospital is one enabling factor b/c our* ***ultimate target is to improve patient outcome which satisfy all staffs and the patients.*** *The main* ***enabling factor is magnitude of problems which is high*** *and you can got attention from the management and others.*

**Interviewer:** How would you feel if prospective audit [the interviewer gave detail description how prospective audit and feedback works] is s applied in this institution? What are the barriers?

**Respondent:** Prospective Audit is important and we agree on that. I don’t think there is a professional who don’t like feedback. ***But some physicians may not want to show them that they had wrong doings [changing what they have prescribe would mean to saying them what they prescribed was not right or correct].*** As barriers most seniors are not available in hospital rather resident students are avail in hospital. More challenge is finding seniors to give feedback. So the team members should include seniors but finding seniors is a challenge b/c they may not have adequate time, inadequate ID physicians. The other is, parameter which is used during admission patients like ***test/culture*** are done before prescribing antibiotics and this should be putted as a mandate. ***So availing data to prescribe antibiotic and applying or using that data for drug prescription is a barrier.*** Also availing policy for prospective auditing and feedback. Before starting this, first availing all drug and reagents to recommend during feedback but otherwise it will be difficult to recommend unavailable medicines although it could be the best choice.. Another thing is ***knowledge gap of some team members is an issue and seniority of professionals should be considered while establishing the team.***

**Interviewer:** What do you think the enabling factors for prospective audit intervention?

**Respondent:** The enabling factors are: the intervention can improving patient’s outcome which will increase staff stratification and acceptance, increase team work of pharmacist with physicians. ***The decrease in cost of patients and hospital b/c use of numbers of antibiotics can be decreased and thus cost is decrease, so the hospital budget can be shifted to purchase and avail other essential antibiotics.*** Also it can help us to increasing efficiency and effectiveness of our work that ultimately improve patient outcome. The fact ***that pharmacists are interested to in team work is also a good to start.***

**Interviewer:** What is do you suggestion of implementing these intervention, should they be implemented simultaneously or one after the other? Can you explain that?

**Respondent:** The prospective audit should be first implemented. But to that end the pharmacy department should be strengthened first with involvement of school of pharmacy staff. This can be done by assessing the capacity of the hospital pharmacy and should be support accordingly. Some few years back, when clinical pharmacists were working in this hospital, we see practice being changing and physicians were curious by that time. Improving time of taking culture test, preparing parameter to take culture test in emergency room should be considered. Generally in team work there is no pharmacy involvement and I think working in team should be improved. We [pharmacists] are like observers, only nurses and physicians work in team but even they did it in order manner not discussion. So I think we can serve as a bridge for this weak communication and poor team practices.

**Interviewer:** Thank you.

=================================================================================

## Pharmacist KI#11

Age: 32 years

Sex: Male

Department: Pharmacist [Director of Pharmacy]

Staff Position: Director of Pharmacy

Total work experience: 8 years

Number of patients treated prescription per day: 25

Number of patients with ABX prescription per day: 5

Total Interview time (duration): 26:36 Minutes

**Interviewer:** Ok thank you. One is about antibiotic use and to start with that, how do you describe the current use of antibiotics in Ethiopia and particularly in this institution?

**Respondent:** Ok thank you. In general, the usage of antibiotic have different problems. Here, most disease treatment is empirical treatment where they have no trend to differentiate the type of bacterial pathogen, and there no infrastructure to differentiate bacteria too. ***In addition to that, physician do not have the habit of asking the client about history of antibiotic usage before prescribing another antibiotic. Even at times there is a possibility where they prescribed the same antibiotic that the patient took it prior they came to hospital.*** Nationally, in pharmacy side everyone can simply obtain antibiotic from any dispensing unit without prescription. Also from the public side, they not finish the given antibiotic probably because dispensing pharmacists may not give them proper/full information for clients so those clients may discontinue the drugs after two days. From the regulatory part, there is no system to enforce policy for their use or there is no policy for antibiotic usage at national or as institutional level.

**Interviewer:** you were telling me there is poor adherence from the patient. But from professionals, Do you think inappropriate use of antibiotics is a problem? How do you compare the use of broad vs. narrow spectrum antibiotics?

**Respondent:** currently, physicians usually tend to start with broad spectrum antibiotics and they don’t usually stick to standard guideline. I have tried to discuss with some prescribers and t***he reason why they don’t comply to the guideline is that they believe that it doesn’t reflect the reality of the hospital as it is a*** tertiary hospital rather they want to use most broad-spectrum antibiotic which is major problem. But as to my belief, it include the major concerns are included in guideline and if they want preparing threshold is possible to do that. Second problem is, the trend of frequent switch of antibiotics in shorter time mostly to broader antibiotics demanding an immediate effect. At least one antibiotic should be switched after 72 hours but they switched it in 24 hours to broad spectrum ones b/c they wanted to see an immediate effect or cure.

**Interviewer:** How do you describe patients’ pressure to prescribe antibiotics?

**Respondent:** As my believe, in governmental institution there is no influence of clients or patients during prescribing antibiotic because most of clients came from rural area of country and I don’t think they have adequate knowledge to influence the prescribe. but some clients may be give suggestion what they want but in my opinion, physician have full of freedom to prescribing as s/he want to prescribe.

**Interviewer:** How about their preference of IV and oral antibiotics?

**Respondent:** Oral to IV antibiotic change, some patients want to prescribe IV antibiotic instead of oral antibiotic, especially pediatric patients they don’t want to prescribe oral, so that they ask to switch oral to other routine or injectable antibiotic but it is not common because if physician take time they can convince the patient to take PO antibiotic as they simply believe them, rather the physician don’t want to take commitment.

**Interviewer:** How do you describe the current status of antimicrobial resistance How serious is the problem? Do you think it is a growing public health treat or not?

**Respondent:** So to discuss about resistance issue, it needs data related with resistance profile of pathogens but from my personal experience the major fear is I can say most of the clients who came to our hospital are already taking or after exhaustively taking antibiotics. So, most of them became exposed antibiotics and this could increase resistance of antibiotics. Another thing is with resistance is the issue of dosing and duration of antibiotics but it is difficult to give exact reason for cause of resistance because it need concreted study. ***From my experience, most of clients checked in this year for resistance of antibiotic and their result is positive for most antibiotics and we had no the last choice of antibiotics, so we discuss with other hospital to find drug (like kanamycin from Petros Hosp) for these patients. So resistance is very common now.***

**Interviewer:** Which types of pathogens/infections do you think have more resistance profile?

**Respondent:** Most commonest pathogens are *streptococcus pneumoniae* and *H. influenza.*

**Interviewer:** you were telling me inappropriate use of antibiotic is contributing for resistance, can you explain that for me. What other factors do you think are contributing to increasing antimicrobial resistance?

**Respondent:** When are talking about resistance there are different reason for increasing resistance of antibiotics. First is the wrong use of antibiotics by patient [poor adherence], extensive use of antibiotics, incorrect usage of antibiotics, incorrect prescribing practice antibiotics and also extensive use of antibiotics for veterinary purposes which means we use animals and their product for d/t reason. These animals find antibiotics by different way and this can be a reason for increasing resistance.

**Interviewer:** What other factors do you are there? Like infection prevention and availability of antibiotics?

**Respondent:** Again poor infection control practice and lack of supplies to perform good infection prevention is a reason. Another is, knowledge of physician and pharmacist aspect, In our hospital there is poor ways of infection control because there is no enough antiseptic solution in our hospital for instance hand washing materials are not available But if anti-septic solution is available practical usage is not problem.

Yes there is frequent shortage of antibiotics but this year availability is better than before but new broad spectrums antibiotics supply had interruptions. But if there is proper usage of antibiotic, availability would not be a major problem as we can project from the true consumption***. To me the major issue is working in prescribing practice and if we improve the prescribing practice, drug supply will not major problem. Improper usage of antibiotics was unpredictably increasing consumption of antibiotics so if this is improved then I think supply is not major problem.***

**Interviewer:** Do you think the hospital has worked enough to control, the problem of antimicrobial resistance.

**Respondent:** The effort of the hospital or particularly the pharmacy is not strong. I personally believe the present effort is almost null and we have to do more in that.

**Interviewer:** What do you think should be done to solve the problem of AMR?

**Respondent:** In these point as my opinion, as intervention, as institution developed antibiotic use policy should be available and not only availability of policy but the regulation (policy) should be enforced, and everybody should stick to this policy or will be accountable for breaking the policy. Policy should be there to restrict antibiotic prescribing and physicians should stick to that agreed guideline. Policy includes new broad spectrum antibiotics prescribed with consultant authorization. Especially those potent, broad spectrum should be prescribed by consultants. So in TASH everyone ***from intern up to consultant*** can prescribe any antibiotics but the ***hospital antibiotic policy should be specify/categorize which antibiotics should be prescribed by whom [restriction at different level is very important].*** Another things is, pharmacist should give full information for patients who are taking antibiotics because pharmacist can influence the usage of antibiotics by giving adequate information. From clients perspective they should use antibiotics appropriately as per the provided information. At national level, creating awareness about antibiotics usage and resistance is the main responsibility of each media because antibiotic problem is not only national but also international issue. Nationally antibiotics usage policy should implement in all over country because only TASH antibiotic policy cannot improve the whole problem of antibiotic usage but I think the BLH antibiotic policy can be used as a baseline for nationally policy.

**Interviewer:** You were telling me about restriction [interviewer briefed how restriction policy works], how do you see if restriction policy is implemented in this setting? Would it feasible and useful?

**Respondent:** In our hospital (BLH), we only have two adult and one pediatric ID Specialist, so at least consulting them by phone is possible hence it would be feasible if we devise mechanism to smoothly implement it. It is also good because the practice of improper use of antibiotics is high, due to that implementing restriction policy is important to minimize those inappropriate uses. ***Few months back, the hospital DTC has decided to restrict selected antibiotics such as meropenem [to be prescribed by senior physicians] but the problem was there was no a system to monitor the progress; no follow-up to document the success and drawbacks of the intervention. So, although its acceptance by physicians was high, the program failed [discontinued] b/c it was not started with proper follow-up mechanisms.*** In DTC meeting we decided to intervene it. Seniors and physician accepted but it falls/ fail due to no follow up and integrated commit.

**Interviewer:** OK, so you think it applicable and useful. But what do you think could be the barriers to implementation of restriction policy?

**Respondent:** In my opinion the major barrier is ***low number of ID specialist***. Others are: ***poor infrastructure of microbiology*** for sensitivity test of antibiotics, ***prescriber negligence*** or lesser attention to the consequence of antibiotics, ***high work load of pharmacist*** that hinder them to provide appropriate counselling and ***clients’ poor awareness on the effect of antibiotics***[they don’t think antibiotics have harm] are other barriers for implementation. Another barriers is ***probably unavailability of authorizing physicians or senior physicians.***

**Interviewer:** What about the enabling factors?

**Respondent:** To apply these enabling factor the availability of ID consultants [although few in number], even most of the culture medias are available. ***Another important thing is the commitment of management to implement such initiatives as a quality improvement tool is another opportunity.***

**Interviewer:** Another type of antimicrobial stewardship intervention is prospective audit and feedback [the interviewer briefed how it works]. So, How would you feel if prospective audit and feedback is applied in this institution? What are the barriers and enablers for this intervention?

**Respondent: *Indeed, prospective audit very important and is definitely applicable if we put well trained and competent clinical pharmacists.*** But with regard to pharmacy professionals, I fear their competency to work at this high level. Hence, they should be competent, must trained well and should be adequate in number. Now in practice, patient oriented pharmacists are working with consultants but I am not sure if they have that capacity, so if we trained them, definitely it can be applicable. Unavailability of senior physicians during the time of auditing might be the challenge too.

**Interviewer:** What enabling factors do we have?

**Respondent:** Enabling factor is ***management’s strong commitment*** to implement such intuitive as they consider it part of the quality improvement. Also, ***strong commitment or need of pharmacists*** to involve in such team work is a good opportunity for its implementation. **The improvement in availability of antibiotics is one good thing.** Currently there is no problem in drug supply management; the supply of medicines is good as compared to previous years and can be considered as enabling. But still there payment problem for supplied drugs. Another challenge for restriction is ***lack of time, unavailability of senior/consultant, pharmacist competence*** but finance will not be a major challenge.

**Interviewer:** If these two interventions are to be implemented, how do you recommend us to do. I mean should they be implemented concurrently or sequentially?

**Respondent:** It would be fantastic if are integrated. If we can integrating both interventions, they should integrated from the start, and I am sure we can get better outcomes.

**Interviewer:** That is the end of my interview, is there anything you would like to add?

**Respondent:** No I don’t have anything, you have address it all.

**Interviewer**: Thank you for your time!!!

=================================================================================

## Pharmacist KI#12

Age: 27 years

Sex: Male

Department: Pharmacist [Drug Information Services, Pharmacy]

Staff Position: DI Pharmacist

Total work experience: 4 years

Number of patients treated prescription per day: 30

Number of patients with ABX prescription per day: 20

Total Interview time (duration): 36:44 Minutes

**Interviewer:** Thank you. I have three major areas for discussion. So to start with my first question, how do you describe the current use of antibiotics in Ethiopia and particularly in this institution?

**Respondent:** I have worked in other wards but for the past 4 month, I am in ICU department and the ***usage of antibiotic is scaling up***. There is ***no guideline is no guideline or protocol*** to prescribe antibiotics so they simply prescribe as they want from their personal experience. Although I have no data but from my experience the use is not appropriate. Antibiotics are prescribed ***empirically without culture and sensitivity data to correctly differentiate the etiology***. To say over use or under use of antibiotics, we need to know the morbidity and mortality data because there are many infectious diseases and how we can say there is over use of antibiotics. ***But if we see the 2010 medication use data, ceftriaxone, meropenem, vancomycin and other antibiotics are among the top ten antibiotics in terms of use. For instance, vancomycin only costed the hospital more than 2 million ETB. So, looking at that data, we can see antibiotics are among these highly consumed medications.*** To say over use of antibiotic it must need clear data and if possible culture sensitivity test done is important. When one client comes to our hospital before giving antibiotic requesting culture sensitivity test is better way. If there is protocol to do these, as patient enter to hospital we can draw sample and doing culture sensitivity test is possible. I mean if you can treat the patient with ceftriaxone, why do you need to put him/her in meropenem? I see in ICU different thing which is below the standard where you can see many wastes here and there which is a high risk to infection. Due to this, my respect for ICU is minimized. Even admitted patient develop infection and for these infection other antibiotic prescribing. So this is big problem and you observe on site or I have photo.

**Interviewer:** How do you compare the use of broad vs. narrow spectrum antibiotics? Do prescribers tend to prescribe very broad-spectrum antibiotics empirically and/or to over prescribe antibiotics? What are the potential reasons for the preference of broad spectrum antibiotics? What factors do contribute for the overuse of antibiotics?

**Respondent:** of course they usually tend to prescribe broad spectrum but I am not sure if it is right or not I mean they might have justification for that. So to say how much they use, I need data. ***There may be fungal case but they want to prescribe antibiotic for all case.*** They not see other way and one drug is given for long time or ***longer treatment duration is common***. One time my friend’s brothers enter in ICU and somewhat improved but after some time he died by hospital acquired case, as to me, abnormal use of broad spectrum contributes to resistance in ICU and ***even sometime patients didn’t respond to broad spectrum antibiotics because there high possibility of transmitting resistant pathogens from patient to patient.***

**Interviewer:** What other contributing factors do you think contribute for that.

**Respondent:** Reason for antibiotic resistance is: ***one no protocol or guideline, there is culture test but low utilization of culture and sensitivity test, unavailability of morbidity*** data are contributing factors for use of antibiotics***. For instance, we know that culture to test sensitivity of vancomycin exists but we see vancomycin being prescribed without checking its sensitivity indicating its low utilization and that makes it among the highly consumed drugs. I mean*** ***there are 40 disks to check culture sensitivity test. From those vancomycin (they paid for vancomycin more than 2 million birr) is 40^th^ disk but they usually did not checked for effectiveness or its resistances indicating its low utilization.*** Patient must take the effective drugs which treat their case as last broad spectrum but after checking of culture test. ***Poor involvement of clinical pharmacist or lack of team work*** also contributes.

**Interviewer:** You said the use of microbiology is low, what do you think are the reasons?

**Respondent:** It need more in depth study but in my opinion, most health professionals are not aware of the availability of these culture and sensitivity test. But as a pharmacist, I don’t have more information on this area.

**Interviewer:** How do you describe patients’ pressure on use antibiotics? How about their preference of IV over PO?

**Respondent:** I have no experience inpatients’ pressure on antibiotics use.

**Interviewer:** How do you describe the current status of antimicrobial resistance? What factors do you think are contributing to increasing emergence and spread of antimicrobial resistance?

**Respondent:** ***The magnitude is subjective and I can’t give you objective evidences. But it is high even for the new antibiotics.*** I have experience in few patients, they treated by ampicillin, meropenem and vancomycin but patient didn’t show improvements and fever persisted. As to my perception, if study is done in this hospital, the resistant would high. ***My personal feeling is, if I become ill and I had surgery in this hospital, my fear of acquiring resistance is much higher than the surgery itself. I strongly believe that the possibility of acquiring resistance pathogen is very high and that makes to worry a lot.*** In addition to the inappropriate antibiotic usage, the hospital set up also contributes for increasing resistance. So, weak infection prevention practice with inappropriate antibiotic usage, hospital setup problem [at ICU, I tell you the system is not closed and no control movement. The door is always open which could be a reason for infection, so you have to use high standard antibiotic like meropenem but even using potent antibiotics many patients expired due to infection prevention] are factors for increasing resistance. Simply, what I say to you is there is no way of controlling infection prevention; if one patient resist for ***antibiotic then he will be not isolate so that simply contaminate/pollinate other patients in the hospital.*** Other health professionals do not take care or don’t not wear clean gown. ***Only two or three resistance cases could affects the whole hospital.***

**Interviewer:** How do you see the effort to tackle antimicrobial resistance? What possible measures do you recommend for better antimicrobial resistance containment practices to be implemented in this facility?

**Respondent:** Before we say we are doing something, first we have to know the magnitude of the problem but we don’t have that. We don’t know where we and if you don’t know the magnitude and it is difficult to intervene. If you do not know your challenge, you have no idea to tackle it or to find the solution for it, so to me we are asleep.

**Interviewer:** What possible measures do you recommend for better antimicrobial resistance containment practices to be implemented in this facility?

**Respondent:** I recommend, ***availing protocol*** for antibiotic usage restriction. Showing the problem is not sufficient and we have to develop a strict system that everybody has to follow. For instance, ***restriction of selected antibiotics*** such as meropenem and vancomycin to residents and interns. ***We are doing this with NPS and why don’t we do the same system for antibiotics?*** Otherwise we have risking our future life. After some year we have to check or evaluate the impact (outcome) of this system. Another is ***increasing knowledge and skill*** of health professional about antibiotic usage. Also we can ***apply antibiotic cycling*** meaning for some duration using one class antibiotics then shift to other (alternative antibiotics) after one or more years/ using some antibiotics at every interval. Prescribing one class of antibiotic for long time of time predispose for resistance of that drug which done by system. To implement this we need to have policy. We shouldn’t establish nonfunctional committee. Rather the committee should have clear duties and responsibility to implement given system. These committee members also should know their right and responsibility. There should be a system to make them accountable for that and there should be monitoring & evaluation of outcome.

**Interviewer: Interviewer:** You were telling me about restriction policy [the interviewer briefed how restriction policy works], how would you feel if it is applied in this institution? What are the barriers and enablers for this intervention?

**Respondent:** The ***current practice might be a barrier***. Currently, one senior prescribe then others follow it or anyone can prescribe as they want to prescribe. If I prescribe ceftriaxone, there is no one who evaluate the prescription and they can be easily dispenser dispense it. ***From my experience in this hospital, I have encountered few nonhealthcare professionals or no-prescribers who used to prescribe antibiotics.*** ***Prescription printed for BLH have no serial number so that dispenser do not know where it comes from or they are unable to evaluate it. Everybody prescribe vancomycin, ceftriaxone and other antibiotics.*** So, due to these irrational use will increase resistance, then etiology may change and difficult to improve the patient, they may deteriorate or no outcome after take of these antibiotics.

Other barriers are ***lack of clinical pharmacist*** (if they are present, they can evaluate each prescription and solve problem in patients side). In ICU, One patient paid 20,000 birr for drug and now 4 heparin vial opened at once, this show no monitoring and evaluation for prescribing and availability of drug. To solve these, the clinical pharmacist can directly follow the intake of patients in bed side. Other barriers are ***poor communication channels [b/n heath care professionals like b/n pharmacists, nurse and prescribers]*.** Resistance from prescribers can a barriers.

**Interviewer:** What other enabling factors do we have?

**Respondent:** Enabling factors for restriction can be ***commitment of pharmacy professionals*** including its management to be involved in such team works, availability of sensitized staff to wards antibiotic usage and antimicrobial resistance in hospital, and ***availability of DIC***. Also ***implementation of APTS*** is helpful for medication management which ultimately would increase availability of medicines.

**Interviewer:** Another type of antimicrobial stewardship intervention is prospective audit and feedback [the interviewer briefed how it works]. So, How would you feel if prospective audit and feedback is applied in this institution? What are the barriers and enablers for this intervention?

**Respondent:** I see it as a good intervention. Applying prospective auditing is better idea because pharmacists know what the problem is and which antibiotics are available; and physician knows proper prescription method, so ***collaboration work is better***. Pharmacist support physicians during prescribing each antibiotic Its creates good channel between physicians and pharmacists

If not collaborate pharmacist with physicians, from my experience patient had prescribed with other potent/high class and costly antibiotics since they don’t know it is available, due to lack of communication (gap) but the pharmacist knows this. Another Barrier is ***fear of pharmacist due to poor communication and physicians don’t have a trend to work with pharmacist. Being a new practice, physicians might be resistant to accept and work with pharmacist. On the other hand, I am not sure if pharmacists are knowledgeable to work with senior physicians with subspecialty. In general, k***nowledge of pharmacist and reluctance of physicians to accept to work in tea or accept pharmacists’ recommendation might be a barrier..

**Interviewer:** That is the end of my interview, do you have additional ideas to share with me?

**Respondent:** No, that is all I would like to discuss.

**Interviewer:** Thank you.

=================================================================================

## Pharmacist KI#13

Age: 27 years

Sex: Male

Department: Pharmacist [DIC Clinical Pharmacy]

Staff Position: Drug Information Pharmacist

Total work experience: 2 years

Number of patients treated prescription per day: 60

Number of patients with ABX prescription per day: 20

Total Interview time (duration): 46:33 Minutes

**Interviewer:** Ok, thank you. To start with my first question, how do you describe the current use of antibiotics in Ethiopia and particularly in this hospital?

**Respondent:** When we see antibiotic use; some professionals use appropriately ***but the more others who use them inappropriately*** without following guideline meaning prescribing as they want or following ***trendy kind of prescribing***. For example in URTI treatment, there are updated guidelines but they don’t use it rather they use their experience (trendy prescribing). If we see use in wards and OPD, in OPD most ***common problem is use appropriate dose*** but the use of broad spectrum antibiotics is not a big problem. But in wards, there is ***high usage of broad spectrum antibiotics where patients immediately start vancomycin then quickly switched to meropenem***. Thus, when microbial test is done ***most are resistance to all available antibiotics***. Other thing is abusing meropenem because they perceive vancomycin is becoming resistant. The problems in there is ***gap of communication b/n physicians*** [R1, R2 and senior]. ***The senior may say to prescribe fluoroquinolones or macrolides but we know that each antibiotics have different bioavailability and other attributes. They only ask it has been prescribed but they do focus on the choice of antibiotics and future consequence. This is resulting in problem of choices, some of them may not be available and then resulting problem of shortage. At times patient may suffer to get the prescribed class of antibiotic which might not be available in the country.***

**Interviewer:** So you think inappropriate use of antibiotics is a problem?

**Respondent:** Definitely. If you go to pediatric wards, you see a lot of admitted children can not be treated with the available antibiotics due to resistance, so life of many children is now at risk because due to resistance and as access to alternative antibiotics is very limited or no access to the possible options. Most antibiotic in our country have only one alternative from four or more possible option in one class. ***So my fear is resistance is and will affect us highly and patients may die due to lack of choice.***

**Interviewer:** How do you compare the use of broad vs. narrow spectrum antibiotics? What factors do contribute for the overuse of antibiotics?

**Respondent:** I have a fear of antibiotic resistance and this is mainly b/c physicians ***use most of the time broad spectrum antibiotics***. The reason they use broad spectrum is ***due to fear of infection and need of immediate cure, fear of accountability by junior prescribers*** ***meaning if the junior physicians prescribe antibiotics with narrow coverage they do fear the possible negative feedback/response from consultants which could negatively affect their future relation; lack confirmatory diagnosis*** e.g. a patient admitted with cough without investigation usually prescribed with antibiotics and once it is started you see the patient taking the medicine for longer duration without confirmation and not want to stop already prescribed drug. ***One patient takes antibiotic without any reason for long time***. Antibiotic once prescribed don not stop and even seniors don not usually intervene. ***Poor follow-up and poor transcription are also common problems. For instance, one time we found a patient taking ceftriaxone with ceftazidime.*** So, physicians should take history from patient every time and check which drug is discontinued and which should be continued.

The big problem of inpatient department is they ***don’t follow patient properly and don’t properly stop discontinued antibiotic*** rather we find such cases in dispensing unit. So that even after real infection confirmed, the treatment may not work b/c the has been already exposed to unnecessary antibiotics. So they may not respond even to broad spectrum antibiotic as they might already develop resistance. Now physicians and pharmacist fear is about antibiotic resistance. In antifungal resistance is not an issue but antibiotic resistance is a big issue. Another big problem is ***lack of clinical pharmacist involvement*** in clinical wards. I have seven months of experience in Gondar Hospital as clinical pharmacist and by then we check every point ( seven points) like drug adverse effect, others important point and then we discuss with intern, resident and seniors, and finally we change drug if the evidence showed us to do so. But here it is difficult because they routinely apply what they have learnt and do not want to discuss or discover new thing rather they go as it.

**Interviewer:** How do you describe patients’ pressure to use or not to use antibiotics?

**Respondent:** They may say that they have already took similar antibiotic for 15 or 30 days before and don’t see any improvement and even then question why he prescribe it again. We discuss this issue with physicians and the seniors are receptive but still there is problem with the junior. ***Juniors immediately accept their senior physicians message without questioning …they see it as Gods word that shouldn’t be violated*** but for our suggestion they are so reluctant even they know it is right. Another problem is documentation.

**Interviewer:** You were telling me resistance is a problem and it is increasing. From your experience, which pathogens are commonly encountered?

**Respondent:** Yes, in this hospital resistance of antibiotic is increasingly high. From my experience more resisted pathogens are staph coccus, staph aureus. The problem is microbiology test is not done on time but at the same time there is fear of antibiotic resistance. In this hospital if culture test is done, it is mostly after the patient started taking antibiotic. From my experience, in pediatric ward one patient was admitted with sepsis and he took different antibiotic but do not improve, rather he become deteriorated and infection was radiated to different parts of body. They tried meropenem but meropenem resisted and finally he was susceptible only for tobramycin and amikacin but these drug are not available in Ethiopia. I am not sure what the fate of the patient was. The reason is they started from broad spectrum but they do not started by grading.

**Interviewer:** What factors do you think are contributing to increasing emergence and spread of antimicrobial resistance? You were telling me about inappropriate use, what others factors do we have?

**Respondent:** One, they ***don’t use/follow treatment guideline***, I mean there are updated guidelines but I don’t see they follow these guidelines. Second, absence of follow up on prescribed medicine or ***unavailability of professionals like clinical pharmacist who can audit and follow antibiotic use*** , ***not using culture sensitivity test*** before prescribing, ***high use of broad spectrum or tendency to use last generation antibiotics.*** Inappropriate use of antibiotic and less involvement of clinical pharmacist in each point with well recognized level are therefore a major factor. I also see most professionals do give adequate attention to drug resistance indicating ***poor awareness about that*** and I think they do also a challenge or knowledge gap in choosing the right correct antibiotics.

**Interviewer:** How do you describe the current status of antimicrobial resistance (at institutional and national level)? How serious is the problem? Do you think it is a growing public health treat or not?

**Respondent:** ***Resistance is now increasing and this is creating a problem in procurement.*** Most drugs to our country as fund/support program. Now AMR is worldwide problem but the problem is disturbing for us as we have ***problem of access to possible choices of antibiotics*** which can be easily accessed in other countries. For instance, there were times where meropenem and ceftazidime were stocked-out for about six months but this is not a problem for other countries. Meaning other countries have many option but we have no option. On the other hand, we do not reserve those antibiotics we are losing those limited options. ***So, my fear is how to treat patient if resistance continues in this manner***. Also we have low supply and availability of antibiotic. Most of the time antibiotic misused and abused for URTI disease like pneumonia type.

**Interviewer:** What possible measures do you recommend for better antimicrobial resistance containment practices to be implemented in this facility?

**Respondent: *It is good to follow the treatment guideline*** i.e. to start the treat from first or second generation and increasing up to fourth or other generation. ***Also close follow-up and involvement of seniors in decision should be enhanced.*** ***Increasing the role of clinical pharmacist*** with additional specific trainings so as to add knowledge and increase their self-confidence. These clinical pharmacists can follow or audit patient use of antibiotics. ***Unless specific training is given to pharmacist, it is very difficult for a junior pharmacist to work with senior physicians.*** Also there should be sharing of different knowledge or skill b/n us and physicians or improve team work by integrating all relevant professionals so that we can learn from them and they can learn from us. ***The knowledge obtained in the team is not used only for one patient but it can used for many patients with similar cases.*** ***In this hospital if it started by integration, next season pharmacists and physicians use this system and become influential in different area across the country.*** The benefit is win-win, mutually for pharmacist and physicians. The current government problem is expiring medication and also resistance which make the government to pay a lot of money. This problem can be controlled or prevented by team work starting from nurse up to physicians. Strength of clinical pharmacist may solve problem b/c our contact with all staff members from nurse up to physicians (intern, residents and seniors). Sometimes there is difference b/n nurse administration and physicians prescribed, so we can serve as bridge b/n nurse and physicians.

**Interviewer:** Ok, what do you feel if restriction policy [the interviewer briefed how restriction policy works], is applied in this institution? What are the barriers and enablers for this intervention?

**Respondent:** To apply restriction, ***first do we have the man power?*** Do we have senior physicians that ca be available all the time? But you can’t leave its implementation b/c always there is at least one senior in each ward but they don’t see it as their responsibility. But if there is restriction policy, then everyone will consider it as a mandatory. Then, you can assign responsible professionals for each area with responsibility. As my opinion the main challenge is physicians fear to take risk or not risker taker. So before starting restriction, there should be a forum for open discussion with physicians about importance and side effect of single medication in hospital and nationally. Otherwise it could have repercussion for each professional. So, if doctors believe in this idea (restriction), we can create better environmental for clients. ***But physicians not want to take risk or they are not risk taker.*** For example, if there are new and expensive medicine, and we ask them to prescribe the medicine but said they don’t want to take risks. But if you show them about importance of this intervention for the patient or nationally, they may accept it.

**Interviewer:** Interviewer: Another type of antimicrobial stewardship intervention is prospective audit and feedback [the interviewer briefed how it works]. So, How would you feel if prospective audit and feedback is applied in this institution? What are the barriers and enablers for this intervention?

**Respondent:** When you give feedback, feedback should be for specific prescribing doctor who prescribe it. So as there is round of physicians the auditing should be designed considering that rounding circumstances. This intervention is good as prescribers would be aware of that and improve their prescribing behavior thinking that it will be audited. So that doctors take care for prescribing drugs and prescription by understanding there is prescription evaluation and auditing. This will have direct impact for patient, prescriber and others. After that doctors may discuss each other and reevaluate their prescribing behavior. Also it is important to tackle the problem from prescription before reaching to patients. This information can be disseminated to other fellow professionals too. ***But anyways, prescription auditing is a very important thing.***

**Interviewer:** What is challenge or barriers of it?

**Respondent:** From pharmacy aspect, ***manpower is main barriers*** but now somewhat it is improving and we are recruiting clinical pharmacists but still there is ***gap of knowledge gap*** so that training is important. Also it is good to ***create positive environment for the team*** and for benefit of patient b/c we had ***no previous experience of working in team.*** If we can create positive environment by showing them the burden or magnitude of the problem, I don’t think there will be major barriers from pharmacist side. Time of ID physician is other problem. ***But, infrastructure is not as such a major problem.***

**Interviewer:** What do you think are the enabling factors to implement antimicrobial stewardship environment?

Respondent: There are forcing factors and now it becomes a must to do, not an option. So the high prevalence and magnitude of the problem of resistance I mean with increasing morbidity and mortality we have to act. The problem is increasing, it is going out of control and it is now a must to act. Yes, there are challenges but if you let the management about the magnitude of the problem, I am sure they will fully support us. Honestly, they know it but they don’t give it adequate attention. When there is discussion, you can see how much they are worried about it but they don’t act and thus we can easily implement. ***The fact that the problem is big and being recognized by practitioners and the management is an enabling factor.*** Another thing is the increase in number of clinical pharmacists.

**Interviewer:** That is the end of my interview, do you have additional ideas to share with me?

**Respondent:** My suggestion is, if you want to implement this intervention you have to involve the senior physicians and the management.

**Interviewer:** Yes, you are right and they are already involved and we will do so. Thank you.

=================================================================================

## Pharmacist KI#14

Age: 25 years

Sex: Male

Department: Pharmacist [Clinical Pharmacy]

Staff Position: Ward Pharmacist

Total work experience: 2 years

Number of patients treated prescription per day: 65

Number of patients with ABX prescription per day: 40

Total Interview time (duration): 27:33 Minutes

**Interviewer:** Ok thank you. To start with my first question, how do you describe the current use of antibiotics in Ethiopia and particularly in this institution?

**Respondent:** Most of the time antibiotic usage inappropriate. For instance there are antibiotics that prescribed for almost all patients, it is a trendy prescribing. Antibiotics e.g. like amoxicillin or ceftriaxone are simply prescribed in firstly empirically without lab investigation or evidence. Antibiotic prescribed without any evidence for any client who is may need or may not antibiotics (meaning the case can be viral or other infection which don’t require antibiotics but the first choice is to simply, blindly prescribe antibiotic.

**Interviewer:** How about the use of broad vs. narrow spectrum antibiotics? Do prescribers tend to prescribe very broad-spectrum antibiotics empirically and/or to over prescribe antibiotics? Why?

**Respondent:** They mostly use broad spectrum to cover any infection because they have fear of infection and they wanted to settle the problem immediately or see immediate effect. Another reason is lack of well-equipped laboratory investigation set-up, then if resistance occur simply change to other antibiotics. Also, antibiotic supply is not sufficient as we compared to the need. We don’t have some very essential medicines and this is forcing prescribers to shift to highly potent antibiotics like meropenem. ***I think there is also trendy prescribing habit, I mean if the consultant prescribe one potent antibiotic then we usually see repeated similar prescriptions coming from the residents or interns.*** So problem of drug supply, unavailability of strong culture sensitivity tests and ***receipt or copying consultant physicians prescribing habit without questioning or supporting evidences*** are reasons to increasing the inappropriate use of antibiotics and then resistance.

**Interviewer:** How do you describe patients’ pressure to prescribe or not to prescribe antibiotics? Also their preference of IV and PO?

**Respondent:** Usually admitted clients have low adherence in P.O and they wanted IV medication. Also after long stay they differentiate kind or type of antibiotics so after prescription most clients said that this is not my drug, they say this drug is not convenient for me or it doesn’t work for me. ***Injectable drug is their preferable choice.***

**Interviewer:** Why do you think they prefer injectable drug?

**Respondent:** sometimes they differentiate by color of cover and if cover change they don’t believe it heals them. If they have good improvement in first drug don’t believe in the next drug. There is no one who don’t want to prescribe antibiotic in my experience

**Interviewer:** How do you describe the current status of antimicrobial resistance (at institutional and national level)? How serious is the problem? Do you think it is a growing public health treat or not?

**Respondent:** I am not participated in ward round but depending on prescribed drug, there is drug resistance in our hospital because in our hospital one the first line drugs prescribed is usually high class antibiotic [broad spectrum antibiotics] and not start from low class antibiotic. We see admitted clients do not improve by first line antibiotic and that forced to use the broad spectrum ones***. I personally believe that prescribing broad spectrum is increase from day to day which indicate increasing of antibiotic resistance. Even there is no fear to prescribe new broad spectrum and potent antibiotics.***

**Interviewer:** Do you face any challenge in your daily practice as a result of antimicrobial resistance? How does it affect you and the patient?

**Respondent: *They tend to prescribe new broad spectrum antibiotics and its supply is not equal with demand. Hence, these antibiotics are stocked-out in one or two weeks’ time which resulted customer and staff complaint or dissatisfaction.*** Other reason for resistance ***is prescriber’s problem*** like they prescribe for unspecified antibiotic without culture evidence by simply relying on sign and symptom without investigation. Also, since patients don’t get adequate counseling patient adherence is low, they discontinue in two or three days even though it was prescribed for 14 days like Augmentin because they have no knowledge or awareness about antibiotic usage and its resistance, also ***lack of good counseling or good professionals***. These is ***shortage of health professionals for instance there are a lot people waiting you, you can’t take time to counsel the patients because you hear patients complain*** of longer waiting time and thus you want to dispense as soon as possible. ***Again there is lack of commitment from professionals***. Another thing is pharmacy professionals dispense antibiotic without counsel even if there are no other clients waiting in the area.

**Interviewer:** You were telling me prescriber behavior as a factor to increase AMR but what other factors do you think are contributing to increasing antimicrobial resistance?

**Respondent:** From my observation and experience the culture of working in team is very weak. I think it might be b/c the complexity of the hospital. For instance 8^th^ floor clients take prescribed drug from OPD, emergency or inpatient pharmacy so difficult to communicate each other, there is no team work in these hospital and staff even don’t know each other. In other hospital not in Addis Ababa they communicate each other and clinical pharmacist are contributing well. So we have weak team work.

**Interviewer:** How do you describe the utilization of laboratory findings in the diagnosis of infectious etiologies? What are the major problems you experienced?

**Respondent:** The microbiology have suffered with shortage of supplies. There is frequent stock-out and shortage of reagent, there is sustainability issue and this might but due to shortage of staff and knowledge of staff or poor communication with the supplier (PFSA) b/c while they say they have these supplies, you see shortage in TASH. So, it may be system problem because manager to manager communicate they said that it is available but when we go there is no reagent.

**Interviewer:** How do you see the efforts to control resistance problem?

**Respondent:** To control resistance of antibiotic, the is a good improvement in the laboratory. But I don’t see such a concerted effort to tackle the problem.

**Interviewer:** What possible measures do you recommend for better antimicrobial resistance containment practices to be implemented in this facility?

**Respondent:** First I would recommend to make a step-wise prescribing policy. Instead of jumping to broad spectrum antibiotics there should be a policy that restrict the prescribing of such antibiotics i.e. they should be prescribed if the narrow spectrum failed to respond. Step wise of drug prescription means prescribing low class to high class antibiotic and controlling prescription of high class in as first line drug. Second, strengthening laboratory setup and diagnostic facility is also a priority intervention.

**Interviewer:** You were telling me about restriction policy [the interviewer briefed how restriction policy works], how would you feel if it is applied in this institution? What are the barriers and enablers for this intervention?

**Respondent: I can see restriction has more advantages! But the challenge might be** the consultant may not be available all time or it is difficult to find consultant when they are needed. If consultant can be availed all the time, the using restriction is more advantageous. Another thing is, resistance from frontline prescribers. ***But if it comes and is supported as policy, everyone will accept it.***

**Interviewer:** What other enabling factors do we have?

**Respondent:** These time the number of ward pharmacy is increasing and this improved easy access to medicines and also resulted in good communication with other staffs in the ward.

**Interviewer:** Another type of antimicrobial stewardship intervention is prospective audit and feedback [the interviewer briefed how it works]. So, How would you feel if prospective audit and feedback is applied in this institution? What are the barriers and enablers for this intervention?

**Respondent:** Prospective audit and feedback is important for patients b/c it is done in team work. It can control the unwanted or unplanned usage of drug and avoiding wastage of medicine. Also drug will reach to clients effectively.

Its challenge is ***organizing of a team could take more time and very difficult***. As there ***no culture of team work*** and it will take more time to organize as a team because physicians may not accept the pharmacy professionals, also the educational background of pharmacy is not up graded, only two master pharmacy are available in the hospital. So the ***knowledge gap or the low level of education* and specialty of pharmacists** **[lacks infectious disease related and high level trainings]** could be a barrier to bring these two professionals to work together. I mean, pharmacy professionals (low level of knowledge and skill of pharmacists) is not adequate enough to equally contribute or challenge the seniors. On the other hand, ***shortage of well-trained pharmacy professionals*** could be a barrier to implement the program and this might be an issue for other professionals too.

**Interviewer:** What enabling factors do we have?

**Respondent: *Implementation of APTS is*** a good factor to ***increase availability*** of antibiotics which is an enabling factor. ***Willingness and commitment of pharmacy professionals*** to implement such intervention is also an opportunity. Again, the ***increasing acceptance of the role of clinical pharmacy by other health professionals*** is enabling factor.

**Interviewer:** If these two interventions are to be implemented, how do you recommend us to do. I mean should they be implemented concurrently or sequentially?

**Respondent:** It is good if you both are implemented together.

**Interviewer:** That is the end of my interview, do you have additional ideas to share with me?

**Respondent:** No, that is all I would like to discuss.

**Interviewer:** Thank you.

=================================================================================

**Interview with physicians**

## Physician KI#01

Age: 36 years

Sex: Male

Department: Pediatric Ward

Staff Position: Consultant Physician[ID fellow]

Total work experience: 12 years

Number of patients treated per day: 20 patients

Number of patients with ABX prescription per day: 10 patients

Duration of interview (minutes): 47:33 Minutes

**Interviewer: Ok** thank you. So start with my first question, how do you describe the current use of antibiotics in this institution or even at country level?

**Respondent:** There is no strict or stringent regulation of antibiotic use in Ethiopia. Even if the regulation exists since there is a lack of well-functioning executive body, a very large amount (number) of medications is dispensed without prescription (over the counter sales is high).

**Respondent:** As a consequence of this, a lot of research was being conducted in this area in the past three years, the awareness around hospitals was increasing and also the ministry of health took a special interest and opened up an agenda trying to address regulation on antibiotic but there is still a problem of proper practice (implementation). The problem isn’t only seen on commonly used antibiotics but also for example; as in practice, a simple correct pronunciation of the medications suffices in order to be able to purchase them from any community pharmacy (without the request of a prescription). ***I mean if you correctly name the name of any antibiotics that preserved to be used for serious infections such as vancomycin, you can get any amount without prescription as over the counter. Hence, antibiotic use regulation is very weak.***

**Interviewer:** Ok, over the counter is common in the community pharmacies. How do you describe the use of antibiotics in hospital?

**Respondent:** antibiotic use in hospital is not appropriate. First we don’t have strong microbiology that guide our prescribing practice. ***Most of the prescribing practice, especially in the private sector, is more of market oriented which mostly focus on profit and immediate result oriented; and they tend to prescribe more antibiotics.*** We also don’t have a means to exactly identify the type of infection and we frequently change antibiotics to potent or las choices. So you see antibiotics like Vancomycin and meropenem are highly abused especially in the private hospital. For instance ertapenem is not used in this hospital and even I never used it in this hospital but it is highly consumed in the private hospitals. So antibiotic use is highly misused in the private sector.

**Interviewer:** so do you think inappropriate use of antibiotics is a problem in Ethiopia and in this institution? How do you describe the pattern of this problem over time (Is it increasing, decreasing or stable)? Why?

**Respondent:** In pediatrics there is a definite overuse of antibiotics, always. ***This is because one parents do not want to see their children in pain [child can’t tolerate pain] or are very insistent on immediate use of the medications, the other is even if one doctor denies antibiotic prescription for a child another pediatric doctor from a different health institute will eventually provide it.*** Over use is high, ***there is also use of antibiotics for viral infections either by a poor diagnosis or just the lack of knowledge, dedication or confidence to explain why antibiotic use won’t be necessary for a viral infection.*** So in conclusion, there is an overuse in the private sector.

The prescription usage in hospital pharmacies is good but the antimicrobials are still not properly utilized. ***The contributing factors for this drawback*** are unavailability of microbiologic studies, for guidance a misunderstanding or failure to understand the cause or pattern of infections, switching up between antibiotics within a short period of time, and in the private sector there is an abuse over highly potent antibiotics (like meropenem & vancomycin for example) because the doctors prescribing behavior is business or profit oriented; hence private hospitals even before the government hospital pharmacies start availing them.

**Interviewer:** How do you compare the use of broad vs. narrow spectrum antibiotics? What are the potential reasons for the preference of broad spectrum antibiotics?

**Respondent:** Coming to TASH (or similarly other government hospitals) patients here are often severely infected [critical patients] so potent broad spectrum antibiotics are used. ***This is done for the sole purpose of wanting to decrease the risk of mortality whereas in private sector doctors tend to prescribe broad spectrum potent antibiotic before this severity in the disease is reached, for profit and immediate effect.***

**Interviewer**: So if I understood you well, the contributing factors you mentioned are like poor microbiology capacity and patient pressure. Are there other factors?

**Respondent:** yes, the need to increase ***profit where our practice is market centered*.** There is also a big problem of ***medication scarcity in government hospitals***. There is also the issue of the patient’s ***economic capacity hence purchasing capacity & availability of antibiotics*** in the facility are greatly limiting. But antibiotic availability has rapidly increased over the past 3 years. For example drugs like amikacin, moxifloxacin, amoxicillin, levofloxacin, that were only familiar to us in textbooks and not in real practice prescribing are now available in the market (if you really look). Currently as mentioned earlier a lot of antibiotics have been availed the problem here though is because the health system had been deprived of these medications before doctors tend to overuse (overprescribe) them now that they have been availed. ***Their use appears to be like a current fashion trend that everyone seems to like. An example could be cefepime introduction into our hospital in recent time but a consequent cefepime resistance.*** Like this many more potent antimicrobials are being rendered useless due to improper use.

**Interviewer**: You telling me about patient pressure, how do you see their influence in antibiotic prescribing and their preference of IV and PO?

**Respondent:** There are patients who need IV prescription. Yes especially from rural area patients want to preferably get IV medications **but the parenteral medication only seeking behavior has decreased.** Such behavior is decreasing over time. **On the other hand, Urban families show a decrease in desire for use of antibiotics for their children in fear of overuse and an understanding of antibiotic resistance as a consequence.** So from my experience, urban patient population awareness in improving and it is now easier to convince them that antibiotic is not useful to their child.

**Interviewer**: Do patients ever express concern about themselves/ their child being prescribed antibiotics? Do they ever pressure you into not prescribing antibiotics?

**Respondent:** In the past two years the awareness of parents of the patients has improved but there is still a desire for antibiotic use by parents. ***Even though few patients from rural areas request specifically for IV medications, urban families show a decrease in desire for use of antibiotics for their children in fear of overuse and an understanding of antibiotic resistance as a consequence.*** Especially in the urban area, people came by reading different sources or ask their relatives/families and challenge you even the dose. This understanding can bring both a challenge and an advantage to the prescribing behavior of a physician. If parents resist to take antibiotics: the advantage is in case of mild infections that do not require antibiotic use but the challenge is faced in having to convince patients or parents that use of the antibiotics is necessary when the infections are severe. ***But these cooperative occurrences from patients are infrequent or rare.***

**Interviewer**: How do you describe the current status of antimicrobial resistance (at institutional and national level)? How serious is the problem? Do you think it is a growing public health treat or not?

**Respondent:** Of course it is a huge threat. Antibiotic use as currently practiced is definitely a big problem mainly because of the economic incapacity of the country to supply drugs; ***so it is necessary that we use what we have on hand properly or else we could lose a lot of patients due to this malpractice.*** There aren’t many stewardship programs & even the ones that exist aren’t appropriately managed. The problem is increasing over time and if not managed, it will have serious consequences.

**Interviewer**: when you se it over time how do you see its trend?

**Respondent:** The trend is increasing over time. When you have chronic shortage of drugs and when new drugs are imported, then they started to prescribe them like a fashion, recklessly . Even potent antibiotics are used without regulation and as a result we are losing them too fast.

**Interviewer**: How frequently do you encounter drug resistance organisms within your clinical practice? Which types of pathogens/infections do you think have more resistance profile?

**Respondent:** Multidrug resistance (MDR) – 8 pathogens out of ten are resistant. With PAN drug resistance- 5-6 children in a month, for which no drug works at all. And XDR- 2 out of 10 children. We don’t see a pathogen which is not resistant to at least MDR…..SSS is very rare to almost none. There definitely won’t be a non MDR pathogen but klebssila the most frequent, Acinetobacter the second frequent and E.coli & Enterococcus third frequent.

**Interviewer**: In which samples do you see a lot more reports of antibiotic resistance?

**Respondent:** They are most frequently obtained from blood samples.

**Interviewer**: Do you face any challenge in your daily practice as a result of antimicrobial resistance? How does it affect you? How does it affect the patient?

**Respondent:** Use of potent drugs is necessary in order to tackle the drug resistant microorganisms that are common to the TASH hospital setting. For example for the currently circulating klebssila are highly resistant and we would need potent antibiotics at least peptazo, ciprofloxacin & meropenem. Piperacillin-tazobactam & meropenem are necessary but they are difficult to find as compared to ciprofloxacin. But the hospital might not be able to provide these drugs either due to ***unavailability of budget or the drugs in the country which leads to increase morbidity & fatalities hence negatively affecting the prescribing practice.***

**Interviewer**: In general what factors do you think are contributing to increasing emergence and spread of antimicrobial resistance?

**Respondent:** First reason is ***microbiology unguided treatment*** (prescribing on an educated guess only). For example, most Staph aureus isolates can be a good testament to this fact. For the past 18 months with the help of Dr. Makeda, ***we have been able to treat majority of the infections with drugs other than vancomycin like cloxacillin & cotrimoxazole but prior to this practice we used to use vancomycin for every staph culture by assuming it was methicillin. resistant.*** The second reason is there is ***no committee to control prescriptions or the prescribing practice***. The third reason could also be ***poor infection prevention practice***. For example, ciprofloxacin & cotrimoxazole in the prior two months there have been several neonatal klebssila infections, this indicates our poor infection preventing practices and possibly a cross infection occurring within the pediatric center after a 4 to 5 days stay of the child in the center. The fourth reason could be ***poor adherence***. Most patients admitted are from the rural areas and they come to TASH forced to leave on average of 4-5 children unattended at home so even though they understand the severity of their disease, they will often choose to get back to their children & their work rendering themselves or their sick child untreated. The stock out of antibiotics ***[unavailability of medicines] due to poor communication*** between pharmacists and physicians and PFSA is also another factor. Although working in team is improving, but yet it needs more effort.

**Interviewer**: How do you describe the utilization of laboratory findings in the diagnosis of infectious etiologies? Are microbiology lab results timely communicated to the treating physician in this institution?

**Respondent:** In pediatrics, most doctors send samples to the lab for analysis there is also ***poor technical & interpretation sill in determining antibiotic susceptibility as well as poor sample collection, methods & inappropriate sample transportation to the laboratory. Although there are problems in these three area, they are still a problem.***

**Interviewer**: How frequently do treating physician request for cultures of relevant clinical samples to guide his/her choice of antibiotics? If less frequent, why?

**Respondent: *In previous time negative lab results were common which discouraged doctors from sending samples to the lab. But the current laboratory practices are very much improved. Relatively now it is better.***

**Interviewer**: Do you have any complaints on the laboratory service or its quality?

**Respondent:** Still there is a problem of delayed notification of positive results to the doctor by the lab & of the same time there is a weak follow up practice by physicians responsible for that patient so the problem is bilateral though I feel it seems to weigh more on the physicians side since there is a usually a high work load burden on the microbiology/labs personnel.

**Interviewer**: If the problem is this much, do you think enough is done to tackle the problem? What possible measures do you recommend for better antimicrobial resistance containment practices to be implemented in this facility?

**Respondent:** There really aren’t any strategies currently in place in TASH to curb drug resistance. But one solution could be **categorizing the antibiotics in terms of hierarchy**, for example antimicrobials that can be prescribed by any physician (category one), antimicrobials that can be prescribed by interns or general practitioners by consultation with their residents (second category), third category antimicrobials prescribed by consultants & a fourth category of antimicrobials to prescribed by ID consultants only. But its only with strict implementation practices that this can be a solution, all ***parties involved; i.e***. the physicians, the pharmacists. ***The hospital administration would have to strictly abide by this strategy with no exceptional social factors by any professional.*** It is also necessary that the non-medical society is well informed about the issue of drug resistance so this strategy will also have to give emphasis to a good public relation approach. Imagine you go to hospital with child and you have been told that antibiotic will not be prescribed unless it is authorized by someone. It might have backlash, so we have to create awareness among the public too. The ***antibiotic guideline is also very important***. One of the challenges for the Infectious Disease practice is lack of an antibiotic guideline (which will hopefully improve in the near future).

**Interviewer**: From your experience, can you think of potential factors that might influence the antimicrobial resistance containment practice in the hospital?

**Respondent:** The antimicrobial stewardship as well as the auditing principles in place of TASH are good but the problem lies in proper implementation. Implementation needs to stringent to avoid corruption & exploitation of antibiotics by government officials for personal gains.

**Interviewer**: How do you see implementation of prospective audit and feedback intervention [interview briefed how prospective audit works]?

**Respondent:** I think the principles are very good. Like auditing is good but it is the implementation that matters. ***I mean you can think of as if the government allocated you some fund, then you have the responsibility to make sure the fund is utilized efficiently and you are expected to report, there is even external auditors. Likewise, antibiotics are resources that should be audited to make sure they are used appropriately. used wisely.***

**Respondent:** There is an autonomy problem regarding auditing but it will resolve with time. Previously infectious cases that come to doctors attendance were few due to the pharmacists believe that they could manage it on their own but now that microbial yields are improving & positive results are seen from result oriented management of patients; the number of visiting doctors seeking medical attention has increased from an average of one to two per day to around five to ten patients per day.

**Interviewer**: How would you feel if prospective audit and feedback is applied in this institution and a multidisciplinary team review and give you feedbacks on your prescribing?

**Respondent:** I think it would be very good. Here in TASH over the past couple of years there is great improvement and an increased cooperation seen between the health care professionals on antimicrobial use but there is a gap in information exchange between PFSA, the hospital administration & the physicians as well as a poor working relationship among the 3 parties. But now in the past year, there is a good communication between doctors & the pharmacy head in TASH. They let us know what is available & what is not in the hospital pharmacy but, we physicians do not return that favor when we stock out. There might be an excess stock of vancomycin in PFSA for example while I am struggling with a shortage of vancomycin in TASH but because there is a poor link or weak information exchange system between PFSA, the hospital administration and myself the drugs might probably expire in stock while I fail to provide the prescription to the patient in TASH.

**Interviewer**: Can you help us understand the potential factors that might influence the implementation of prospective audit and feedback in this institution? How do these factors become barriers?

**Respondent:** I expect to see great challenge with the surgical departments like Gyn/Obs so greater focus should be given to these departments because it’s in these departments that care for antimicrobial is neglected **[resistance from frontline staff].** I believe, another challenge I expect to see is within **the administration [management level] support.** As seen in practice the surgical staff, i.e., the doctors & nurses have a strong working relationship or understanding so when an unfamiliar pharmacist responsible for implementing changes regarding antimicrobial stewardship, the opposition/resistance that he/she could face from the surgical staff is very high hence the full support of the administration to back this program and reassure its practicality is necessary.

A certain physician would have to be able to **dedicate himself/herself 24 hours** to this program, focused & with a good cooperative relationship with the ward pharmacists & the microbiology unit. So the administration in support of this program should come up with **incentives to encourage a certain physician to fully dedicate himself/herself** to the cause in order to assure the success of the antimicrobial stewardship program as it sores not one or two but thousands of lives.

**One enabling factors; is the recognition from the ministry of health** and in turn, the **awareness that is being created in medias** are very good things.

**Interviewer**: That is the end of my interview, is there anything you would like to add?

**Respondent:** it is going to require full dedication until this approach has a stable frame work, so probably about 5 years of work has to be put into it. And the success of all the important components has to be reassured, that is the auditing, the dispensing pharmacists has the stewardship program, the physicians and the administration will all be here to work in unity and in understanding.

**Interviewer**: Thank you for your time!!!

=================================================================================

## Physician KI#02

Age: 29 years

Sex: Male

Department: Emergency Ward

Staff Position: Resident Physician[R3]

Total work experience: 3 years

Number of patients treated per day: 20 patients

Number of patients with ABX prescription per day: 20 patients

Duration of interview (minutes): 37:34 Minutes

**Interviewer:** Ok thank you. How do you describe the current use of antibiotics in this institution or even at country level?

**Respondent: *For me there is no standard use of antibiotics in this country because use greatly differs between the private hospitals and the government ones. in this three years of my experience, one day I have encountered a child prescribed with ceftriaxone at night shift and in the morning the child’s father expressed his observation/frustration by saying that “….wait, is this medicine prescribe to all patients like holy water.” So now it indicates every person is observing that antibiotics are prescribed to everyone suspected not confirmed, I mean to those who need it or don’t need antibiotics. So you can see antibiotics are overused.***

**Interviewer:** Do you think inappropriate use of antibiotics is a problem? Which problems are more common in this setting? Why? How do you describe the pattern of this problem over time (Is it increasing, decreasing or stable)? Why?

**Respondent: *Yes, it is definitely a problem and it seems o be increasing overtime as well.*** The more common problem is seen with the decision of when is the best to provide broad spectrum antibiotics treatment and when to initiate early antibiotics treatment in general. ***Most of the time we tend to start with broad spectrum antibiotics and I think if they don’t respond it is very difficult to tapered it down.***

**Interviewer:** How do you compare the use of broad vs. narrow spectrum antibiotics? What are the potential reasons for the preference of broad spectrum antibiotics? What factors do contribute for the overuse of antibiotics?

**Respondent:** Prescribers tends to use broad spectrum antibiotics. One promoting factor for this performance could be the different researches that are published in favor of early initiation of antibiotics use without specification on as to when it is best to initiate broad spectrum use. While the other is the ***mortality benefit*** that is offers. Second factor is that there is ***no resistance of sensitivity profile available and microbial support for the antibiotics is very low*,** prescription is based on assumption that patient will have most likely acquired hospital infections rather than proof of the hospital acquired infections being present. **Third factor is *unavailability of reliable culture result* when physicians send sample for the laboratory**. Even if some ***culture and sensitivity are done, they are done for drugs which are not available in the market.*** On the other hand there is shortage of disc for the necessary drugs. But at the same time ***doctors rarely send samples appropriately (prior any antibiotic use) if they send any samples at all****.* Fourth factor is ***unavailability of drugs that push physicians to*** make a decision that could result in influencing fellow physicians and force an unjustified trend.

**Interviewer:** How do you describe patients’ pressure to prescribe antibiotics? Do you ever feel pressure from patients to prescribe antibiotics? If yes, how do they pressure you?

**Respondent:** In my experience at TASH, I have not come across any patients who push my prescribing decision or choice of antibiotics selection unless it ***is cost*** related but in the private sector there are patients that are opinionated about their treatment plans and comment accordingly. But in public I haven’t come across patients asking you to prescribe antibiotic b/c of antibiotic but yes in terms of cost.

**Interviewer:** Do you ever feel pressure from patients to prescribe IV antibiotics instead of PO? Why do you think so?

**Respondent:** Yes such prefers exists. Some patients tend to prefer injectable in light of wanting to either prolong their stay in the hospital or shorten it but it is usually cost that is the a most influencing factor for preference by the patient not ROA or medication selection. ***There is also a strong belief among few patients and the public that IV is more effective and they don’t think PO are effective.* *But the major factor for compliance is not route of admin but the cost.***

**Interviewer:** Do patients ever express concern about themselves/ their child being prescribed antibiotics? Do they ever pressure you into not prescribing antibiotics?

**Respondent:** In the private hospitals yes bit not common in public hospitals.

**Interviewer:** you were telling me some issue about resistance, how do you describe the current status of antimicrobial resistance at institutional and national level? Do you think it is a growing public health treat or not?

**Respondent:** Antimicrobial resistance, though I am not supported by research evidence or studies to present to you with specifics, ***I would say antimicrobial resistance is big problem that worries me. It is growing public health threat because it is a draw back in achieving cure rates and also incurs additional cost due to failure of previously successful and easily available older and cheaper molecules.*** You know we don’t have evidence and most of our decisions are based on westerns recommendation but I can see there is a huge gap and resistance a growing public health threat. I have a sense that a medicine that used to treat five years ago is not working now; previously you can treat many infections using ampicillin but now you don’t see a physician prescribing that antibiotic. Still our treatment decision is based on expert opinion, no strong evidence but you can see its treatment outcome.

**Interviewer:** Which types of pathogens/infections do you think have more resistance profile? How is the pattern of antibiotic resistance across the years?

**Respondent:** Resistance is increasing year to year. But I am not sure about which specific pathogens of infections have greater resistance profile.

**Interviewer:** Do you face any challenge in your daily practice as a result of antimicrobial resistance? How does it affect you? How does it affect the patient?

**Respondent:** My daily practice has changed when compared to last year. This is because most patients that were responsive to cephalosporins for example are not any longer and as opposed to last year. But now carbapenems are available in the market and in TASH pharmacies so I have switched to carbapenems. But this isn’t a completely medically justified decision because only single clinical criteria is used as a factor to push this decision forward and culture and sensitivity isn’t always initially or even after therapeutic failure (48 to 72 has of no response) rather a switch of antibiotics is carried out. The rate of response is changing like cephalosporins are not responding so we are prescribing carbapenems although they are very expensive. But the problem is we do this without culture and sensitivity evidences. ***We don’t sent culture and I think we think of the cost of the laboratory for resistance.***

**Interviewer:** Ok, the culture of sending culture for microbiology test is not common and you were telling me that cost of the test is one factor, what other factors are there?

**Respondent:** one it is cost and the other is its consistency. You are not sure whether it will be performed or not. But before we sent many samples and they don’t respond if they do they bring it after the patient is discharged or died.

**Interviewer:** Is there a complaint in quality of the lab result?

**Respondent:** last year in ICU a bronchoalveolar lavage sample was sent to lab and the result indicated that the patient was resistant to all antibiotics but sensitive to CAF. So, the discussion in the morning was there might be error or problem of quality. There is still there is fear on the quality of the findings.

**Interviewer:** What factors do you think are contributing to increasing emergence and spread of antimicrobial resistance?

**Respondent:** The first contributing factor for the emergence of this problem is ***low staff knowledge and ignorance of issue even by those who are aware.*** You know physicians are ***prescribing from what they have thought in UG class which is mostly based on western countries recommendation or expert opinion*** which may not be always true and there is no system to update that knowledge. I can say our treatment is not supported by strong evidences. Second, even they have knowledge, attitude is very important. The other is the ***availability* and *cost of the supplies*** while the third is ***delay in obtaining preliminary laboratory results.*** If you ask when will be the delivery of preliminary result is not well known and this indicates a huge gap among us.

**Interviewer:** How do you describe the utilization of laboratory findings in the diagnosis of infectious etiologies? How do you describe the culture of sending specimen samples and the way findings are communicated?

**Respondent: *Not very frequently. The reason why we are so hesitant to send culture is because they are not usually (consistently) done in TASH and they would be too costly to require that the patients have them one from the private sector and brought in for consult.***

**Interviewer:** Do you have any complaints on the laboratory service? Please help us understand those concerns.

**Respondent:** There is lack of trust in the laboratory findings.

**Interviewer:** Do you think enough is done to curb the problem and what further action do you suggest? What possible measures do you recommend for better antimicrobial resistance containment practices to be implemented in this facility?

**Respondent:** for example ***infection precaution is not appropriately implemented*** within the hospital starting from very minor practice will be hand washing to more complicated details like maintaining ICU sterility at all times or fixing roof leakage in hospital rooms. But it isn’t the administration that is ***lacking appropriate implementation skill it*** is also individual staff members that do not use what have been made available for them in order to bottle infection spread. So many recommendation would be to hold continuous refreshment training programs so that and attitude change is influenced on staff members. ***The best thing is, implementing such programs is multiplicative by itself as it is a teaching institution.***

**Interviewer:** How would you feel if antimicrobial stewardship program [description of restriction policy and prospective audit and feedback intervention was given] is applied in this institution?

**Respondent: *I think everybody would love the implementation of audit and feedback intervention b/c it is everybody’s concern and as for your question of threat to autonomy by prescribes, I do not think that it would be an issue because*** these days the integration among specialist [saying that we need ID physicians recommendation is highly growing among physician] is encouraged rather than their autonomies practice. ***These is about supporting your practice by evidence and everybody would love to have.* *Restriction of prescribing authority could also work very nicely*** if the authorizing physician [highest prescribing rate] is assigned to be the on call consultant in the ward because as a fellow resident I can say that no other resident would have a problem would having go to a consultant for supervision but I think that it would be very difficult if the highest prescriber rank were to be assigned to sub-specialists like ID physicians*.* ***So restriction would work well if it is within specialty but if the authorization is given to other specialties, I doubt in its effectiveness and probably it could negatively affect the patient care.*** So, to implement the restriction policy, there should be discussion among prescribers to reach in consensus. ***From resident side they would be willing to follow b/c we are still under supervision, but if you apply the same to consultants, I am not sure about that.***

**Interviewer:** If these insertions are to be applied, what barriers and enablers should we anticipate?

**Respondent:** One facilitator as mentioned earlier is the ***supportive attitude of the residents (the physicians In general*)** whereas challenging factors could be in ***accessibility of the professionals assigned to consult for prospective audit and feedback intervention, uniformity issue among ward acceptance on the type of drugs with restrictions ward agreement and hence and an inconsistency in practice of this approach.*** So in order to avoid different treatment approach as a patient is transferred between the different wards we might have to focus on linking the practice of department that work with close ties with one another in treating the same patient. For example internal medicine and the emergency ward often work together, so implementation of this approach would have to necessarily be implemented consistently among the two. But pediatrics can be managed separately. With infectious disease until, the pharmacy and microbiology unit having the upper hand in running the program and the wards are in agreement in light of this new approach.

**Interviewer:** That is the end of my interview, is there anything you would like to add?

**Respondent: *My only suggestion would be to give regards to training and creating awareness very frequently with the continually changing prescribing staff in TASH*** since TASH is an academic institution and it’s always letting go of the prescribing student staff and accepting new students to practice. Upon graduation (interns and residents).***This will have a very vast influence nationwide because the students will reach almost every corner of the country after graduation.***

**Interviewer:** Thank you a lot.

=================================================================================

## Physician KI#03

Age: 36 years

Sex: Female

Department: Surgery [Pediatric]

Staff Position: Consultant Physician

Total work experience: 12 years

Number of patients treated per day: 2 patients

Number of patients with ABX prescription per day: 2 patients

Duration of interview (minutes): 23:34 Minutes

**Interviewer:** Ok thank you, my question focuses on three major themes. So to start with the first, how do you describe the current use of antibiotics in this institution or even at country level?

**Respondent:** It depends. ***Management of patient of antibiotics use isn’t evidence based, it is rather a personally guided clinical presentation oriented decision. So, usually there is a decision to go with a broad spectrum antimicrobial to avoid having to wait longer time for test results*** from the laboratory especially if the patients are at critical condition or to go for narrow oral antibiotics in an attempt to avoid resistance development if we think the patient is relatively stable. ***It is a more personal preference guided practice but it is not evidence based practice.***

**Interviewer:** Do you think inappropriate use of antibiotics is a problem? Which problems are more prevalent and Why?

**Respondent: *I do believe that there is overuse of antibiotics. For prophylaxis we usually use broad spectrum and for the post op usually guidelines recommend one or two doses but we tend to prolong duration of antibiotics prescription even up to seven days for fear that there could be infection complications post operative procedures despite what treatment guidelines recommend. So yes there is an extremely high overuse of broad spectrum antibiotics.***

**Interviewer:** How do you describe patients’ pressure to prescribe antibiotics? What is your personal experience in this regard?

**Respondent: it is not common.** But patients post operation demand medication use to control or better suppress their pain based on past experience with certain antibiotics and manage to obtain it from outside the hospital even with previously used old prescription. So, there are some community pharmacies who sold antibiotics with old prescription or even without prescription. ***Some also demand injection medications but we won’t be swayed unless we believe it is necessary ourselves.* *There were times when the patient insisted so hard, we decided to give them analgesic injections.***

**Interviewer:** How do you describe the current status of antimicrobial resistance in our hospital and at national level?

**Answer:  *Resistance is indeed a problem that we force in practice and I believe it is an increasing one too.*** We don’t usually have culture results but there were a time where received a report finding showing that the patient was resistant to all antibiotics. Although not common to use culture, from our personal judgement when frequently see failure of responses and we usually assume it is mostly due to resistance, so we see high resistance pattern especially ***urology patients***.

**Interviewer**: Which types of pathogens/infections do you think have more resistance profile? How it is affecting your daily practices?

**Response:** E.coli is what I would say is the most resistant pathogen. Resistance prolongs post-operation hospital stay, it could prolong it from the recommended 3-5 days up to a period of 3-4 weeks, it also affects the admission and surgical intervention we take. It also results in additions cost for treatment.

**Interviewer**: In which samples do you see a lot more reports of antibiotic resistance (urine, blood, sputum, pus or other body fluids)?

**Response:** Samples with this issue are urologic, hematologic samples also have this profile.

**Interviewer**: What factors do you think are contributing to increasing emergence and spread of antimicrobial resistance? Please explain on how commonly each of them exist in your hospital?

**Response: *Overuse and misuse of antibiotics*** *as well as* ***increase in cross contamination contribute to increase emergency*** *and spread of resistance****.* *Infection prevention activities*** [resulted in high cross contamination] aren’t well developed within the hospital. Though there is improvement nowadays, it still hasn’t reached the standard quality of practice. There is also the issue of ***adherence*** especially in pediatric patients.

**Interviewer**: How do you describe the utilization of laboratory findings in the diagnosis of infectious etiologies? Are microbiology lab results timely communicated to the treating physician in this institution?

**Response:** Microbiology laboratory results take a minimum of about 72 hours or more to reach us back. But you know the patient is already highly infected and you should start is immediately. ***So, delay of lab findings is common that discouraged us from sending specimens for culture.***

**Interviewer**: How frequently do treating physician request for cultures of relevant clinical samples to guide his/her choice of antibiotics? If less frequent, why?

**Response: *So in an attempt to avoid a delay initiation of therapy we are forced to start treatment without getting culture and sensitivity results back or maybe not even sending them. Physicians tend to request these tests only after therapeutic failure with initially started antibiotics.***

**Interviewer**: Do you have any complaints on the laboratory service? Please help us understand those concerns.

**Answer: *Delay*** of result is one. Another thing is, t*here* ***are usually not many cultures sensitivity test requested because there are only a few common antibiotics covered in the test and we often feel discouraged because the antibiotics of our interest aren’t checked for culture and sensitivity.*** So our prescribing will be totally swayed to the antibiotics on which the test were conducted and not on our original preferences. ***Doubts about quality of the results obtained for the laboratory really isn’t an issue for us.***

**Interviewer**: Do you have concern in the way antibiotics are prescribed and used today? Do you think you can impact the problems of antibiotic use? If yes, in what ways?

**Response:** I do not think that there is anything being done in the surgery department but at the hospital level in general culture sensitivities are being conducted on surgical site infection and hospital acquired infections and we are readily volunteering to help provide samples from our patients. But we are discussing on implementing a new protocol to standardize the prescribing pattern in our department. **Interviewer**: Ok, you are telling me the development of protocol is on the way as a solution but what other possible measures do you recommend to be implemented in this facility?

**Response:** Management protocol is essential, second patient focused culture and sensitivity testing especially for the common infections has to be mandatory and it has to be timely as well. Infection prevention should also be improved (like cross contamination minimized).

**Interviewer**: From your experience, can you think of potential factors that might influence the antimicrobial resistance containment practice in the hospital?

**Response:** In my opinion it would be best to have ID physicians run the infection control in all wards to gain better acceptance by the rest of the staff members. Yes, there might be hospital factor or patients factor where they may not afford it.

**Interviewer**: How do you see if antimicrobial stewardship [full description of the prospective audit and feedback intervention; and restriction policy was given by the interviewer] is implemented in this hospital?

**Response:** Prospective audit and feedback, ***I think would be more acceptable if the actors of choice are the consultants, not specific department.*** But it still is an integrative team work requiring the involvement of more than one responsible personnel from each department so it should involve a team of representatives from all wards or at least a minimum of five wards. With regard to the restriction, it can work but we have to take care of its implementation as each department could have their own protocols. I mean there might be disagreement, if the authorization is give to one specific department. So would be good if this things are discussed prior to its implementation. ***There were a time, three to four years when I was a resident, where carbapenem was restricted to few physicians but that was due to shortage of the drug. Although the reason was due to shortage, that experience showed that restriction can work.***

**Interviewer**: Can you help us understand the potential factors that might influence the implementation of prospective audit and feedback in this institution? I mean what barriers and enabling factors do you see to implement?

**Response:** I do not believe that there are many challenges that this stewardship program will face. I believe that it will promote the teamwork spirit amongst the healthcare professionals and open up a discussion forum to better the health care provision. ***I feel that it would be better for seniors from every department to be the senior consultants on the prospective audit and feedback intervention so as not to disturb the hierarchy in place and for better feasibility of the program.*** I think that advising seniors might become difficult to practice for the juniors and hard to accept for the seniors i.e. *if you make the juniors to make the member of the team, there would be resistance to accept by the senior so I think it is better to make the senior be the leading in order not to disturb the hierarchy.* ***If you make teams from the same department with the same specialty it might be challenge but if it is led by ID physician, it won’t be a problem.*** I also think that the ***recognition that surgical site infections are much higher than they should be is attracting attention to the problem*** at hand and will help encourage culture and sensitivity testing as well as the use specific antibiotics only to control drug resistance. ***Because at the end of the day it’s in every health care professionals interest to contain this problem***. It’s also my recommendation that we conduct studies on outcomes of treatments when both the curative and prophylactic treatment durations are prolonged as is done in current practice and when shortened as per the guideline recommendations to help us realize the impact of treatment duration on both the emergence of antibiotic resistance and the risk for window infection development. I think, if you are encouraging ASP, it is very good idea and I am a pro of this program b/c it will encourage the poor team work we have where every professionals are working on their own box where there is boundary among profession. This can be considered as initiation to improve to working in team.

**Interviewer**: That is the end of my interview, is there anything you would like to add?

**Response:** I think that we would have to consider supplying smaller doses of medications since 40% of the patient population here in TASH is pediatric population and dividing large doses that are available in the hospital pharmacies for pediatric use is a risk factor for developing resistance so I would like for you to give regards to this problem as well.

**Interviewer**: Thank you.

=================================================================================

## Physician KI#04

Age: 36 **years**

Sex: **Male**

Department: **Pediatrics**

Staff Position: **Consultant Physicians**

**Interviewer:** How do you describe the current use of antibiotics in Ethiopia and particularly in this institution?

**Respondent: The antibiotic use in private institution and in public institution especially in teaching hospital is different. They also use different guideline. The antibiotic use is inappropriate. The patients that doesn’t require antibiotic might be given one, there is over prescription and also inappropriate antibiotic prescription. Most prescribe are 2^nd^ line antibiotics especially in private set up.**

**Interviewer:** Do you think inappropriate use of antibiotics is a problem in Ethiopia and in this institution? Which problems are more prevalent or common in this setting? Why? How do you describe the pattern of this problem over time (Is it increasing, decreasing or stable)? Why?

**Respondent: At this hospital the antibiotics use is improving through time. There is antibiotic stewardship, regular lectures & seminars on antibiotic use that has been contributing to the improvement. But still limited availability of antibiotics, poor culture sensitivity result negatively influences the appropriate choice of medication (antibiotics). The availability of antibiotics is better than before. Now, there are fourth generation Cephalosporin, Carbopenem, Vancomycine, Meropenem ready available. Cefipim may not be available at all time.**

**Interviewer:** How do you compare the use of broad vs. narrow spectrum antibiotics? Do prescribers tend to prescribe very broad-spectrum antibiotics empirically and/or to over prescribe antibiotics? What are the potential reasons for the preference of broad spectrum antibiotics? What factors do contribute for the overuse of antibiotics?

**Respondent: Potential reasons for preference of broad spectrum antibiotics include physician’s lack of knowledge, absence of regular lecture or seminars on creating awareness of rational antibiotics use, absence of a known culture sensitivity profile, usually the culture result is negative. If culture sensitivity test result is negative but the clinical presentations are present empiric treatment is used. Fever per say presence may not always indicate infection and so rather than investigating more on it antibiotic is given. And if the fever persists there change of antibiotics.**

**Interviewer:** How do you describe patients’ pressure to prescribe antibiotics?

**Respondent: Other than cost issue patient influence is not that common. When you think of fourth generation cephalosporin or carbapenems there is cost issue which restricts the prescription. There is also community influence some patient prefer injection but generally for hemodynamically stable patient PO is the safest way of administration specially for antibiotics. For critical patient IV antibiotics is given and when the patient’s condition improves it’s changed to PO & discharged from the hospital and this is a good thing because one bed occupancy decreases, there is psychological issue specially children prefer to be home and take their medication rather than stay in the hospital, repeated IV tube , catheter, NG tube exposes the patient to nosocomial infections.**

**Interviewer:** How do you describe the current status of antimicrobial resistance (at institutional and national level)? Should healthcare professionals be worried of this problem? How serious is the problem? Do you think it is a growing public health treat or not?

**Respondent: This antibiotics resistance profile is increasing. Rational use of antibiotic is not practiced there is over prescription. Example cephalasporine, ceftriaxone almost all patients that comes to the hospital is injected, its over used. There is cross reactivity with penicillin.**

**Interviewer:** How frequently do you encounter drug resistance organisms within your clinical practice? Which are common?

**Respondent: Most bacteria that grew are gram negative like clapsella pneumonia, E.coil stephorius. Clepsella pneumonia is resistant to most of the antibiotics. Methicillin resistance stephorius is not common as said most of them are sensitive to cloxacillin. The gram negative like clepsella pneumonia are developing resistance.**

**There is blood culture and body discharges sample taken and it’s hard to say which antibiotic resistance is reported from. On daily practice, if the resistance development continue like this it is difficult. We have limited type of antibiotics not in our setup but in developing country as a whole. Specially oncology patient that have feverial neutropenia if cefipim, merepinum, pepirazine ,tazobactame develop resistance there is not much choice moropinem is used as the last resort here but there are some strains that has already developed resistance to it. For immune compromised & neutropenia patients the problem is worse.**

**Interviewer:** What factors do you think are contributing to increasing emergence and spread of antimicrobial resistance? Please explain on how commonly each of them exists in your hospital?

**Respondent: getting positive blood culture result is challenging patient having clear infection sepsis bacterium clinical indication with a lot of issue the culture result may turns out to be negative also those patients having blood culture result positive usually are resistant.**

- **Hospital stay duration being long is another factor so IV antibiotics should be changed to PO. Antibiotic is not prescribed to right diagnosis giving antibiotics to patient having mere fever.**
- **Infection prevention is really poor on oncologic department patients with hematologic malignancy their absolute neutrophil count is <500 and they require meticulous infection prevention.at this institution alcohol is used for hand rub most common ways of transmit ion is physicians’ hand from one patient to another patient.**
- **Multi dispensary practice is a good way to minimize the problem. If clinical pharmacist round with the physicians there in put for the choice of antibiotics, the dose the available drugs update is helpful also if the microbiologist joins too and update on blood culture grew**

**Interviewer:** How do you describe the utilization of laboratory findings in the diagnosis of infectious etiologies?

**Respondent: Sample are sent to the laboratory but they don’t do gram stain which is fast procedure. What they do is after they incubate in the dish if the bacteria grow, then they do gram stain. The gram stain may not tell us the sensitivity but it will identify the etiology (gram negative or positive, diplococci or in cluster) it’s important for decision making also it’s fast procedure. Example meningitis suspect after turbid CSF sample sent gram stain procedure is ideal for choice of antibiotics culture takes 5 to 10 days urine culture is the fastest collected which takes 3 days.**

- **Mistakes could occur anywhere. It could be from microbiology or from the physician investigation result. If the culture result highly deviates from the physician, he communicates the microbiologist and ask to repeat the test There are other complains on microbiology laboratory like losing samples, they don’t do gram stain, culture results are not collected on time.**

**Interviewer:** What possible measures do you recommend for better antimicrobial resistance containment practices to be implemented in this facility? Please relate your recommendations based on its importance. **Respondent: Creating awareness on rational use of antibiotics on every personnel that are involved in the management of a patient. Local resistant pattern should be identified and made known, there should be clear guideline.**

- **Antibiotic restriction is not a good way to overcome every physician has a right to prescribe as he sees fit creating awareness is much better way to handle the situation. For instance, on emergency cases quick measures should be taken but going through all the process, in the meantime the patient may die.**

**Interviewer:** From your experience, can you think of potential factors that might influence the antimicrobial resistance containment practice in the hospital?

**Respondent: Autonomy which is every physician has the write to prescribe & availability of physician (senior) when ever needed are some of the factors that might influence the antimicrobial resistance containment practice regards to implementation of antibiotic restriction.**

**Interviewer:** How would you feel if prospective audit and feedback is applied in this institution and a multidisciplinary team review and give you feedbacks on your prescribing? Can you help us understand the potential factors that might influence the implementation of prospective audit and feedback in this institution? How do these factors become barriers?

**Respondent: Antibiotic stewardship team established is acceptable the physician may have some knowledge on microbiology some knowledge on medication. But the pharmacist is the right person to know the mechanism of action of an antibiotic. The presence of pharmacist in the team could update on which medications are available the dose. Challenges will exit until the program is well practiced but considering the center of discussion which is the patient and focusing on it will overcome the challenge.**

- **The discussion shouldn’t take time on commenting and the team should be available on time at the right place. Human resource allocation is factor in the process but may health professionals be graduating so on problem on human resource**

**Thank you for your time!!!**

=================================================================================

## Physician KI#05

Sex: **Female**

Department: **Pediatrics**

Staff Position: **Consultant Physicians**

**Interviewer:** How do you describe the current use of antibiotics in Ethiopia and particularly in this institution?

**Respondent:** Rational use of medication antibiotic are two ways one Is infliction caused by germs if not treated on time and appropriately it may in danger life, there is inappropriate use of medication limited and expensive antibiotic use in the community antibiotic resistance could be developed.

**Interviewer:** Do you think inappropriate use of antibiotics is a problem in Ethiopia and in this institution? Which problems are more prevalent or common in this setting? Why? How do you describe the pattern of this problem over time (Is it increasing, decreasing or stable)? Why?

**Respondent:** The antibiotic use is dividing into two one in private practice and other is in governmental hospital specially teaching hospitals practice. There is a clear difference between those two. At private sectors fear of losing customer if the patient (customer) doesn’t improve immediately which is unethical fear is observed so they use broad spectrum antibiotic or those antibiotics that should have been given after resistance has developed are given at first line. Fear of losing a patient’s life because the antibiotic used/given may not work this also leads to use of broad-spectrum antibiotic.

**Interviewer:** How do you compare the use of broad vs. narrow spectrum antibiotics? Do prescribers tend to prescribe very broad-spectrum antibiotics empirically and/or to over prescribe antibiotics? What are the potential reasons for the preference of broad spectrum antibiotics? What factors do contribute for the overuse of antibiotics?

**Respondent:** Other is poor culture sensitivity test. The quality is low and also the differentiation result is minimum, it doesn’t have wide coverage of bacteria, because of limited media covers where the bacteria grows on. Comparing to other country, our culture and sensitivity yield is minimum. So broad spectrum antibiotics is given empirically.

**Interviewer:** How do you describe patients’ pressure to prescribe antibiotics?

**Respondent:** I don’t agree on patient pressure, it is up to us to decide. Its not patient pressure but our lack of knowledge that is pushing towards them.

**Interviewer:** How do you describe the current status of antimicrobial resistance (at institutional and national level)? Should healthcare professionals be worried of this problem? How serious is the problem? Do you think it is a growing public health treat or not?

**Respondent:** This institution is wide area and there is a difference on antibiotic resistance development in every department like oncogene pediatrics. Penicillin resistance though has developed everywhere methicillin sensitivity resistance, methicillin resistance, Beta-lactamase resistance extended ones our last resort antibiotic like carbapenem resistance (imipenem, meropenem) resistance development has been seen. The extended spectrum beta lactamase resistance and Carbapenemase resistance i.e. Imipenem, Meropenem. Is highly used regular at this unit form clinical response point of view. In other countries like India antibiotic use is right and, it’s irrational. So, their major problem is resistance development. The major cause of death in cancer patients is antibiotic resistance presence which no response can be observed. In our country new antibiotic, not begin purchased, have positive impact on limitation of antibiotic resistance development. Resistance pattern is high in the hospital than in community area. Now a day’s those new antibiotics are begin purchased by different methods and the community’s ability to buy has increased. So resistance development is increasing.

**Interviewer:** How frequently do you encounter drug resistance organisms within your clinical practice? Do you face any challenge in your daily practice as a result of antimicrobial resistance?

**Respondent:** Within the last 6 months 3 carbapenemase resistance where reported by klebsiella pneumonia. Major cause of death of cancer is inflection they are immune suppressed. If the infection site is exposed to this resistance 3^rd^ line Carbapenem is used. It could be right on the beginning. Inflection treatment for cancer patients is empirical which bacteria cause the infection so broad coverage is required. The infection in cancer patients is usually caused by more than on bacteria (poly microbial). Even if only a single bacterium is detected, antibiotic with broad coverage is required to be given. Main (common) problem is fever & neutropenia so irrespective of culture and sensitivity finding antibiotic is given. In pediatric oncology, cancer outcome improvement is attributed to the wide availability of antibiotics & it good quality. In fact in certain patients if the prescribed antibiotic is not available in the country there won’t be future treatment. Example in acute myeloid leukemia even if they 50% of survival chance the antibiotics required are not available & even if they are available they are very expensive.

**Interviewer:** What factors do you think are contributing to increasing emergence and spread of antimicrobial resistance? Please explain on how commonly each of them exists in your hospital?

**Respondent:** From experience, there is high security on which medications are allowed to be registered in the country. Antibiotic that are used for highly resistance bacteria are not allowed or they are restricted to be registered. If there is no limitation on antibiotics the community would have been exposed. The culture and sensitivity’s poor differentiation ability is the other major factor.

**Interviewer:** How do you describe the utilization of laboratory findings in the diagnosis of infectious etiologies?

**Respondent:** Most of the times laboratory culture results are negative so when positive results are reported two things are considered one is if the culture result positive b/c of contaminants, So further investigation is done on the bacteria to see its’ present in the area where the sample was taken form. The investigation is more of suspension which is based on the type of the bacteria and the time it took to grow. If the bacteria aren’t commonly found in area where the sample was taken form definitely with no doubt the result is used for planning treatment process. In other countries like India antibiotic use is right and left it’s irrational the major problem is resistance development. The major cause of death in cancer patients is antibiotic resistance presence which no response can be observed. In our country new antibiotic, not begin purchased, have positive impact on limitation of antibiotic resistance development. Resistance pattern is high in the hospital than in community area. Now a day’s those new antibiotics are begin purchased by different methods and the community’s ability to buy has increased. So resistance development is increasing.

**Interviewer:** Do you have concern in the way antibiotics are prescribed and used today? Do you think you can impact the problems of antibiotic use? If yes, in what ways?

**Respondent:** At TASH in oncology department there is organized infection unit. Almost every patient is taking an antibiotic b/c infection prevention is not optimal so the patients are exposed. Even the normal flora can cause infection in this patient. Hospitals stay induces this infliction and they manifest febrile neutropenia and antibiotic should start within one hour if not, death rate doubles. Antibiotics are given irrespective of culture result so wide use of antibiotic is present. There is a study being done by the inflection team an oncology patient that is going to be published soon on hospital antibiotics resistance development pattern. Lots of positive result seen but also shows the increasing resistance pattern. There is also a plan on conducting a study in community antibiotics resistant development pattern their resistant pattern is done before admission to hospital. The limitation on registration of new drugs has protected the community in general form increment of drug resistance. But when we see it at individual level delay on registration has negative effect.

**Interviewer:** Do you feel regular auditing have an impact on prescribers’ behavior? How would you feel if prospective audit and feedback is applied in this institution and a multidisciplinary team review and give you feedbacks on your prescribing? Can you help us understand the potential factors that might influence the implementation of prospective audit and feedback in this institution? How do these factors become barriers?

**Respondent:** Generally, at TASH there is team decision. The decision maker is the senior physician. The interns make suggestion. Magnates if not on timely treated it because growth retardation on children even here 48 hr. check in stool penicillin is give & chloramphenicol or ceftriaxone. As long as new studies emerged and show resistance the treatment will continue like this. But for seven meningitis 2^nd^ line or greater may be given.

For TASH involving pharmacists & ID specialist in auditing and feedback intervention is not difficult but in other sector it’s difficult because of human resource.

Protocol should be stated by the agreement of team members. ICU team, oncology team… should be organized b/c it is where antibiotics are prescribed right & left. The team should always be aware of the hospital’s antibiotics resistance pattern. Currently though it is empirical decision and individualized.

Thank you for your time!!!

=================================================================================

## Physician KI#06

Age: 63 years

Sex: Male

Department: Pediatric Ward

Staff Position: Consultant Physician

Total work experience: 44 years

Number of patients treated per day: 20 patients

Number of patients with ABX prescription per day: 7 patients

**Interviewer:** thank you so much. We aimed to understand the status of antibiotic use, resistance pattern and barriers and enablers to implementation of antimicrobial stewardship. So start with, how do you describe the current use of antibiotics in Ethiopia and particularly in this institution?

**Respondent:** What is the word appropriate; it is circumstantial, b/c you should look the reality otherwise if you want to apply the principle, the setup does not allow you. Where the culture is dead and two antibiotics disc use are applied in this hospital, I do not expect the right use of antibiotics. The appropriate use of antibiotics is not correct rather it is almost zero. ***Where there is no culture, it is useless. We simply apply our experience***.

**Interviewer:** Thus this leads to irrational use of drugs?

**Respondent:** Of course, yes. B/c when we say rational use, we consider so many things; the first is prescribing the right antibiotic to the right organism. I could not know the right organism in our situation (our hospital). Therefore this is the reason I said the matter requires objective reality. Unless the facility is equipped, it is useless to apply the scientific method. We use our empirical experience and by looking at literatures. So to say appropriate use, we need to see the existing background/context but otherwise we know that our use of antibiotics is not based on practical and institution specific evidence.

**Interviewer:** Are there other reasons which enforce you to do so I mean in addition to issues related the poor laboratory infrastructure?

**Respondent:** ***Yes, availability of the antibiotic (drug) is very important. Even is the result from laboratory is at hand, there is chronic shortage of antibiotics. Shortage of antibiotics is another big challenge.*** Therefore there is a problem in the diagnosis and availability of drugs. The availability is two types, one in the private health institutions it is good but in public institutions it is not affordable. Even if the medicines are available in the market, it is very expensive I mean practically it is unavailable for the poor.

**Interviewer:** How do you see the resistance of antibiotics from time to time?

**Respondent:** It is increasing from time to time, there is no question to that. There is one fact that we should take care of; continuous surveillance must be done about culture and sensitivity in one hospital. Probably the data we are using are very old; there is no up to date data. We don’t have data but if it is done then dissemination is also poor. In general we don’t have data, but from practice we can see patients are not responding to cocktail of 3 to 4 antibiotics and even rarely when we receive culture and sensitivity result it showed they are resistance to 3 to 4 antibiotics.

**Interviewer:** What do you think are the prominent contributing factors for antibiotic resistance?

**Respondent:** The basic is lack appropriate utilization of antibiotics. For this we should teach and it must be changed into practice. For instance we are applying it in our department (pediatrics). However in general when we see in the town, it is rampant; everybody is prescribing. When new antibiotic comes, there is high tendency to use antibiotics quickly. Therefore, misuse of antibiotics is rampant and the resistance pattern is increasing. ***We see referred patients from the lower health facilities, we see that they are already in the broad spectrum, they came after they already tried all choices of antibiotics and we have difficulty to treat those patients.***

**Interviewer:** so to solve the problems, what do you think should be done at department level or hospital level?

**Respondent:** At department level, infectious disease (ID) lead team should be in place. This should give guidelines, for instance about antibiotic pattern. The team should be strong. Another responsibility of the committee is infection prevention. Antibiotic pattern, infection prevention and data are responsibilities of the committee. The ID team must be well strengthened. If we have evidence, then we can say this drug is not good/good b/c it is resistant or sensitive to this pathogen….this can be helpful to guide all prescribers. ***Otherwise organisms are not stagnant they are moving every corner of the institution (from ward to OPD) like some in vacation. , organisms can’t say I am not going to affect pediatric but others, Hence, it is not the responsibility of one department or one person but should be coordinated among all departments and professionals.***

**Interviewer:** if there is an idea of implementing antimicrobial stewardship, do you think the ID lead antimicrobial team will be accepted and feasible here?

**Respondent:** ***Oh, yes. This should be based on mutual understanding not by letter from medical director or formulating a team, it does not work.*** For example if the medical director passes by letter it will not work. By rather by indicating the magnitude of the problem and inviting all the stakeholders and those who are concerned should discuss, agreed and recommend a solution. If all the facilities are fulfilled the ID team will be effective. ***Concerted effort by including physicians, pharmacists, laboratory/microbiologist is very important. It works in other place, why not here. Let me told you my experience, when I was a medical student I remember that cotrimoxazole was a big drug [exclamation] by that time it was only allowed if the medical director’s puts his signature. That doesn’t work, I mean do you expect him to sign the whole day? That doesn’t work, it fails in short time. So, it shouldn’t not individual’s work but should be made in consensus/agreement.***

**Interviewer:** As you said, the laboratory setup is not good, is one indicator but do you think there is another barrier?

**Respondent:** After this all, they should bring to the department level for discussion. The departments should discuss and implement it. It is as I told you earlier. For instance if pediatrics department accepted and implemented it but Gynecology department does not accept and implement; it will not work b/c we are in one hospital. ***B/c there is interdepartmental transition of micro-organisms.*** ***Therefore if it is going to be effective if all of the departments should speak the same language. This could be effective if there is a strong committee armed with strong evidences.***

The rate of resistance of antibiotic is alarming. But we are poor. ***I, you and your families may face a problem of shortage of antibiotics. We should think to ourselves.*** ***So since it the problem we are facing all, no one will not resist it, this is my opinion. Any department will accept it and will not resist. There might be bureaucracy and this could be solved together. In principle, we are crying and striving for better intervention and I do not think there will be a problem.***

**Interviewer:** Thus, the ID team could be successful by that but do you think restriction of antibiotics will have a problem?

**Respondent:** Yes. Do you know why if the team has agreed reached consensus they should decide together. For example one department (internal medicine) may say we should use this antibiotic, another department may say no***. We agree on science, I mean the game is not on words but on scientific evidences. Medicine is evidence based science. We should agree based on science. We can argue on science, literature and finding. If there is an individual who do not believe on science this is arrogance, this is disease which is difficult to treat. B/c we believe on science and should be do based on it.***

**Interviewer:** Thanks a lot and this is what would like to discuss with you but you can help me understand the issue if there is something I forget to discuss.

**Respondent:** *Well that is good, I don’t have for now.*

**Interviewer**: *Thanks.*

=================================================================================

## Physician KI#07

Age: 44 years

Sex: Male

Department: Pediatric Ward

Staff Position: Consultant Physician

Total work experience: 28 years

Number of patients treated per day: 15 patients

Number of patients with ABX prescription per day: 5 patients

Duration of interview (minutes): 15:00 Minutes

**Interviewer:** Ok Thank you! So to start with my first question, how do you describe the current use of antibiotics in Ethiopia and particularly in this institution?

**Respondent:** When we see the use of antibiotic in our hospital, first our hospital is tertiary hospital, the cases which are coming to our hospital are more difficult cases and the patients are /were assessed by other physicians [by residents and interns] as I am working as consultant physician which different practice from other hospitals. From my experience in private and previous experiences***, I can* see there is misuse of antibiotics. There are unscientific use antibiotics. I observe three or more antibiotics are prescribed together.** If you ask me what is the risk of this, first the patient will be ***exposed to unnecessary side effect, second there is an issue of cost related to family and country level, third antibiotic resistance.*** We can also consider time is consuming [longer hospital stay].

**Interviewer:** How about using broad spectrum antibiotics? How do you describe the practice of physicians in this regard?

**Respondent:** Most of the time, we use broad spectrum antibiotics. We use narrow spectrum if it is culture proven or if the causative agent is known to be effective to specific antibiotics-this is from our clinical knowledge. But that is not the case, the tendency of using broad spectrum is very high. However, we also see that there is quality problem/ and the yield of culture and sensitivity is not satisfactory and that should be improved.

**Interviewer:** OK, so you saying absence of culture and sensitivity contributes to resistance. What do you think is the reason for overuse of antibiotics?

**Respondent:** As I said before, the cases coming to our hospital are complicated or critical or they already tried with more potent antibiotics or are chronic. ***As the physician is the end hope for the patient and the patients are critical or subcritical; patients are not stable, the physician do not want to miss anything, these leads to overuse.***

**Interviewer:** We discussed about the problem and shortage of laboratory, are there other complaints from physicians, I mean about the laboratory?

**Respondent:** Yes, there is. I want to speak the most important complaint, it is about lumbar puncture (CSF gram stain). If CSF gram stain result is reached on time, it will help us to use narrow spectrum for example meningitis patient instead of treating blindly we can treat them using specific antibiotics. **However there is a delay and the worst thing is there were times where the sample got lost. It has been repeatedly reported as lost, very difficult issue.**

**Interviewer:** How do you describe patients’ pressure to prescribe antibiotics?

**Respondent:** I can speak only about Black Lion in general, pediatrics department specifically and I can say there is no influence. But as first line or frontier physician at lower health facilities, the pressure of patients, family or caregivers is consider***able. As I observe a patient with common cold has been given a prescription for antibiotic.***

**Interviewer:** we discussed about antibiotic use and the contributing factors. How do you describe the antimicrobial resistance problem?

**Respondent:** From my personal practice or observation and from researches done in Black Lion, the antimicrobial resistance is at a very serious stage. It is increasing from time to time.

**Interviewer:** What do you think are the reasons for increasing AMR?

**Respondent:** Absence of rational use of antibiotic is the cause. Using antibiotic when it is necessary, narrow spectrum and appropriate duration must be considered when they are prescribed. In addition, the infection prevention practice should be improved and also strengthen team work and improve staff awareness. But as it is teaching institution, I don’t think knowledge is a problem. There are no problems related with knowledge. Since this is a teaching hospital there is a communication among the intern, resident and the physician. I cannot say there is no but it is not a major problem.

**Interviewer:** How about infection prevention?

**Respondent:** The infection prevention is not satisfactory. In some wards there is a good infection prevention practices but in some wards infection prevention (either there is no or it is substandard). It should be improved across all wards/departments.

**Interviewer:** If it is so, and resistance is increasing; do you think the work being done is satisfactory?

**Respondent:** It is not satisfactory

**Interviewer:** In your opinion, what should be done?

**Respondent:** ***First, research should be done to know the pattern of antibiotic resistance in our hospital. Second, awareness creation programs (lecture, seminar) should be prepared and discussed continuously. Both formal and informal discussion should be encouraged. And money should be allocated for the research and discussion. Since it is a critical issue, trained human resource should be assigned for the same.***

**Interviewer:** Do you think it will be accepted if antibiotic restriction policy [……After describing what restriction policy is…..]?

**Respondent:** May be there are potent antibiotics at the verge or risk of becoming extremely resistant (vancomycin, meropenem etc.), in such latest drugs, I accept some restriction should be implemented. But those first line antibiotics should be prescribed by low level health professionals since our population is in the rural and the number of physicians is low in number…I am not against that. But training should be given and infection prevention mechanism should be improved.

**Interviewer:** How about the high level, do you think it will be acceptable here in our hospital.

**Respondent:** Yes it will be accepted b/c there is an already established hierarchy.

**Interviewer:** Another antimicrobial stewardship intervention is prospective audit and feedback which is a multidisciplinary lead by ID physician [the interviewer give brief description what prospective audit and feedback is]. Do you think this type of intervention is acceptable and feasible?

**Respondent:** ***I think any educated person is not against feedback. We improve our practice not only by what we do or believe but by feedbacks we get from others.*** This is a good thing as long as it maintains proper channels of communication; as long as the aim is to improve the practice and to alleviate the antibiotic resistance in the given setup.

**Interviewer:** What barriers do you anticipate if these types of interventions are to be implemented?

**Respondent:** Right! there are barriers. For instance the physician may say this is my autonomy. First a physician may claim that s/he is certified to prescribe any antibiotics from simple to highly potent antibiotics but as I said earlier this could be solved by training and discussion.

**Interviewer:** But in addition to autonomy; do you see other barriers for instance seniority, availability, incentive.

**Respondent:** For instance if we say it should be prescribed by senior physician, where? It might work for black lion hospital, but if we do not change the practice in the country, it does not make sense. If we see the number of senior physicians in different hospitals, who is doing bulk of the job; it is by low level health professionals and general practitioners. From this point, it might have barrier.

**Interviewer:** Thanks a lot for your time.

=================================================================================

## Physician KI#08

Age: 39 **years**

Sex: **Female**

Department: **Pediatrics**

Staff Position: **Consultant Physicians**

**Interviewer:** How do you describe the current use of antibiotics in Ethiopia and particularly in this institution?

**Respondent:** Antibiotics are highly used at this institution. Every professional the higher up (consultant, ID physicians) to low level (the interns) prescribe it. The prescription is not following the guideline or it’s not culture sensitivity based. Its western data empirically prescribed. Two to three antibiotics may be prescribed for a single patient, antibiotics given for long duration than appropriate. Physician’s perception on antibiotics is its safe with minimum side effect. Forexample when prescribing medication for cardiovascular disease special attention is given on its side effects, adverse effect but this attention lacked in antibiotics prescription. The idea physicians have is any patient who is admitted in the hospital have to take antibiotics. So like every patient in the ward takes antibiotics without restriction.

**Interviewer:** Do you think inappropriate use of antibiotics is a problem in Ethiopia and in this institution? Which problems are more prevalent or common in this setting? Why? How do you describe the pattern of this problem over time (Is it increasing, decreasing or stable)? Why?

**Respondent:** In children viral disease is common so since it’s hard to differentiate b/n bacterial & viral cause of infection antibiotic over use of may exist. Also, if per kilogram is measured incorrectly this may cause under or over use of antibiotics. IV administration for long duration when it could be substituted by PO is another problem seen in this institution.

**Interviewer:** How do you compare the use of broad vs. narrow spectrum antibiotics? What are the potential reasons for the preference?

**Respondent:** When you compare the private institutions to the (public institution) the governmental hospitals (public institution) has beater use of antibiotics but still it’s not immune. One reason for this is not doing etiologic based treatment. When you say etiologic based specially in children being able to differentiate b/n bacteria and virus would avoid the antibiotics over use. Knowing which bacteria is sensitive to antibiotic. Microbiology result reported on time the use of broad spectrum antibiotics will decrease.

Being aware of sensitivity pattern also has positive impact. Microbiology laboratory is not strong well developed. This is due to lack of trust of physicians on microbiology results so they don’t send sample for culture even if they send the samples its after they give antibiotics so they get negative results, they don’t take samples to the micro biology at the right time. Micro biology laboratory factors one is human resource is minimum and most of the times there is no micro biology service at night times and weekend.so if a patient come on the weekend antibiotics is administered empirically and the culture is done on Monday. The problem is Training commitment, reagent, result delay due to communication gap.

Medications availability was problematic at TASH in the previous years. But currently rather than antibiotics availability problem affordability is a problem. As observed patient that stays long admitted in a hospital have the tendency to develop the hospital acquired resistant bacteria because of poor infection prevention. So broader spectrum or 3^rd^ generation, 4^th^ generation antibiotics may be required and most of them are expensive by then the patient may not afford it regular availability is another issue. Resistance over usage leads to especially to resistance gram negative presence & this limited the type of antibiotics to be used. So, you might not get antibiotic for MDR gram negative and the patient may die b/c of it.

**Interviewer:** How do you describe patients’ pressure to prescribe antibiotics?

**Respondent:** The patients after being admitted to TASH don’t really have an influence but before they get to the hospital they might take OTC antibiotics. At private institution the physicians prescribe very broad antibiotics, also for community acquired infection the patient may take cefepime & vancomycin. when the physicians prescribe combination of antibiotics or Piperazine & Vancomycin or Meropenem which are the last option drugs and when they get to the hospital it’s hard to choose the appropriate antibiotics.

The patients after being admitted to TASH don’t really have an influence but before they get to the hospital they might take OTC antibiotics. At private institution the physicians prescribe very broad antibiotics, also for community acquired infection the patient may take Cefepime & Vancomycin. when the physicians prescribe combination of antibiotics or Piperazine & Vancomycin or Meropenem which are the last option drugs and when they get to the hospital it’s hard to choose antibiotics.

**Interviewer:** How do you describe the current status of antimicrobial resistance (at institutional and national level)? Should healthcare professionals be worried of this problem? How serious is the problem? Do you think it is a growing public health treat or not?

**Respondent:** Antibiotics resistance is dangerously increasing. At this hospital in neonates we used to manage by using ampicillin & gentamycin well. At the ward meningitis crystalline penicillin with cough was our first line and we could see when the patients improve. But now prescribing those meditations and waiting for patient’s improvement is hard. This caused by overused of meditation. Poor infection prevention, patient’s taking antibiotics before admit ion. Since TASH is a referral hospital patient may be already in antibiotics, absence of restriction on patient & guardian / visitors’ interaction, health professionals (students, residents) together with the lack of infection management of the hospital has increase the infection rate. There are evidences showing the increment of the mortality rate secondary to MDR organism. The patient may get to critical condition or die on the process of obtaining result from culture. The imperilment in microbiology laboratory indicates the right cause of death.

**Interviewer:** How frequently do you encounter drug resistance organisms within your clinical practice?

**Respondent:** The sample which is seen to report more of antibiotics is blood sample and the pathogen is gram negative resistance to 3^rd^ generation cephalosporin, aminoglycoside. Casella & E-coil are most common organism that is isolated. From ward the oncology & ICU, neonatal ICU are most common areas for this kind of organisms.

**Interviewer:** What factors do you think are contributing to increasing emergence and spread of antimicrobial resistance? Please explain on how commonly each of them exists in your hospital?

**Respondent:** antibiotic stewardship implementation by clear guideline and strength the infection prevention at the hospital this two are major things that need attention and work on

**Interviewer:** What possible measures do you recommend for better antimicrobial resistance containment practices to be implemented in this facility?

**Respondent:** Antibiotic restriction is hard to achieve. You need personnel responsible. If antibiotics is restricted to be only prescribed by infection disease specialist, at a hospital where one infections disease specialist or none at all is not visible.

**Interviewer:** From your experience, can you think of potential factors that might influence the antimicrobial resistance containment practice in the hospital?

**Respondent:**

- Challenge for implementation of ID specialist let audit & feedback time, commitment & organizational support. The hospital has to accept & support the idea for implementation. One challenge contribute to the poor infection management is patient’s visitors come and go as they pleas this is allowed by the health minister of the country so the hospital management should see the problem with infection management and restrict visitors.
- So basically, strengthen infection privation committee has to be organized and policy and support from the health minister office and hospital management should be present.
- To overcome the challenges of time, commitment financial compensation is required or recognition has to be given. So generally financial or non-financial incentives given would be helpful for devotion.
- Enabling factor presence of work force health professionals is one. The other is there is a resource available but the problem is with management of resource. There is project on hospital associated infection that has been strengthening the microbiology and get good data. The availability of antibiotic has also increased.

**Interviewer:** How would you feel if prospective audit and feedback is applied in this institution and a multidisciplinary team review and give you feedbacks on your prescribing?

**Respondent:** Periodic audit and feedback implementation can work. If the micro biology laboratory is strengthened and every month updating comparative infection on which bacteria are found at the hospital the used antibiotics in the last month then giving suggestion on use of antibiotics for the coming month. In the future though when health professionals (especially ID specialists) number increase restricted antibiotics to the consultants or ID specialist is possible. Even on just restricting 4^th^ generation antibiotics the ID specialist should be available 24/7.

**Thank you for your time!!!**

=================================================================================

## Physician KI#09

Age: 27 years

Sex: Male

Staff Position: Resident (R3-Chief resident)

Department: Emergency Medicine

Work experience: 4 years

Number of patients treated per day: 50 patients

Number of patients with ABX prescription per day: 25 patients

Duration of interview (minutes): 36:10 Minutes

**Interviewer:** I have 3 major question. The first one is related to antibiotic use, the second one is related to antimicrobial resistance and the third one is continence strategy particularly on the antimicrobial stewardship program. So to start with. How do you describe the current use of antibiotics in Ethiopia and particularly in this institution?

**Respondent:** Our antibiotic use, more of this days, from what we are observing, it is difficult to say it is rational. It is difficult to say we are using it properly. Even when we see in emergency experience, when the patients arrive here they may come here after they start any/every antibiotic. That means, an antibiotic which is a very broad antibiotic or from the list, they may come here after they started a medicine among the top on the list. The problem is that, not only those patients started those medication but also, there might not be any test done. Those patients do not have tests done like sensitivity, blood culture and urine culture. Already the treatment has been started, so after this, if we do culture it will be much distorted. We could not find the correct thing. And the patients do not only come from home, and when they come here after starting the top level antibiotic, they come here after being admitted in another hospital. ***So the infection is not only acquired from the community, it is also acquired from the hospital.*** So sometimes the treatment started is for the hospital acquired or for the community infection. Or has it been shifted because the community acquired has not been resolved. We cannot differentiate all those. ***So I say the rational use is right antibiotic, right time, right dose, and right selection. So it is very difficult for me to say it is rational.*** Even not only for those who come, but also when we use or when we prescribe the antibiotic for them, sometimes it could be due to material shortage, it could be due to that. This days there are equipment’s for culture. But otherwise if those materials are not available, treatment will be started empirically, even a very top antibiotics. The patients are really sick, they are very critical. Or they could have malignancy. So in order to cover that, they will be started on a very wide spectrum. ***The culture will either be sent lately, or might not be sent at all. So the result will not arrive early. So before the result reaches, if the patient doesn’t respond the antibiotic will be changed.*** So when we see this, it’s not only for the diseases at that time on arrival but subsequently when this patients are admitted it will be difficult for selection. So I think it will be difficult.

**Interviewer:** So through time, as of your four years’ experience, how do you see the use of antibiotics over time? Is the problem of irrational use increasing, constant or decreasing as time to time?

**Respondent:** This days considering the burden of diseases, and from those patients admit how many of them are critical how many of them have a difficult diseases meaning since it is critical, it will be difficult. It is difficult to blame that the antibiotic use is irrational. It is also difficult to accept that it is also rational. So it is more of some kind of mix. So not to say it is rational, let us say as of my four years’ experience, when I see in my practice, in my first year experience ***ceftriaxone was the antibiotic that has been used right and left. So it is a broad spectrum, it covers and so on. But later on with in that year what has happened every one prescribes ceftriaxone. That means, private sectors even at a clinic level, started to prescribe. Later it happened to be, I think in a study in Black Lion a study involving an ICU patients, if I am not mistaken about figure the resistance reaches to 40 %.*** So while ceftriaxone as it is, has been used right and left, right and left, in a manner that is not rational, ***another antibiotics came. That covers a lot, like ceftazidime, cefepime, vancomycin. All of a sudden while they were available, currently this days commonly we used ones potent antibiotics like Peptazo and meropenem***; we reached there. so what it is, we do not have study which antibiotic have how much, for example I casually told you about ceftriaxone, but after that there was no a wide or a hospital wide study or any other study that shows the sensitivity. So without being based on that we moved to the other broad spectrum antibiotic. Without looking at the other studies. To that extent the diseases thing is also increasing. The patients, not only come here with an acquired infection, but they also have comorbidities and other illness, that means for example when they are neutropenic and will come with neutropenic fever. Or they could have malignancy and their immunity will be low. ***So we tend to make the antibiotic very wide to cover it. And sometimes it’s because of being concerned for the patient. Even if, we are concerned for the patient, due to shortage of material; and because of the time, since we are using it without culture or without any support,*** with in these four years, it is difficult for me to say that the rational has been increasing.

**Interviewer:** You have told me in relation with infrastructure, laboratory, but what about the influence related to medicine shortage so is there an influence. The over use or broad spectrum antibiotic. So does the availability of medicines has an influence or not. (00:06:24).

**Respondent:** Yes, this is really a great question. Because commonly antibiotics used after ceftriaxone are ceftazidime with vanco. For example if a patient develops hospital acquired infection we go to this antibiotics. Now when we use this antibiotic at that time, they are provided by the hospital and have a relatively cheaper prices. Later on when this antibiotics are not available at the hospital, the choice of the patent is to buy from a pharmacy outside the hospital. And buying from outside is very costly. They will differ in brand, the patients come from country side. They will sell everything they have. And at the end they will be very critical. So when we need antibiotic for this patients the patients may not find them. Because of the cost. And above the cost the availability is on and off in the country. So let us say a patent who has aspiration pneumonia who is taking ceftriaxone and metronidazole, if he doesn’t respond with that and if there is a new fever shoot and we still says its hospital acquired so our option to treat as a hospital acquired is by using ceftazadin and vanco. So while working, this really happen at this hospital, and at some point this antibiotic are not available. So when this antibiotic are not available, our option is to continue the antibiotic. And when we continue with this antibiotic, of course this patients is having a new fever shoot, we are not doing the culture and sensitivity, and the patients cannot afford to work up the laboratory investigation outside the hospital. So because of that, due to the availability of the medication in terms of the cost, in terms of the infrastructure, we will continue the one which is already available or with the one which is relatively good, if it is available, we will move to that one. As to my opinion the first reason that contributes for irrational use is the shortage of resource and the medicines, which is also one of the factors. Because the mediation that we think is suitable for the patient, if it is available at the hospital, and if the patient finds it with a reasonable cost, of course we will treat him with that. If it is not available in the hospital and if the patient cannot afford it our option is, we will use the medication that is available at the hospital with a cheaper cost. But, has a little help for the patient. So availability of the medication plus even if the medication is available our infrastructure also matters.

**Interviewer:** How do you see the patient influences? Like there is something seen in the private. But here in this hospital basically in government hospitals, we see in 2 sides. The 1^st^ one is the need of the patient to prescribe antibiotic or in the opposite side the fear of the patient’s when antibiotic is prescribed for them or for their child. So does this has an influence? (00:08:59)

**Respondent:** Of course in antibiotic it has an impact. Patient more often go to a nearby health facility or may prefer to go to private and soon. Here we cannot advocate any different thing that is; one is better than the other one. So as an emergency health care professional, if an emergency condition happens they may go to a nearby clinic; or initially go to health center then go to another hospital. But what we observe here is, in all the places they go the trend is, the sector that treats them at that time gives a treatment from his side instead of considering another thing. We are not observing those things. If it would have been that because this days the issue of antibiotic, we all should be concerned of. It is one of the concern of WHO. Antibiotic resistance is one of the big issue this days. So if one center prescribes an antibiotic, he should be reasonable. For example when we say reasonable, when he order a very wide antibiotic, there should be a reason. And if possible if he refers a case for example like here in BLH, if he keeps the reason; write the result of the test done; so the communication will be based on that. Otherwise, if one … 00:10:39 with out supporting evidence. And if the other orders …. that is very difficult. In addition there is no sufficient expect. Now here infectious diseases expert are not plenty enough to suggest this; there are no expert. So whenever there is no such things, may be in academic hospitals, may be an exception, because there are still discussion, there are people from every specialty, so there can be a discussion.

**Interviewer:** There will be a good opportunity.

**Respondent:** Private centers not necessarily. They could have. On the part time practice or private practice they could have an infectious diseases specialists. But other than that it is difficult to say that they are almost always available and order throughout the time. So it is concern all of us. Everyone should be concerned and worried about antibiotic resistance while working, otherwise it will be difficult.

**Interviewer:** So proceeding to the 2^nd^ question. Antibiotic resistance is an issue. How is the magnitude like from your daily practice? So do you think it is a problem for the hospital?

**Respondent:** Yes, from formerly I have described one of a study. And other than that there are no figures that I found, ceftriaxone resistant in ICU has been reported around 40%. Otherwise we see the other antibiotic in a case wise. For example while treating one of the case, let us say, the result of the culture sometimes reaches lately. So when we observe that culture result as compared to the previous 3-4 years we see many resistant. But in general I don’t have an exact figure.

**Interviewer:** From your experiences, which pathogens shows more resistance?

**Respondent:** (00:13:38). Since an issue of resistance raised there is a medication called methicillin resistance Staphylococcus aureus (MRSA). This days all the time in our antibiotics coverage, we are concerned about this area. For example let us say a patient with neutropenic fever, they can adequately be treated with cefepime. But in our practice, while working in our emergency practice, when a patient with neutropenic fever come we always discuss. We always argue with infectious diseases experts about this. When we see in our practice, all patients are here. All patient in a sense, there is a malignancy patient, the environment is so crowded. So it is difficult to say that the environment is so clean. Sometimes for this patients, a producer can be done, still being in this condition. So while the patient is in this condition, it is difficult to only say that we can cover with only cefepime. Because, methicillin resistance Staphylococcus aureus is becoming very common. For that reason vancomycin is added, and this is the first one. The other is E.coli by its strain. (00:14:31). we most often found E. coli to be very resistant. When we go to ICU practice klebsiella are more commonly found to be resistant bacteria. Like pseudomonas are the commonest pathogens found to be resistance.

**Interviewer:** How is the delay practice affecting? what are you doing to use vancomycin, the choice is limiting more of. Is treatment failure more common?

**Respondent:** Now here there is no exact figure. It needs study. It needs a research. Now of course because of being concerned for the patient, by compromising the rationality to some extent. We add antibiotic. Of course we see most of the patients improving. Sometimes if the patient is very critical, the infection becomes hospital acquired infection, the sepsis become very progressive. They develop septic shock. And then there is a higher mortality rate. And their main diseases, Let us say they have ALL, AML the main malignancy, and if the other is left untreated, this one also contributes, so the treatment fails. They may even end up in death. But when we see, most of them, there is an improvement. But the issue here is, we are compromising the rational use.

**Interviewer:** For resistance to increase one factor can be irrational drug use as you have told me, related to prescribing and dispensing. But do you think there are other contributing factors for resistance to increase.

**Respondent:** The other here that contributes is the strain of the pathogens. The pathogens with different mechanism, different strain, the possibility of resistance of the antibiotic, it is increasing, our environment.

**Interviewer:** infection prevention.

**Respondent:** Yes, those things, they contribute, in addition to this, we physicians, of course. I have said the rational use. In addition to that sometimes, our clinical decision may be evidence base. From the recent literatures, from the recent findings, and not going with evidence based practice. Those things are the contributors.

**Interviewer:** in terms of the private clinic basically at lower level, irrational antibiotic prescribing do you think there is knowledge gap.

**Respondent:** Yes, there is a gap. There in our health system is based on tire. The tire is not only to treat in terms of the population density, But also by experts too. So what it is, the more the level is lower, for example, experts in Primary Health Care Unit, and then referral hospital, and general hospital there is a difference in health professional experts. So based on that, there is a knowledge gap. So when we take an academic institution, to that extent there will be no knowledge problem. Because, there is a consultation, there is a discussion, Grand round, and journal clubs, there is an update. So in PHCU most often covered with GPs. so in that areas the recent, from the technology, from different aspect they could not find the recent evidence. So old practice still persists. Those gaps still are there. So I think it is one of the factor.

**Interviewer:** So as you have said here in academic institution working as a team is also one important thing. Lack of team work is one factor. You have said there is laboratory delay the culture and sensitivity test result. But do you think there are complains that are raised in laboratory raised from the physicians side. The first one is delay but other like in quality 00:19: 01

**Respondent:** Yes, there are, and one of them is, in some hospitals, in my internship practice that is almost 04 years back when we observe, in some hospital the lab technicians, they themselves come and we see how they collect culture. So this means we can obtain a skill while we are on work. However here the patients is given the request paper, he will handle the materials, wait handling the materials, then either the nurse, or interns or if it a producers physicians or other physicians will do the producers, and the patient will take the take. So it starts with the collection. So the 1^st^ thing in universal precaution is clean environment. And this things needs to maintain the sterility. So the contamination will start there. So starting from this and then after there is a delay in the laboratory. That delay because of there may be a lot of requests there or it could be from material, equipment or infrastructure. So this things start from sample collection. The problem is from sample collection. Delay may also be if, even after collection the attendants may delay it. Even they do not know how to handle it. So later the sample may be discarded. So starting from things like this and after it reaches to the laboratory till the result reaches it is also a big factors. But this days because of our research initiatives, relatively there are good things.

**Interviewer:** Most often it is used to stay longer day. Right.

**Respondent:** Yes, normally what is it, is that, blood culture preliminary reaches with 24-48 hours, the preliminary result. But if the preliminary result is negative still the patient wait for 10 days, for blood culture, for urine culture it reaches early, with in short period of time. But the good thing is, since the bottle for the culture of this things are found early, As much as possible before antibiotic is started the sample is collected early and sent. And later as the result reaches, it helps us to revise the antibiotic.

**Interviewer:** This is the current trend. But for example previously result of blood culture will not arrive within 24 hours. 21:56

**Respondent:** Yes, we also couldn’t find the preliminary also. That was why we continue empirically. That trend was what hurts now. Now even for this too, even if the bottles are available that trend hurts us. This trend still has an influence. Even if bottles are available, even if they are near the antibiotic will be started without using them.

**Interviewer:** But do the physicians send Culture? Is it a trend this time?

**Respondent:** Yes, this days different from the previous trend, because the bottle and material are available they will send. Sometimes there is an awareness problem. We are trying to address those things. But relatively from the previous one they are sending

**Interviewer:** we have said there is still delay, contamination. But, do the physicians trust the quality? Let you representing the others

**Respondent:** This days it has a good change / progress/. Previously culture of TASH was said to be negative always. Meaning there is no growth. So later on when it starts to have a growth, there as a doubt about this culture, which it used to have no growth, but this days it has a growth. So there were doubts like this, on the physician side. But now this days, from this clinician’s perspective, the case perspective, from the condition of the patient, the clinician decision itself if you treat and there is no respond and something that shows resistance and found to be sensitivity from the laboratory. So accepting the …. (00:22:32). of course if there is a contamination, the physician go and discuss and if the laboratory professional think it is contaminate, they will write contaminate. But always saying a contaminate report, it is difficult to accept that contaminate. For example in a sample taken from CNS and CSF it is very rare. For us if there is a significant growth, and if it is said it is contaminate, the person who did in the laboratory do not know the clinical condition. So here comes our decision, is important. So this days more to the discussion.

**Interviewer:** There is a discussion, it interests me very much, and some physician even go to the laboratory. I think it is a good thing.

**Respondent:** Yes, academic environment should be just like this, it is not like, the laboratory professional do the lab test there, then he gives you the result then you go and treat the patient. It shouldn’t be like that.

**Interviewer:** observing how they work.

**Respondent:** Exactly, we have to know, for the next time it has a significant input.

**Interviewer:** Final Question, for instance the use of antimicrobial resistance is like this and antimicrobial problem … (24:47). But currently antimicrobial containment strategy. If you think it out of the project. Do you think it is satisfactory of the things done at the hospital? What does it lack?

**Respondent:** Here, it concerns me a lot. I think it is difficult for me to think that there are enough things. So far the containment strategy, previously there was infection prevention, universal precaution trainings, posting in wards. But now this days when I observe I think there is no any. So here what I think is, for example while working here in emergency this result delay, as much as possible to minimize the time specifically, we are working for the antimicrobial containment strategy (00:25:53). So I think it is more of what we should do after forwards.

**Interviewer:** For the laboratory to have speed, quality related to contamination strategy, what others do you recommend to work on?

**Respondent:** The other thing what I think is that, here in emergency practice, taken as one quality improvement project, when septic patients or infectious patients that use antibiotics when they arrive, the use is rational and to let it be, from the contemporary … (00:26:30) based medicine plus to how much extent the septic work ups are working to the point. Before we go to antibiotic how much certain are we are and we are thinking of something like quality improvement project.

**Interviewer:** So in selected are quality improvement projects …..

**Respondent:** If septic patient arrives, is the workup for septic thing done? Is our antibiotics are used based on that or soon, so to differentiate all those things.

**Interviewer:** As you know them this Antimicrobials ……. program (00:27:13).you know there are 2 main things. So, the 1^st^ one is restriction based on the level of the physicians, for example when we take emergency, the consultant physicians or physicians at that level, when the intern prescribes selected medicines at high level it could be either by consent or argument. We call this restriction. And the 2^nd^ one, before explaining the 2^nd^ one, do you think this will have an acceptance or how do you see the impact for example in this hospital if it is implemented … (00:27:50) should ID be consulted.

**Respondent:** Of course I think it will help us. As far as the patient is critically septic and if he doesn’t miss the antibiotic in need; My fear is that, if he doesn’t miss that again for consultation if the patient still doesn’t miss it I think the restriction is helpful.

**Interviewer:** If a physician, if an intern or after he graduates does autonomy problem question him, like to let not all prescribes, actually the graduates it doesn’t include the graduates, but it is better if you tell me all in relation to all person.

**Respondent:** Autonomy, in my opinion it is not that much a problem. Because it is all about the rational use. To be rational, not to harm the patients and later not to lose the antibiotics, not to make the resistance serious. As far as the physicians become thoughtful and this antibiotic ought to be here, the issue or idea here, if he discuss with the consultant he will learn more about it but I don’t think this will restrict the autonomy. As an intern, for example they are just practicing under supervision, even the resident is doing practice meaning he is learning the practice learning the residency practice. So at the end of the day the decision is done with the consultation. So he will had a part in the learning. But no autonomy problem.

**Interviewer:** So to implement this, you think there will be no barriers.

**Respondent:** Yes.

**Interviewer:** Since there is an agreement and only what you picked like availability of the consultants. So as not to hurt the patients. So availability of consultants is very important. Because if they are not available, if you cannot prescribe If one consultant, if one resident, or intern. So as you have said it is a very big issue. So I take it as barrier. The other is Antimicrobial stewardship, prospective audit and feedback you will have physician, ID physician or consultant with team pharmacist, microbiologist probably what we taught was lab supported audit and feedback team is created. One physician, or it could be a resident or internist or other prescribed medicines or selected antibiotics or ward. I will hear from you which will be best / preferred. And those prescribed drugs the team will go on and as an option there in ward or in an office they will discuss as an option, “let this medicine be changed from IV to oral, from broad spectrum to narrow spectrum, dose should be decreased, duration should be decreased,” for one prescriber one physician is one of the main work in prospective and auditing and feedback. How is this? Do you think it is applicable in work, what about the feasibility?

**Respondent:** Yes, I think this is a quality improvement project. Because quality improvement project is to look at the end of the day and to correlate with the evidence based practice to improve what has been missed for the future. So this goes right to that and when the audit is done this days for one disease majority of the world are using what type of evidence practice. And what are we using so in the next step where should we go what did we miss by checking those things to have a continuous improvement. So auditing is very important. So in ICU practice there is usually an audit. The audit is not only for the antibiotic. But also over all for the patent care. At the end what will happen what we should continue for the next step. so relatively from the researches this immediately takes us to the rational use. Because researches may take time, research findings may take time. But auditing with I a day, they guide us to judgment early. So it let us to judge early. So they are important.

**Interviewer:** (32:33) It will have sustainability when you recommend it today, you are shaping it for tomorrow’s practice

**Respondent:** Yes, and also from others evidence, paper will be reviewed again, literatures are reviewed, discuss with the changed things, it is more of making the environment an academic environment.

**Interviewer:** But what kind of barriers are there to implement this. For example, implement as a pilot here in Ethiopia as a country what kind of barriers you think it will have.

**Respondent:** As BLH, I don’t think there will be difficulty, because more or less, the resources are available the professionals are available, the teams are available, so everything the academic environment is here. So implementing here as a pilot will be rather good. If we go to other places the problem may be experts. In that sense if a person gets educated when more specialists are trained that thing can be decreased / minimized so to start it, I think it will be very important.

**Interviewer:** Where as your opinion, where do you think it will be implemented? Like with which selected antibiotics in the ward or one ward specifically it could be like emergency, ICU, pediatric and soon what is your recommendation? Is it the ward or a specific antibiotics from the utilization of highly use?

**Respondent:** Of course, it will be good if it is from the sights, it should be from emergency. Because all diseases burden patient come, emergency in a sense both adult and pediatric. And also ICU very critically ill patients most often be admitted from emergency. So ICU is also an important area. The other is ward it could not be all wards, one from surgical ward, one from medical ward, one from pediatric ward, and the other labor ward, from gyn. And I think including this.

**Interviewer:** which one is better; one at a time or in a step wise manner.

**Respondent:** Probably patients transferred from emergency to ICU so, following that patient more of helps to let us work on a continuous work.

**Interviewer:** Follow up

**Respondent:** At the same time different area, it will help us to go through both ways, and in a time wise.

**Interviewer:** If we combine both for example restriction and prospective and feedback what is your opinion. For example if we already have a 12 month antibiotic utilization. Actually we do have so if we separate the highly consumed antibiotic and very broad antibiotic at high level and restrict it somehow if combine perspective auditing and feedback, how do you feel.

**Respondent:** Still the combination will work very much. I think it is a very best thing to work combined.

=================================================================================

## Physician KI#09

Age: 27 years

Sex: Male

Staff Position: Resident (R3-Chief resident)

Department: Emergency Medicine

Work experience: 4 years

Number of patients treated per day: 50 patients

Number of patients with ABX prescription per day: 25 patients

Duration of interview (minutes): 36:10 Minutes

**Interviewer:** I have 3 major question. The first one is related to antibiotic use, the second one is related to antimicrobial resistance and the third one is continence strategy particularly on the antimicrobial stewardship program. So to start with. How do you describe the current use of antibiotics in Ethiopia and particularly in this institution?

**Respondent:** Our antibiotic use, more of this days, from what we are observing, it is difficult to say it is rational. It is difficult to say we are using it properly. Even when we see in emergency experience, when the patients arrive here they may come here after they start any/every antibiotic. That means, an antibiotic which is a very broad antibiotic or from the list, they may come here after they started a medicine among the top on the list. The problem is that, not only those patients started those medication but also, there might not be any test done. Those patients do not have tests done like sensitivity, blood culture and urine culture. Already the treatment has been started, so after this, if we do culture it will be much distorted. We could not find the correct thing. And the patients do not only come from home, and when they come here after starting the top level antibiotic, they come here after being admitted in another hospital. ***So the infection is not only acquired from the community, it is also acquired from the hospital.*** So sometimes the treatment started is for the hospital acquired or for the community infection. Or has it been shifted because the community acquired has not been resolved. We cannot differentiate all those. ***So I say the rational use is right antibiotic, right time, right dose, and right selection. So it is very difficult for me to say it is rational.*** Even not only for those who come, but also when we use or when we prescribe the antibiotic for them, sometimes it could be due to material shortage, it could be due to that. This days there are equipment’s for culture. But otherwise if those materials are not available, treatment will be started empirically, even a very top antibiotics. The patients are really sick, they are very critical. Or they could have malignancy. So in order to cover that, they will be started on a very wide spectrum. ***The culture will either be sent lately, or might not be sent at all. So the result will not arrive early. So before the result reaches, if the patient doesn’t respond the antibiotic will be changed.*** So when we see this, it’s not only for the diseases at that time on arrival but subsequently when this patients are admitted it will be difficult for selection. So I think it will be difficult.

**Interviewer:** So through time, as of your four years’ experience, how do you see the use of antibiotics over time? Is the problem of irrational use increasing, constant or decreasing as time to time?

**Respondent:** This days considering the burden of diseases, and from those patients admit how many of them are critical how many of them have a difficult diseases meaning since it is critical, it will be difficult. It is difficult to blame that the antibiotic use is irrational. It is also difficult to accept that it is also rational. So it is more of some kind of mix. So not to say it is rational, let us say as of my four years’ experience, when I see in my practice, in my first year experience ***ceftriaxone was the antibiotic that has been used right and left. So it is a broad spectrum, it covers and so on. But later on with in that year what has happened every one prescribes ceftriaxone. That means, private sectors even at a clinic level, started to prescribe. Later it happened to be, I think in a study in Black Lion a study involving an ICU patients, if I am not mistaken about figure the resistance reaches to 40 %.*** So while ceftriaxone as it is, has been used right and left, right and left, in a manner that is not rational, ***another antibiotics came. That covers a lot, like ceftazidime, cefepime, vancomycin. All of a sudden while they were available, currently this days commonly we used ones potent antibiotics like Peptazo and meropenem***; we reached there. so what it is, we do not have study which antibiotic have how much, for example I casually told you about ceftriaxone, but after that there was no a wide or a hospital wide study or any other study that shows the sensitivity. So without being based on that we moved to the other broad spectrum antibiotic. Without looking at the other studies. To that extent the diseases thing is also increasing. The patients, not only come here with an acquired infection, but they also have comorbidities and other illness, that means for example when they are neutropenic and will come with neutropenic fever. Or they could have malignancy and their immunity will be low. ***So we tend to make the antibiotic very wide to cover it. And sometimes it’s because of being concerned for the patient. Even if, we are concerned for the patient, due to shortage of material; and because of the time, since we are using it without culture or without any support,*** with in these four years, it is difficult for me to say that the rational has been increasing.

**Interviewer:** You have told me in relation with infrastructure, laboratory, but what about the influence related to medicine shortage so is there an influence. The over use or broad spectrum antibiotic. So does the availability of medicines has an influence or not. (00:06:24).

**Respondent:** Yes, this is really a great question. Because commonly antibiotics used after ceftriaxone are ceftazidime with vanco. For example if a patient develops hospital acquired infection we go to this antibiotics. Now when we use this antibiotic at that time, they are provided by the hospital and have a relatively cheaper prices. Later on when this antibiotics are not available at the hospital, the choice of the patent is to buy from a pharmacy outside the hospital. And buying from outside is very costly. They will differ in brand, the patients come from country side. They will sell everything they have. And at the end they will be very critical. So when we need antibiotic for this patients the patients may not find them. Because of the cost. And above the cost the availability is on and off in the country. So let us say a patent who has aspiration pneumonia who is taking ceftriaxone and metronidazole, if he doesn’t respond with that and if there is a new fever shoot and we still says its hospital acquired so our option to treat as a hospital acquired is by using ceftazadin and vanco. So while working, this really happen at this hospital, and at some point this antibiotic are not available. So when this antibiotic are not available, our option is to continue the antibiotic. And when we continue with this antibiotic, of course this patients is having a new fever shoot, we are not doing the culture and sensitivity, and the patients cannot afford to work up the laboratory investigation outside the hospital. So because of that, due to the availability of the medication in terms of the cost, in terms of the infrastructure, we will continue the one which is already available or with the one which is relatively good, if it is available, we will move to that one. As to my opinion the first reason that contributes for irrational use is the shortage of resource and the medicines, which is also one of the factors. Because the mediation that we think is suitable for the patient, if it is available at the hospital, and if the patient finds it with a reasonable cost, of course we will treat him with that. If it is not available in the hospital and if the patient cannot afford it our option is, we will use the medication that is available at the hospital with a cheaper cost. But, has a little help for the patient. So availability of the medication plus even if the medication is available our infrastructure also matters.

**Interviewer:** How do you see the patient influences? Like there is something seen in the private. But here in this hospital basically in government hospitals, we see in 2 sides. The 1^st^ one is the need of the patient to prescribe antibiotic or in the opposite side the fear of the patient’s when antibiotic is prescribed for them or for their child. So does this has an influence? (00:08:59)

**Respondent:** Of course in antibiotic it has an impact. Patient more often go to a nearby health facility or may prefer to go to private and soon. Here we cannot advocate any different thing that is; one is better than the other one. So as an emergency health care professional, if an emergency condition happens they may go to a nearby clinic; or initially go to health center then go to another hospital. But what we observe here is, in all the places they go the trend is, the sector that treats them at that time gives a treatment from his side instead of considering another thing. We are not observing those things. If it would have been that because this days the issue of antibiotic, we all should be concerned of. It is one of the concern of WHO. Antibiotic resistance is one of the big issue this days. So if one center prescribes an antibiotic, he should be reasonable. For example when we say reasonable, when he order a very wide antibiotic, there should be a reason. And if possible if he refers a case for example like here in BLH, if he keeps the reason; write the result of the test done; so the communication will be based on that. Otherwise, if one … 00:10:39 with out supporting evidence. And if the other orders …. that is very difficult. In addition there is no sufficient expect. Now here infectious diseases expert are not plenty enough to suggest this; there are no expert. So whenever there is no such things, may be in academic hospitals, may be an exception, because there are still discussion, there are people from every specialty, so there can be a discussion.

**Interviewer:** There will be a good opportunity.

**Respondent:** Private centers not necessarily. They could have. On the part time practice or private practice they could have an infectious diseases specialists. But other than that it is difficult to say that they are almost always available and order throughout the time. So it is concern all of us. Everyone should be concerned and worried about antibiotic resistance while working, otherwise it will be difficult.

**Interviewer:** So proceeding to the 2^nd^ question. Antibiotic resistance is an issue. How is the magnitude like from your daily practice? So do you think it is a problem for the hospital?

**Respondent:** Yes, from formerly I have described one of a study. And other than that there are no figures that I found, ceftriaxone resistant in ICU has been reported around 40%. Otherwise we see the other antibiotic in a case wise. For example while treating one of the case, let us say, the result of the culture sometimes reaches lately. So when we observe that culture result as compared to the previous 3-4 years we see many resistant. But in general I don’t have an exact figure.

**Interviewer:** From your experiences, which pathogens shows more resistance?

**Respondent:** (00:13:38). Since an issue of resistance raised there is a medication called methicillin resistance Staphylococcus aureus (MRSA). This days all the time in our antibiotics coverage, we are concerned about this area. For example let us say a patient with neutropenic fever, they can adequately be treated with cefepime. But in our practice, while working in our emergency practice, when a patient with neutropenic fever come we always discuss. We always argue with infectious diseases experts about this. When we see in our practice, all patients are here. All patient in a sense, there is a malignancy patient, the environment is so crowded. So it is difficult to say that the environment is so clean. Sometimes for this patients, a producer can be done, still being in this condition. So while the patient is in this condition, it is difficult to only say that we can cover with only cefepime. Because, methicillin resistance Staphylococcus aureus is becoming very common. For that reason vancomycin is added, and this is the first one. The other is E.coli by its strain. (00:14:31). we most often found E. coli to be very resistant. When we go to ICU practice klebsiella are more commonly found to be resistant bacteria. Like pseudomonas are the commonest pathogens found to be resistance.

**Interviewer:** How is the delay practice affecting? what are you doing to use vancomycin, the choice is limiting more of. Is treatment failure more common?

**Respondent:** Now here there is no exact figure. It needs study. It needs a research. Now of course because of being concerned for the patient, by compromising the rationality to some extent. We add antibiotic. Of course we see most of the patients improving. Sometimes if the patient is very critical, the infection becomes hospital acquired infection, the sepsis become very progressive. They develop septic shock. And then there is a higher mortality rate. And their main diseases, Let us say they have ALL, AML the main malignancy, and if the other is left untreated, this one also contributes, so the treatment fails. They may even end up in death. But when we see, most of them, there is an improvement. But the issue here is, we are compromising the rational use.

**Interviewer:** For resistance to increase one factor can be irrational drug use as you have told me, related to prescribing and dispensing. But do you think there are other contributing factors for resistance to increase.

**Respondent:** The other here that contributes is the strain of the pathogens. The pathogens with different mechanism, different strain, the possibility of resistance of the antibiotic, it is increasing, our environment.

**Interviewer:** infection prevention.

**Respondent:** Yes, those things, they contribute, in addition to this, we physicians, of course. I have said the rational use. In addition to that sometimes, our clinical decision may be evidence base. From the recent literatures, from the recent findings, and not going with evidence based practice. Those things are the contributors.

**Interviewer:** in terms of the private clinic basically at lower level, irrational antibiotic prescribing do you think there is knowledge gap.

**Respondent:** Yes, there is a gap. There in our health system is based on tire. The tire is not only to treat in terms of the population density, But also by experts too. So what it is, the more the level is lower, for example, experts in Primary Health Care Unit, and then referral hospital, and general hospital there is a difference in health professional experts. So based on that, there is a knowledge gap. So when we take an academic institution, to that extent there will be no knowledge problem. Because, there is a consultation, there is a discussion, Grand round, and journal clubs, there is an update. So in PHCU most often covered with GPs. so in that areas the recent, from the technology, from different aspect they could not find the recent evidence. So old practice still persists. Those gaps still are there. So I think it is one of the factor.

**Interviewer:** So as you have said here in academic institution working as a team is also one important thing. Lack of team work is one factor. You have said there is laboratory delay the culture and sensitivity test result. But do you think there are complains that are raised in laboratory raised from the physicians side. The first one is delay but other like in quality 00:19: 01

**Respondent:** Yes, there are, and one of them is, in some hospitals, in my internship practice that is almost 04 years back when we observe, in some hospital the lab technicians, they themselves come and we see how they collect culture. So this means we can obtain a skill while we are on work. However here the patients is given the request paper, he will handle the materials, wait handling the materials, then either the nurse, or interns or if it a producers physicians or other physicians will do the producers, and the patient will take the take. So it starts with the collection. So the 1^st^ thing in universal precaution is clean environment. And this things needs to maintain the sterility. So the contamination will start there. So starting from this and then after there is a delay in the laboratory. That delay because of there may be a lot of requests there or it could be from material, equipment or infrastructure. So this things start from sample collection. The problem is from sample collection. Delay may also be if, even after collection the attendants may delay it. Even they do not know how to handle it. So later the sample may be discarded. So starting from things like this and after it reaches to the laboratory till the result reaches it is also a big factors. But this days because of our research initiatives, relatively there are good things.

**Interviewer:** Most often it is used to stay longer day. Right.

**Respondent:** Yes, normally what is it, is that, blood culture preliminary reaches with 24-48 hours, the preliminary result. But if the preliminary result is negative still the patient wait for 10 days, for blood culture, for urine culture it reaches early, with in short period of time. But the good thing is, since the bottle for the culture of this things are found early, As much as possible before antibiotic is started the sample is collected early and sent. And later as the result reaches, it helps us to revise the antibiotic.

**Interviewer:** This is the current trend. But for example previously result of blood culture will not arrive within 24 hours. 21:56

**Respondent:** Yes, we also couldn’t find the preliminary also. That was why we continue empirically. That trend was what hurts now. Now even for this too, even if the bottles are available that trend hurts us. This trend still has an influence. Even if bottles are available, even if they are near the antibiotic will be started without using them.

**Interviewer:** But do the physicians send Culture? Is it a trend this time?

**Respondent:** Yes, this days different from the previous trend, because the bottle and material are available they will send. Sometimes there is an awareness problem. We are trying to address those things. But relatively from the previous one they are sending

**Interviewer:** we have said there is still delay, contamination. But, do the physicians trust the quality? Let you representing the others

**Respondent:** This days it has a good change / progress/. Previously culture of TASH was said to be negative always. Meaning there is no growth. So later on when it starts to have a growth, there as a doubt about this culture, which it used to have no growth, but this days it has a growth. So there were doubts like this, on the physician side. But now this days, from this clinician’s perspective, the case perspective, from the condition of the patient, the clinician decision itself if you treat and there is no respond and something that shows resistance and found to be sensitivity from the laboratory. So accepting the …. (00:22:32). of course if there is a contamination, the physician go and discuss and if the laboratory professional think it is contaminate, they will write contaminate. But always saying a contaminate report, it is difficult to accept that contaminate. For example in a sample taken from CNS and CSF it is very rare. For us if there is a significant growth, and if it is said it is contaminate, the person who did in the laboratory do not know the clinical condition. So here comes our decision, is important. So this days more to the discussion.

**Interviewer:** There is a discussion, it interests me very much, and some physician even go to the laboratory. I think it is a good thing.

**Respondent:** Yes, academic environment should be just like this, it is not like, the laboratory professional do the lab test there, then he gives you the result then you go and treat the patient. It shouldn’t be like that.

**Interviewer:** observing how they work.

**Respondent:** Exactly, we have to know, for the next time it has a significant input.

**Interviewer:** Final Question, for instance the use of antimicrobial resistance is like this and antimicrobial problem … (24:47). But currently antimicrobial containment strategy. If you think it out of the project. Do you think it is satisfactory of the things done at the hospital? What does it lack?

**Respondent:** Here, it concerns me a lot. I think it is difficult for me to think that there are enough things. So far the containment strategy, previously there was infection prevention, universal precaution trainings, posting in wards. But now this days when I observe I think there is no any. So here what I think is, for example while working here in emergency this result delay, as much as possible to minimize the time specifically, we are working for the antimicrobial containment strategy (00:25:53). So I think it is more of what we should do after forwards.

**Interviewer:** For the laboratory to have speed, quality related to contamination strategy, what others do you recommend to work on?

**Respondent:** The other thing what I think is that, here in emergency practice, taken as one quality improvement project, when septic patients or infectious patients that use antibiotics when they arrive, the use is rational and to let it be, from the contemporary … (00:26:30) based medicine plus to how much extent the septic work ups are working to the point. Before we go to antibiotic how much certain are we are and we are thinking of something like quality improvement project.

**Interviewer:** So in selected are quality improvement projects …..

**Respondent:** If septic patient arrives, is the workup for septic thing done? Is our antibiotics are used based on that or soon, so to differentiate all those things.

**Interviewer:** As you know them this Antimicrobials ……. program (00:27:13).you know there are 2 main things. So, the 1^st^ one is restriction based on the level of the physicians, for example when we take emergency, the consultant physicians or physicians at that level, when the intern prescribes selected medicines at high level it could be either by consent or argument. We call this restriction. And the 2^nd^ one, before explaining the 2^nd^ one, do you think this will have an acceptance or how do you see the impact for example in this hospital if it is implemented … (00:27:50) should ID be consulted.

**Respondent:** Of course I think it will help us. As far as the patient is critically septic and if he doesn’t miss the antibiotic in need; My fear is that, if he doesn’t miss that again for consultation if the patient still doesn’t miss it I think the restriction is helpful.

**Interviewer:** If a physician, if an intern or after he graduates does autonomy problem question him, like to let not all prescribes, actually the graduates it doesn’t include the graduates, but it is better if you tell me all in relation to all person.

**Respondent:** Autonomy, in my opinion it is not that much a problem. Because it is all about the rational use. To be rational, not to harm the patients and later not to lose the antibiotics, not to make the resistance serious. As far as the physicians become thoughtful and this antibiotic ought to be here, the issue or idea here, if he discuss with the consultant he will learn more about it but I don’t think this will restrict the autonomy. As an intern, for example they are just practicing under supervision, even the resident is doing practice meaning he is learning the practice learning the residency practice. So at the end of the day the decision is done with the consultation. So he will had a part in the learning. But no autonomy problem.

**Interviewer:** So to implement this, you think there will be no barriers.

**Respondent:** Yes.

**Interviewer:** Since there is an agreement and only what you picked like availability of the consultants. So as not to hurt the patients. So availability of consultants is very important. Because if they are not available, if you cannot prescribe If one consultant, if one resident, or intern. So as you have said it is a very big issue. So I take it as barrier. The other is Antimicrobial stewardship, prospective audit and feedback you will have physician, ID physician or consultant with team pharmacist, microbiologist probably what we taught was lab supported audit and feedback team is created. One physician, or it could be a resident or internist or other prescribed medicines or selected antibiotics or ward. I will hear from you which will be best / preferred. And those prescribed drugs the team will go on and as an option there in ward or in an office they will discuss as an option, “let this medicine be changed from IV to oral, from broad spectrum to narrow spectrum, dose should be decreased, duration should be decreased,” for one prescriber one physician is one of the main work in prospective and auditing and feedback. How is this? Do you think it is applicable in work, what about the feasibility?

**Respondent:** Yes, I think this is a quality improvement project. Because quality improvement project is to look at the end of the day and to correlate with the evidence based practice to improve what has been missed for the future. So this goes right to that and when the audit is done this days for one disease majority of the world are using what type of evidence practice. And what are we using so in the next step where should we go what did we miss by checking those things to have a continuous improvement. So auditing is very important. So in ICU practice there is usually an audit. The audit is not only for the antibiotic. But also over all for the patent care. At the end what will happen what we should continue for the next step. so relatively from the researches this immediately takes us to the rational use. Because researches may take time, research findings may take time. But auditing with I a day, they guide us to judgment early. So it let us to judge early. So they are important.

**Interviewer:** (32:33) It will have sustainability when you recommend it today, you are shaping it for tomorrow’s practice

**Respondent:** Yes, and also from others evidence, paper will be reviewed again, literatures are reviewed, discuss with the changed things, it is more of making the environment an academic environment.

**Interviewer:** But what kind of barriers are there to implement this. For example, implement as a pilot here in Ethiopia as a country what kind of barriers you think it will have.

**Respondent:** As BLH, I don’t think there will be difficulty, because more or less, the resources are available the professionals are available, the teams are available, so everything the academic environment is here. So implementing here as a pilot will be rather good. If we go to other places the problem may be experts. In that sense if a person gets educated when more specialists are trained that thing can be decreased / minimized so to start it, I think it will be very important.

**Interviewer:** Where as your opinion, where do you think it will be implemented? Like with which selected antibiotics in the ward or one ward specifically it could be like emergency, ICU, pediatric and soon what is your recommendation? Is it the ward or a specific antibiotics from the utilization of highly use?

**Respondent:** Of course, it will be good if it is from the sights, it should be from emergency. Because all diseases burden patient come, emergency in a sense both adult and pediatric. And also ICU very critically ill patients most often be admitted from emergency. So ICU is also an important area. The other is ward it could not be all wards, one from surgical ward, one from medical ward, one from pediatric ward, and the other labor ward, from gyn. And I think including this.

**Interviewer:** which one is better; one at a time or in a step wise manner.

**Respondent:** Probably patients transferred from emergency to ICU so, following that patient more of helps to let us work on a continuous work.

**Interviewer:** Follow up

**Respondent:** At the same time different area, it will help us to go through both ways, and in a time wise.

**Interviewer:** If we combine both for example restriction and prospective and feedback what is your opinion. For example if we already have a 12 month antibiotic utilization. Actually we do have so if we separate the highly consumed antibiotic and very broad antibiotic at high level and restrict it somehow if combine perspective auditing and feedback, how do you feel.

**Respondent:** Still the combination will work very much. I think it is a very best thing to work combined.

=================================================================================

## Physician KI#10

Age: 25 years

Sex: Male

Department: Emergency Department [R1]

Staff Position: Resident [R1]

Total work experience: 2 year

Number of patients treated per day: 25 patients

Number of patients with ABX prescription per day: 12 patients

Total Interview time (duration): 27:53 Minutes

**Interviewer:** Thanks. So as a starting point, in general from what you observe in our hospital or country or nationwide, also from your private experience, How do you describe the use of antibiotics?

**Respondent:** When you observe antibiotic use, especially on private health facilities in our country, ***antibiotic use is not appropriate.***  We are on a situation of antibiotic resistance which is higher. Because in private clinic the ***urge is to treat patient early and get immediate effect***. So the situation there is not by starting from the lower and increase to the higher but immediately it is started with the more effective broader spectrum antibiotics. ***So, to get immediate benefits, we use broad spectrum antibiotic more frequently, on private setup.*** But when we come in TASH, it is also a teaching hospital many consultants are involved, somehow it is lower than that. But however even if it is not like that, TASH or any other, antibiotic resistance is there and globally it is the 21^st^ century threat.

**Interviewer:** So which types of inappropriate use of antibiotics is common? How do you see the level of overuse, underuse or others?

**Respondent: *Under use is very few***, if it is available sometimes it is very few who says, let’s try it and this is usually due to affordability issues. ***Majority of what is found is over use either as a broad spectrum, when they can start with other antibiotic.*** Or you will start with broad spectrum. Or you will escalate the does. Or after investigation but before all results are seen antibiotic will be started. So when you observe this, as a country, the use is almost similarly like this.

**Interviewer:** So reasons for what you told me like the first one is to cure early.

**Respondent:** Especially in private setup. Because normally the hospital in ***private clinic normally they need the patient to come frequently, they will see the effect of today rather than anticipating the effect of tomorrow. So they simply give more potent antibiotic. .***

**Interviewer:** What other factors do you think are there? Other reasons for use of the empirical treatment?.

**Respondent:** Mostly, you start with empiric treatment. Even if the situation lets you to start empiric treatment. However in empiric treatment, you will ***treat without knowing the specific organisms b***ut we have enough investment to identify if there is an infection or not. For example, the 1^st^ reason that we do not give specific antibiotic for a specific organism/ bacteria is that we ***don’t have strong microbiology.*** It is like laboratory or all professional could have work together and bring a changed it but at the physician level yes there is a knowledge at all level, there are few persons who do not know what should be prescribed for whom. ***But everyone make it a trend and they are doing hit hard, hit hard, I think this is negligence.***

**Interviewer**: But what could be the reason. One reason could be what you already told me like, ***broad is given in private setup because they want to cure the patient***, but what major reason to use broad spectrum.

**Respondent:** I think you know if you are asked about antibiotic resistance, there is something you know, like it is something in our subconscious, a knowledge from using it. Consciously you are not using that. If someone asks you about something that you already read I, you will discuss freely. But practically peoples don’t practice it. Like on antibiotic choice ***didn’t use the guideline***. And the other thing is ***hospitals do not have their own guideline.*** For example nationally, if there is a national guideline, and in each hospital if there is guideline that says for this disease this antibiotic. This will solve it. ***Now our decision is personal.*** ***There is nothing done in our country to say that, you are right, you are wrong rather than reading books. So peoples may order you, and say in my experience it is like this. So this needs a guidelines based on each diseases.*** And also showing persons the cause of real of antibiotic resistance treats [***lack of awareness on the consequences]***. We know it but it is subconscious. As I have told you it is a knowledge that we don’t use it, something far from us. It is a knowledge that we don’t use it daily. If it is done this way. And I think this are the things I think.

**Interviewer:** As you have said; it may be important to show the impact or the burden for all the professional. But does availability of medicines affect you? You might need a lower antibiotic.

**Respondent:** Most often medicines at higher level will not be available most often. But recently I am not certain about it. Medicines found at lower level are accessible. ***So when you are looking for the higher level medications, hit hard, you won’t find those medicines.*** Even if it is like that, we prescribe those medicines, the price is known. So here the base is like, to teach the physician to come in to practice, and guideline. Guideline solve this. If there is a guideline for every disease in each hospital, so in this hospital, the manager can say; why did you treat this with this. ***But, at this time you cannot say why you gave this. But in the country, there is no guideline in the hospital that says treat this with this.***

**Interviewer:** If there is no evidence, it will be difficult. Is there a patient influence in prescription to prescribe antibiotic or not to prescribe?

**Respondent:** In private, definitely**. *In private some who came from rich family, or someone who saw on an internet, he need to be cured early, he won’t hear you if you tell him about antibiotic resistance***. So you tell him try with thus, with the lowest cost, if you said to him try with this and I will change it to another, they refuse to do so. Not most people, but there are basically here in Addis Ababa, private, population like this are coming. Let this be, I want this. They want to be on broad. At that moment, it might not be a serious thing. ***But now this trend is coming and increasing***.

**Interviewer:** You think it has an influence in prescription, probably in private.

**Respondent: *Yes, it has an influence in private. But the number is not that much significant/ major, but currently it has its own influence, and in the future there will be a lot.***

**Interviewer:** You think it is growing over time?

**Respondent:** Yes, it is a growing.

**Interviewer:** To the opposite, due you encounter patients who don’t want to prescribe antibiotics to themselves or their family member?

**Respondent:** Rarely, there is. And sometimes, rarely patients are saying that “..no, no let’s see it first, then you will write it to me later. But this is rare. ***Even if these two are rare, but the influence of demanding more potent antibiotics is increasing more.*** Let this be prescribed. The other is the research is poor. For example when you say antibiotic resistance is this much, you will tell the figure other than our country. ***We don’t have a correct figure***, like if there is an evidence, a study done on antibiotic resistances, each hospital has a strong microbiologic units and if the trend of the hospital is develop, this is how we you can change the peoples. So like research, ***guidelines are scare***, they have an effect, have a prominent effect. ***The other is a trend. Now physicians are not ordering antibiotic from current evidences but from what they have seen from their mentor or seniors, even it is a previous thing or not up to date, you follow what he has done. You will proceed by following what he is doing.*** Antibiotic resistance is a very changing topic, antibiotics choice should be updated daily. It is changing frequently, ***we are poor in update,*** ***we follow the trend, and you could also order a medicine which has already developed a resistance***, for example if you have an update of an antibiotic resistance which is uplifting, you could be cautious but instead of doing that, we are following the trend. This also has its own effect.

**Interviewer:** How about the preference of IV Vs PO?

**Respondent:** In BLH there are a lot of person and those who come here are already stiffed and he is already accept what you say. So, this problem is not common, it is rare, this is not raised as a problem, but ***in private there will be, may be not as a major problem, currently it is raising problem in private hospitals.***

**Interviewer:** The second question is related to resistance, do you think resistance of bacteria is a problem?

**Respondent:** [laughed] Every time! Like overtime there are global threats that we faced and currently the major global threat is antibiotic resistance. ***It is equivalent to disaster of climate change and terrorism.*** Normally my work with other companies, is maybe, because it is related to this. ***I think everybody knows that antibiotic resistance is a major threat,*** it is known. ***So this is a significant public health problem.***

**Interviewer:** Why is it increasing through time? What do you think, could be the reason from your experience?

**Respondent:** The is different from country to country or from place to place. But the reason can be classified it in 3. The first problem is ***over the counter prescription***, in our country in rural areas, other than Addis Ababa, Amoxicillin, you can buy it ***so when people feel something even viral infection or any other thing, they take amoxicillin***. Food poisoning, if it don’t have ciprofloxacin will be taken, so this over the counter prescription has a major impact. The second one is from the prescriber side, the first one is ***prescribers has less knowledge***, when I say prescriber, from the one who writes the prescription to dispenser, ***lack of knowledge***. The second one is this ***persons do not know the update of antimicrobial resistance***, and as I have told you earlier there are ***no guidelines***, there are no major guidelines. Now at hospital level, you can solve antibiotics problem by guideline. By saying work with this guideline. Since this guidelines are not available, they have their own impact. The other is social, social means the community’s perception. As what you have said earlier, let it be higher, let it be higher. So the perception of the public related to antibiotic and antibiotic resistance matters. Now if the public understands about antibiotic resistance as a threat, health professionals know about it, but works by denying it. ***But the public doesn’t know that antibiotic resistance a major public health problem***. Why do I concern if it cures me, for the public there is nothing done that it has a problem later on. ***Like if you tell to the public, when you order him tomorrow, like you can make him to ask you that, can’t this work with something lower than this?*** The 3^rd^ component is the major component is the public, the one who takes the prescription, so in here you have to work. You have to work with media, educating patients, health education, and a lot. Like ***making microbiological facility strong***, like when you send a blood culture, now one of the problem that the physicians really facing is, when he can treat with a specific blood culture result, when a sepsis patient comes initially you ***start him with broad spectrum,*** later with blood culture you come to a specific organism. ***But here blood culture is negative***, so you will not stop that broad spectrum antibiotic, so you will continue to use that broad spectrum antibiotic, because you do not know where it act on. So here major lab, microbiologic facility should be majorly improved.

**Interviewer**: So you mean you mostly receive negative results?

**Respondent:** So what you do is, even if you know there is an infection, when I say you negative, you will not change from broad spectrum to narrow therapeutic range. Those that work narrowly. Meaning if it is told that this will work for this, if you don’t know the organisms, you will continue with the broad spectrum. You have to make the lab system strong, you have to teach the physicians, you have to teach pharmacist’s, you have to develop guideline, you have to educate the public through medias, health education for example, it has previously been stated when peoples sat own, about TB, malaria, about antibiotics, like this be a major issue, this should be an issue that needs an attention. Also ***team work should be strengthened.***

**Interviewer:** How do you see the status of infection prevention in this hospital?

**Respondent:** Normally you do not separate antibiotic resistance and infection prevention. As you prevent infection, you will decrease the antibiotics that you will order. So you will also decrease the resistance too. There is a change in infection prevention from the previous one. It will be discussed, in everywhere, every time. ***But infection prevention, it is a work done during an assessment time, when there is something said, infection prevention, education, then after.*** This is because, public health issues the problem is not only at the initiation, there is a problem in continuously sustaining, so in here, there are some periods where infection preventions, or where assessment is done, there is some periods. ***But this is a major issue where infection prevention should be used to assess hospital performance.*** But later it will not be done, it will benefit us if it is sustainable. You cannot prevent antibiotic resistance, by doing infection prevention for one week. But this hospitals protocol, what can you do. For example in the abroad, it is one of the way for assessing the functionality of the hospital performance. For example infection prevention has its own indicators abroad their own, they have an indicators of infection prevention that shows where they go. So with that indictors, you will assess the persons. But in here we have to assess that the indicators work or indicate correctly. When we want to do an assessment, and if the indicator is that it will be low. So a day to day report, on infection prevention, antibiotic resistance. So a system should be developed. A strong system, a system that has a control and monitoring system. You can do a lot. Starting from, teaching, infection prevention, from laboratory, and a system that is integrated from all, which can be sustained and maintained, and develop a system and follow continuously. In every step you should have an indicator. It should not be in a sense of, I have done. But there should be an indicator where we can see ourselves from the indicator, there should be an indicator for every person.

**Interviewer:** As you have said, most often the laboratory results are negative. Do you think physicians order many lab requests, and do the physicians have trust on the laboratory results.

**Respondent: *To give an honest reply, it is difficult to say we have a trust in laboratory findings.*** You will send it as a formality, if fortunately it is positive you follow their recommendations but if it negative it is very difficult to trusting the laboratory result and thus to change your treatment. We don’t stop treatment if they say blood culture is negative. So until the lab gets strong and trust is build, you can’t not blame the physicians, I mean when you know that there is an infection, but you are told it is negative, you should not stop, you have to work on laboratory,

**Interviewer:** From your experience which pathogens are found to be most resistant pathogens, it will also help us on interventions,

**Respondent:** From the pathogens that are resistant for lot of things are, ***S. aureus,*** when you found S. aureus most often resistance for many medicines, basically patients that are discharges from hospitals, S. aureus are resistant. ***E.coli*** previously responds for ciprofloxacin, and ceftriaxone, but now you see resistance on it, you will find E.coli on UTI. But majorly, Staphylococcus specious

**Interviewer:** There is a problem of antibiotic use, there is also antimicrobial resistance, and do you think the work done do decrease resistance is satisfactory?

**Respondent:** This become an issue here recently, when you think something, you should not make the previous thing zero, this is a good start, and to discuss an issue about an antibiotic resistance, and starting some kind of things like this is a good start, even if the assessment of infection prevention program is not sustainable, its availability is also a is a good start. Now the major thing is, it’s should not be only the starting but also, to develop a sustainable system, we are immature in it. But this is something that you can build on it, starting from here you can do a lot of things, we cannot say that, the things done right now are zero, but it is not enough, there are a lot of things left undone. From the things that are already done, the thing that we will do is much bigger. So it is a good start.

**Interviewer:** What do you think if an antimicrobial stewardship program [the interviewer briefed how prospective audit and feedback works] is applied. So, How would you feel if prospective audit and feedback is applied in this institution? What are the barriers and enablers for this intervention?

**Respondent:** If it’s done properly it has a ***positive impact***, but to work this, at the very beginning, for the physicians to work with the lab, t***he lab should be a trusted, a laboratory that should be strong and should be strengthened.*** Then commitment of peoples, which follow this, working on this things, a ***commitment of the consultants***, and the other is, a system that is developed from minister of health, a strategic plan a system, and to execute this persons, as we have discussed earlier, by using this as one of the assessment way, in each hospital, you can execute it and make it part of the system in each health facilities. If it done it is a good thing, but the ***challenge*s,** it is ***difficult to trust the laboratory, it needs a strong commitment from the people***. And the other is, ***first line prescribers, they will say what is my work, if I am doing, what I am told,*** it depends, but it could also makes people to feel like that, if it is done it is good for public health.

**Interviewer**: So autonomy, do you think there will be problem of autonomy?

**Respondent: *In teaching institution it won’t be. Because the consultant is your teacher, there won’t be a resistance,*** although we do not know whether they are doing by force or willingly. But you will follow what they will do so in teaching hospital there will be no problem. ***But we will not be positive when we are told to do so, for our mistakes.* *But other hospitals, the relationship is GP and consultant, it can cause a problem of resistance due to autonomy.***

**Interviewer:** What do you think should be done for that?

**Respondent:** On this program, you should not begin by saying ‘do this’ instead, this should be done based on consensus or agreement with all prescribers. I think the magnitude of exiting problem would make majority of them to agree and ***you know, if you agreed to be a slave, it is not problem, right.*** So you will ask from their side and you may find a better solution, you will incorporate it. ***If you make them feel from their side, creating sense of ownership.*** ***As to me if it is an idea that has been aroused from my side I will accept that, but if the consultant come and if he says it’s like this, probably a lot of persons might not accept that.***

**Interviewer:** So you mean ownership.

**Respondent:** Yes. You can also do this by giving trainings; you can make them feel that they bring the idea from their side by leading them towards that, what if we do this; from this angle, and by showing them the magnitude. You can make them to think about it. It will be a better way. Like if the people argue, because you gave them the guideline and tell them to follow it. If you make, the person who is trained, will later on train the untrained. It is a very long way. It is a system. You will assess, where we are now, where we want to be. There is a need of many research like that of what you are doing now. Trainings, discussions, when you make a discussions, it should not be only with consultants, because most of the time these who prescribes are resident or GP, so including from this groups, reaching to a common consensus.

**Interviewer:** OK, what if it is like this. How would you feel if antibiotic restriction policy [the interviewer briefed how restriction policy works] is applied in this institution? Do you think it is feasible and important? What are the barriers and enablers for this intervention?

**Respondent: *It is good in as an initial step. At the current situation, it might be difficult and could take much time and resource to implement the prospective audit. So this [restriction] short cut is good temporarily. This is not a sustainable solution but y***ou can use it temporarily. I am mean that person is didn’t not accept it but you forcing him to do so. So if you hold him in a rule, but don’t work on that person, not as a major goal but as a temporary. ***It will help you temporarily until you design sustainable solution.*** ***It will be good if there is a system that all agree upon. Instead of strictly ordering peoples.***

**Interviewer:** So this is what you recommend. Any final thing that you will add about resistance.

**Respondent:** This is a major issue in public health it should be done. Attention should be given.

**Interviewer:** Thanks.

=================================================================================

## Physician KI#11

Age: 47 years

Sex: Male

Department: Medical ICU [Head of Critical Care and Pulmonology Unit]

Staff Position: Consultant Physician [Critical Care and Pulmonology fellow]

Total work experience: 22 year

Number of patients treated per day: 8 patients

Number of patients with ABX prescription per day: 7 patients

Total Interview time (duration): 22:17 Minutes

**Interviewer:** Ok, thanks. So in general from what you observe in our hospital or country or nationwide, also from your private experience, How do you describe the use of antibiotics?

**Respondent:** First of all, we ***don’t have guideline*** for most of the diseases like pneumonia, sepsis, and UTI. There is no guideline prepared based on evidence of ***culture and sensitivity findings*** so you can ***guess that there will be a problem***, and we also observe where ***unnecessary medicines Unnecessary antibiotics*** like 4^th^ generation cephalosporin, meropenem ***are prescribed.***. We are trying to control them, while we are working in ICU but in ***general there is a problem of antibiotic use and antibiotics are prescribed unnecessarily.***

**Interviewer:** How about the use of broad spectrum antibiotics? Do they tend to prescribe broad spectrum antibiotic and how are they utilized in a hospital?

**Respondent:** As I have told you earlier, basically in ward and ICU, ***broad spectrum antibiotics are is used very widely.*** We don’t see it step by step to identify the etiology [which bacteria’s causing it, what kind of sensitivity pattern is there]? It’s not after assessing this but we simply make guess and immediately prescribe/we jump to prescribe broad spectrum antibiotics. It could be due to ***fear that it is dangerous***, unnecessarily, we jump to broad spectrum and antibiotics that should be reserved are commonly used.

**Interviewer:** So among the reasons, one is you said fear of infection. What do you think are the other reasons for this inappropriate use of antibiotics?

**Respondent:** Yes, ***fear.*** We fear that the narrow antibiotic may not cover what we need and thus we do not want the patient to suffer with problem, or to die. The second one ***is not having adequate knowledge.*** If we have a good knowledge it would have been good. And the 3^rd^ one is there is ***not guidelines, as well as we are not following the available international guidelines.*** Guidelines alone am not certain how much change it will bring alone, you cannot see it separately. And the 2^nd^ problem that I observe is, ***giving antibiotics for a long time***. ***Recent studies show that 7-8 days is enough, but there are 14-15 days given, now we are trying to stop this*.** And the other problem is there is ***no de-escalation.*** It could not harm to start with broad spectrum, basically when it is sepsis, if there is a serious infections, if there are systemic infections, starting with broad spectrum then, later after seeing the culture and the result/ response, we don’t deescalate. And that is ***culture and sensitivity are not strengthen enough and not giving a good yield could be.*** But most often we don’t consider it as a concept. I think which is very important for antimicrobial. They are important to decrease antimicrobial resistance. ***There is no situation to change IV to PO usually due to fear of taking risks.*** And sometimes till he fully responds, using parameter like WBC reduction, patient’s medical clinical response, by using different parameters, we do not deescalate early and also changing to PO medicines. ***So in our hospital our antibiotics practice, I think it is in dangerous. And doing so it will expose to increasing antibiotic resistances. And I think it’s a major problem.***

**Interviewer:** Is there no policies for switching of IV to PO?

**Respondent:** So till you go and say, do this, I think there is no consistent thing, or they might wait for you. They might wait the senior, I think there is a gap, because, till the attending consultant go and tell them what to do or what to change, since there is ***no similar pattern, monitoring methods and guideline.***

**Interviewer:** How do you see the patient effect in prescribing? Basically in antibiotics. Some want you to prescribe them a medicines and some due to fear don’t want you to prescribe to them or their child. How do you see the impact?

**R: *Yes, patients usually try to manipulate you.*** They will try to lift you a heavy load. But if have a scientific base, you have to convince them, Just as you said, if you think that the patient needs, and the patient said I don’t need, you have to convince him, if it is a must. But, do we prescribe for those who don’t need? Yes, we prescribe. We see both of this problems. ***There are also family influences.*** We have to convince.

**Interviewer:** How can you explain the level of resistance?

**Respondent:** There are studies in this hospital, in Gram negatives, the resistance is higher basically for gram negative bacteria’s, for the antibiotics, specifically done in here and elsewhere, there was a presentation in pneumonia, and there were persons coming from microbiology, and when they present, ***it is alarming, even streptococcus pneumonia***. ***For the commonly prescribed antibiotics, including penicillin, amoxicillin, there is a higher rate of resistance in our country.*** In different places like Gondar, Jimma, and studies done here in Addis Ababa studies done in different hospitals shows and also antibiotics do not respond early, and even when we take antibiotics if we develop sore throat, Ampicillin or amoxicillin will not respond early unless you took Augmentin. ***And I think the more we use the more the resistance will be. And that’s what is happening. Even meropenem resistance is seen with a small proportion and with pep/tazo there is a higher resistance report being observed. Basically*** ***acetobacter shows resistance for all tested antibiotics in ICU.*** ***So it has a scary level.*** ***On the other hand innovation of new antibiotics is so rare and I think we are losing the last choices of armaments we have.*** ***I think is has not been officially declared in Ethiopia about antibiotic resistance as a threat unlike others but in other countries it is seen as a major problem like in US it’s like a threat, for example when Ebola occurs, MDR TB is a threat in Addis Ababa, just like that antimicrobial resistance is a problem.***

**Interviewer:** You earlier said inappropriate use is one of the contributing factor, But what could be the other factors. Basically in this hospital.

**Respondent:** The ***1^st^ one is inappropriate use*** and the other is ***appropriate medicines are not available***. ***For example there is a medicine that you should give first [firstling drug] but because it is not available and meropenem is available you give meropenem.*** For example because, cefepime or ceftazidime is not available. ***So unavailability also.*** With an affordable price, here in this hospital for those ***patients who cannot afford*** with a fair price is also another problem. The second one is ***culture and sensitivity is not available***, and ***not available with the yield we want it.*** The other is there is ***no proper guideline***. ***And not properly using the available international guidelines, inappropriate use.***

**Interviewer:** What other contributing factors do we have?

**Respondent:** It also matters with ***infection prevention*** also because in ICU, there were a lot of person admitted. Currently it is a little bit under control, ***human traffic***. When we were supposed to finish the discussion outside and, see the patient briefly, but instead a lot of person encircle the patient, and we ***don’t either use antiseptic or water and soap***, his also contributes a lot, for this hospital acquired infections, like skin infection, soft tissue infection, ventilated transmitted pneumonia, sepsis and for different things like UTI, if we didn’t properly use catheter properly,

**Interviewer:** Related to the yield of culture, does many physicians has an experience in sending culture? And is there any complaint about the microbiology?

**Respondent:** The desired number of samples are not sent. It is little better in ICU but in other units the interest to send sample is very low. ***But to be honest, we lost hope*** ***in that area.*** If it comes, you might say it is ***contaminant because the result is mostly null, it is negative***, you are told that it has nothing, and yes may be it could be because ***patients come here after they take antibiotics***, but the ***trust we have there is very low.*** In microbiology, so maybe they have to do a lot to improves their services. And, most ***often our team working is very poor,*** from my observation. If we get closer and if we knew the patterns, it could be monthly or every six months, we could have adjusted the antibiotics prescribing based on the situations. ***But the culture of working in team is weak that should be improved.***

**Interviewer:** Do you think that the works done to decrease antimicrobial resistance are satisfactory? How do you describe that?

**Respondent: *I think it is very poor, I hear that there is antimicrobial resistance committee, Infection prevention committee, but I have never seen any visible work done so far.***  I think it is very poor because antimicrobial resistance have 8-9 packages, but I have never seen any of them implementing. When some professors are invited from other places and teach us, last time from south Africa a professor taught us same issue, for some time it will bring an effect and immediately it will be forgotten, because these are not in a systematic way. So I do not think that a good work is done so far. There is no control, for example, one of them is post prescription evaluation, or control in some specialists, and there are a lot of things that need an order, a strategies, but none of them are not available are here. ***And some antibiotics that should be reserved are prescribed here and there.*** But such efforts should not be limited here as a lot of things will be ruined outside here, clinics, and hospitals found at the periphery. It should be compressive, or in a country wide, otherwise I don’t think working only in TASH will bring a change.

**Interviewer:** So what do you think should be done to curb the problem?

**Respondent:** *Yes, guideline, audit, all kind of compressive things should be done. Otherwise, if not, I don’t think there will not be a good outcome. So the antimicrobial culture and sensitivity should be improved. There should be a team work. All packages should be accomplished.*

**Interviewer:** What do you think if restriction of antibiotics [the interviewer briefed how restriction policy works] is applied in this hospital? Would it be feasible? What are the challenges and enabling factors?

**Respondent:** It is a ***little bit difficult***, ***because, that specialist may not be found***, the 1^st^ thing is that they are ***few***, if there is enough man power if there is available person daily, and we try it ***I will be glad***. And as some studies show, ***restricting it with limited persons, I don’t think it will bring a significant change.*** ***I think more of prospective audit is better.*** I think if we have a man power and try it, in our circumstance, it may work or might not work. But in the current situation it is difficult to implement it, because there are few specialists, like ID specialists, even here. ***There are 2 persons in a big hospital in our country. For example both of them may go somewhere, so it is difficult, so it may cost a patient life.***

**Interviewer:** So for you the major barrier is availability. What other barriers and enabling factors do we have?

**Respondent: *Availability, but it could be possible to bring a behavioral change.* *Here there is a man power, relatively better from the other but still inadequate.*** So to apply the intervention, here there a suitable conditions, availability of ID The other is we can develop a guidelines, here there are relatively a better, so if you develop a guideline, and do based on this, so that we can protect those medicines, You will set a preconditions for those antibiotics, set a restriction criteria not to early use them, and if it is they need to use it, you will say consult a physician, so here it is more easy.

**Interviewer:** What do you think if prospective audit and feedback [the interviewer briefed how prospective audit and feedback works] is applied in this hospital? What are the barriers and enablers for this intervention?

**Respondent:** I think it is possible to do it. Basically clinical pharmacist, microbiologist, critical care physicians, ID physicians, and others should be included in the team. I belief a strong work can be done.

**Interviewer:** But what kind of barriers can there be?

**Respondent:** The barriers are, the 1^st^ one us there is ***no culture and sensitivity***, the 2^nd^ one is ***acceptance by the other physicians, i.e. you can face a resistance with other senior physicians***, the 3^rd^ one is ***attitude problem***, the other is ***time problem, because all cannot be available at the same time, you will observe a lot of things getting delayed***. Professionals may not consider it as their ***major role and thus there could be commitment problem*** from the team […we see this in other committees in the hospital]. It could have this problems. But I think, if we belief that this is our problem, we can do it. Not only the ID but also the microbiology and the pharmacy should work with the team. Yes, you can convince them by showing the seriousness of the problem, and I think it has an importance to work with FMOH. FMOH can help us with this area. They have an interest in this area. Last time Ethiopian society of internal medicine has raised this issue. And it also ***has written that this is an alarming conditions.*** There is also ***policy just written in paper*** as an enabling one although there is a practical work done so far. ***Another barrier is the unavailability*** of antibiotics. Important antibiotics are no available, and if that is the case, you jump to the higher ones or the lower which is less effective. This should be seen as a barriers. Drug availability.

**Interviewer:** What other enabling factors do we have?

**Respondent: It is now an agenda and** FMOH can help us with this area. They have an interest in this area. Last time also Ethiopian society of internal medicine has raised this issue. And it also ***has written that this is an alarming conditions.*** There is also ***policy just written in paper*** as an enabling one although there is a practical work done so far.

**Interviewer:** During prospective audit there is provision of suggestions to the prescribing physicians. Do you recommend to be given in orally or in a written form?

**Respondent: *Combined or both.*** Because a written thing is good because the culture we have is people deny if you don’t have documented evidences. Again, the seriousness can be recognized if you leave written document. In our country, people usually understand the seriousness if it written. But if you write it and keep it they may not read it again, so you will send the written thing and by this day, by this time, I will come and discuss with you. I think if it is combined , it will be more effective. Unless it is written, people may not consider it seriously.

**Interviewer:** Thanks.

=================================================================================

## Physician KI#12

Age: 42 years

Sex: Male

Department: Medical (Non-surgical, Cardiology) ward

Staff Position: Consultant Physician

Work experience: 12 years

Number of patients treated per day: 20 patients

Number of patients with ABX prescription per day: 2 patients

Duration of interview (minutes): 35:48 Minutes

**Interviewer:** How do you describe the current use of antibiotics in Ethiopia and particularly in this institution?

**Respondent:** The country including this hospital have no policy of antibiotics use and as a result the practice of antibiotic use is not uniform. We rely on empiric therapy which is mostly based on our clinical judgments but the use of culture and sensitivity is very rare. We know that antibiotics should be started at lower and can be continued to highly potent ones but from my experience, I can witness that the inappropriate use of antibiotics like overuse, broad spectrum use and their use for longer duration is increasing over time. **But in general there is no consistent and predictable use of antibiotics.**

**Interviewer:** Which problems are more prevalent or common in this setting? What are the potential reasons for the preference of broad spectrum antibiotics and overuse of antibiotics?

**Respondent:** As I mentioned before, inappropriate use of antibiotics increasingly very common practice but the use of broad spectrum antibiotics and over use of antibiotics are the most prevalent ones. We are observing that the consumption of vancomycin and pep/tazo, potent medicines, is so huge (in millions if converted into monetary value) and it seems increasing from year to year. ***This inappropriate use antibiotics is resulted due to many reasons to mention some like the erratic supply or unavailability of antibiotics, high cost of antibiotics making it unaffordable to patients and prescribe less important ones, poor infrastructure of microbiology leading to poor culture and sensitivity test, lack antibiogram, absence of hospital antibiotic policy leading to individual experience based empiric therapy, absence of policy to reserve precious antibiotics and lack of up-to-date evidence on pathogen susceptibility.*** I think it would be ***much better to restrict antibiotics prescribing*** at different levels especially the new and highly potent antibiotics should be preserved through hospital policy of such type. Yes, policy is important; I mean lack of strong policy and system is a major factor for increasing the problems of antibiotic use but equally the enforcement is so weak. I mean, even in the presence of policy like restricting over the counter sales of antibiotics, there is lux regulation and weak enforcement of the existing regulations.

**Interviewer:** How do you describe patients’ pressure to prescribe or not to prescribe antibiotics? What about their preference of IV and PO antibiotics.

**Respondent:** Patient pressure depends on the type of health facility. I mean if you are working in the private health facility, yes it is very common and there were times where I prescribe antipain and vitamins. I did this as I couldn’t convince my patient. Such patient demand is emanated for some reasons like one, patients in private clinic usually pay more and they think they should get whatever they ask; another thing is most of the clients of these facilities do have relatively better income and education and most of them have prior knowledge from ready in the internet or books. As a result, they wanted to challenge you. I think this all could have an influence to prescribe antibiotics but still very minimal. On the other hand, there is no patient pressure in the public health facilities. These clients are in most of the cases characterized with low socio-economic status, less knowledgeable and they fully trust the prescribesr.

**Interviewer:** what about patient’s preference of IV and PO antibiotics?

**Respondent:** few patients tend to prefer IV over PO but it is not a major issue. If you prescribe PO, patients don’t usually insist to give them IV but rather we physicians tend to prescribe more IV medication especially for inpatients while patient are in a good condition to take PO medication.

**Interviewer:** How do you describe the current status of antimicrobial resistance and its impact in your daily practices?

**Respondent:** We don’t have up-to-date data but from my day to day observations and patchy studies, I think resistance is becoming a huge threat and the evil side of it is it is increasing in an exponential manner while development of **new antibiotics is scanty. AMR alarming and I usually equate it, if not beyond, with global terrorism. The problem is now common and t**he number of patients who resistant to all available antibiotics is increasing. It a serious issue. we see many patients dying due resistance I mean we see pathogens like Klebsiella and Acinetobacter are resistant to many of the antibiotics including the new ones and patients have no choice to take. In general, AMR is affecting the patient in many ways and that by itself could affect physicians satisfaction on their service provision. ***But the good news is that from my personal observation, some of the antibiotics such as CAF which has been labelled as resistance some years back are now becoming more effective and I thin relieving some antibiotics from prescribing could a good policy to see.***

**Interviewer:** What factors do you think are contributing to increasing emergence and spread of AMR? Please explain on how commonly each of them exist in your hospital?

**Respondent:** I mean as I mentioned it before the issue of AMR is alarming. Although the exact magnitude is unknown but I believe it is highly prevalent even for the new antibiotics. ***“If I personally had surgery, I have a huge fear of AMR than the surgery itself.”*** Of course many factors do contribute to increasing resistance but the major ones are like inappropriate use of antibiotics, poor regulation, absence of national guidelines and policies, lack pre-authorization policy. ***We had an experience of pre-authorization for ceftriaxone which has been started by some group but the program failed due to lack of clear policy direction. Thus, yes preauthorization is very essential but this should be designed based on available staff and there should be clear policy on how to implement.*** Other factors like absence of local AMR pattern, local guideline, lack of IV to PO conversion policy and lack of related trainings also contribute to the problem. And finally the poor infection prevention practice should be improved if we want to decrease the prevalence of AMR.

**Interviewer:** How do you describe the utilization of laboratory findings in the diagnosis of infectious etiologies? Are microbiology lab results timely communicated to the treating physician in this institution?

**Respondent:** Our laboratory was not strong. But I think in the last few years we are observing improvements both in scope and quality, especially blood culture results. However, still it is not sufficient and there is frustration even in the findings. Although belief on culture result is improving, some of the findings which are frequently deviating from the reality and inconsistency of findings with the clinical assessment are making us not believe on the findings. We also know that the number of discs available are very limited and you may not get culture and sensitivity result for many antibiotics. The worst part is again even for those available ones, I think is problem of stock-out which I believe might be resulted due to poor communication of the pharmacists and microbiologist/lab personal and also Pharmaceutical Fund and Supply Agency (PFSA, national procurement agency).

**Interviewer:** Are you satisfied with the efforts of antimicrobial containment strategy? What possible measures do you recommend for better antimicrobial resistance containment practices to be implemented in this facility?

**Respondent:** We know that AMR is an issue at every level know but I don’t see any concrete actions being implemented. We see some research projects which appear for a while and because they are not institutionalized, then once the project terminates the activity stops immediately. So, if we want to curtail this problem, I think those which I mentioned earlier as major contributing factors for emergence and spread of resistance should be addressed well. There should be concerted efforts to improve the infection prevention practice of hospitals, training of prescribers’, developing hospital based antibiotic policy including restriction and IV to oral conversion policy. **May be as I mentioned before for CAF, relieving some antibiotics from prescribing for some time [antibiotic cycling intervention] could also be considered as one of the intervention.**

**Interviewer:** OK, if can look into one intervention method that is restriction, how do you see its applicability? What challenges do you anticipate?

**Respondent:** I think restriction is also a good intervention to decrease unnecessary use of antibiotics. I would say, go for it. **We have experience with other expensive drugs like anticancer and ART medicines where restriction proved to be very effective in decreasing the unnecessary consumption but that doesn’t mean it doesn’t have a challenge.** The major challenge I observed is the availability of experts who can authorize the prescription, which ultimately may affects the patient care . For antibiotics, the number of ID physicians are very few and I am not sure if they would be available all the time. Yes, the workload for the experts will be too much but it is so crucial. I also suspect a minimal resistance from the prescribers may be claiming as if there is breach of their autonomy. I said minimal because we see it working well for the expensive medicines, so why not for antibiotics? **But my advice is. if you need to start the restriction, then please make sure you bring everyone onboard and try to build a consensus.** So I believe if it done appropriately, restriction is a very good tool to decrease antibiotic consumption and then the problem of resistance. **The bottom line is, the focus of the intervention should be to improve the patient outcome.**

**Interviewer:** what enabling factors do you think we have to implement the restriction policy in this hospital?

**Respondent:** I think the experience of restriction I mentioned for costly medicines can be taken as an opportunity to learn, to draw lessons and design the restriction intervention accordingly. Also with teaching hospitals like ours, restriction could be easier to implement as there is an already established hierarchy among physicians (intern, resident, fellow and consultant) and this might be helpful to implement as per the hierarchy where those at the lower level can be obliged to not to prescribe the selected antibiotics unless they got go ahead from the senior physicians.

**Interviewer:** Similarly to the above, can you help understand your perception of implementation of laboratory supported prospective audit and feedback (multidisciplinary team composed of ID and/or other senior physician and pharmacists) in this hospitals. What do you think if it implemented and what challenges shall we expect?

**Respondent:** I think it is feasible to implement the antimicrobial stewardship in our hospital. The availability professionals with required expertise including ID physicians and clinical pharmacists put us in a good position to start the program. **But the challenge is it is an additional work load for the professionals and hence it may need additional fund to pay for the team. I am not sure if the management can pay them for the additional works. But as an option the hospital management can consider this as a responsible and make the team members free of other responsibilities during these working hours.** I think it is very intensive work which commitment from the providers especially from the ID physician and I am not sure if we can have such commitment in a sustainable manner. So, commitment of staff, workload, and others like narrow scope of the laboratory and frequent stock out of reagents and medicines can hinder the implementation of the program. Lastly, I know there is a policy of AMR in Ethiopia and that policy identified ASP as one of the interventions to promote prudent use of antibiotics, so the existence of such national policy could be considered as one of the enabling factors.

**Interviewer:** Thanks.

=================================================================================

## Physician KI#13

Age: 38 years

Sex: Male

Department: Medical (Non-surgical) ward

Staff Position: Consultant Physician

Work experience: 13 years

Number of patients treated per day: 20 patients

Number of patients with ABX prescription per day: 5 patients

Duration of interview (minutes): 30:42 Minutes

**Interviewer:** How do you describe the current use of antibiotics in Ethiopia and particularly in this institution?

**Respondent:** ***My observation of antibiotic use in hospitals and other setting of the country worries me so much. This time overuse of antibiotics without evidence is becoming a culture. You can see they are used like sugar or any other commodity goods. It is shocking! […..] I mean if you properly pronounce the name of any antibiotic, you can get it without prescription.*** Prescribers also tend to use those highly potent and broad spectrum. Plus, these strong agents that should use wisely are now being used inappropriately for longer duration without due attention.

**Interviewer:** How do you compare the use of broad vs. narrow spectrum antibiotics? Which problems are more prevalent or common in this setting? Why? How do you describe the pattern of this problem over time? Why?

**Respondent:** From my experience, I think the problem of antibiotic use is increasing overtime. **Besides, antibiotics are available to anyone at any level and anyone can prescribe any antibiotics.** Almost all prescribers prefer to prescribe broad spectrum antibiotics empirically. Absence of institution specific hospital antibiotics policy and guideline, absence of preauthorization, fear of infection or complication, physicians need to see a quick response/outcome are some of the factors contributing for overuse and broad spectrum uses. In addition, when patients are reaching in hospital most of the cases we found the patient already started the highly potent drugs leaving us with no choice. As a result, **we usually prescribe cocktail of antibiotics and as there is no up-to-date evidence, we don’t have confidence to change or scale down the treatment**. Thus, absence of pathogen susceptibility is also hindering us from improving our quality services which ultimately affects patient outcome. Although the microbiology infrastructure is not strong and physicians’ culture of sending samples is not as such a common, there problem of delay of findings, report of poor growth and most of all there is doubt in the quality. *We know that the problem of antibiotic is huge and it has a spillover effect. It is not an agenda of one health institution as ones inappropriate practice do affect other even they have the perfect practices. Thus, appropriate use of antibiotics should be agenda of all stakeholders at all levels. Considering its serious consequences therefore, I can say only little attention is given to the problem. As a result we are facing many problems and if actions are not taken timely we might be in a position where we can’t treat our patients. I mean, at this time I am not sure if ceftriaxone is really working.* ***We are sandwich in between getting effective treatment visa-vis proper utilization visa-vis lack evidence or data.***

**Interviewer:** How do you describe patients’ pressure to prescribe or not to prescribe antibiotics? What about their preference of IV and PO antibiotics.

**Respondent:** I don’t see the influence of patient in prescribing antibiotics at all. Patient respect whatever prescription you provide them. But I had a few experience of patients preferring to get IV antibiotics instead of PO and their very reason is that thy believe getting any IV treatment equated with getting effective treatment. The perception that IV are more effective than PO is true with few patients but that by itself doesn’t an influence in choosing the type of antibiotics I mean such patient perception are resulted due to lack of knowledge and if you try to explain to them they fully accept your justification. I have no objections from my patient in this regard and I believe this works for my colleague physicians too.

**Interviewer:** How do you describe the current status of antimicrobial resistance?

**Respondent:** It is difficult to describe in percentage or number but I personally believe that AMR is shocking and it leading us to a nightmare where we will be surrendered by microbes. I think resistance is also increasing from time to time. I know there is no comprehensive study but I am saying this from clinical point of view and from the fact that many patients with minor infection are not responding even to potent antibiotics. I think resistance is one of the major reasons for treatment failure. We also see this from few culture and sensitivity test results, where isolated pathogens are resistant to many of the antibiotics.

**Interviewer:** From your daily practices, which pathogens do have high resistance? How do you describe its effect on your daily practice?

**Respondent:** I think pathogens like e. coli, Klebsiella and Acinetobacter are the major ones. Resistance is leaving us with no or little choices to our patient and this is also manifested by high treatment failure and high mortality. And if the outcome of my service is not good not because of me but because of the problem in the medicine, this is creating discouragement and dissatisfaction among the health care providers. I think such poor patient outcome will also increase patient treatment costs, hospital expenditure and after all it could have an impact to the country’s economy.

**Interviewer:** What factors do you think are contributing to increasing emergence and spread of AMR? Please explain on how commonly each of them exist in your hospital?

**Respondent:** Although AMR is a national and global problem that need concerted effort and commitment, the level of attention and commitment to curb the problem is still so minimal. The poor regulation and absence of strong system to control antibiotic use are the major factors for increasing AMR. **You know that anybody can prescribe any antibiotic at any time for any patient. Absence of hospital based and national antibiotic prescribing policy play its role for that. This also true for dispensers where the bigger challenge lies, I mean anyone can dispense any potent antibiotic for any patient, although we know that it is against the law. But weak regulation can’t protect such illegal practices. I personally believe that availability of some antibiotics which are solely to be used in hospitals like meropenem should be restricted**. Poor lab infrastructure, lack of some discs, absence of antibiogram and frequent stock out of antibiotics are also some of the major challenges that I think may contribute for increasing emergence of resistance. Apart from this poor communication between and within healthcare professional and poor culture of working in team might also contribute for that problem. **I personally believe that team work is crucial for such kind of services, you know provision of healthcare service should be in team like a fully functioning organ system. I mean you can imagine how we feel If one of our organ system fails and I think that is the case when we are not involving all relevant health professionals**. I believe working in team is not a culture and there is an Ethiopian proverb **“We Ethiopian are very good in eating together but not good in working together.”**

**Interviewer:** How do you describe the utilization of laboratory findings in the diagnosis of infectious etiologies? Are microbiology lab results timely communicated to the treating physician in this institution?

**Respondent:** From the physician perspective the culture of sending samples is not strong but even for some of us whom we send samples, the result is not satisfactory. Our microbiology lab is not strong enough and still they have problem of getting relevant discus. The scope culture and sensitivity is very narrow i.e. they do susceptibility test for very limited pathogens and antibiotics and even those there problem in the quality. Result deliver or communication is very weak and I always wondered what will happen if there is alarming bacteria which needs immediate action. It took them several days even for preliminary results and that by itself discourages prescribers not to send samples. **At times they sends no growth or report of high resistant to specific antibiotic but by that time the patient has typical clinical signs of infection and s/he is already showing a very good prognosis with that specific antibiotics which was claimed as resistant. So, how can I trust this guys if they reporting such results. The is also big challenge in quality sample collection and very poor growth. In general, we know that the laboratory is so crucial for our practice but it should be improved in many ways including infrastructure and training of professionals.**

**Interviewer:** Are you satisfied with the efforts of antimicrobial containment strategy? What possible measures do you recommend for better antimicrobial resistance containment practices to be implemented in this facility?

**Respondent:** you can see minimal efforts to tackle AMR but I don’t believe it is enough as compared to its health and economic impacts. It is terrible problem affecting everyone in everywhere but you don’t see as such a commitment to monitor the problem. Yes, we have seen policies in paper and when we discuss with anyone including policy makers, you can see everyone being worried by it but on the contrary we don’t see concrete **actions. Even the regulatory body has some documents on rational use of antibiotics but yet the enforcement is so weak. I say, it is enough to be paper tiger, I think now it is time for Action.** The hospital should also have its own antibiotic use policy, prepare antibiogram and update it annually, strengthening the infection prevention practice, provide continuous trainings to all professionals and improve availability of medicines. **Another things, the undergraduate program curriculum of all health care professionals should be designed in way that gives more weight to antibiotic resistance and its containment strategies.**

**Interviewer:** You were telling me that any antibiotic including the potent ones can be prescribed by any physician at any level. But what do you think if the hospital come up with a restriction policy where selected antibiotics would require approval of ID physicians prior to prescribing but otherwise will not allowed to prescribed. How do you see its effect and what do you think would be the challenges, if implemented?

**Respondent:** I think it can be effective policy if designed meticulously. Of course there might be resistance from prescribing physicians but we don’t have a choice and we have to do immediately. This resistance of frontline staff might be emanated from individual’s interest to protect their autonomy but I think this mentality has to be changed and make sure that every decision is made in the best interest of the patient. Serving the patient at our disadvantage is something well-articulated in our professional oath and we have to comply to that. Apart from resistance from staff, other challenges might the continuity of care especially during weekend, holidays and night shifts. I mean unavailability of the experts to consult for 24/7 might be a big challenge; we also know that we have no or very limited number of specialists (ID specialists) especially in the non-teaching hospitals. On the other hand, initiation time for antibiotics has great consequences and a delay due to their unavailability might cause chaos in the hospitals. So to avoid those unwanted consequences to the patient, concerted efforts should be made to design a strong system that solve such problems.

**Interviewer:** what enabling factors do you think are there to implement the restriction policy?

**Respondent:** These days there is a good attention to antibiotics especially from policy makers. I think the concern that AMR is threat (both from policy makers and providers) is an opportunity to get strong support to implement. **“I personally can tell you how much I am afraid off resistance especially to my children since I am rounding in congested hospital with many patients identified as having highly resistant pathogens and I can assure you that this kind of concern and attitude is growing among many physicians.”** So I think if you came up with such an idea and convince them the intervention decreases AMR, definitely the acceptance will be very high. Also our previous experience on few drugs showed us it is possible to do the same.

**Interviewer:** OK, there is also another type of antimicrobial stewardship intervention i.e. the prospective audit and feedback by a multidisciplinary team composed of ID and/or other senior physician and pharmacists. So what do you think if this is implemented in this hospital. Do you think it is feasible? What do you think are the challenges and enabling factors?

**Respondent:** I think it is feasible. Previously I said health service should be organized like our organ system and thus team work is something that we should nurtured in the healthcare system. I believe everyone has something to contribute. The nature of the work which requires much time of the physician’s time and at the same time inadequate expertise in infectious disease might be some of the challenges. Another thing it might be very difficult to bring different professional and make them work together as such culture doesn’t exist in our set-up. I have also doubt on the level of training of pharmacists but I think such gaps can be solve through trainings. As it is lab supported program, the poor infrastructure of the laboratory or the poor capacity of the laboratory to do culture and sensitivity test might also be one of the problems you will encounter.

=================================================================================

## Physician KI#14

Sex: **Female**

Department: **Pediatrics**

**Interviewer: How do you explain about antibiotic utilization?**

**Respondent:** I was learning here, so when I observe it now a days, our antibiotic utilization is more to the new coming ones, it focusses to the new arrivals, probably it could because the previous one doesn’t work, for pneumonia, ceftriaxone is given by skipping amoxicillin, without having an evidence, it encourages us to use them. The patients that are sent in the place where I work, since they are a critical patient, and by taking the risk, but, penicillins are aside and cephalosporin’s are common. And currently carbapenems are very common. There is shifting to those.

**Interviewer: While observing, do you think it is increasing from time to time.**

**Respondent:** Yes, I think it is increasing.

**Interviewer: Is utilizing of broad spectrum observed. And since you oversee all of them, like for a short duration long duration of treatment.**

**Respondent:** Here related to dosage, in our setup, there is something that we say renal adjustment. Here critical cases arrive. And this renal adjustment varies from person to person. If we don’t have something common. The first one is this. So sometimes, because of we say that they have a renal injury the antibiotic that can work will be avoided. As I have told you we use broad spectrum and highly effective. So, the first one is that. And he second thing is, related to duration, deescalate, they don’t need to decrease the duration. Duration 10 days 14 days, even if they are fever free, if they are responsive, there is no situation that avails the result early, what is the positivity of our culture, how is our confidence on that aspect, 10 - 14 days. Here in ICU we argue till he finishes the antibiotic to 14 days. So, related to duration, I accept that. Everyone follows their own steps. Dosage, basically in our setup, newborn dosage, even as to me dosage for malnutrition should be done. Now your focus is on antibiotic, for example when we give benzo diazepam, and we observe a dramatic depression. And sometimes we observe nothing. Even for malnutrition, how do they respond to the drugs should be seen. In ICU setup, renal adjustment, and duration.

**Interviewer: But as you we have stated earlier, for the utilization to be poor, you have said the culture and sensitivity test, confidence, but what any other problems could there be?**

**Respondent:** The first one is there is no common diagnostic criteria, there should be a means of communication, protocol, if you need to do any quality improvement, we need to agree on the diagnostic criteria’s, there may be some exceptional, for our hospital this is the diagnostic criteria, from what we have, we have to do this, we have to have a means of communication, all of us. Because we don’t many have ID specialists, we are coming in to…. (00:5:58). we might have no experiences. The other is our laboratory utilization. How is our laboratory quality, so the interns, the nurses, the residents, what does the microbiology lab needs, for instance collecting the blood culture, the producer, the producer is this, contaminant, we sometimes sent tracheal aspirate, and they say what is the use of this and it will be returned back. It shows you the flora, so what is the use if this and it will be returned back. Really CSF culture will be sent and, the time of inoculation is not known. Was is sent…. .(07:04). so, the lab also, needs its own guideline, and all stakeholders should now that guideline. …. (07:20). the 3^rd^ one is the antibiotics, which one we shall preserve, I think they shall be preserved. Since they are available, I think we shouldn’t use all of them, if we reach to that stage. So, it is important, we have to agree our diagnostic criteria. For ventilated associated pneumonia, if he gives peptazon…. (07:59), and if I say why do we go there, let’s try cefepime. It should not be like that. It should not be personal. This is the gap we have and it should be this way. If it didn’t respond to this we have to go up to this… (08:19). There should be something that says like this. There should be grading. Before we reach to that, we have to do about prevention, most important. Hygiene. We have to do on the things that we can avoid. Because we know antibiotics are not coming… In our 3^rd^ world, we known the cost. Antibiotic stewardship…., it is better or quite important to do in infection prevention. And I personally say the 4 interventions.

**Interviewer: As you see from your students, is there a patient influence in prescribing antibiotic or not to do so.**

**Respondent:** It depends on my working setup, when I say working setup, here usually comes a trustful community, they accept what you said to them, and there will not be that much push. Their literacy might not be that much. Actually, it is changing. Here there is no pressure. But in private set up there is. In private set up there are persons who feel discomfort. So, you need to prescribe something. Vitamin or whatever. I think the private thing is different, even if you know when it is common cold, and it is severing, it is not let’s observe it for the coming days, you just give it.

**Interviewer: Do they prefer injections,**

**Respondent:** No, unless the vomiting disturbs them they don’t need injection,

**Interviewer: For a reasonable thing,**

**Respondent:** In children’s it is not common may be in adults I don’t know,

**Interviewer: How do you see the resistance of antibiotics from time to time?**

**Respondent:** I might not be a good example because I am working in intensive care or emergency unit, since am not active in OPD, but it is active the colonization, 2 days 3 days, … (11:34), it might come to you like resistance to all of the drugs, only … (11:41) sometimes you will be in dilemma, shall we treat it or not, so that’s why I say we have to preserve some of the antibiotics, sometimes klebsella, acetobacter… I think extended beta lactamase is not done in here I think. From the pattern that we see, and sometimes if we go back to penicillin, can it work,

**Interviewer: In some countries, recycling strategy,**

**Respondent:** Go back to the previous ones like CAF,

**Interviewer: DE cycling you have to give relief, like if over use increases resistance also increases. Related to lab…..**

**Respondent:** “MRAS” I don’t know, we are scared of it, but it’s not that much, the Gram negatives are in a Very scary situations,

**Interviewer: It’s because supported by literatures,**

**Respondent:** Yes, that’s what I say, but it is not that much

**Interviewer: But how much does it have an impact in your work, basically in your work, if this resistance come how it challenges in your work,**

**Respondent:** In the patients that we admit a child as a sepsis, we rarely become successful, to get them out of it. Actually, I don’t think its antibiotics, early recognition, transportation, health belief, there are a lot of factors. Here most of them are multi-organ failure, I am doubtful if it is the really the antibiotics only, Sepsis is one of the killers, in the ICU… (15:16)

**Interviewer: What do you think are the causes for resistances?**

**Respondent:** Unnecessary prescription, in private setup, there is no agreement in the diagnostic criteria, for example for pharyngitis high dose of amoxicillin for 10 days, people prefer to keep it azithromycin 5 days, so pneumococcal …. (16:14), and the other one is ease of administration of drugs, instead of giving TID, they prefer once per day, it will be tolerable highly, formulations, either more potent or easily, not to stop in the mid time (Metronidazole and tinidazole). That contributes, because it has an impact adherence, poor adherences. Majorly this 3

**Interviewer: on lab how is the habit of the physicians to send to laboratory.**

**Respondent:** when I was undergraduate; I have never seen a nurse collecting for culture, any culture we used to do it. Intern resident, a difficult situation, we properly clean it, the area, the bottle, we apply iodine, but currently phlebotomy most of the nurse do, related to pediatrics, the adult they previously do it, but there I no problem, here basically a lot of physicians. The work place should be considered, because a physician cannot do all of the things. When there is one physician, a team work is needed. So here most of them are students so they will take this practice out there. So, related to lab, it is a plus to actively involve the physicians. A correct sample, how do you transport it, (19:36), organisms, so some of the guidelines are needed?

**Interviewer: Why do samples not sent, is it because they are not confident on the quality,**

**Respondent:** Here it will be sent. A lot of culture will be sent, I don’t know they say to me it is sent.

**Interviewer: On the result,**

**Respondent:** We need to see it, as a quality parameter

**Interviewer: As quality parameter, what is there view?**

**Respondent:** For the last one year, since your study started, hospital acquired infections, there is some change, as well as a result, and they and they say fungal is growing, and I say is it from us or you. We are somehow being more confident. It needs a constant communication, now the lab says this comes from your and it was very interesting to us. The lab monthly or every 3 monthly the resistance pattern, which pathogens were seen in our hospital aggressively, alarm, so we can act, hand hygiene, we need to reactivate,

**Interviewer: Anti-bio gram will be created. To prevent resistance in the hospital, and in the country,**

**Respondent:** It is no enough. When we start from the infection prevention, alcohol, glycerin, and next to that, how do we sterilize, instrument, disinfection. If they are washed, if we culture them, we might find something, even our ventilators, when is the day of their filter changed, we don’t know, for the day of their bought they are working, nonstop. Laundry, guan mask is not available, segregation is not available, it is better, but it needs a neat work, even to protect themselves. The setup is difficult.

**Interviewer: So, these strategies are not available**

**Respondent:** So, these strategies are in paper but they should be implemented. The antibiotic usage may decrease. Transmission based, why do we have ventilated associated. Antibiotic prescription it will be better if it is based on guideline, if antibiotics aside from that is prescribed, an alerting system, like, why meropenem is prescribed.

**Interviewer: Restriction (some explanation about Restriction)**

**Respondent:** I will say go for it, because it should not be a break on the work, the system should be 24/7, make it a system based, if you pour it like incentive based it will go down, make it system based, because I feel it, if you wear the guan,

**Interviewer: What could be the barriers**

**Respondent:** It needs commitment, commitment from staff, pharmacies from all of the upper bodies, department heads commitment from all of us.

**Interviewer: prospective and feedback (Explain about prospective and feedback)**

**Respondent:** It is good, what I say you alert is. It needs commitment

Thanks.

=================================================================================

## Physician KI#15

Age: 39 years

Sex: Male

Department: Pediatric Ward [ICU]

Staff Position: Consultant Physician

Total work experience: 13 years

Number of patients treated per day: 25 patients

Number of patients with ABX prescription per day: 20 patients

Duration of interview (minutes): 19:31 Minutes

**Interviewer:** How do you explain the use of antibiotic our hospital or nationwide?

**Respondent:** This is a difficult question. Related to antibiotic, currently we have a lot of antibiotics. Antibiotics which are good, potent, broad spectrum, there are also very expensive antibiotics coming. Including those that come recently like … (00:00:44). But in terms of the utilization we **don’t have a guideline*. Most of us, from what we read, or from what our instructor or our senior previously uses, most professionals uses. So prescribing practice is highly subjective and consulting ID physicians is very rare. We simply prescribe based on our personal judgement and things we learn from our seniors but you know that could be changed over time.*** Apart from that anyone prescribes antibiotic. Nurses prescribe, GPs prescribe, and HO prescribe, including other senior staffs. And sub specialists prescribe. And some pharmacists give without any thing. Like over the counter drugs, we know that there are places where antibiotics are sold. Generally, this is our antibiotic utilization. Related to BLH, What we do in BLH is as much as possible we consult senior physicians, residents are the one who prescribe at first, and then decisions are given at rounds. But what it is that there are situations that will be subjective. We do not have guidelines for infections, for example we do not have guidelines for pneumonia, no guideline for sepsis. Still we treat from what we read and some from the suggestion of senior physicians. Infectious diseases specialists are not that much engaged. May be they don’t have time to consult for each and every cases. If we have a protocol or guideline it would stop majority of the problems.

**Interviewer:** From your observation the utilization of antibiotic and utilization of potent antibiotics like broad spectrum (00:03:04)

**Respondent:** There is no guideline but, for example how to treat HAP, ventilator associated pneumonia there are western and textbooks. We use them.

**Interviewer:** Will there be inconsistency.

**Respondent:** Yes might be no consistency. But more or less we use a potent antibiotics. Like more of (00:03:39) cefepime, vancomycin. This are the usual drugs we use.

**Interviewer:** As you have said me there is no guideline, which makes us not to use the medicines. What other things are there that do you think there is in this hospital not to utilize the antibiotics properly. From prescribing up to dispensing.

**Respondent:** The 1^st^ one is there is **no guideline,** ***lack of proper training is also one of the factor***. Training in a sense is, our physicians; will learn, they will read. But to make it consistent, updating, plus maintaining ***the hierarchy***, for example while managing sepsis, either you have to consult Infectious diseases or critical care physicians. When you are treating pneumonia, either infectious diseases or pulmonologist should be consulted. Trends like this are needed. Probably it is because of the number of health professionals subspecialists. It is not consulted that much. I think this could be one of the factor. In dispensary I do not think there is a lot of problem in this hospital.

**Interviewer:** Because it is a government.

**Respondent:** Yes, because it is a government. But outside I think there will be. But I cannot be certain tell.

**Interviewer:** As you have said there will be over the counter.

**Respondent:** And the other is there is no enough studies. It could be due to lack of infrastructure and capacity. For example, a year back I want to conduct a study in related to ventilated associated pneumonia, and I could not do it, because of issues like there is no fund. And I have to do a study in another area. And things like this can be a reason. We do not have an evidence. Like what does it look like? Like do we have an anti-bio gram? No we do not have an anti-bio gram. Very few studies are done, for a studies like, which bacteria’s or etiologic agents are commonly seen, for which antibiotics are resistant. And it is difficult to use them by generalizing.

**Interviewer:** The aim of this study/project as you know is to after having an input including this, and we will see the 12 months of the antibiotics pathogen and susceptibility will be seen. Anti-bio gram will be done. Then evidence based. And the 2^nd^ one is, is there is a patient influence in prescribing from your experience.

**Respondent:** In this hospital there is no. In private there is a lot. And basically if they are a bit educated, they need injection, they do not need medication PO, and if they are youngsters, they rather need to take antibiotics injections than PO. I see this kind of influences, in private.

**Interviewer:** What does the resistances look like. There may not be culture and sensitivity.

**Respondent:** currently we do have culture and sensitivity. In ICU’s we take culture and sensitivity like blood, urine, tracheal aspirate. The results that we see, even antibiotics that we use …(00:07:42) and recently used antibiotics, are becoming resistant including , (00:07:47). … It has been not more than 2 or 3 years since it is introduced. now we got significant number of patients with resistance. When we go to ICU, there are some etiologic agents for example Acinetobacter, klebsiella, coagulase negative staff aureus, and at some time, they predominately isolated and becomes MDR. This is very common specially Acinetobacter.

**Interviewer:** So Acinetobacter, and klebsiella are more (00:08:35)

**Respondent:** Yes, Exactly

**Interviewer:** Okay I think this most are found from blood.

**Respondent:** Yes, from Blood, Urine and tracheal aspirate.

**Interviewer:** on practice what kind of impact does this shows? How many of them are (00:08:55)

**Respondent:** Oh it’s very difficult. Sometimes, you will have a patient with persistent refractory sepsis. And when you have to isolate like this it is difficult to manage. So what we do is. Most of our patient took every antibiotics. Every antibiotic including (00:09:17) vancomycin and the like. And they took …. And becomes resistant then later, what we do is search for … in the city. They cannot afford it. It might not be available, if found it may it is expensive. It will be very challenging, very challenging.

**Interviewer:** Okay, so related to antimicrobial resistance, what is the culture or trend of the physician of utilizing of laboratory.

**Respondent:** Previously we don’t rely on the laboratory, like most of the time there is a negative result, there was no microorganisms detected or growth and the like were reported. But now we are seeing a good result and recently there are good results especially around ICU, in a patient with sepsis, in a patient with unexplained fever, in a patient with lung infiltrates or …. (00:10:28). we always send. After we managed it, and after 3-5 days, and later on if fever persists we repeat blood culture. So currently we are utilizing it very properly. But previously it was like this. And some results explain that our laboratory is improving.

**Interviewer:** So there you have a confidence (10:55) since it is capturing

**Respondent:** A year back Norwegians team were here and there were making a round with us in ICU for some time. We discuss together. If there is an organism detected then they come and tell us, they also teach us. There were things like this and how there is a good change in microbiology.

**Interviewer:** In the quality

**Respondent:** Exactly.

**Interviewer:** But what do you think could be the cause for resistance

**Respondent:** In my opinion, sometimes we over diagnosis. We over treat. And the other thing is our infection prevention practice is poor, very poor. It is an issue that is not solved even after a guideline is developed, also after training is given.

**Interviewer:** So this are the major things that you think.

**Respondent:** Yes this are.

**Interviewer:** So the final question, is how you see about the effort to decrease resistance.

**Respondent:** It is not enough. I am a temporary head of the quality, head of the quality in this hospital. And the head of ICU. We are trying to improve it, by discussing with the responsible body in the hospital, we also discussed with infectious disease specialists physicians. Infection prevention committee is also established. We are also discussing with the committee. But it is not enough. Now the hospital bought alcohol, to prevent infections, alcohol around 2000 liters but is almost finished.

**Interviewer:** There are interruptions.

**Respondent:** Yes there are interruption, but it is fine since there are also some starting. Related to ICU, we have … an infection prevention protocol. Give trainings to the nurses, to make health professionals engaged and we are trying our best for the practice. Other than that we are trying to make responsible specialists physicians to be involved in decision. For example, pulmonologists like on pneumonia, Infectious diseases specialists and critical care physicians in ventilated associated pneumonia and sepsis and sometimes we may need their expertise, even in ICUs patients we consult Infectious Disease people. And there are also other trails, like hospital clinician director, the committee that established infection prevention and to let thus led by Id specialists. For every treatment we are trying to have protocols (It is on trial). Again specifically on ICU, to have an ICU treatment protocol, pneumonia, sepsis protocol. Even if we are very few who works in ICU, among ourselves still there could be variations, and we are doing a work to have a protocol, guideline.

**Interviewer:** what do you think will be done?

**Respondent:** As to me, an institutional study, anti-bio gram, an infection prevention protocol which is institutional, treatment protocol. Like specific diseases protocols. And microbiologist, infectious diseases specialists, pharmacist, and the hospital also the clinical director may be the quality bureau should sit down and discuss on this issue. How to improve the quality of the care we give, and how to prevent drug resistance, surge of drug resistance, so discuss about this issued and gave some thing. and something that guide us.

**Interviewer:** One intervention by the way ….. There will be an Id physician, senior consultant physicians, pharmacists, and microbiologist. And then a bigram. So the audit and feedback. For example if I am s junior physician, on the medicine that I prescribe senior physicians come and give me a feedback so how do you see it.

**Respondent:** That is important and it should be regular. Every month, every 2 month we need to come, sit down and see ourselves and get feedback and give feedbacks also.

**Interviewer:** It should be consistent, but do you think there will be no barriers. When it is implemented what kind of barriers you anticipate.

**Respondent:** Barriers can be, time constrain, when a person is given a feedback they may resist, we don’t want to accept negative feedback, we don’t want to be said “correct it”, we just want to be said good, great… I think this can be one barrier. May be finical or economically.

**Interviewer:** Is it from the one who works there.

**Respondent:** Yes,

**Interviewer:** From making it incentivized, I have caught 3 thing and they are; from time, it could also being resistance in nature, and can it be autonomy, for example a junior physician

**Respondent:** Yes they could think like that. But here the hospital is an institution, it is more than an individual. The institution is for the country, it is for the community, so I think the benefit of the community is better. And if that individual improves**, it will helps him to be a better professional tomorrow. I don’t think autonomy will be that much a problem. As my personal view.**

**Interviewer:** If it became a policy. Finally, restriction, for example highly consumed vancomycin alone has a cost of 3 million. And we have seen utilization of antibiotics of >12 antibiotics, so which antibiotics were highly consumed and if there is a restriction in every stages,

**Respondent:** That’s is a very good idea. For example many patients from the outset starting from emergency came here after they started meropenem, vancomycin. And sometimes infections that you can simply treat them with simple PO fluoroquinolones and cephalosporin. Can be difficult to do so.

**Interviewer:** So what do you think the barriers are?

**Respondent:** One thing is still autonomy issue, the resident think that they know and they feel that they are the one who should prescribe.

**Interviewer:** Thank you.

=================================================================================

## Physician KI#16

Age: 30 years

Sex: Male

Department: Medical (Non-surgical, GI) ward

Staff Position: Consultant Physician

Work experience: 6 years

Number of patients treated per day: 10 patients

Number of patients with ABX prescription per day: 3 patients

Duration of interview (minutes): 26:01 Minutes

**Interviewer:** Ok thank you. So, first how do you describe the current use of antibiotics in Ethiopia and particularly in this institution?

**Respondent:** With regard to antibiotic utilization, we can see it in different perspectives. The first one if, from the general antibiotics that we use, which antibiotics do we commonly prescribe, how many of them are prescribed correctly. Second, from experience which antibiotics works and which doesn’t work, and most of time we ***prescribing antibiotics is not based on an evidence***. The 3^rd^ one is ***supply issue, availability***, meaning which antibiotics are available in our country, and also there is ***knowledge gap on which patient should be treated with what kind of antibiotics.*** So in relation to that, there is a ***tendency to use the 2^nd^ level or 3^rd^ level antibiotics***. So with the first case, ***commonly antibiotics are prescribed for most patients.*** In the inpatient currently ceftriaxone is usually prescribed to treat infections and in the 2^nd^ level when there is a serious infection vancomycin, ceftazidime are being prescribed. But recently ***carbapenem are frequently prescribed.*** This is related to inpatient. When we go to outpatient there are 2 major commonly prescribed antibiotics, the 1^st^ one is Augmentin, and the 2^nd^ one is Ciprofloxacin. Th***ese are commonly since they are broad spectrum, it has a high utilization tendency.*** From my experience failure of ceftriaxone in inpatient is raising recently. Some, few people who are around academic areas uses the local evidence and there is an effort trying to make decision upon that. ***Even if it is like that, most of the antibiotic prescribing decisions are made from experiences or by sustaining the practices, like by sustaining what people did it.*** So there is a big gap. That is what I see. And the 3^rd^ one, what I have said earlier, availability issues, when ceftriaxone fails, what should be prescribed, when trying to intervene that, ***available options are limited in Ethiopia***, probably there is a huge gap between the medicines that are available in books and medicines that are available in Ethiopia. So it is good that carbapenems are available. However, some other important antibiotics such as fluoroquinolones are still ***not available and*** this has a huge impact in our daily practice.

**Interviewer:** You were telling me that unavailability of medicines and lack of evidenece are contributing for inappropriate use of antibiotics. What other factors do you think are contributing for that?

**Respondent:** If we see is from the whole health system practice, ***there is a huge amount of antibiotics abuse in private setups as compared to the government hospitals***. This is because, whether the patient has an infection or not, but they have ***little suspect, they blindly or roughly prescribe antibiotics in order to reduce risk of liability from the unexpected consequences. They wanted to maintain the reputability of their hospital as good.*** For example, if there is a confusing situation to clearly identify heart problem or pneumonia, they wanted to roughly treat broadly b/c they wanted to avoid any possible risk. There is over prescribing behavior, whether it is available or not. I believe that Ceftriaxone, then ceftazidime, vancomycin, and carbapenem are highly abused at private facilities. And in some places even if they are few, there are some facilities which ***prescribe expensive antibiotics to increase their income.*** So this practice starts from small health clinics to larger health facilities indicating that the ***patient are being exposed to antibiotics*** ***at an early stage meaning at the lower health clinics.*** And that have an effect in resulting culture negative or it increase the selection pressure of resistant pressure. So it is difficult to follow treatment based on that. So it will be a must for us to give a treatment that is not based on evidence and culture. I see that as a major problem. Next to that, ***poor microbiology service is one reason for that bad practice***. There is a big gap in the microbiology. If that has been done, I think we will gate a good support. But I think, it has a big gap in our antibiotic use. The other in terms of gastroenterology, we the basic reason we commonly use antibiotic is H. Pylori infection. The threshold of treating H. Pylori infection is very low. ***Many people are being treated easily for abdominal complain***, and most people when the abdominal pain didn’t stop to use antibiotic more frequently. And that, the effect is systematic, it will be resistant, and selecting for other bacteria’s too. I see that as a gap.

**Interviewer:** How do you describe the influence of the patient to prescribe or not to prescribe antibiotics to them or their family members?

**Respondent:** ***Yes, such practice is common in private facilities but in the government it is less often. In government places most often patients obey what their physicians say.*** In private places it has a little pressure. When you see it as a general practice, this pressure is usually from fear i.e. if someone has viral pharyngitis, although antibiotic has worth to it, the patient has a tendency to influence you to give them antibiotics. But in governments facilities this is not a problem. The tendency to prescribe ***IV antibiotic is usually with the intention of avoiding or reducing a risk***. This is practiced b/c one antibiotic are available; there is no liability from or will not be blamed for prescribing it. Plus it is not a must to confirm that there is an infection or not. So the easy way will be to simply prescribe an antibiotic.

**Interviewer:** How do you describe the current status of antimicrobial resistance?

**Respondent:** This is important. Especially in governmental facility, big facility, this problem is seen in an ***enormous amount***. What I have seen in my practice is, ***Ceftriaxone, a commonly used antibiotic, has higher resistance.*** It is higher on gram negative bacteria’s. studies are indicating that ceftriaxone effectiveness is not more than 30% and ceftazidime that we prescribe on the next place, has similar resistance profile. Wrongly we repeatedly say, if someone doesn’t respond to ceftriaxone we shift to ceftazidime but in practice I don’t see it working effectively or becoming successful. Plus cephalosporin has a resistance and it is observed that the patient doesn’t respond. So following this, the diseases will exacerbate or the patient will dies. Hence from such experiences, physicians who see this thing happening will do; (1) they tend to prescribe carbapenem like meropenem, or use IV fluoroquinolones like using ciprofloxacin IV and it not, they prescribe Gentamycin. Some physician do like that. It is done in few place, but it works. Using other class of antibiotics it works. ***The gap in between is that, there is nothing to view that, which bacteria resists.*** There is a ***gap in changing to another antibiotic.*** But patients in between will suffer, that is what I notice. But in some studies I have contributed, 5 years back in 2012, data’s then shows that, basically gram negatives, in ICU and ward in TASH they ***were highly resistant to*** both ceftriaxone and ceftazidime. There is a problem of utilizing the available data. A master’s thesis was done on it. It was done as a big international collaboration. I was there when this studies were done. I was there when the results were presented. But little action was taken for that evidence. There is a gap on the professional’s side. But consistently, results shows that there is a resistant on cephalosporins. It is better to focus on other classes, still vancomycin is good for gram positive, but as to me in gram negatives, if someone doesn’t respond, it is better to move to another antibiotic, because, ***I think we already lose them, basically at hospital level. Resistance is also increasing over time.***

**Interviewer:** But which pathogen are common?

**Respondent:** Here when we say gram negative***; E.coli, and klebsiella*** are very common. This two technically E.coli, and klebsiella. When pneumonia culture was positive, this two were common. ***E.coli, and klebsiella as a major resistant organisms***. The other organisms are in a small amount.

**Interviewer:** What do you think are the causes for resistance to increase.

**Respondent:** The first one is misuse, antibiotic is being abused at all levels of the health facilities. Since it is abused, the first one is that resistant organisms are selected. The second is, infection prevention practice in our hospital is poor. There are changes from the previous one, but still infection prevention is poor, I see this two as a major gaps. I think there will be a change if work is done on this two areas.

**Interviewer:** How do you see the utilization of lab, for example sending a laboratory requests.

**Respondent:** What I told you previously, my presentation focuses on this area, what I tried to see as a practice was, how much blood was collected and sent. As a guideline there should be a minimum of 10 ml blood collected after antibiotic taken….. (13:38). we can see it with 2 sides, because this is what I witness, a microbiology masters student, he was the one who collected blood, he also send the blood, and conducted a masters study, in 2012. And in his study most results consistently have a positive culture, he told us the result, and based on that result, we gave medications and the patient respond well. We had a good time in 2012 the whole year. He shows us that a culture can be positive. Even in the previous culturing system. But it is not available with the physicians. Culture might be drawn by the nurse, if she collects 2-3 cc and inserted in the bottle, we can’t identify how much it is***[quantity].*** Problems like that ***[sample collection problem]*** occur, and the second one, there is ***no support from microbiology***. They don’t consider themselves as member of the treating team. ***They don’t totally report the result or if reported it is delayed.*** In other countries, if culture has some growth with ***24- 48 hours, they report it immediately. But in here there is no report even in the 7^th^ day***. If gram stain is positive there is no report, But at that time the student was doing that. So from my witness, if it is done properly, it is certain that the results will be positive. Support from microbiology, and considering themselves, as a team, and provision of feedback if there is more negative results should be considered. From the physician side, sending a sample in a proper amount and proper time is critical. I think, this are the big gaps. Results should also be communicated timely.

**Interviewer:** How do you see the trust of physicians in the quality of the microbiological results?

**Respondent:** In general, there is no trust in the result because I have witnessed this from the student but even before I had no trust on the result b/c you see results are ***consistently negative and sometimes, when you send a pus it is negative which is true infection, you don’t know where the problem is***. When you send a sample that you are certain, it is an infection, and the result says negative, then you will start to become hesitant and loss your trust. So that a tendency of not to send will be observed.

**Interviewer:** How do you explain the effort to prevent antimicrobial resistance in hospital?

**Respondent:** I know there were a projects to look at the pattern of antibiotic resistance, and by then there good resource and professionals who collect the sample, but I don’t see this projects bringing a fundamental change in the system. Still there are a gaps. To change the fundamental system, there should be a strong persons who can persistently change the situations.

**Interviewer:** What kind of intervention do you recommend to decrease antimicrobial resistance?

**Respondent:** The first one with a short period of time is, to have a good microbiology culture practice and result. The second one is to control antibiotics meaning we need professional who can work the stewardship in hospitals. So the first step to correct the culture result.

**Interviewer:** As a tool to tackle AMR, there is what we call it prospective audit antimicrobial stewardship program [interview briefed how prospective audit works], how do you feel if this is implemented here?

**Respondent:** I think it will be possible/feasible to implement.. Because auditing would be helpful to know what is exactly happening at the ground. As it is fact based practice, I think it will take us one step further. It will help them to know what the junior physicians are doing. If constantly feedback is given, it will also change their practice. So stewardship is important. ***Carefulness should be made not to take over their autonomy.*** ***During autonomy, if you say you can’t prescribe this, other person can only prescribe this, probably, and it can cause a problem in autonomy.***

**Interviewer:** Ok, what other barriers in addition to autonomy?

**Respondent:** From physicians i.e. ***resistance; autonomy,*** the 2^nd^ is, if ***feedback is given, there will be inertia, to change it to practice.*** It is natural, there will be less tendency to adopt to new practices and change practice. And from ***administration audit and recommendation, when unavailable medications are told to avail them, and there will be some resistance***. ***Cost will increase, and there will be an issue to get medicines available early, since cost will increase***, there ***will be barriers related to finance.*** The other is, in the long run, if they didn’t see the results, in the utilization of culture and antibiotic, there might be pessimism. **In the physician side. If all those effort has been made and is not taken to the lower level or other facilities, a question might be raised about its use and there might be unacceptance.** Most often it is prescribed in private hospitals, and clinics. This is one of the barrier, Fear of anticipating that it will fail, they will not participate or adopt. I will see this as a problem. This are what came to my mind now.

**Interviewer:** The second one is restriction (Explain about restriction)? How do you see its feasibility, will there be barriers,

**Respondent:** ***This is a difficult situation, an autonomy issue will be raised***. And while treating a patient, ***patients who need to immediately start antibiotic early, and those who need to have a decision early, it could affect a patient, it can cause a problem***. I see that as a potential problem. When there is a conflict of who should prescribe, if the situation gets harder, sometimes it can also ***be a start for a conflict.*** And the 3^rd^ one is the ***pharmaceutical company.*** If pharmaceutical company brings antibiotic, he needs to sell a lot and they might try to bypass the barriers using different mechanisms. ***And the 3rd one is private facilities it is not possible to regard barriers.*** It is difficult.

**Interviewer:** What do you mean when you say private setups?

**Respondent:** For example in private hospitals, ***you cannot say you cannot prescribe vancomycin.*** There is one physician. ***The problem is in private hospitals there is no check and balance. If he wants to order the antibiotic what he wants there is no check and balance.*** In academic institution, senior physicians, in presentation, audit, there is no one who checks it so, that gap will happen.

**Interviewer**: That is the end of my interview, is there anything you would like to add?

**Respondent:** There should be consensus before implementation. Vancomycin is commonly used blindly for infection coverage by default but we know Vancomycin is inherently resistance to gram negatives. For E.coli and klebsiella, they are inherently resistant Vancomycin, we know it will not work for gram negatives. Just in case if there is S. auras organ positive infection, Vancomycin will be prescribed. If culture result is available and if there is an infection and the culture result it is possible to automatically stop Vancomycin use for such cases. If there is no culture this may take us to 10 days and the cost will also increases. So the critical step is to make culture based. Another issue is, restriction of already common prescribed medicines like Vancomycin is difficult but this might work for new arrival antibiotics.

**Interviewer:** Thanks.

=================================================================================

## Physician KI#17

Age: **43 years**

Sex: **Female**

Department: **Gyn/Obs**

Staff Position: **Consultant**

**Interviewer:** How do you describe the current use of antibiotics in Ethiopia and particularly in this institution?

**Respondent:** Antibiotics use is high and selection of antibiotic is determined by level of knowledge of the physician. Specially relating it to mother’s health, there is a fear of death associated with infection so antibiotic is used as prophylaxis during pregnancy, child birth and after delivery. Plus, the antibiotics used are broad spectrum antibiotics. Urinary tract infection on mothers has to be treated with antibiotic because it may have effect on the fetus. The other is mothers with HIV/AIDS if they are present with urinary tract infection or skin infection antibiotics are prescribed. At this hospital most mothers give birth with surgery so to prevent operation site infection antibiotics is given as both prophylaxis and after surgery

**Interviewer:** Do you think inappropriate use of antibiotics is a problem in Ethiopia and in this institution? Which problems are more prevalent or common in this setting? Why? How do you describe the pattern of this problem over time (Is it increasing, decreasing or stable)? Why?

**Respondent:** Most antibiotics are used for short period but combination of antibiotics is used. Two to three antibiotics covering different bacteria are used single dose is given as prophylaxis and after delivery 3 to 7 days antibiotics given until the fever goes down. Generally greater than 90% take antibiotics for short period like 3days maximum of 7 days. ICU patients take antibiotics for long duration. Change of root of administration from IV to PO, when the patient starts taking food, is required but not usually carried out. Medical laboratory service is used but differentiation of type of bacteria other than bacteria involved in the contamination are not identified. Plus there is a delay on culture result report the fastest time is 3 days and waiting with patient is hard and even the result report the fastest time is 3 days and waiting with patient is hard and even the result reported usually doesn’t change the management already on progress recently though change & good results from microbiology is being reported in ICU.

**Interviewer:** How do you compare the use of broad vs. narrow spectrum antibiotics? What factors do contribute for this kind of use?

**Respondent:** Sending samples to the laboratory decrease through time because of one is sample collection technique undermined and this is any health professional takes sample and they may not have good skills so the problem starts here. The other is for long period of time in the past at tash culture results were not evaluated so the physicians send the samples but he relays on the clinical outcome. Even after the culture result is reported to the physician the culture result and the clinical presentation of the patient doesn’t match.

**Interviewer:** How do you describe patients’ pressure to prescribe antibiotics?

**Respondent:** Patients usually have fear of injection and prefer oral administration but the patients ask the brand of the medication, they have question on which country’s product is better. Also related to cost issue they prefer PO than IV. The patient lack trust on Asia’s product & choose Europe’s quality.

**Interviewer:** How do you describe the current status of antimicrobial resistance (at institutional and national level)? Should healthcare professionals be worried of this problem? How serious is the problem? Do you think it is a growing public health treat or not?

**Respondent:** The antibiotics use practices is going in a very dangerous road. This is the antibiotics that were used few years back without even identifying there result if they work or not, choosing the new medication(antibiotics) that don’t have proven value in this set up is observed from the physicians. New Broad-spectrum antibiotics are prescribed which has a huge impact on relating to antibiotic resistance development. The prescription made by interns, Residents also consultants are not in level. That is choosing antibiotic (1^st^ generation,2^nd^ generation, 3^rd^&4^th^ generation) is wild. Antibiotic that is restricted to be given as last resort for critical patient may be given to patient that has been improving.

**Interviewer:** How frequently do you encounter drug resistance organisms within your clinical practice? Do you face any challenge in your daily practice as a result of antimicrobial resistance? How does it affect your practice?

**Respondent:** Carbapenem that was introduced last year was reported to develop 14% resistance. This is very shocking. Antibiotic use has to be rational small dose & specific to the condition. If the dose is not adjusted & Root of administration not changed by considering the situation, it’s going to lead to even worse condition. Sometimes for patients that are admitted in the hospital for long time, the physicians may run out of choice of antibiotic.

**Interviewer:** What factors do you think are contributing to increasing emergence and spread of antimicrobial resistance?

**Respondent:** The Antibiotic should be restricted to selected health professionals; they have to be protected by the hospital. In oncology department the cancer medications unless the senior prescribes it, it won’t be given so this custom should be applied on antibiotics as well.

- There needs to be a protocol on use antibiotic that is revised every time that states guideline on use of antibiotics. The antibiotics used for prophylactic and treatment should be different.
- Creating awareness on how is irrational use of antibiotics is a threat in the community and showing on antibiotic on smallest dose specific antibiotic should be at first line. Before using broad spectrum consult others.
- At a hospital level there should be a committee organized that is responsible on protection of the antibiotics, policy implementation. This committee organized from patient and d/t health professional. Practical course should be given arranging field visit or hospital visit on antibiotic rational use for every level student at pre service & on service will strengthen.
- Studies should be done an antibiotic resistance profile at the hospital data base on antibiotic use and bacteria pattern.

**Interviewer:** What possible measures do you recommend for better antimicrobial resistance containment practices to be implemented in this facility? Please relate your recommendations based on its importance.

**Respondent:** Every physician that has contact with a patient has a right to prescribe as we see fit but the authority should be restricted in relation to protection of antibiotics. The existing system already states that any physician when he sees a reason to prescribe he should let his immediate senior know, or should follow the protocol. Identifying which antibiotics are available and which of them to restrict is the first step.

- Challenge: physician that has knowledge and follows updates on the western evidence and implement it in Ethiopian set up, the knowledge maybe right but the implementation is wrong. The physician can give opinion to his senior but should have the authority to implement it without discussion.
- Individual discussion should stop. The system should control it authority extremely use is the challenge.

**Interviewer:** Do you feel regular auditing have an impact on prescribers’ behavior? How would you feel if prospective audit and feedback is applied in this institution and a multidisciplinary team review and give you feedbacks on your prescribing? Can you help us understand the potential factors that might influence the implementation of prospective audit and feedback in this institution? How do these factors become barriers?

**Respondent:** The auditing & feedback intervention can be organized based on the hospital’s capacity. The person that recommends & the person whose executive should work hand in hand.

- Challenge is the trust lost through time on microbiology laboratory results the microbiology laboratory should be standard and the result should be reported as fast as possible. Sometimes the samples get lost, the sample collection may not be appropriate so work needs to be done to improve and strengthen the microbiology laboratory.
- Other is inflection prevention which is a pillar to antibiotic stewardship infection is transmitted form patient to patient, patient to doctor, doctor to patient so inflection prevention implementation should be there.
- The recommending team and the executive body should identify the best way of communication. The implementation should form the higher up that is most senior physician so form top down movement is better. The senior physician should be engaged.

Antibiotic use is everyone’s problem so there is no reason why the senior or other physical won’t be engaged. Constant antibiotic availability should be there/ the recommendation and implementation to exist. Specially the basic antibiotic should be available 24/7. If antibiotic is available at one time and lost in the others. This breaches the trust.

**Interviewer:** That is the end of my interview, is there anything you would like to add?

**Respondent:** Antibiotic is like a bullet it could kill or protect. So everyone should be aware of its side effect. “When you use it, use it wisely for the right indication”. Media coverage should give attention to antibacterial. Image should be created that could also show the dangerous side.

**Thank you for your time!!!**

=================================================================================

## Physician KI#18

Age: **26 years**

Sex: **Male**

Department: **Oncology**

Staff Position: **Resident [R1]**

Total work experience: **2 years**

Number of patients treated per day: **20 patients**

Number of patients with ABX prescription per day: **7 patients**

Duration of interview (minutes): **20:01 Minutes**

**Interviewer:** Ok, thank you! Let me first ask you your opinion on the current use of antibiotics, I mean how do you describe the overall utilization of antibiotics in this hospital and in the country in general?

**Respondent:** Most of the time we prescribe empirically. We don’t have culture, there is no hospital specific study and we don’t have guideline on how to use antibiotics. So overall, it is very difficult to say the use of antibiotics is appropriate but on the other hand it is difficult it is not right. I mean decisions are made by individual experience not based on guidelines.

**Interviewer:** So do you think there are times where broad spectrum antibiotics are used where the narrow can work equally. And also what about under or overuse of antibiotics?

**Respondent:** Yes, these are common problems particularly for RTI. We see antibiotics are commonly prescribed for viral infection plus when we can manage infection with narrow spectrum we usually prefer to prescribe the broad-spectrum antibiotics. These days we see Amoxicillin and ceftriaxone are being prescribed for almost all patients even without knowing its response and its resistance profile. It commonly happens that antibiotics are prescribed while they are not necessary to manage the disease condition and at the same time some of the infections can be well managed by narrow spectrum antibiotics but mostly broad-spectrum antibiotics are prescribed. I can say inappropriate use of antibiotics of these kinds is a common especially at the lower health facilities, clinics or health centers.

**Interviewer:** Ok, you were telling me some of the reasons like lack of culture and sensitivity test, lack of institutional guidelines and up-to-date evidence on resistance pattern. But what other factors do you think contributed for this problem?

**Respondent::** The other thing is there is lesser attention given to the problem of resistance. I mean the trainings at the UG level and other educational levels doesn’t address the resistance problem very well. So unless due attention is given at the education, such gaps could also contribute for that.

**Interviewer:** What about the impact of others like medicine availability? Do you think it would have an impact?

**Respondent:** Yes, unavailability of medicines has a strong influence. For instance, when I want to treat my patients with narrow but sensitive antibiotics, sometimes because of scarcity I was forced to prescribe broad spectrum antibiotics or to prescribe multiple antibiotics at once. **At times, I prescribe antibiotics knowing that they are resistant because of shortage or scarcity.**

**Interviewer:** Let see the influence of patient to prescribe or not to prescribe antibiotics. How do you describe patients’ pressure to prescribe or not prescribe antibiotics to themselves or their children? How about their preference of IV and PO routes?

**Respondent:** Particularly in private health facilities, you can see patient pressure to prescribe more antibiotics but it is not as such a common problem in public hospitals. Yes there are also customers who resist to take antibiotics but very few in number. Some patients also prefer to IV over PO and you can see they are not happy in you prescribe PO antibiotic and at times they ask you to change it to IV. So, in general there such demand from the patient but their influence to prescribe depends on individual character of the care giver. I mean there are physicians who can resist to obey their demand but there are also few caregivers who kneel down to the demand of the patient especially this is common in the private health facilities.

**Interviewer:** But why do you think so the patients need to give them IV instead of PO antibiotics?

**Respondent: Most of these patients think IV are more potent, more effective than PO and expect to get immediate and better outcome in shorter time possible.**

**Interviewer:** So it is related to the perceived potency of the medicines.

**Respondent:** yes, it is related to the potency and most of them think they can get better effect from IV. They believe IV is more potent.

**Interviewer:** If this is how antibiotic utilization looks like, what about the resistance pattern? I mean yes you were telling me the culture and sensitivity is not common but if you see it from your experience and with the few culture findings, how do you describe the current status of antimicrobial resistance?

**Respondent:** Although we don’t have a comprehensive studies, from my clinical observation and looking into patient response to antibiotics, I can say resistance is now a big challenge. **Many patients are now not responding to common antibiotics. For instance, ceftriaxone, although there is no study it is prescribed here and there, currently its response is not good. But we don’t have studies of its magnitude and pattern in the hospital.**

**Interviewer:** OK, from your experience, which pathogens do frequently encounter as resistant? How do you see the effect of AMR your daily practices?

**Respondent::** Particularly UTI, fluoroquinolones these time are frequently prescribed for UTI but from my experience the response is becoming very poor. Failure rate of these drugs is increasing. With regarding to its impact on my daily practice, I can say you it is limiting my choice of antibiotics and even the next choices I have is very expensive where my patient can’t afford it.

**Interviewer:** you were mentioning the increase in resistance of AMR, in your opinion what factors do you think are contributing to increasing emergence and spread of antimicrobial resistance?

**Respondent::** As I mentioned earlier, we don’t have pathogen directed guidelines, no culture and sensitivity results and antibiogram. Thus, antibiotics like ceftriaxone, amoxicillin are prescribed to any patient because it is very difficult to clearly identify which pathogen it is, also its susceptibility is not known and we prefer to prescribe broad antibiotics which ultimately increase resistance.

**Interviewer:** So laboratory problem is …..

**Respondent:** Yes poor lab infrastructure for instance we send sample to lab but some time they may not have the disc, I mean the scope of coverage of susceptibility test is not sufficient. Since we don’t have that test, it is difficult which bacteria is the cause. Another thing is there is poor growth in the laboratory.

**Interviewer:** But how do you see the culture of sending samples to laboratory and in general the culture of using laboratory set-up as input for clinical decision.

**Respondent:** There is demand to get results of culture and sensitivity but the problem is there is delay in reporting the finding. Especially in large hospitals like ours, most patients are critical which needs immediate intervention and you know the timing of antibiotic initiation highly matters**. But the problem is there is delay and mostly we receive results after three weeks and even for the preliminary results delayed up to a week. So the only option you have is to start with broad spectrum antibiotics and by the time the laboratory send us the result the patient might be discharged or possibly gained more complication.**

**Interviewer:** So you think the low utilization of culture is this. But even though result is received late, what about issues related to its quality? Do you think such issue are also have an impact?

**Respondent:** I don’t think there is resistance or problem to send samples. But the problem is only the delay and availability of some tests. But there is no concern in the quality.

**Interviewer:** Seeing on the current use of antibiotics and magnitude of resistance, how do you describe the current effort to contain antimicrobial resistance?

**Respondent:** I don’t think there no such a concerted effort to curb the problem. Most of the time we start empirically and the use of culture is minimal due to some reasons mainly delay of results.

**Interviewer:** So what do you suggest should be done to improve that?

**Respondent:** I think first there should be studies in antimicrobial resistance pattern and then based on the findings we can develop institutional guidelines which can direct our prescribing. Second, it is important to prepare systemic based pathogen lists and another thing is it is better to shorten the delay in reporting culture and sensitivity test at least the preliminary finding should be sent within 24-48hrs. That we can confidently change the empiric treatment as per the finding report. The other thing is, issues related to antibiotic use, resistance should be given more attention at the UG educations. This education should not be limited to medical school but to all health care providers.

**Interviewer:** What do you think if restriction policy is introduced in this hospital [question was forwarded after discussing what restriction is meant about]. **How do you see it and what kind of challenges do you anticipate?**

**Respondent:** It essential to implement such monitoring of antibiotic use and I think it will decrease over prescription of antibiotics, at least prescription of antibiotics here and there would be minimized. Moreover, as the authorizers are ID specialist with more experience in the area, the judgment would much better. Challenge is, will they be available. I mean will the ID physician be available at all time but if not will have a negative impact on the patient outcome. Patient might not be able to get treatment on time. **So in this case, we need to devise mechanisms/system of authorization to handle such circumstances for instance we can permit telephone authorization or other means but if signatures are a must then I think there will be a problem. So main challenge is unavailability of the physicians selected for authorization and to this end we know that they are few in number.**

**Interviewer:** What about other factors like autonomy. Do you think physicians will accept or resist the implementation?

**Respondent:** I think there will be such feeling as if autonomy of the care provider is breached. But if we educate the prescribers on its benefit, I am sure they will accept it. So agreement and consensus is important.

**Interviewer:** OK, if these are the challenges what enabling factors do you think there to implement the restriction policy?

**Respondent:** One of the enabling factor is the presence of hierarchy system in teaching hospitals (starting from intern, resident to consultant).

**Interviewer:** Another antimicrobial stewardship intervention is the prospective audit and feedback [description of the intervention given to the interviewee]. So like we discuss for restriction, how do you see if this intervention is implemented? What are the challenges and enabling factors?

**Respondent:** **I think this one [prospective audit and feedback intervention] is much better that restriction because it is based on cooperation and willingness.** Again providing feedback is also very good as it makes the prescribing physician to aware of the resistance pattern, sensitivity pattern and other issues given during the feedback. So it is will be better and sustainable. But the challenge might be shortage of professionals who can manage this auditing. Can we afford the man power. Another thing is the absence of such team work practices from undergraduate and that might be a challenge to bring different professionals to work together.

**Interviewer:** Ok, like wise what enabling factors do we have to implement this intervention.

**Respondent:** **The issue is a concern of everyone and equally everyone is ready to learn. So, unlike restriction here there will not be resistance from the prescriber.**

**Interviewer:** That is what I have and thank you so much.

=================================================================================

## Physician KI#19

Age: 37 years

Sex: Male

Department: Internal Medicine

Staff Position: Consultant Physician (ID Specialty)

Work experience: 13 years

Number of patients treated per day: 15 patients

Number of patients with ABX prescription per day: 15 patients

Duration of interview (minutes): 73:35 minutes

**Interviewer:** Ok, thank you for your time. Let’s first discuss about antibiotic use. So, in general how do you describe the current use of antibiotics in Ethiopia and particularly in this institution? How does it look like.

**Respondent: *Well, antibiotic use in our country is totally off the track and unacceptable. I don’t think we are using them appropriately when I say this; first at the community level the culture of using antibiotics without prescription seems a long time culture/practice, it is a chronic problem practiced widely. And with regard to health professionals, most of them don’t know what type of antibiotics should be given, when and where, so we see antibiotics prescribed unnecessarily resulting in overuse of antibiotics and as compared to other countries probably we might be ranked as highest antibiotic consumers.*** So as to me, inappropriate use of antibiotics is widely practiced.

**Interviewer:** Which types of inappropriate uses are most prominently available and why?

**Respondent:** Let’s see it in two categories: (1) the use of antibiotics outside the hospitals i.e. at the community level also including the outpatients and (2) antibiotic use at the health institution level (inpatients). The use of antibiotics in OPD and general community is almost similar. These time, there are very commonly known antibiotics to the community like ***Ampicillin, Amoxicillin, TTC, metronidazole, albendazole. Such drugs are now considered by the community as over the counter products which actually don’t need prescription.*** Every member of the community wanted to get these drugs. The worst thing from my experience (my informal discussion with pharmacists and community members) **is that the public is considering these antibiotics like antipain.** For instance, almost all of our out patients are now telling us that they are taking Amoxicillin and when we ask them why they are taking it, they responded us that they are taking it **as antipain for joint pain, and headache as antipain.** I mean this this due to lack of knowledge. Also, they mostly took it for abdominal cramps and diarrhea. I mean self-treatment is good for selected medicine but the problem is they don’t know what to take when and for which disease. ***At the same time they started taking the drug but they stopped/discontinue to take it immediately after they get slight relief from the first symptom probably if relieved from pain or cough, they took them PRN like any antipain drug taken.*** This is the problem with the general public. In the outpatient ***physicians are overtreating.*** The reasons are one it is partially related **to *lack of knowledge of prescribers*** but it is also resulted due *to* ***lack of proper diagnostic instruments or test tools to correctly identify the infection*** e.g. being un able to identify which is viral and which is bacterial caused infection; also when patient come to health facility they came here expecting treatment ***[patient pressure, tendency to get prescription] which affect prescribers decision as they wanted to satisfy and handle patient demand. As a result, there is over prescription. There are common infection which are prone to over prescribing*** such URTIs (like cough, pharyngitis, bronchitis infections are resulted due to viral or bacterial toxins where antibiotic have no benefit [they are self-limiting] but when the patient are presented with such complaints then most physicians tend to prescribe antibiotics. ***This are usually wrong treatments. The major primary reasons for overprescribing are b/c there is no test to clearly identify the infection types and there is also high pressure demand to prescribe where physicians want to fulfill.*** Others like diarrhea are self-limiting and can be managed without antibiotic. But the problem of over prescription for

The major problem is antibiotics don’t have immediate problems unlike other drugs like anticancer. In cancer even knowing what type cancer and what treatment is needed, physicians don’t tend to immediately prescribe the anticancer drugs b/c their side effects are visible at times it can be more severe than the disease itself. ***But antibiotics, except few of them, they are considered as the safest medicine and there is no hesitation to overprescribe. But to my understanding this abuse of antibiotics could have unprecedented effect in the future, they are silent killers.***

From impatient perspective; the problem seems similar to OPD patients. ***One, diagnostic services are not available and done appropriately to correctly identify the infection. Second, the antibiotics are not used based on international guidelines or scientific recommendations but rather based on personal experiences or patient demand.*** For instance, international guidelines recommend a duration 5-7 days IV to treat pneumonia but some time the patient demand for longer duration b/c patients may feel some of the symptoms are persist [b/c patients may not relieve from all symptoms]. ***So, prescribing for longer periods [by some physicians] which might goes up to 20 days might be due to lack of knowledge but there is also patient influence.***

***Physicians prescribe antibiotics based on availability of antibiotics;*** I mean when some lifesaving first line medicines are not available then prescribers could prescribe what is available even if that is not the best option. But science doesn’t work like that. ***Un fortunately the sad reality is, it is the resource which is guiding our practice, but not science or evidence which is I think is also the truth in many developing countries.*** I am saying from experience I mean in the past few years, WHO treatment guidelines were not based on science/evidence based on resources. The absence of infectious disease diagnostic tools is a systematic problem and all stakeholders including the FMOH should work had to improve the diagnostic facilities.

**Interviewer:** How do you see the use and practice of culture and sensitivity test in your practice?

**Respondent:** I have long experience starting from my UG to fellowship training and when I see the practice over the years, the capacity of microbiology was relatively good. ***But after time, it becomes very poor which I don’t why. Currently, the possibility of getting culture and sensitivity test results is very minimal.*** There is no disc and I think practitioners become desperate about that. The use of culture is minimal but even we use I have seen people ***complaining about the quality of findings. they say, over the years they have never seen positive result. For instance experience from other countries indicates that, in patients with infective endocarditis on average more than 90% of them do have positive blood results. So you can see patients with such problem are identified using blood culture but when you see in our case the most senior physician will tell you that over couple of years, they have never obtained positive culture result for infective endocarditis.* So, when they say this you can imagine how desperate it is and how can you trust the other results.** To identify the reasons as an ID physician I tried to explore the reasons. The lab personal mentioned that the **problem specimen collection, adequate quantity of specimen[blood], specimen is not sent immediately to the lab which may have problem in growth as most of them died before they reach in the lab. They also complained that patient took antibiotic prior to the data collection but the microbiology department is not informed on the type of antibiotics taken by the patient. We also observe that the techniques used in the microbiology is old which need a very strict QC, poor infrastructure and knowledge gap; poor communication of results are some of the problems of the department.** Even when there was positive culture, they may kept it instead of reporting*.* ***So, probably this unrealistic practice discouraged physicians to use the service.*** I think the lab professionals have knowledge gap and even with that limitation they don’t communicate you their result. The practice of infectious disease is highly dependent on microbiology results but unfortunately, the infectious disease unit/ internal medicine and department of microbiology are disjointed. **The attitude of working together or working in team work is not a culture as you know. It is very difficult to work in team, which is chronic disease but you can’t treat it using medicines.** But if you work collaboratively it is possible to improve the services and we are seeing this with the small projects but that is not enough. Scope of the services has to be widened and after all this means we need financial support to do this. I think strengthening the microbiology should be the priority but otherwise it is impossible to discuss about other interventions. **I think, antibiotics are public goods, if we used them wisely then they will give us more benefits for longer time but if not, they will be lost and we will get lost.** Science is developed due to development of antibiotics development, I mean it is **very difficult to do other medical treatment treats such as cancer or surgery without antibiotics.** So, it is not only about infections disease but we are putting other treatments at risk.

**Interviewer:** From your experience, how do you describe antimicrobial resistance. How do you see it overtime?

**Respondent:** Well, generally antimicrobial resistance a carbon copy of its utilization/consumption. Antibiotics are overused for many reasons and as a result pathogen are now developing resistance. I think this has been proven in many countries and it is also true from science. So, many antibiotics such as beta-lactams, penicillin, cephalosporin are highly resistant. Let me tell you my experience, carbapenems are officially imported in the last two to three years and you can imagine that these antibiotics are not available in Ethiopia and their level of resistance would be low. But after three years of use, these times we repeatedly [not one or two times] see carbapenem resistant pathogens. I think this is an indication of how serious it is. So, the problem is a huge concern which is also true in other developing countries.

**The reasons:** one antibiotic is not prescribed correctly and even after they are prescribed the inappropriate use is a big challenge where patient discontinue treatment; took them for unnecessarily longer duration; prescribed for unnecessary conditions. We have many problems.

**Interviewer:** Ok, you mentioning to me the reasons for increasing resistance. Can you tell me other reasons.

**Respondent:** So, it is interrelated. The public abused antibiotics, use of antibiotics at the OPD is inappropriate, hospital antibiotic prescribing and use is not right; and it is difficult to correctly diagnose the infection. So, all these factors are promoting resistance. Another thing is, the easy transmission of infection and resistant pathogen [congested and it is open ward system where there is free access to wards] and generally the poor infection prevention practice mainly due to limited resource and poor awareness are contributing to increase development and spread of resistance. Isolation practice is also poor. So at times we might be in a position where all options are limited.

**Interviewer:** So if the problem is to this extent, what major interventions do you recommend.

**Respondent:** The primary intervention should be strengthening the microbiology services. There is no other intervention stronger than this. You can’t think of strong infection diseases practice without strong microbiology. ***Another best intervention is, increasing properly trained motivated, respecting CRC attributes of professional including ID physician, nurses, pharmacists and other professionals. This lead us to the necessity of establishing strong multidisciplinary practice. So, antimicrobial stewardship program could have a lot of benefits. Hence, antimicrobial stewardship program with properly training health professionals should be realized.*** This are the major things but if you ask me to mention others I can see the importance of infection prevention practice and others. But others interventions can be addressed if the above mentioned two interventions are fulfilled.

**Interviewer:** you were telling me about antimicrobial stewardship program. How do you see its feasibility and importance? What barriers and enabling factors do you anticipate.

**Respondent:** Let me start by mentioning its benefit. ***First, it is the most cost-effective program.*** It can prevent the occurrence of serious infections and minimize the healthcare cost. I started by this b/c many mangers are interest on financial benefits, so the fact that it minimizes cost can guarantee you winning financial assistance; **I mean you can show them that if they give you some amount of money then the return of investment is higher. So, the economic value is high.** Secondly, you can think long term effects of antibiotics i.e. we may not be common to see an ***immediate or short-term effect of inappropriate use of antibiotics but if we continue to misuse antibiotics then the future is dark and frightening. I mean everybody is now talking about post-antibiotic era*** where the benefits we enjoyed from antibiotics will no more be obtained for granted. We may reach where we can’t defeat antibiotics, can’t treat infections and ***this will take us back to 50-60 years in history and will affect our growth. So, if a team [ASP] is established to help us to responsible use antibiotics and alleviate this problem it can be championed and I think it is a program with no other choices.***

**Interviewer:** What about the challenges?

**Respondent: challenges?** Frist at this point, the program has to be implemented properly and to do in that manner, it **needs resource**. I told you earlier, it needs ***trained personnel, microbiology services has to be strengthened and*** other infections disease related programs like IPP has to be strengthened. Up front there should be investment. The second problem is, the confusion it may create among prescribers i.e. in general most of us don’t what to be told what to do so usually there ***is resistance from the staff*** it is not easy to convince fellow professionals. Although resistance could be an issue but it can be solved by convincing them using evidences i.e. aware them about the impact of the problem]. The third barriers will be the poor **infrastructure of microbiology**. One of the core inputs for ASP is microbiology results which can inform you the most prevalent pathogens in the hospital and their susceptibility to antibiotics. Institutional antibiogram should be prepared and updated periodically like yearly or 6 months. ***For Institutional antibiogram is like a map for treatment as the usual map guides you where to go about or otherwise you got lost and that is the same for treatment of infections. But otherwise it is impossible and doesn’t work to making auditing, to say don’t prescribe or change this antibiotic or implement antimicrobial stewardship without having such evidence. I think preparation antibiogram could be one of the difficult task.***

**Interviewer:** If these are the barriers, what do you think the enabling factors are for the implementations?

**Respondent:** I think the exiting initiative and the attention given at national and international community is a very good thing. ***It is now a number one global priority agenda and that by itself would be helpful to get the required funds for its implementation.*** The previous WHO general assembly had a pertinent deliberation for countries to have their own antibiotic policy and strategies, to encouraging research and solicit funds. So, this high focus is an opportunity to work more and we need to grasp this opportunity. In line to this, stakeholders are now giving more attention to this area and we can get funds to improve our microbiology and implement the intervention. Another thing, is the awareness is increasing well in the country like in Gondar University. So I think if can work together, it is possible to do efficiently implement the program. The FMOH is also giving more attention by preparing strategic plan, and implementation guidelines for ASP, which I think is great. Most importantly the huge interest of non-treating health professionals is good thing. We know that we have a problem to work in team, to me it is a disease although we can’t treat it using medicines. This might be for the reason to find glory and recognition. There is also problem vision alignment and if don’t align it, we can’t achieve our goals. I mean there is an interest and it is our agenda but it seems we miss the lane [you can imagine what will be the impact if the train misses the path…].

**Interviewer:** so you think there is problem of working in team?

**Respondent:** Of course, yes! Although it can’t be generalized to all staff, working in team is a big challenge. But at this time, it seems there is an interest from different professionals. So if it is started at some point and the ball starts rolling, I don’t think it would be a problem. It is not without challenge but if those who were resisting saw a visible impact, then I think they can change their mind and joins us later.

**Interviewer:** In your opinion if we want to implement restriction and prospective audit and feedback, how would you recommend us to do? Do you think both should be implemented at the same time or should be implemented one after the other? Can you help understand how they should be implemented?

**Respondent:** I think you can have a mix of antimicrobial stewardship program and both can be applied in Ethiopia. To implement the ASP, first the microbiology should be strong and then we you have to prepare the antibiogram to be updated periodically. This antibiogram can be used as input to prepare formulary, a menu for antibiotics. Then you can prepare antibiotics to be restricted and also you can use it as an evidence for auditing. I think both restriction and auditing are feasible for us. There are some antibiotics that they should be restricted. In Ethiopia *staphylococcus aureus* is common problem and vancomycin can be taken as first line drug due to fear of the MRSA is increasing [ due to fear that the other first line drugs like cephalosporin, ampicillin might become resistant] but sadly you can’t see Linezolid to be considered as first line drug but the trend we are seeing now is Linezolid prescribed as first line in OPD. So, if you ask me what to do, to me Linezolid shouldn’t be prescribed with clearance/authorization or shouldn’t prescribed without evidence. Carbapenems are another broad spectrum, potent or last-ditch antibiotics, so unless it is a must to prescribe we shouldn’t consider it as first line. I mean until evidence is obtained or you got clearance for those restricted medicines, the patient can stay at other first line antibiotics. ***So you can implement restriction for selected antibiotics and the rest of the antibiotics can be addressed through prospective audit and feedback scheme.*** We don’t have firsthand evidence in our country, but I think both can work in our set-up. Global evidence indicates the same but the prospective audit works much better and it is also sustainable. I think in the first instance it more of politics, so if you can convince the senior and avoid the fear that it might took their autonomy to prescribe, then it will work. If we design the intervention appropriately, then it can work. Even in the absence of such ASP team, I have been consulted even by senior physicians ***which is an indication that there is a need to such intervention and there is informal networking***. I think I am optimistic, there were times where I got desperate but now it seems it is getting attention. It is now a choice but it becomes a must to do. We have reached to the point of return but if we don’t act then the consequence is unbearable. My suggestion is during the implementation we have to make sure we have ***customized and make it locally applicable*** intervention.

**Interviewer:** the plan is to explore the barriers and enablers for the interventions through qualitative which I am doing it now and there is also quantitative survey. In addition, we do have prospective data obtained from inpatient including the antibiotic use and pathogen susceptibility. So based on these information we will design our intervention. So, your inputs are very valuable and helpful for our purpose. Thanks for your time and your willingness.

=================================================================================

## Physician KI#20

Age: **57 years**

Sex: **Male**

Department: **Oncology**

Staff Position: **Consultant Physicians**

Total work experience: **26 years**

Number of patients treated per day: **25 patients**

Number of patients with ABX prescription per day: **5 patients**

Total Interview time (duration): **20:37 Minutes**

**Interviewer:** Ok, my first question is on antibiotic use. So from your experience, how do you describe the overall utilization of antibiotics in this hospital and in the country in general?

**Respondent:** As you know to do appropriate prescribing you need evidence or information of microbiology, you need the anatomical description of the pathogens which pathogens are more prevalent, where are the patients admitted as it would be helpful to identify the type of possible infection acquired e.g. pseudomonas is high in surgical and others too. So, if you start empirically, you need to make to the most possible empiric. That should be the case.

**Interviewer:** How about the real practice of prescribing antibiotics?

**Respondent:** In the real practice, ***most of us prescribe broad spectrum antibiotics*** especially ***if s/he is a critical patient b/c we don’t want to a risk***. Since the ***possibility of acquiring multiple infection or resistance is so high***, we prescribe ***multiple antibiotics and most of them broad spectrum antibiotics which have wider coverage***. The is ***overuse of antibiotics***. In principle, yes we have to use the culture and sensitivity test but practically not b/c there is ***no strong microbiology laboratory.***

**Interviewer:** How do describe the culture using microbiology in this hospital? Do physicians sent samples and how about the quality of reported findings?

**Respondent:** Previously we were using the microbiology for culture and sensitivity but these times ***I have doubt on the availability of the tests we need them to do***. Anyways even when we sent samples before we start antibiotics treatment, there ***a delay of reporting. It took them too long time to report the results, usually more than 72 hours and in that regard we don’t want to take the risk.*** So, usually prescribe empirically. What I am telling you is about myself and residents. But even though I don’t have detail information, I believe it is not different with other physicians too. ***We don’t strictly use the culture and sensitivity test for prescribing.***

**Interviewer:** Ok, how about the status of antimicrobial resistance? how do you describe the current status of antimicrobial resistance? Do you think it is a growing public health treat or not?

**Respondent:** I remember when TTC become highly resistant but after sometime I mean when its use ***decreased or stopped for almost 4 to 5 years, again I can see TTC is now showing good sensitivity* *[de-escalating of antibiotics use].*** These time *ceftriaxone is being use for everything including cough. As a result, ceftriaxone rate of resistance reached up to 70 to 80%.* It is simply empiric treatment, there ***no effort to differentiate whether it is viral or bacterial causes especially this is a common practice. T***hese all are contributing to increasing. I can also see this from some researches.

**Interviewer:** What do you think are the major factors for increasing antibiotic resistance?

**Respondent:** First, ***inappropriate prescribing*** of antibiotics; I mean ***antibiotics are prescribed left and right.*** There major is *overuse of antibiotics especially in the private health facilities*. If patient is admitted especially in private facilities, almost everyone took antibiotics. ***Sorry to say this but there are professional who think that any admitted patient should take antibiotics, which is bad practice. When patient is in normal saline, they think the patient should start antibiotics. So there is no good, evidence based judgement in selecting antibiotics increased inappropriate use and thus increase resistance.***

**Interviewer:** How about other factors like IPP?

**Respondent:** Yes, from my experience ***there is problem of strictly follow the infection prevention principles*** like hand rubbing but I am not sure why and it needs study.

**Interviewer:** How do you feel the existing effort to tackle the problem of AMR?

**Respondent:** I think resistance is increasing problem that could negatively affect all of us including our children and the country at large. So, our antibiotic usage should be restricted and we need to use centralized information. The highest problem of antibiotic use is in private hospitals, so private hospitals should follow appropriate antibiotic prescribing practices.

**Interviewer:** I would like to hear your feeling on the implementation of antibiotic restriction [the interviewer briefed how restriction policy works]. How would you feel if it is applied in this institution? Do you think it is feasible and important?

**Respondent: *Selected antibiotics can be restricted and I have seen this working well from my experience in other countries like south Africa and others.*** For instance at that time during my training, physicians were not allowed to prescribe Augmentin unless it is authorized by microbiologist. There was microbiologist on call and the physician will discuss with the microbiologist and the pharmacists will no dispense unless the microbiologist put a signature. ***Even the private community pharmacy call when they see such prescription. But here, how would you control it if a prescription is ordered from Jimma as patients can purchase it in Addis Ababa, so need to have mechanisms for controlling such practices.*** So if antibiotic restriction is implemented, we can have good antibiotic usage and we can halted the increase pattern of resistance. So as a practitioner I am in favor of its implementation. Because, currently I am observing the high utilization of vancomycin and meropenem which the last choices of drugs, I am discussing this with residents. ***Even ceftriaxone resistance is about 80% but you see now ceftriaxone being prescribed right and left but I don’t think it is effective. So unless, we precautions the fate of other drugs will be same as ceftriaxone.***

**Interviewer:** What are the barriers and enablers do you anticipate for this intervention?

**Respondent:** I am afraid that due to individual variation there might be nonacceptance or resistance by prescribers but it is better to start though. Even if we can get 70 to 80% acceptance from the physicians that is good as it can be improved overtime.

**Interviewer:** What do you think if prospective audit and feedback [the interviewer briefed how prospective audit and feedback works] is implemented in this institution? Do you think it is feasible and important?

**Respondent:** ***If it is implemented correctly, yes it is beneficiary.*** Yes, it has challenges to implement. One is, there is lack of strong culture and sensitivity set up and then with the interest of treating our patients we opt ***to use voracious antibiotics. So*** it might be a problem to abide with the appropriate principles. *But I don’t think there will be huge resistance*. Because, first it is understanding I mean if prescribers believe that resistance is a problem and as a country it will affect our practices, there will not be problem of acceptance. You know the microbiologist may not know things that I know and the same is true for me, so if the microbiologist is with the team we have to know that he is adding some information for better management of the patient. Otherwise it is a failure. So I hope this practice gets acceptance from the central level and started to be implemented.

**Interviewer:** What is your recommendation on the time of implementation? Should both implemented concurrently or one after the other? How and why?

**Respondent:** I think the antimicrobial committee is good but before that we have to improve the capacity of our laboratory. Sometimes, there are reports which are very difficult for decision. For instance, you know they sent you a result of 48,000 WBC count and you wonder if it is leukemia or its reaction but when it is done again they reported 5,000. So there is no trust in the quality of the findings. ***So, I recommend both of the intervention are implemented together at the same time.***

**Interviewer:** That is the end of my interview, do you have additional ideas to share with me?

**Respondent:** I think this should not be considered only for educational but it has to be institutionalized it to the existing system. We can learn from other countries that they managed to achieve lower resistance pattern. This has to be communicated to relevant authorities and effort should be organized from the central level.

**Interviewer:** Ok. thank you.

=================================================================================
